# Identification of fibronectin type III domain containing 3B as a potential prognostic and therapeutic target for pancreatic cancer: a preliminary analysis

**DOI:** 10.1186/s40001-024-01823-6

**Published:** 2024-04-05

**Authors:** Yizhi Wang, Yang Kong, Qifan Yang, Cheng Zhong, Dongkai Zhou

**Affiliations:** Department of Hepatobiliary and Pancreatic Surgery, The Second Affiliated Hospital, Zhejiang University School of Medicine, Hangzhou, 310009 China

**Keywords:** FNDC3B, Pancreatic cancer, Prognostic value, Immune infiltration, Bioinformatic analysis

## Abstract

**Background:**

Fibronectin type III domain containing 3B (FNDC3B), a member of the fibronectin type III domain-containing protein family, has been indicated in various malignancies. However, the precise role of FNDC3B in the progression of pancreatic cancer (PC) still remains to be elucidated.

**Methods:**

In this study, we integrated data from the National Center for Biotechnology Information, the Cancer Genome Atlas, Genotype-Tissue Expression database, and Gene Expression Omnibus datasets to analyze FNDC3B expression and its association with various clinicopathological parameters. Subsequently, Gene Ontology and Kyoto Encyclopedia of Genes and Genomes, along with Gene Set Enrichment Analysis (GSEA), single sample Gene Set Enrichment Analysis (ssGSEA) and estimate analysis were recruited to delve into the biological function and immune infiltration based on FNDC3B expression. Additionally, the prognostic estimation was conducted using Cox analysis and Kaplan–Meier analysis. Subsequently, a nomogram was constructed according to the result of Cox analysis to enhance the prognostic ability of FNDC3B. Finally, the preliminary biological function of FNDC3B in PC cells was explored.

**Results:**

The study demonstrated a significantly higher expression of FNDC3B in tumor tissues compared to normal pancreatic tissues, and this expression was significantly associated with various clinicopathological parameters. GSEA revealed the involvement of FNDC3B in biological processes and signaling pathways related to integrin signaling pathway and cell adhesion. Additionally, ssGSEA analysis indicated a positive correlation between FNDC3B expression and infiltration of Th2 cells and neutrophils, while showing a negative correlation with plasmacytoid dendritic cells and Th17 cells infiltration. Kaplan–Meier analysis further supported that high FNDC3B expression in PC patients was linked to shorter overall survival, disease-specific survival, and progression-free interval. However, although univariate analysis demonstrated a significant correlation between FNDC3B expression and prognosis in PC patients, this association did not hold true in multivariate analysis. Finally, our findings highlight the crucial role of FNDC3B expression in regulating proliferation, migration, and invasion abilities of PC cells.

**Conclusion:**

Despite limitations, the findings of this study underscored the potential of FNDC3B as a prognostic biomarker and its pivotal role in driving the progression of PC, particularly in orchestrating immune responses.

**Supplementary Information:**

The online version contains supplementary material available at 10.1186/s40001-024-01823-6.

## Introduction

Although the incidence rate of pancreatic cancer (PC) has been gradually declining, it continues to pose a significant mortality risk with an estimated 51,750 deaths projected in the US by 2024 [1]. However, given China’s larger population base, it is anticipated that the burden of PC will be even pronounced in China [2].

Radical resection remains the primary approach for treating PC, however, its efficacy is limited. Comprehensive therapies such as chemotherapy, immunotarget therapy, and photodynamic therapy have been employed to manage disease recurrence and metastasis in PC patients, but their effectiveness remains suboptimal [3–5]. PC is characterized by abundant deposits of extracellular matrix (ECM), and the refractoriness of PC can be attributed to abnormal collagen fibers expression to promote tumor migration and invasion which may lead to an early recurrence and also increase PC resistant to treatment. A previous study indicated that knockdown of type I collagen homotrimers enhanced T cells infiltration and increased the efficacy of anti-PD-1 immunotherapy [6]. Additionally, the structural organization of collagen can indirectly influence therapeutic efficacy by regulating drug delivery [7]. Fibronectin 1 (FN1), an essential component of ECM has been found elevated expression in PC tissues and it can exert regulatory effects on ECM remodeling as well as tumor metastasis [8]. Notably, FN1 has recently been implicated as a mediator facilitating oncogenic signaling through MACC1 pathway activation leading to distant spread of PC [9]. Besides, FN1 expression in extracellular vesicles were also believed to induce gemcitabine resistance of PC [10].

Fibronectin type III domain containing (FNDC) proteins are characterized by the presence of at least one conserved fibronectin type III domain and comprise 11 proteins in Homo sapiens. Among them, FNDC3B, also known as FAD104, has been previously reported to regulate adipogenesis and osteoblast differentiation [11]. Recently, an increasing number of studies have elucidated the mechanisms underlying the role of FNDC3B in tumor progression, particularly in tumor invasion and metastasis. In cervical cancer, a comprehensive bioinformatic analysis revealed that differential FNDC3B expression could distinguish between different stages of cervical cancer, with high FNDC3B expression indicating a shorter OS and DSS. Furthermore, FNDC3B demonstrated predictive value for cervical cancer progression [12]. Additionally, activation of PI3K/mTOR signaling pathway mediated by FNDC3B was found to induce invasion and metastasis in cervical cancer [13]. Moreover, bioinformatic analysis indicated FNDC3B as a promising prognostic and immunotherapeutic biomarker in glioma and a target of several miRNAs [14–17]. Moreover, it has been observed that mRNA 3′-UTR shortening enabled FNDC3B to evade miRNA-mediated gene repression. Remarkably, the overexpression of FNDC3B shortened 3′-UTR form exhibited more potent malignant behaviors compared to its longer counterpart in nasopharyngeal carcinoma through activation of  Wnt/β-catenin signaling pathway [18]. Despite exhibiting oncogenic characteristics in various tumors types, the association between FNDC3B or other members within the entire family of FNDC proteins with PC remains largely unexplored. In the present study, we aimed to investigate the expression, prognostic value, biological function, and immune infiltration status of FNDC3B in PC to explore its potential as an efficiency biomarker of PC treatment.

## Material and methods

### Data obtainment and preprocessing

The mRNA expression data of FNDC3B and clinical characteristics of PC patients, including 178 tumoral tissues and 4 normal tissues (Data Type: HTSeq-TPM) were retrieved fromthe prestigious TCGA database (http://portal.gdc.cancer.gov) (http://tcga-data.nci.nih.gov/tcga/). Expression levels of FNDC3B in normal pancreatic tissues and other organs were obtained from the GTEx database and the National Center for Biotechnology Information (NCBI) Gene database, BioProject PRJEB4337 (http://gtexportal.org) (http://www.ncbi.nlm.nih.gov/gene/). TCGA and GTEx datasets were downloaded from the University of California, Santa Cruz Xena browser platform (http://xenabrowser.net/datapages). RNA expression profiles of FNDC3B were also collected from GEO database (http://www.ncbi.nlm.nih.gov/geo/) using accession numbers GSE16515, GSE28735, GSE62165, GSE62452, GSE15471 and GSE101448. Additional file 2: Table S1 provides comprehensive clinical data on PC patients included in this study based on TCGA dataset.

### FNDC3B expression status in PC and normal pancreatic tissues

The expression of FNDC3B in PC and normal pancreatic tissues was compared using scatter plots and boxplots, along with the application of the Wilcoxon rank sum test and paired or unpaired student t-test. Visualization was achieved using the ggplot2 package (v3.3.6). To assess the diagnostic potential of FNDC3B in PC patients, receiver operating characteristic (ROC) curves were generated through pROC (v1.18.0) and ggplot2 (v3.3.6) package. Based on statistical ranking, FNDC3B expression above or below the median value was categorized as FNDC3B-high or FNDC3B-low groups.

### Identification of differential expression genes (DEGs)

Using DESeq2 (v1.26.0), we leveraged TCGA database to discern DEGs between the FNDC3B-high and FNDC3B-low groups. The identified genes were considered significantly associated if the absolute log (fold change) exceeded 1.5 and the adjusted *P* < 0.05. Subsequently, we employed the ggplot2 package (v3.3.6) to visualize these findings through captivating volcano plots.

### Enrichment of biological function and immune infiltration analysis

The enrichment of FNDC3B-associated DEGs was investigated using the Database for Annotation, Visualization and Integrated Discovery (DAVID) (http://david.ncifcrf.gov). Biological function evaluation of positive DEGs was performed through GO (geneontology.org) and KEGG (www.kegg.jp) enrichment analysis. To ensure significant differences, criteria were set as an enrichment factor > 1.5, a minimum count of 3, and a P < 0.01. GSEA was conducted using the FNDC3B differential expression matrix to identify potential pathways and biological processes associated with FNDC3B-related genes in patients stratified by high or low FNDC3B expression levels. Significance was determined when FDR < 0.25 and adjusted P < 0.05. ClusterProfile package (v4.4.4) was utilized for data visualization while gene sets were obtained from MSigDB database (https://www.gsea-msigdb.org/gsea/msigdb/collections.jsp) [19]. The immune infiltration score of six immune cell types including B cells, CD4 + T cells, CD8 + T cells, neutrophils, macrophages, and dendritic cells across pan-cancer was estimated using the TIMER method from IOBR (v0.99.9) package. Additionally, ssGSEA analysis based on gene expression profiles of 24 immune cell types [20], implemented via GSVA (v1.46.0) package, further explored immune infiltration patterns. Subsequently, the associations between FNDC3B mRNA expression levels and immune infiltration levels were assessed using Wilcoxon rank sum test and Spearman correlation analysis. Furthermore, ESTIMATE (v1.0.13) package was utilized to calculate Stromal Score, Immune Score, and ESTIMATE Score in order to explore the correlation between FNDC3B expression level sand stromal/immune scores. The quantification of Tumor immune dysfunction and exclusion (TIDE) can evaluate the sensitivity of immune checkpoint blockade by simulating tumor immune evasion mechanisms [21]. The TIDE score, along with T-cell dysfunction and exclusion scores for each patient, were determined using the online TIDE platform (http://tide.dfci.harvard.edu/).

### The clinicopathological and prognostic value of FNDC3B, model construction and estimation

Initially, we evaluated the relationship between FNDC3B expression and clinicopathological variables using logistic regression and Wilcoxon rank sum test. Moreover, two-sided log-rank tests were employed to evaluate the prognostic significance of FNDC3B expression in PC patients and various subgroups based on overall survival (OS), disease-specific survival (DSS), and progression-free interval (PFI). To investigate the impact of FNDC3B expression on survival in conjunction with other clinicopathological factors (TNM stage, radiation therapy, primary therapy outcome, gender, race, age, residual tumor status, histologic grade, anatomic neoplasm subdivision), univariate and multivariate Cox analyses were conducted. A *P* < 0.05 was considered statistically significant while the median value of FNDC3B expression served as the cut-off for further analysis. Subsequently, a prognostic nomogram incorporating Cox analysis results along with FNDC3B expression was constructed to predict 1-, 2-, and 3-year survival outcomes using the survival (v3.3.1) package and RMS (v6.3.0) package (https://cran.r-project.org/web/packages/rms/index.html) and calibration plots were generated accordingly. Finally, time-dependent ROC curves were utilized to compare the prognostic efficacy of our novel prognostic model against pure FNDC3B expression using timeROC (v4.0) package in combination with ggplot2 (v3.6) package.

### Cell culture and clinical specimens

The PC cell lines, including AsPC-1, BxPC-3, CFPAC, MIA PaCa-2, PANC-1, T3M4 and human pancreatic ductal epithelial (HPDE) cell line, were acquired from the American Type Culture Collection (ATCC) (Manassas, VA, USA). MIA PaCa-2, PANC-1 and T3M4 were cultured in Dulbecco’s Modified Eagle’s Medium (DMEM) (Hyclone, Logan, UT, USA), BxPC-3 and AsPC-1 were cultured in Roswell Park Memorial Institute (RPMI) 1640 medium (Hyclone, Logan, UT, USA) and CFPAC was cultured in Iscove’s Modified Dulbecco’s Medium (IMDM) (Hyclone, Logan, UT, USA) supplemented with 10% fetal bovine serum (FBS) (Gibco, CA, USA) at 37 ℃ in a 5% CO_2_ cell culture incubator. Furthermore, seven paired samples consisting of tumor tissue and corresponding paratumor normal tissues collected from patients with PC who received surgical resection in the Second Affiliated Hospital of Zhejiang University (SAHZU) were procured for further study.

### Immunohistochemistry (IHC)

Anti-FNDC3B polyclonal primary antibody (1:400 dilution; 22605-1-AP, Proteintech, Chicago, IL, USA) and a two-step staining kit (EnVision™ Detection System, Dako, Copenhagen, Denmark) were used to detect the expression of FNDC3B by IHC. The IHC procedure followed the methodology described in a previous study [22].

### Cell transfection

After being seeded in 6-well plates at a density of 3 × 10^5^ cells/well, the cells were cultured for 24 h. Once reaching 40% confluence, the cells were transfected with FNDC3B small interfering RNA (siRNA) and negative control siRNA (NC siRNA) following the manufacturer's instructions. The FNDC3B siRNAs, including sequences si1: GCATCCACGTGCAAAGGTA; si2: CGGAGAACGTGAACCAAGA; and si3: CCACTATTTTGT-AATGACA, as well as the retained sequence of NC siRNA, were purchased from RiboBio (Guangzhou, China). Subsequent assays were conducted on the respective cells after continuous culture for 24–48 h.

### qRT-PCR

The total RNA was extracted from PC cell lines using TRIzol Reagent (15596026; Ambion, Life Technologies, Carlsbad, CA, USA), followed by first-strand synthesis utilizing a First-Strand Synthesis System for qRT-PCR (A6001, Promega, Madison, USA). The cDNA was then quantified by real-time PCR using a Veriti 96-well Thermal Cycler (4375786; Applied Biosystems, Foster City, CA, USA). Subsequently, PCR was conducted using a StepOnePlus™ system (Applied Biosystems) in accordance with the manufacurer’s instruction. The forward primer sequence of FNDC3B is 5′-GGCGGAATCCCCCATCAAA-3′, while the reverse primer sequence is 5′-ACCTCTCCGTTCAGCAATGG-3′. GAPDH served as the reference gene and its primer sequence was as follows: forward primer 5′-CGGAGTCAACGGATTTGGTCGTAT-3′, reverse primer 5′-AGCCTTCTCCATGGTGGTGAAGAC-3′. Fold changes relative to GAPDH were calculated using the − 2^ΔΔ^CT method.

### Western blot analyses

The detailed procedures of western blot analyses were analogous to a previous study [23]. The primary antibodies employed were as follows: rabbit anti-FNDC3B (1:1000 dilution; 22605-1-AP, Proteintech, Chicago, IL, USA), mouse anti-E-cadherin (1:1000 dilution; ab76055, Abcam, Cambridge, UK), rabbit anti-N-cadherin (1:5000 dilution; ab76011, Abcam, Cambridge, UK), rabbit anti-p21 (1:1000 dilution; ab109520, Abcam, Cambridge, UK), rabbit anti-cyclin B1 (1:50000 dilution; ab32053, Abcam, Cambridge, UK), rabbit anti-CDK1 (1:10000 dilution; ab172730, Abcam, Cambridge, UK) and rabbit anti-GAPDH (1:10000 dilution; ab181602, Abcam, Cambridge, UK).

### Cell proliferation and colony formation assay

Cell Counting Kit-8 (CCK-8) assay was used to evaluate cell proliferation ability. Preprocessed cells were cultured at 3 × 10^3^ cells/well in 96-well plates. And 10 μl/well CCK-8 reagent (Dojindo, Kumamoto, Japan) was added at 0, 24, 48, 72 and 96 h after cell adherence. Then the optical densities were measured at 450 nm (OD450) using microplate reader (Wellscan MK3; Thermo Labsystems, Helsinki, Finland). The values of OD630 were also measured as a reference. In the colony formation assay, 500 preprocessed cells were added to each well of 6-well plates and cultured in cell incubator for 14 days. Subsequently, the cells were first fixed with 4% paraformaldehyde for 20 min and then stained using crystal violet for 10 min before washed by phosphate buffered saline for three times. The colony were photographed and measured by Image J software (NIH, Bethesda, MD, USA).

### Wound-healing and transwell migration and invasion assay

In wound-healing assay, the cells were cultured in 6-well plates at 3 × 10^5^ cells/well in FBS-free medium. When cells reached 70–80% confluence, sterile pipette tips were used to scratch and form a “wound”. In transwell migration and invasion assay, we used non-coated and coated membranes in transwell chambers (24-well insert; 8-μm pore size; Corning Life Sciences, Corning, NY, USA) respectively. Preprocessed cells (3 × 10^4^) were added in the upper chamber with FBS-free medium and medium containing 10% FBS was added to the lower chamber. After cultured in cell incubator for 48 h, a cotton swab was used to wipe the unpenetrated cells in the upper chamber, and the penetrated cells were fixed in methyl alcohol for 20 min and then subjected to hematoxylin and eosin staining 10 min and 5 min respectively for counting. The “wound” and the stained cells were observed and photographed via a DFC300FX microscope (Leica, Jena, Germany). The width of “wound” and cell number were further obtained using Image J software (NIH, Bethesda, MD, USA).

### Statistical analysis

All the statistical analyses were conducted by RStudio software (v2022.12.0 + 353) (https://posit.co/download/rstudio-desktop/) and R software (v4.2.1). One-way analysis of variance (ANOVA) and two tailed Student t test were employed to analyze the data while other statistical methods were shown beforehand. All *P* < 0.05 were deemed as significantly difference.

## Results

### FNDC3B expression levels in pan-cancer and PC

**Fig. 1:**
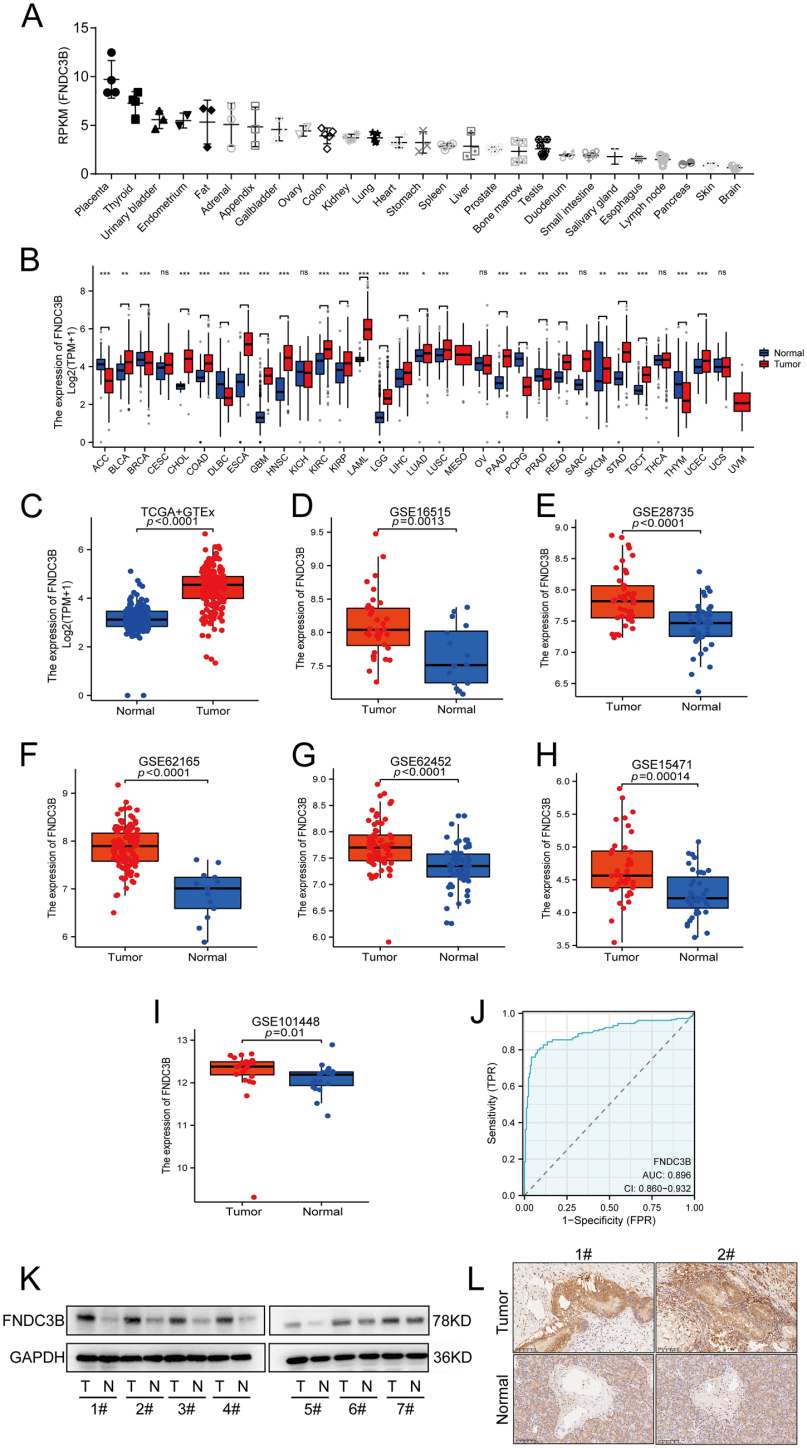
FNDC3B expression levels in normal tissues, pan-cancerous and PC tissues. **A** FNDC3B expression levels in various normal human tissues (data from NCBI, BioProject: PRJEB4337). **B** FNDC3B expression levels in tumor and normal samples based on TCGA and GTEx databases. **C** FNDC3B expression levels in PC and normal pancreatic tissues based on TCGA and GTEx databases. **D**–**I** FNDC3B expression levels between tumor and normal samples in GSE16515, GSE28735, GSE62165, GSE62452, GSE15471 and GSE101448 datasets. **J** Receiver operating characteristic curve was recruited to evaluate the diagnostic value of FNDC3B expression in PC. **K** Expression of FNDC3B in paired tumor tissues and adjacent nontumor tissues from seven PC patients. **L** Representative images of CXCL5 in tumor tissues and the adjacent nontumor tissues. *P < 0.05; **P < 0.01 and ***P < 0.001. *AUC* area under the curve, *FNDC3B* fibronectin type III domain containing 3B, *GTEx* genotype-tissues expression, ns not significant, *PC* pancreatic cancer, *TCGA* the cancer genome atlas, *RPKM* reads per kilobase per million, *TPM* transcripts per million, *TPR* true positive rate, *FPR* false positive rate

Firstly, we evaluated the expression of FNDC3B in normal tissues and in pan-cancer using data extracted from NCBI, TCGA and GTEx database, respectively. The data from NCBI revealed that FNDC3B expression in normal pancreatic tissues was significantly low among all the 27 normal tissues or organs (Fig. 1A). Moreover, among the 33 types of malignancies analyzed, FNDC3B expression was upregulated in 19 types of malignancies while downregulated in 6 types of malignancies, indicating a widespread overexpression of FNDC3B in tumors. In addition to PC, Wilcoxon rank sum test demonstrated higher expression levels of FNDC3B in bladder urothelial carcinoma, cholangiocarcinoma, colon adenocarcinoma, esophageal carcinoma, glioblastoma multiforme, kidney renal clear cell carcinoma, kidney renal papillary cell carcinoma, acute myeloid leukemia, brain lower grade glioma hepatocellular carcinoma liver, lung adenocarcinoma squamous cell carcinoma lung rectum adenocarcinoma skin cutaneous melanoma stomach adenocarcinoma testicular germ cell tumor uterine corpus endometrial carcinoma (*P* < 0.05, Fig. 1B). Subsequently, we further compared FNDC3B expression via TCGA & GTEx database and six GEO datasets (GSE16515, GSE28735, GSE62165, GSE62452, GSE15471 and GSE101448), which consistently showed higher levels of FNDC3B expression in PC tissues than those observed in normal pancreatic tissues (*P* < 0.05, Fig. 1C–I). Additionally, diagnostic-related ROC curve analysis indicated a high diagnostic efficiency for FNDC3B with an area under the curve value of 0.896 (Fig. 1J). To validate our bioinformatic analysis results, tissue samples of seven patients with PC treated in SAHZU were recruited for western blot analysis and IHC. The results both confirmed that the expression of FNDC3B was significantly higher than that observed in tumor-adjacent tissue (Fig. 1K and L).

### Biological functions and mechanisms of FNDC3B-related genes in PC

**Fig. 2:**
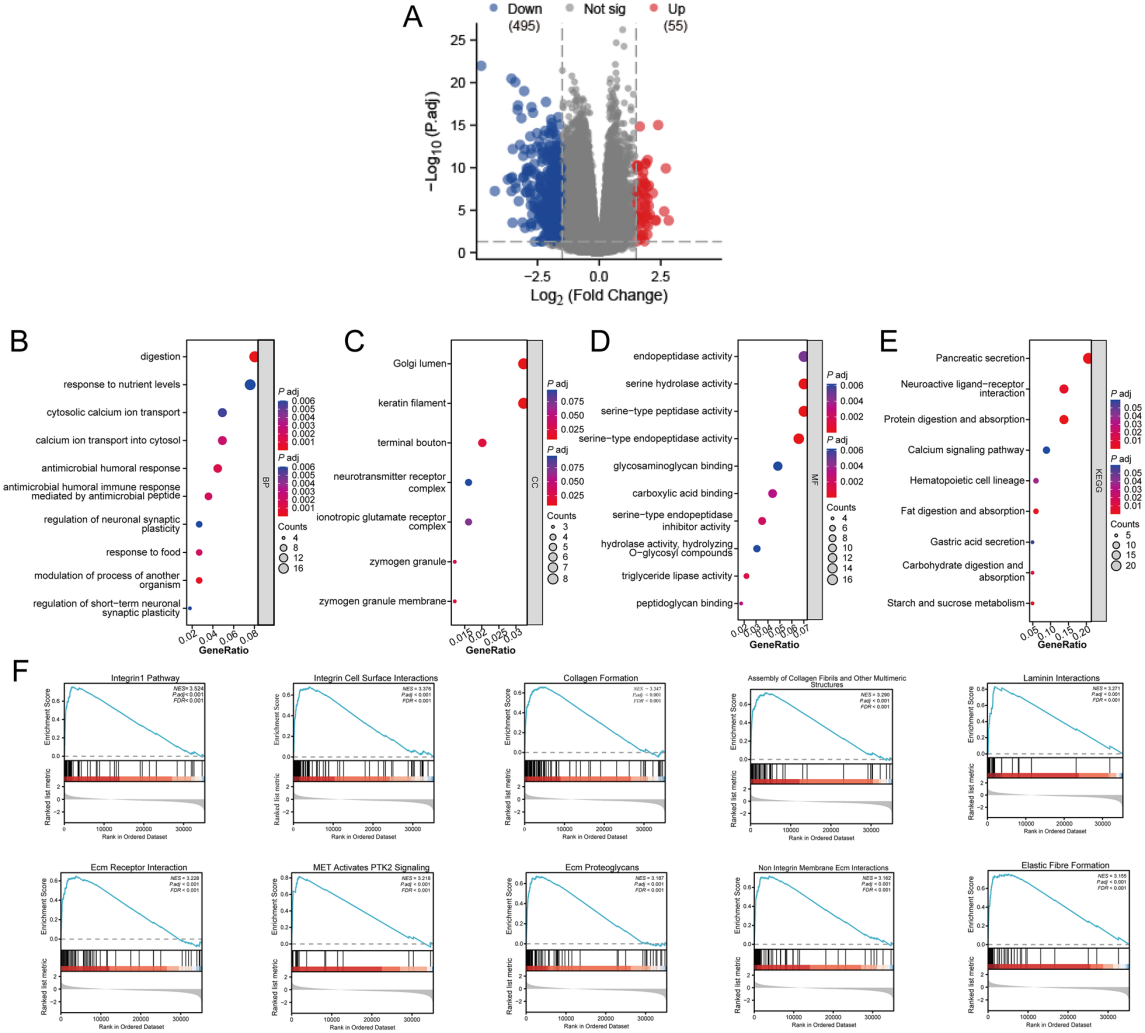
GO, KEGG enrichment analysis and GSEA of DEGs. **A** Differentially expressed genes were presented using volcano plot. GO enrichment analysis of DEGs for **B** biological processes, **C** cellular components and **D** molecular functions. **E** KEGG pathway enrichment analysis of DEGs. **F** GSEA of DEGs. *DEGs* differentially expressed genes, *ECM* extracellular matrix, *GO* Gene Ontology, *GSEA* Gene Set Enrichment Analysis, *KEGG* Kyoto Encyclopedia of Genes and Genomes, *MET* metformin, *P. adj* adjusted P-value, *PTK* protein tyrosine kinase

To identify DEGs associated with FNDC3B, we compared gene expressions between 89 samples exhibiting high levels of FNDC3B and 90 samples with low levels. Employing the aforementioned filtering criteria yielded in a total of 550 significantly DEGs, comprising 55 upregulated genes and 495 downregulated genes. The results were visually represented through volcano plot (Fig. 2A; Additional file 3: Table S2). The statistically significant FNDC3B-related genes mentioned above were further utilized to conduct GO and KEGG enrichment analysis in order to explore the potential biological function of FNDC3B in PC. GO analysis unveiled that FNDC3B could participate in diverse biological processes, encompassing “Digestion,” “Regulation of neuronal synaptic plasticity,” “Keratin filament,” “Neurotransmitter receptor complex,” as well as the activity of several digestive enzymes. Additionally, the outcomes from KEGG analysis indicated that FNDC3B might intricately partake in “Pancreatic secretion” and the digestion of essential nutrients (Fig. 2B–E; Additional file 4: Table S3). Subsequently, GSEA was performed to identify potential processes and signaling pathways associated with FNDC3B expression in PC. The findings remarkably demonstrated a strong correlation between FNDC3B and processes/signaling pathways involved in cell–cell interaction modulation and cell mobility, such as “Collagen formation,” “Laminin interactions,” “ECM receptor interaction,” “Integrin 1 pathway,” and “PTK2 signaling pathway activation” (Fig. 2F; Additional file 5: Table S4).

### Correlation between FNDC3B expression and immune infiltration

**Fig. 3:**
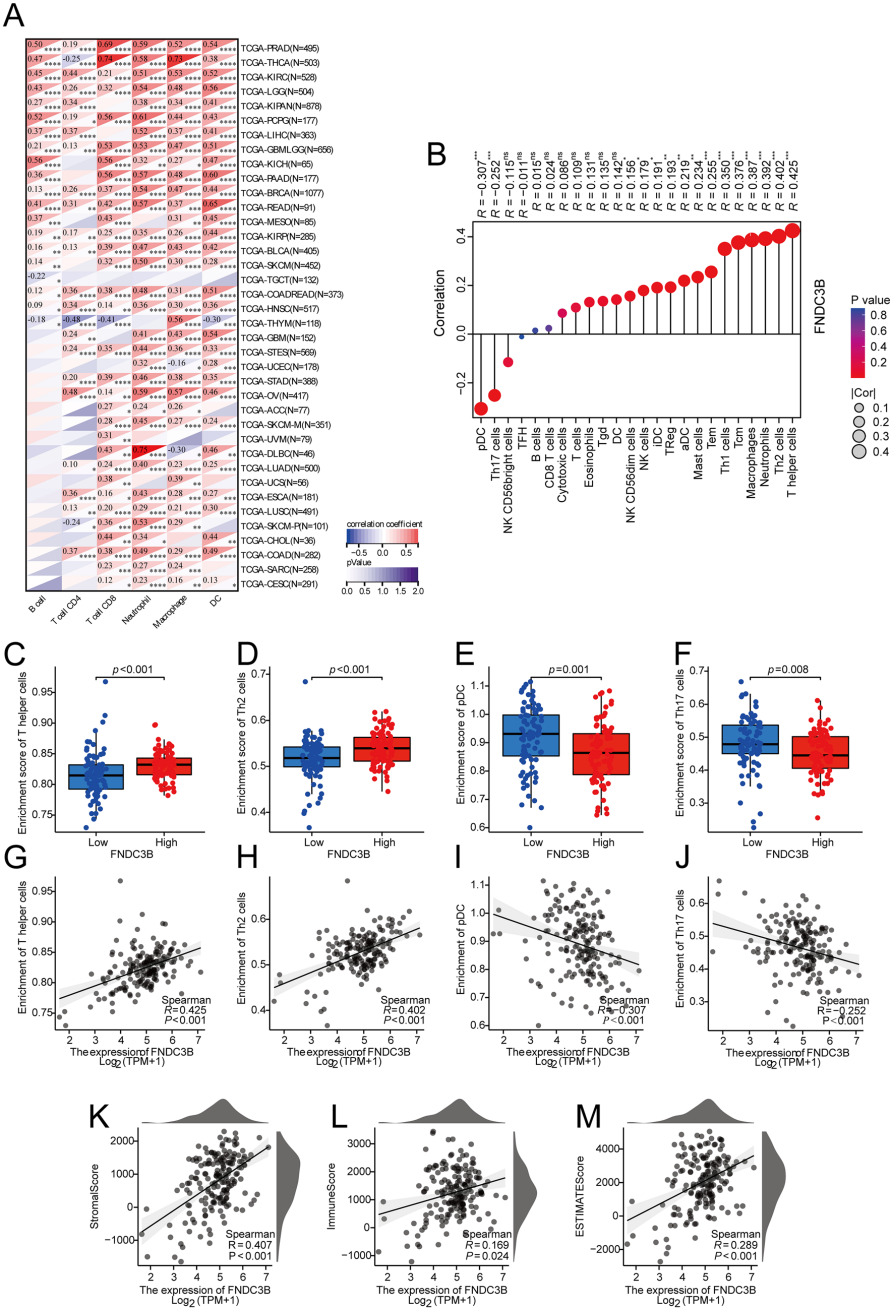
Association between immune cells infiltration and FNDC3B expression in the tumor microenvironment. **A** The association between FNDC3B expression and immune cells abundance in pan-cancer via TIMER online tool. **B** The correlation between FNDC3B expression and immune cells abundance via ssGSEA. (**C**–**F**) Comparison of the abundances of **C** T helper cells, **D** Th2 cells, **E** pDCs and **F** Th17 cells between FNDC3B-low and -high groups. **G**–**J** Scatter plots presented the association between the abundances in **G** T helper cells, **H** Th2 cells, **I** pDCs and **J** Th17 cells and FNDC3B expression levels. **K**–**M** The ESTIMATE related scores built based on TCGA database. *P < 0.05; **P < 0.01; ***P < 0.001 and ****P < 0.0001. *DC* dendritic cells, *iDC* immature dendritic cells, *FNDC3B* fibronectin type III domain containing 3B, *NK* natural killer, *pDC* plasmacytoid dendritic cells, *Tcm* central memory T cells, *TFH* T follicular helper cells, *TGD* γδT cells, *Th* T helper, *TPM* transcripts per million, *Treg* regulatory T cells

The immune response and cytokine secretion play a crucial role in both the immune escape of tumor cells and the potential for immunotherapy [24]. Therefore, we further investigated the correlation between FNDC3B expression and immune infiltration to assess its potential as an indicator for immunotherapy. Initially, we analyzed the correlation between FNDC3B expression and immune infiltration in pan-cancer. The results indicated that FNDC3B served as a reliable biomarker associated with immune infiltration in most cancers, exhibiting a positive correlation with intratumoral infiltration of B cells, CD8 + T cells, neutrophils, macrophages, and DCs (Fig. 3A). Subsequently, we comprehensively explored immune infiltration correlation using ssGSEA and Spearman correlation analysis. As depicted in Fig. 3B, there was a positive relationship between FNDC3B expression and several types of infiltrating immune cells, notably T helper cells (R = 0.425, *P* < 0.001) (Fig. 3B, C and G), Th2 cells (R = 0.402, *P* < 0.001) (Fig. 3B, D and H), and neutrophils (R = 0.392, *P* < 0.001) (Fig. 3B) ranked first, second and third respectively. However, the only significantly negative correlations were observed in the infiltration of plasmacytoid dendritic cells (pDCs) (R = − 0.307, *P* < 0.001) (Fig. 3B, E and I) and Th17 cells (R = − 0.252, *P* < 0.001) (Fig. 3B, F and J). Moreover, the StromalScore (R = 0.407, *P* < 0.001) (Fig. 3K), the ImmuneScore (R = 0.169, *P* = 0.024) (Fig. 3L) and the ESTIMATEScore (R = 0.289, P < 0.001) (Fig. 3M) were calculated to confirm FNDC3B expression was significantly related to immune infiltration. The TIDE algorithm revealed that among the FNDC3B high expression group, 33 patients exhibited a positive response to immunotherapy, while 55 patients did not respond. Additionally, it was observed that both the TIDE score and T cell exclusion score were significantly elevated in the high FNDC3B expression group, indicating a poor immunotherapy outcome. This suggested that high FNDC3B might serve as an unfavorable prognostic indicator for immunotherapy efficacy (Additional file 1: Fig.S1).

### The correlation between FNDC3B expression and clinicopathological variates in PC

**Fig. 4:**
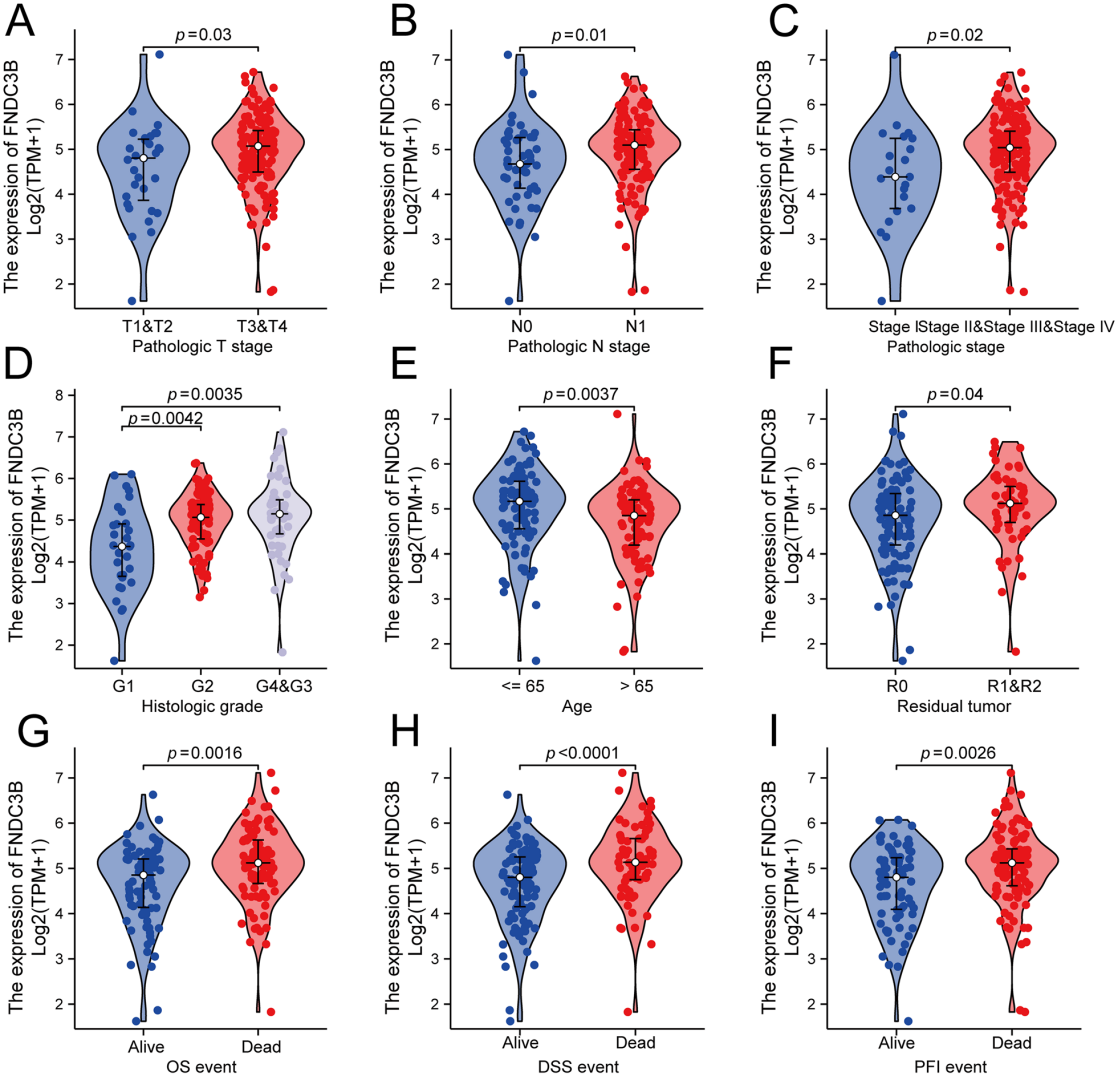
The correlation between FNDC3B expression and clinicopathological characteristics, including **A** pathologic T stage, **B** pathologic N stage, **C** pathologic stage, **D** histologic grade, **E** age, **F** residual tumor, **G** OS event, **H** DSS event and **I** PFI event in PC patients in TCGA cohort. *DSS* disease-specific survival, *FNDC3B* fibronectin type III domain containing 3B, *OS* overall survival, *PC* pancreatic cancer, *PFI* progression-free interval, *TCGA* the cancer genome atlas

To determine the clinical significance of FNDC3B in PC, we evaluated the association between FNDC3B expression and clinicopathological variates in 178 PC patients in TCGA (Table 1). High FNDC3B expression in PC was significantly related to advanced pathological T stage (T3 & T4 *vs.* T1 & T2, P = 0.03), positive lymph node involvement (N1 *vs.* N0, P = 0.01), advanced pathological stage (stage II, stage III &stage IV *vs.* stage I, P = 0.02), higher histologic grade (G2 *vs.* G1, P = 0.0042; G3 & G4 *vs.* G1, P = 0.0035), younger age (< = 65 *vs.* > 65, P = 0.0037), residual tumor (R1 & R2 *vs.* R0, P = 0.04), OS event (Dead *vs.* Alive, P = 0.0016), DSS event (Dead *vs.* Alive, P < 0.0001), PFI event (Dead *vs.* Alive, P = 0.0026) (Fig. 4A–I). Logistic regression analysis revealed that high FNDC3B expression was specifically associated with a poor lymph node status (N1*vs.*N0, 0.004), an advanced pathological stage (Stage II & Stage III & Stage IV *vs.* Stage I, 0.040), more prevalent among younger patients (> 65 *vs.* < 65, P = 0.007) and was correlated with positive residual tumor margin (R1 & R2 *vs.* R0, P = 0.031) while marginal correlated with a higher histological grade (Table 2). These findings suggested that elevated levels of FNDC3B in PC may contribute to high risk of PC progression.

**Table 1:** The correlation between clinicopathological variables and FNDC3B expression in PC patients

Characteristics	Levels	Low expression of FNDC3B	High expression of FNDC3B	*P*
n	89	89		
T stage, n (%)	T1	5 (2.8%)	2 (1.1%)	0.114
	T2	14 (8%)	10 (5.7%)	
	T3	65 (36.9%)	77 (43.8%)	
	T4	3 (1.7%)	0 (0%)	
N stage, n (%)	N0	33 (19.1%)	16 (9.2%)	**0.004**
	N1	53 (30.6%)	71 (41%)	
M stage, n (%)	M0	31 (36.9%)	49 (58.3%)	0.980
	M1	1 (1.2%)	3 (3.6%)	
Pathologic stage, n (%)	I	15 (8.6%)	6 (3.4%)	**0.034**
	II	68 (38.9%)	79 (45.1%)	
	III	3 (1.7%)	0 (0%)	
	IV	1 (0.6%)	3 (1.7%)	
Radiation therapy, n (%)	No	58 (35.6%)	60 (36.8%)	0.633
	Yes	24 (14.7%)	21 (12.9%)	
Primary therapy outcome, n (%)	PD	17 (12.1%)	33 (23.6%)	0.079
	SD	6 (4.3%)	3 (2.1%)	
	PR	4 (2.9%)	6 (4.3%)	
	CR	39 (27.9%)	32 (22.9%)	
Age, n (%)	≤ 65	38 (21.3%)	56 (31.5%)	**0.007**
	> 65	51 (28.7%)	33 (18.5%)	
Race, n (%)	Asian	6 (3.4%)	5 (2.9%)	0.972
	Black or African American	3 (1.7%)	3 (1.7%)	
	White	80 (46%)	77 (44.3%)	
Gender, n (%)	Female	42 (23.6%)	38 (21.3%)	0.547
	Male	47 (26.4%)	51 (28.7%)	
Histologic grade, n (%)	G1	24 (13.6%)	7 (4%)	0.001
	G2	44 (25%)	51 (29%)	
	G3	17 (9.7%)	31 (17.6%)	
	G4	2 (1.1%)	0 (0%)	
Residual tumor, n (%)	R0	59 (36%)	47 (28.7%)	0.056
	R1	19 (11.6%)	34 (20.7%)	
	R2	3 (1.8%)	2 (1.2%)	
Anatomic neoplasm subdivision, n (%)	Head	67 (37.6%)	72 (40.4%)	0.365
	Other	22 (12.4%)	17 (9.6%)	
Alcohol history, n (%)	No	29 (17.5%)	35 (21.1%)	0.280
	Yes	55 (33.1%)	47 (28.3%)	
Smoker, n (%)	No	40 (27.6%)	26 (17.9%)	0.133
	Yes	38 (26.2%)	41 (28.3%)	
History of chronic pancreatitis, n (%)	No	66 (46.5%)	63 (44.4%)	0.854
	Yes	7 (4.9%)	6 (4.2%)	
History of diabetes, n (%)	No	56 (38.1%)	53 (36.1%)	0.680
	Yes	21 (14.3%)	17 (11.6%)	

Total patients’ number does not equal to 89 in all variates due to lack of patient’s information for some cases

*CR* complete response, *FNDC3B* fibronectin type III domain containing 3B, *G1*: well-differentiated, *G2*: moderately-differentiated, *G3*: poorly-differentiated, *G4*: undifferentiated, *M*: metastasis, *N*: lymph node, *PC* pancreatic cancer, *PR* partial response, *PD* progressive disease, *SD* stable disease, T: tumor

Bold values indicate that P < 0.05 which are statistically significant

**Table 2:** FNDC3B expression associated with clinicopathological characteristics (logistic regression)

Characteristics	Total (N)	Odds ratio (OR)	P value
T stage (T3 & T4 vs. T1 & T2)	176	1.793 (0.811–3.962)	0.149
N stage (N1 vs. N0)	173	2.763 (1.379–5.536)	**0.004**
M stage (M1 vs. M0)	84	1.898 (0.189–19.072)	0.586
Pathologic stage (Stage II & Stage III & Stage IV vs. Stage I)	175	2.847 (1.049–7.726)	**0.040**
Radiation therapy (Yes vs. No)	163	0.846 (0.425–1.683)	0.633
Primary therapy outcome (PR & CR vs. PD & SD)	140	0.565 (0.286–1.116)	0.100
Age (> 65 vs. ≤ 65)	178	0.439 (0.241–0.801)	**0.007**
Race (White vs. Asian & Black or African American)	174	1.083 (0.397–2.951)	0.876
Gender (Male vs. Female)	178	1.199 (0.664–2.172)	0.547
Residual tumor (R1 & R2 vs. R0)	164	2.054 (1.068–3.952)	**0.031**
Histologic grade (G3 & G4 vs. G1 & G2)	176	1.913 (0.979–3.738)	0.058
Anatomic neoplasm subdivision (Other vs. Head of Pancreas)	178	0.719 (0.352–1.470)	0.366
Alcohol history (Yes vs. No)	166	0.708 (0.378–1.326)	0.281
Smoker (Yes vs. No)	145	1.660 (0.856–3.219)	0.134
History of chronic pancreatitis (Yes vs. No)	142	0.898 (0.286–2.818)	0.854
History of diabetes (Yes vs. No)	147	0.855 (0.407–1.796)	0.680

Total patients’ number is inconsistent in all variates due to lack of patient’s information for some cases

*CR* complete response, *FNDC3B* fibronectin type III domain containing 3B, *G1*: well-differentiated, *G2*: moderately-differentiated, *G*3: poorly-differentiated, G4: undifferentiated, *M*: metastasis, *N*: lymph node, *PC* pancreatic cancer, *PR* partial response, *PD* progressive disease, *SD* stable disease, *T*: tumor

Bold values indicate that P < 0.05 which are statistically significant

### The prognostic value of FNDC3B expression in PC patients

**Fig. 5:**
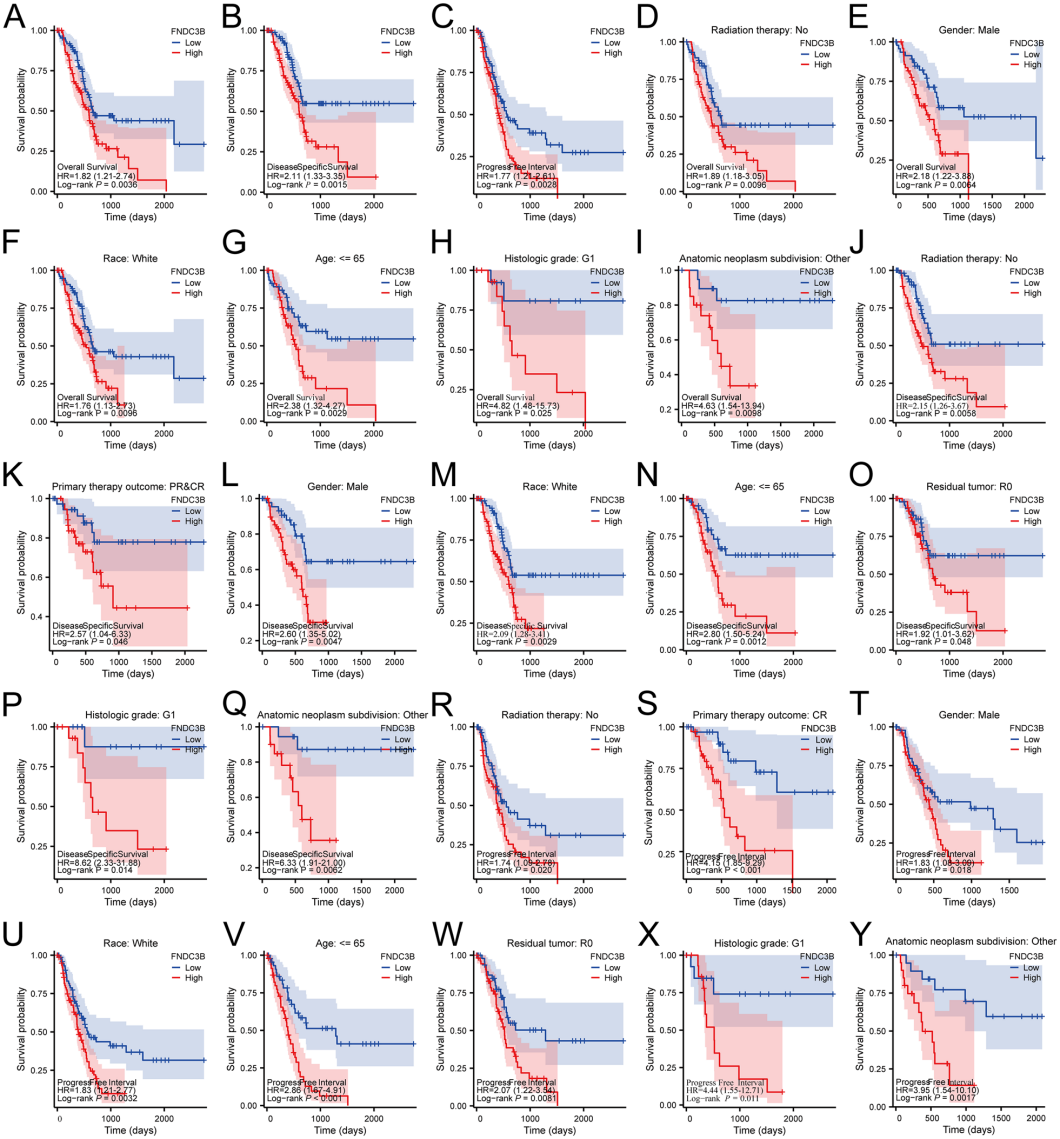
Kaplan–Meier survival plots comparing with high and low FNDC3B expression in PC patients. **A**–**C** Survival curve of OS, DSS and PFI between high and low FNDC3B expression in PC patients. **D**–**I** OS survival curve of Radiation therapy: No, Gender: Male, Race: White, Age: ≤ 65, Histologic grade: G1, Anatomic neoplasm subdivision: Other. **J**–**Q** DSS survival curve of Radiation therapy: No, Primary therapy outcome: PR & CR, Gender: Male, Race: White, Age: ≤ 65, Residual tumor: R0, Histologic grade: G1, Anatomic neoplasm subdivision: Other. (**R**–**Y**) PFI survival curve of Radiation therapy: No, Primary therapy outcome: PR, Gender: Male, Race: White, Age: ≤ 65, Residual tumor: R0, Histologic grade: G1, Anatomic neoplasm subdivision: Other. *DSS* disease-specific survival, *FNDC3B* fibronectin type III domain containing 3B, *OS* overall survival, *PC* pancreatic cancer, *PFI* progression-free interval

**Fig. 6:**
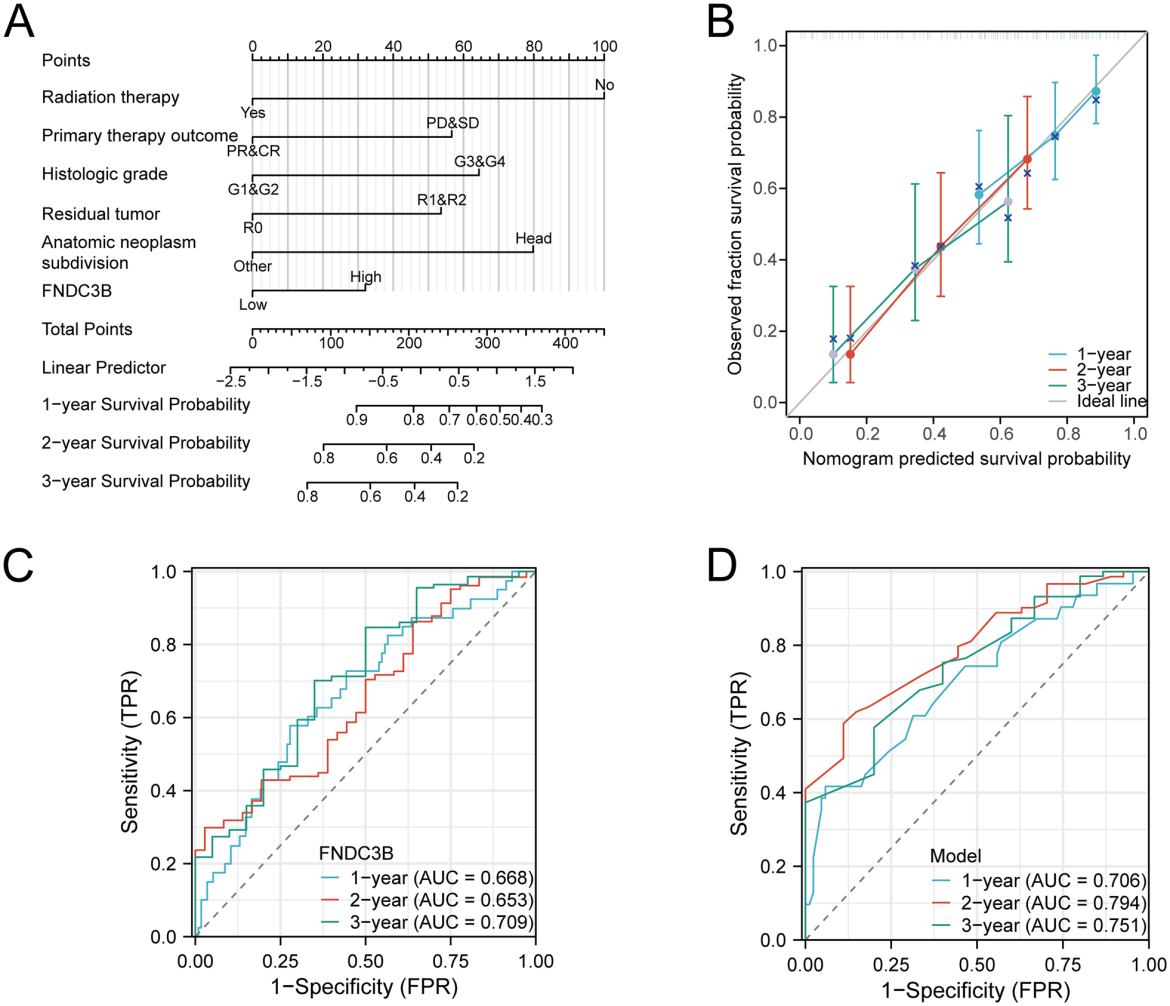
Prognostic model to predict the 1-, 2- and 3-year OS in PC. **A** Nomogram to predict the probability of 1‑, 2‑ and 3‑year OS for patients with PC. **B** Calibration plots of the nomogram for the estimation of the probability of OS at 1, 2 and 3 years. Receiver operating characteristic curve of FNDC3B expression (**C**) and the model (**D**) for the prediction of 1‑, 2‑ and 3‑year OS for patients with PC. *AUC* area under the curve, *FNDC3B* fibronectin type III domain containing 3B, *OS* overall survival, *PC* pancreatic cancer, *TPR* true positive rate, *FPR* false positive rate

To validate the prognostic significance of FNDC3B expression in PC, we initially performed log-rank tests on both the entire cohort and specific subgroups of PC patients. The results revealed that high FNDC3B expression was associated with significantly shorter OS, DSS and PFI compared to low FNDC3B expression in the entire cohort (Fig. 5A–C, P = 0.0036, *P* = 0.0015 & *P* = 0.0028). Then subgroup analyses demonstrated that within the subgroups of PC patients without radiation therapy, male gender, white race, age ≤ 65 years old, histologic grade G1, or other anatomic neoplasm subdivisions, those with high FNDC3B expression had poorer OS outcomes (Fig. 5D–I, P < 0.05). Similarly, high FNDC3B expression was associated with lower DSS among PC patients without radiation therapy or primary therapy outcome PR&CR who were male individuals aged ≤ 65 years old with white race and R0 residual tumor status and histologic grade G1 or other anatomic neoplasm subdivisions (Fig. 5J, Q, P < 0.05). Furthermore, elevated FNDC3B expression correlated with reduced PFI in PC patients without radiation therapy but having achieved CR for primary therapy outcome while being male individuals aged ≤ 65 years old with white race and R0 residual tumor status along with histologic grade G1 or other anatomic neoplasm subdivisions (Fig. 5R–Y, P < 0.05). The prognostic value of FNDC3B in different subgroups of PC patients, as assessed by OS, DSS and PFI was presented in Table 3, Additional files 6, 7: Tables S5 and S6 respectively. Subsequently, univariate Cox analysis revealed that high expression of FNDC3B in PC patients was associated with poor OS (HR = 1.880, 95%CI 1.235–2.862; *P* = 0.003), DSS (HR = 2.168, 95%CI 1.344–3.498; *P* = 0.002) and PFI (HR = 1.822, 95%CI 1.223–2.710; *P* = 0.003). We further performed multivariate Cox analysis using the significantly different clinicopathological variables but found no significant association between FNDC3B expression and OS (HR = 2.223, 95%CI 0.910–5.430; P = 0.079), DSS (HR = 2.209, 95%CI 0.821–5.946; *P* = 0.117) and PFI (HR = 1.545, 95%CI 0.686–3.476; *P* = 0.293) (Table 4, Additional files 8, 9: Tables S7 and S8). To enhance the prognostic efficacy of FNDC3B in PC, we developed a nomogram incorporating radiation therapy, primary therapy outcome, histologic grade, residual tumor status, anatomic neoplasm subdivision along with FNDC3B expression to accurately predict the 1-, 2-, and 3-year OS for PC patients (Fig. 6A). The accuracy of this nomogram was evaluated through calibration curves which demonstrated close alignment between the predicted 1-, 2- and 3-year OS lines with the ideal line (Fig. 6B). Moreover, our innovative nomogram model exhibited superior prognostic efficacy compared to solely relying on FNDC3B as indicated by the ROC curve analysis (Fig. 6C and D).

**Table 3:** The prognostic value of FNDC3B (Overall Survival) in subgroups of patients with pancreatic cancer

Characteristics	Total (N)	HR (95% CI)	P value
T stage			
T1 & T2	31	1.416 (0.433–4.635)	0.5435
T3 & T4	145	1.468 (0.951–2.265)	0.0803
N stage			
N0	50	1.495 (0.593–3.768)	0.3873
N1	123	1.078 (0.681–1.706)	0.7470
M stage			
M0	79	1.566 (0.860–2.854)	0.1470
M1	5	–	–
Radiation therapy			
No	118	1.893 (1.176–3.046)	**0.0096**
Yes	45	1.249 (0.476–3.276)	0.6381
Primary therapy outcome			
PD & SD	58	1.200 (0.673–2.138)	0.5306
PR & CR	81	1.835 (0.906–3.717)	0.0912
Gender			
Female	80	1.239 (0.695–2.209)	0.4661
Male	98	2.178 (1.222–3.883)	**0.0064**
Race			
White	157	1.760 (1.133–2.733)	**0.0096**
Asian & Black or African American	17	0.894 (0.217–3.677)	0.8643
Age			
≤ 65	93	2.376 (1.322–4.269)	**0.0029**
> 65	85	1.186 (0.670–2.101)	0.5524
Residual tumor			
R0	107	1.494 (0.863–2.588)	0.1528
R1 & R2	57	1.398 (0.711–2.748)	0.3227
Histologic grade			
G1 & G2	126	1.925 (1.162–3.189)	**0.0099**
G3 & G4	50	1.193 (0.583–2.443)	0.6201
Anatomic neoplasm subdivision			
Head of pancreas	138	1.284 (0.828–1.991)	0.2604
Other	40	4.631 (1.539–13.940)	**0.0098**

Total patients’ number does not equal to 178 in all variates due to lack of patient’s information for some cases

*CR* complete response, *FNDC3B* fibronectin type III domain containing 3B, *G1*: well-differentiated, *G2*: moderately-differentiated, *G3*: poorly-differentiated, *G4*: undifferentiated, *M*: metastasis, *N*: lymph node, *PC* pancreatic cancer, *PR* partial response, *PD* progressive disease, *SD* stable disease, *T*: tumor

Bold values indicate that P < 0.05 which are statistically significant

**Table 4:** Univariate and multivariate Cox analysis (Overall Survival) of prognostic covariates in patients with PC

Characteristics	Total (N)	Univariate analysis		Multivariate analysis	
		Hazard ratio (95% CI)	*P* value	Hazard ratio (95% CI)	*P* value
T stage (T3 & T4 vs. T1 & T2)	176	2.053 (1.089–3.871)	**0.026**	2.643 (0.296–23.597)	0.384
N stage (N1 vs. N0)	173	2.114 (1.259–3.549)	**0.005**	1.768 (0.428–7.302)	0.431
M stage (M1 vs. M0)	84	1.050 (0.251–4.388)	0.947	1.133 (0.123–10.469)	0.912
Pathologic stage (Stage II & Stage III & Stage IV vs. Stage I)	175	2.326 (1.067–5.069)	**0.034**	0.202 (0.011–3.789)	0.285
Radiation therapy (Yes vs. No)	163	0.501 (0.294–0.854)	**0.011**	0.210 (0.069–0.639)	**0.006**
Primary therapy outcome (CR & PR vs. PD & SD)	140	0.425 (0.267–0.675)	** < 0.001**	0.458 (0.201–1.044)	0.063
Age (> 65 vs. ≤ 65)	178	1.306 (0.867–1.968)	0.202	1.382 (0.625–3.054)	0.424
Race (White vs. Asian & Black or African American)	174	1.176 (0.589–2.345)	0.646	6.404 (0.769–53.321)	0.086
Gender (Male vs. Female)	178	1.214 (0.808–1.825)	0.350	0.872 (0.386–1.967)	0.741
Histologic grade (G3 & G4 vs. G1 & G2)	176	1.517 (0.983–2.340)	0.060	2.220 (1.016–4.852)	**0.046**
Residual tumor (R1 & R2 vs. R0)	164	1.630 (1.051–2.528)	**0.029**	1.107 (0.504–2.436)	0.800
Anatomic neoplasm subdivision (Other vs. Head of Pancreas)	178	0.427 (0.236–0.770)	**0.005**	1.352 (0.367–4.978)	0.650
FNDC3B (High vs. Low)	178	1.880 (1.235–2.862)	**0.003**	2.223 (0.910–5.430)	0.079

Total patients’ number does not equal to 178 in all variates due to lack of patient’s information for some cases

*CR* complete response, *FNDC3B* fibronectin type III domain containing 3B, *G1*: well-differentiated, *G2*: moderately-differentiated, *G3*: poorly-differentiated, *G4*: undifferentiated, *M*: metastasis, *N*: lymph node, *PC* pancreatic cancer, *PR* partial response, *PD* progressive disease, *SD* stable disease, *T*: tumor

Bold values indicate that P < 0.05 which are statistically significant

### Knockdown FNDC3B expression hampers PC cells proliferation, migration and invasion

**Fig. 7:**
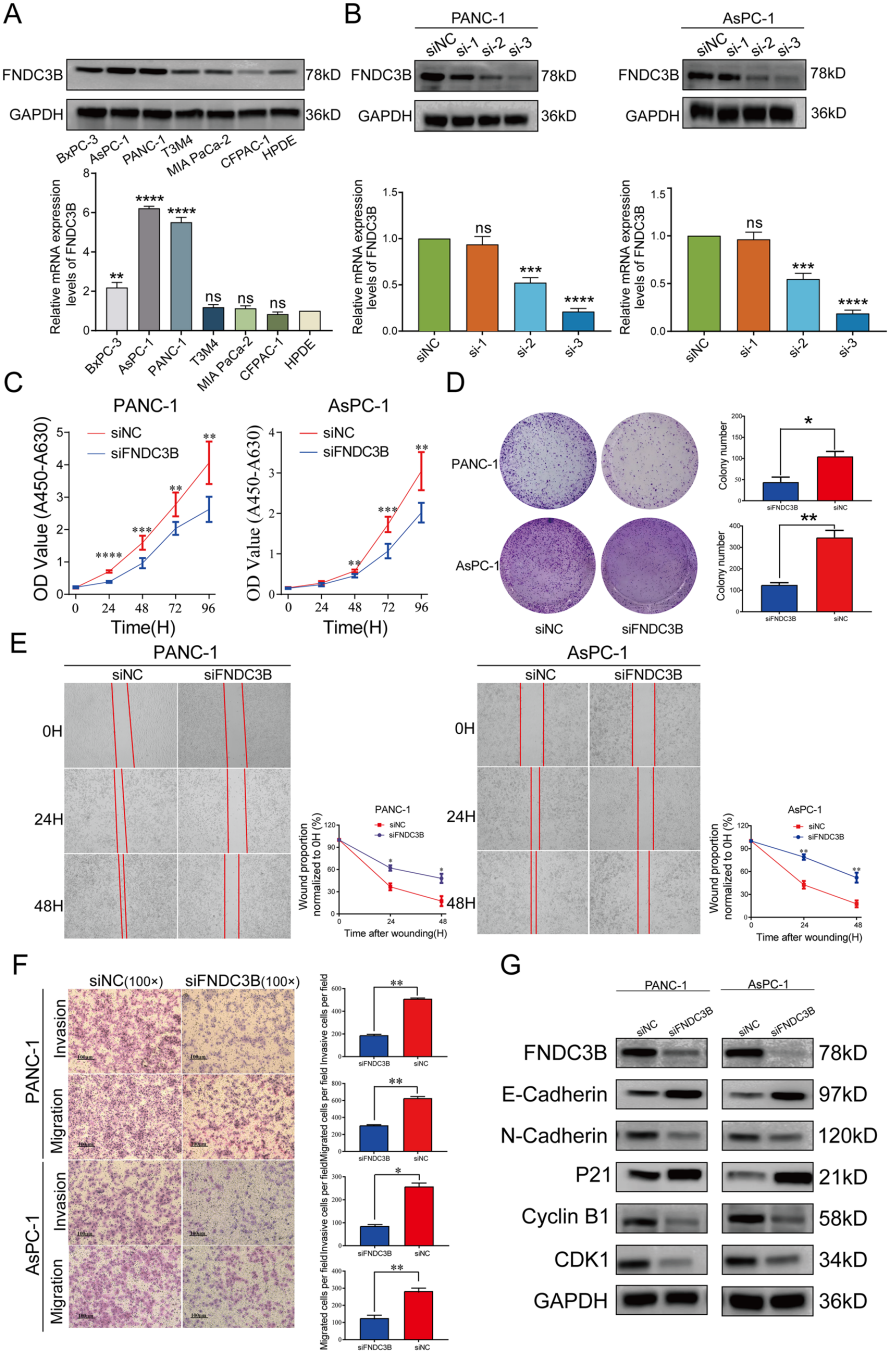
Knockdown FNDC3B expression hampers proliferation and metastasis ability of PC cells. **A** FNDC3B expression in six PC cell lines and one normal pancreatic epithelial cell line via western blot analyses. **B** The efficacies of FNDC3B siRNAs were confirmed via western blot analyses. **C** CCK-8 and **D** colony formation assays demonstrated that cell proliferation ability impaired when FNDC3B expression knockdown. **E** Wound healing and **F** transwell migration and invasion assays showed that cell metastasis ability attenuated when FNDC3B expression knockdown. **G** Protein levels of EMT and cell cycle related regulators were evaluated by western blot analyses. **H** Knockdown of FNDC3B decreased the tumor growth in orthotopic xenograft models established using PANC-1 cells. Representative IVIS images of nude mice are shown. *P < 0.05; **P < 0.01; ***P < 0.001 and ****P < 0.0001. *FNDC3B* fibronectin type III domain containing 3B, *ns* not significant, *PC* pancreatic cancer

In order to further investigate the role of FNDC3B expression in PC progression, we firstly confirmed FNDC3B expression in different PC cell lines through western blot analyses. Figure 7A illustrated that AsPC-1 exhibited the highest level of FNDC3B among the included cell lines while CFPAC-1 showed minimal expression. Additionally, HPDE displayed lower levels of FNDC3B compared to most PC cell lines. Subsequently, we selected AsPC-1 and PANC-1 as tool cell lines due to their high FNDC3B expression levels for verifying knockdown efficacies using siRNAs. The results indicated that siRNA-2 and siRNA-3 significantly decreased FNDC3B expression, with siRNA-3 demonstrating the strongest knockdown effect which was chosen for subsequent assays (Fig. 7B). Then, CCK-8 and colony formation assays were recruited to assess the impact of altered FNDC3B expression on proliferation ability in PC cells. These findings demonstrated a significant weakening in proliferation ability 24 h after transfection and a decrease in colony numbers following downregulation of FNDC3B expression (Fig. 7C and D). Meanwhile, using wound-healing and transwell migration assays, we observed that knockdown of FNDC3B impaired the healing of "wounds" and hindered the migration of upper cells to the lower chamber. And further invasion assay confirmed the role of FNDC3B expression in PC cells invasion ability (Fig. 7E and F). To elucidate the underlying mechanism by which FNDC3B affects proliferation and migration in PC cells, western blot analyses were conducted to assess changes in the expression levels of relevant biomarkers. We found that downregulation of FNDC3B led to an increase in E-cadherin expression while decreasing N-cadherin expression, suggesting a potential influence on epithelial-mesenchymal transition (EMT). Additionally, cell cycle regulation may also be involved in mediating the effects of FNDC3B on proliferation. Western blot analyses revealed a significant decrease in cyclin B1 and CDK1 expression upon knockdown of FNDC3B, while significant increase in P21 expression (Fig. 7G).

## Discussion

ECM proteins have long been believed to regulate metastasis and chemoresistance in PC, but their role in PC progression may be equivocal. Matrix-metalloprotease-cleaved Col1 (cCol1) and intact Col1 (iCol) play opposite roles in PC progression: cCol1 activates the discoidin domain receptor (DDR)1-NF-κB-p62-NRF2 signaling pathway, while iCol1 degrades DDR and disrupts this pathway [25]. Collagen IV, a crucial component of ECM in PC, has recently been found to upregulate in PC and is regulated by YAP which can promote the metastasis and invasion of PC cells while also being associated with poor prognosis for patients with PC [26]. Additionally, hyperglycemia could activate the YAP/TAZ signaling pathway in cancer-associated fibroblasts to upregulate expression of fibronectin, fibroblast activation protein, COL1A1 and COL11A1, to enhance EMT and chemoresistance of PC [27]. Moreover, bioinformatic analysis identified COL12A1 and MMP14 as prognosis-related genes in PC [28]. RRM1, an enzyme involved in DNA synthesis pathway was reported to upregulate in PC which could promote ECM remodeling leading to enhanced invasion and migration of PC cells through induction of expression of ECM-related genes such as N-cadherin, tenascin-C, and COL11A [29]. FNDC3B is a typical fibronectin-related protein that plays a vital role in tumor progression by activating downstream signaling pathways. In cervical cancer, FNDC3B can promote the ability of proliferation and metastasis of tumor cells through activating PI3K/mTOR signaling pathway [13]. Furthermore, several miRNAs were found to target FNDC3B expression, such as miR-1225-5p, miR-4262 and miR-186-5p [13, 30–32]. However, the role of FNDC3B in PC progression has not been elucidated yet.

In our current study, we assessed the expression of FNDC3B in PC using multiple databases, consistently revealing significantly higher levels of FNDC3B in PC tissues compared to normal pancreatic tissues. Western blot analyses further confirmed elevated FNDC3B expression in PC cell lines relative to normal pancreatic ductal epithelial cells, suggesting a potential role for FNDC3B as a tumor promoter in PC. Given the well-established involvement of ECM in tumor progression, it is plausible that FNDC3B may function as a tumor promoter across various malignancies. Additionally, we validated the diagnostic value of FNDC3B in PC using ROC curve analysis which was significantly higher than traditional tumor marker of PC, such as CA19-9 for diagnosis (0.896 *vs.* 0.83) [33]. Subsequently, we evaluated the prognostic significance of FNDC3B in PC and found that increased expression was significantly associated with shorter OS, DSS and PFI. Although multivariate Cox analysis did not identify FNDC3B expression as an independent prognostic factor among PC patients, univariate analysis revealed its significant association with clinicopathological factors. Furthermore, combining FNDC3B with five other clinicopathological parameters yielded a predictive model with superior prognostic efficacy compared to using only FNDC3B alone. Notably, previous studies have also suggested the potential utility of FNDC3B as a prognostic biomarker across several cancers including cervical cancer, hepatocellular carcinoma, colorectal cancer and glioblastoma [32, 34–36]. Therefore, it can be considered that FNDC3B may serve as a valuable prognostic biomarker across pan-cancer.

To further elucidate the potential biological functions in PC, GO, KEGG analyses and GSEA were employed. The results of GO and KEGG analyses indicated that FNDC3B may participate in nutrient substance digestion processes as well as the activity of several digestive enzymes. Additionally, GSEA results revealed that FNDC3B could be involved in key pathways such as ‘collagen formation’, ‘ECM receptor interaction’, and ‘integrin cell surface interactions’. Furthermore, cell functional assays confirmed the regulatory role of FNDC3B in EMT of PC cells. Given the dense stroma of PC, glycolysis emerges as the major pattern for energy supply to PC cells. Obviously, there exists a close association between glycolysis and tumor metastasis. A recent study demonstrated that brusatol could inhibit the malignant behaviors of colon cancer by upregulating ARRDC4 expression-a cancer glycolytic inhibitor- while modulating PI3K/YAP1/TAZ pathway [37]. Another study also highlighted that hyperglycaemia could stimulate fibronectin expression in PC [25]. Therefore, it is plausible to suggest that FNDC3B activates glycose-metabolizing enzymes to enhance energy utilization and subsequently promote progression of PC.

Tumor-infiltrating immune cells (TIICs) are believed to play intricate roles in cancer progression, PC included. Therefore, we further investigated the association between FNDC3B expression and the abundance of TIICs in PC via ssGSEA. In this study, we observed a significant positive correlation between FNDC3B expression and the infiltration of Th2 cells, macrophages and neutrophils. It has been reported that certain cytokines, such as interleukin-4 (IL-4), IL-5, and IL-13, secreted by Th2 cells could stimulate tumor growth [38]. Additionally, Th2 cells have been shown to mediate chemotherapeutic resistance, especially in FOLFIRINOX-treated PC patients [39]. Furthermore, PC tissues exhibit high infiltration of Th2 cells and M2 type tumor-associated macrophages [40, 41]. A recent study indicated that tumor-infiltrating neutrophils could upregulate glycolytic factors and HIF-1α expression which then promoted PC progression [42]. Moreover, metastatic PC cells infiltrated by neutrophils express Gas6 which activates AXL receptor on tumor cells to facilitate their growth [43]. Furthermore, pDC and Th17 cells showed a negative correlation with FNDC3B expression in PC. Th17 cells are known as interleukin-17-producing CD4 + T cells that mediate host defense mechanisms but show relatively low expression levels in PC tissues [44]. Recent studies have suggested that immune checkpoint inhibitors therapy like nivolumab and pembrolizumab could enhance the infiltration of Th17cells and improve OS for resected PC patients [45, 46]. Besides, pDCs play an important role in tumor immune surveillance of PC, and the scarcity of pDCs can lead to immune surveillance dysfunction [47]. Our study demonstrated a significant negative correlation between high FNDC3B expression and immunotherapy response, which further supported the aforementioned findings. Therefore, combining ECM-targeted therapy with immunotherapy can effectively overcome the limitations of immunotherapy alone. A recent study developed a nanodrug that incorporates calcipotriol and anti-CXCL12 siRNA to simultaneously deactivate matrix-producing pancreatic stellate cells and suppress the infiltration of regulatory T cells, thereby enhancing the efficacy of anti-PD-1 antibodies in PC [48]. The potential combination of anti-FNDC3B therapy with immunotherapy holds promise for future PC treatment.

As far as our knowledge extends, the present study stands as a pioneering endeavor in evaluating the expression, prognostic value, biological functions, and immune infiltration of FNDC3B in PC. However, it is crucial to acknowledge certain limitations that still persist. Firstly, the sample size of public databases remains relatively modest and may potentially introduce bias into our findings. Henceforth, acquiring a more expansive sample size becomes imperative for conducting a more precise analysis. Secondly, while primary studies have indicated the role of FNDC3B in PC progression, it is equally essential to conduct comprehensive molecular mechanism studies to validate the results derived from bioinformatic analysis. Lastly but not least, considering the therapeutic potential of FNDC3B in PC contextually necessitates undertaking preclinical and clinical trials to substantiate its efficacy.

## Conclusion

In conclusion, this study comprehensively investigated the expression, prognostic value, clinicopathological correlation, immune cells infiltration correlation, biological and functional pathway of FNDC3B in PC. The findings suggest that FNDC3B plays a crucial role as a tumor promoter in PC progression and holds immense potential as a therapeutic target for PC treatment.

### Supplementary Information

**Additional file 1: Figure S1.** Immune analysis. T cell exclusion (**A**), T cell dysfunction (**B**) and TIDE score (**C**) in the FNDC3B high and low groups.

**Additional file 2: Table S1.** The baseline characteristics of patients in TCGA-PAAD cohort.

**Additional file 3.** Table S2. The detailed information of 550 significantly DEGs.

**Additional file 4.** Table S3. The detailed result of GO and KEGG enrichment analysis.

**Additional file 5.** Table S4. The detailed result of GSEA analysis.

**Additional file 6: Table S5.** The prognostic value of FNDC3B (Disease Specific Survival) in various PAAD subgroups.

**Additional file 7: Table S6. **The prognostic value of FNDC3B (Progress Free Interval) in various PC subgroups.

**Additional file 8: Table S7. **Univariate and multivariate Cox analysis (Disease Specific Survival) of prognostic covariates in patients with PC.

**Additional file 9: Table S8. **Univariate and multivariate Cox analysis (Progress Free Interval) of prognostic covariates in patients with PC.

## Data Availability

Data will be made available on request.

## References

[1] Siegel RL, Giaquinto AN, Jemal A (2024). Cancer statistics, 2024. CA Cancer J Clin.

[2] Xia C, Dong X, Li H, Cao M, Sun D, He S (2022). Cancer statistics in China and United States, 2022: profiles, trends, and determinants. Chin Med J.

[3] Qiu J, Feng M, Yang G, Cao Z, Liu Y, You L (2023). mTOR inhibitor, gemcitabine and PD-L1 antibody blockade combination therapy suppresses pancreatic cancer progression via metabolic reprogramming and immune microenvironment remodeling in Trp53flox/+LSL-KrasG12D/+Pdx-1-Cre murine models. Cancer Lett.

[4] Bockorny B, Grossman JE, Hidalgo M (2022). Facts and hopes in immunotherapy of pancreatic cancer. Clin Cancer Res.

[5] Wang Y, Wang H, Zhou L, Lu J, Jiang B, Liu C (2020). Photodynamic therapy of pancreatic cancer: where have we come from and where are we going?. Photodiagnosis Photodyn Ther.

[6] Chen Y, Yang S, Tavormina J, Tampe D, Zeisberg M, Wang H (2022). Oncogenic collagen I homotrimers from cancer cells bind to α3β1 integrin and impact tumor microbiome and immunity to promote pancreatic cancer. Cancer Cell.

[7] Egeblad M, Rasch MG, Weaver VM (2010). Dynamic interplay between the collagen scaffold and tumor evolution. Curr Opin Cell Biol.

[8] Zollinger AJ, Smith ML (2017). Fibronectin, the extracellular glue. Matrix Biol.

[9] Zhang X, Luo Y, Cen Y, Qiu X, Li J, Jie M (2022). MACC1 promotes pancreatic cancer metastasis by interacting with the EMT regulator SNAI1. Cell Death Dis.

[10] Xavier CPR, Castro I, Caires HR, Ferreira D, Cavadas B, Pereira L (2021). Chitinase 3-like-1 and fibronectin in the cargo of extracellular vesicles shed by human macrophages influence pancreatic cancer cellular response to gemcitabine. Cancer Lett.

[11] Kishimoto K, Kato A, Osada S, Nishizuka M, Imagawa M (2010). Fad104, a positive regulator of adipogenesis, negatively regulates osteoblast differentiation. Biochem Biophys Res Commun.

[12] Zhang L, Yu H, Deng T, Ling L, Wen J, Lv M (2021). FNDC3B and BPGM are involved in human papillomavirus-mediated carcinogenesis of cervical cancer. Front Oncol.

[13] Li Y, Meng F, Sui C, Wang Y, Cheng D (2023). CircRNA hsa_circ_0001627 aggravates cervical cancer progression through upregulation of FNDC3B and activating PI3K/mTOR signaling pathway. J Cell Commun Signal.

[14] Wang X, Huang Y, Li S, Zhang H (2022). Integrated machine learning methods identify FNDC3B as a potential prognostic biomarker and correlated with immune infiltrates in glioma. Front Immunol.

[15] Wang GH, Wang LY, Zhang C, Zhang P, Wang CH, Cheng S (2020). MiR-1225-5p acts as tumor suppressor in glioblastoma via targeting FNDC3B. Open Med.

[16] Xu H, Hu Y, Qiu W (2017). Potential mechanisms of microRNA-129-5p in inhibiting cell processes including viability, proliferation, migration and invasiveness of glioblastoma cells U87 through targeting FNDC3B. Biomed Pharmacother.

[17] Qi ZY, Wang LL, Qu XL (2021). lncRNA LINC00355 acts as a novel biomarker and promotes glioma biological activities via the regulation of miR-1225/FNDC3B. Dis Markers.

[18] Li YQ, Chen Y, Xu YF, He QM, Yang XJ, Li YQ (2020). FNDC3B 3'-UTR shortening escapes from microRNA-mediated gene repression and promotes nasopharyngeal carcinoma progression. Cancer Sci.

[19] Yu G, Wang LG, Han Y, He QY (2012). clusterProfiler: an R package for comparing biological themes among gene clusters. OMICS.

[20] Bindea G, Mlecnik B, Tosolini M, Kirilovsky A, Waldner M, Obenauf AC (2013). Spatiotemporal dynamics of intratumoral immune cells reveal the immune landscape in human cancer. Immunity.

[21] Jiang P, Gu S, Pan D, Fu J, Sahu A, Hu X (2018). Signatures of T cell dysfunction and exclusion predict cancer immunotherapy response. Nat Med.

[22] Wang Y, Zhou L, Lu J, Jiang B, Liu C, Liang Z (2020). Ubiquitin-specific protease 4 predicts an unfavorable prognosis and promotes malignant behaviors in vitro in pancreatic cancer. Exp Cell Res.

[23] Wang Y, Yuan D, Zhou L, Liang Z, Zhou W, Lu J (2020). Transducin-like enhancer of split-1 inhibits malignant behaviors in vitro and predicts a better prognosis in pancreatic ductal adenocarcinoma. Front Oncol.

[24] Torphy RJ, Schulick RD, Zhu Y (2020). Understanding the immune landscape and tumor microenvironment of pancreatic cancer to improve immunotherapy. Mol Carcinog.

[25] Su H, Yang F, Fu R, Trinh B, Sun N, Liu J (2022). Collagenolysis-dependent DDR1 signalling dictates pancreatic cancer outcome. Nature.

[26] Papalazarou V, Drew J, Juin A, Spence HJ, Whitelaw J, Nixon C (2022). Collagen VI expression is negatively mechanosensitive in pancreatic cancer cells and supports the metastatic niche. J Cell Sci.

[27] Liu Z, Hayashi H, Matsumura K, Ogata Y, Sato H, Shiraishi Y (2023). Hyperglycaemia induces metabolic reprogramming into a glycolytic phenotype and promotes epithelial-mesenchymal transitions via YAP/TAZ-Hedgehog signalling axis in pancreatic cancer. Br J Cancer.

[28] Ding J, Liu Y, Lai Y (2020). Identifying MMP14 and COL12A1 as a potential combination of prognostic biomarkers in pancreatic ductal adenocarcinoma using integrated bioinformatics analysis. PeerJ.

[29] Ono H, Murase Y, Yamashita H, Kato T, Asano D, Ishikawa Y (2023). RRM1 is mediated by histone acetylation through gemcitabine resistance and contributes to invasiveness and ECM remodeling in pancreatic cancer. Int J Oncol.

[30] Yue M, Liu Y, Zuo T, Jiang Y, Pan J, Zhang S (2022). Circ_0006948 contributes to cell growth, migration, invasion and epithelial-mesenchymal transition in esophageal carcinoma. Dig Dis Sci.

[31] Zhang J, Peng Y, Jiang S, Li J (2022). Hsa_circRNA_0001971 contributes to oral squamous cell carcinoma progression via miR-186-5p/Fibronectin type III domain containing 3B axis. J Clin Lab Anal.

[32] Han B, Wang H, Zhang J, Tian J (2020). FNDC3B is associated with ER stress and poor prognosis in cervical cancer. Oncol Lett.

[33] Boyd LNC, Ali M, Leeflang MMG, Treglia G, de Vries R, Le Large TYS (2022). Diagnostic accuracy and added value of blood-based protein biomarkers for pancreatic cancer: a meta-analysis of aggregate and individual participant data. EClinicalMedicine.

[34] Lin CH, Lin YW, Chen YC, Liao CC, Jou YS, Hsu MT (2016). FNDC3B promotes cell migration and tumor metastasis in hepatocellular carcinoma. Oncotarget.

[35] Li Y, Yang J, Wang H, Qiao W, Guo Y, Zhang S (2020). FNDC3B, targeted by miR-125a-5p and miR-217, promotes the proliferation and invasion of colorectal cancer cells via PI3K/mTOR signaling. Onco Targets Ther.

[36] Kwon H, Yun M, Kwon TH, Bang M, Lee J, Lee YS (2023). Fibronectin type III domain containing 3B as a potential prognostic and therapeutic biomarker for glioblastoma. Biomedicines.

[37] Huang QH, Zhang J, Cho WCS, Huang Y, Yang W, Zuo Z (2023). Brusatol suppresses the tumor growth and metastasis of colorectal cancer via upregulating ARRDC4 expression through modulating PI3K/YAP1/TAZ Pathway. Phytomedicine.

[38] Alam A, Levanduski E, Denz P, Villavicencio HS, Bhatta M, Alhorebi L (2022). Fungal mycobiome drives IL-33 secretion and type 2 immunity in pancreatic cancer. Cancer Cell.

[39] Peng H, James CA, Cullinan DR, Hogg GD, Mudd JL, Zuo C (2021). Neoadjuvant FOLFIRINOX therapy is associated with increased effector t cells and reduced suppressor cells in patients with pancreatic cancer. Clin Cancer Res.

[40] De Monte L, Reni M, Tassi E, Clavenna D, Papa I, Recalde H (2011). Intratumor T helper type 2 cell infiltrate correlates with cancer-associated fibroblast thymic stromal lymphopoietin production and reduced survival in pancreatic cancer. J Exp Med.

[41] Kurahara H, Takao S, Maemura K, Mataki Y, Kuwahata T, Maeda K (2013). M2-polarized tumor-associated macrophage infiltration of regional lymph nodes is associated with nodal lymphangiogenesis and occult nodal involvement in pN0 pancreatic cancer. Pancreas.

[42] Sieow JL, Penny HL, Gun SY, Tan LQ, Duan K, Yeong JPS (2023). Conditional knockout of hypoxia-inducible factor 1-alpha in tumor-infiltrating neutrophils protects against pancreatic ductal adenocarcinoma. Int J Mol Sci.

[43] Bellomo G, Rainer C, Quaranta V, Astuti Y, Raymant M, Boyd E (2022). Chemotherapy-induced infiltration of neutrophils promotes pancreatic cancer metastasis via Gas6/AXL signalling axis. Gut.

[44] Hong HS, Mbah NE, Shan M, Loesel K, Lin L, Sajjakulnukit P (2022). OXPHOS promotes apoptotic resistance and cellular persistence in TH17 cells in the periphery and tumor microenvironment. Sci Immunol.

[45] Li K, Tandurella JA, Gai J, Zhu Q, Lim SJ, Thomas DL (2022). Multi-omic analyses of changes in the tumor microenvironment of pancreatic adenocarcinoma following neoadjuvant treatment with anti-PD-1 therapy. Cancer Cell.

[46] Wang J, Gai J, Zhang T, Niu N, Qi H, Thomas DL (2024). Neoadjuvant radioimmunotherapy in pancreatic cancer enhances effector T cell infiltration and shortens their distances to tumor cells. Sci Adv.

[47] Hegde S, Krisnawan VE, Herzog BH, Zuo C, Breden MA, Knolhoff BL (2020). Dendritic cell paucity leads to dysfunctional immune surveillance in pancreatic cancer. Cancer Cell.

[48] Wang R, Hong K, Zhang Q, Cao J, Huang T, Xiao Z (2023). A nanodrug simultaneously inhibits pancreatic stellate cell activation and regulatory T cell infiltration to promote the immunotherapy of pancreatic cancer. Acta Biomater.

