# Supplementary figures and images for: Identification of fibronectin type III domain containing 3B as a potential prognostic and therapeutic target for pancreatic cancer: a preliminary analysis
Source: Eur J Med Res. 2024 Apr 5;29:221. doi: 10.1186/s40001-024-01823-6 (PMC10996089; doi:10.1186/s40001-024-01823-6)

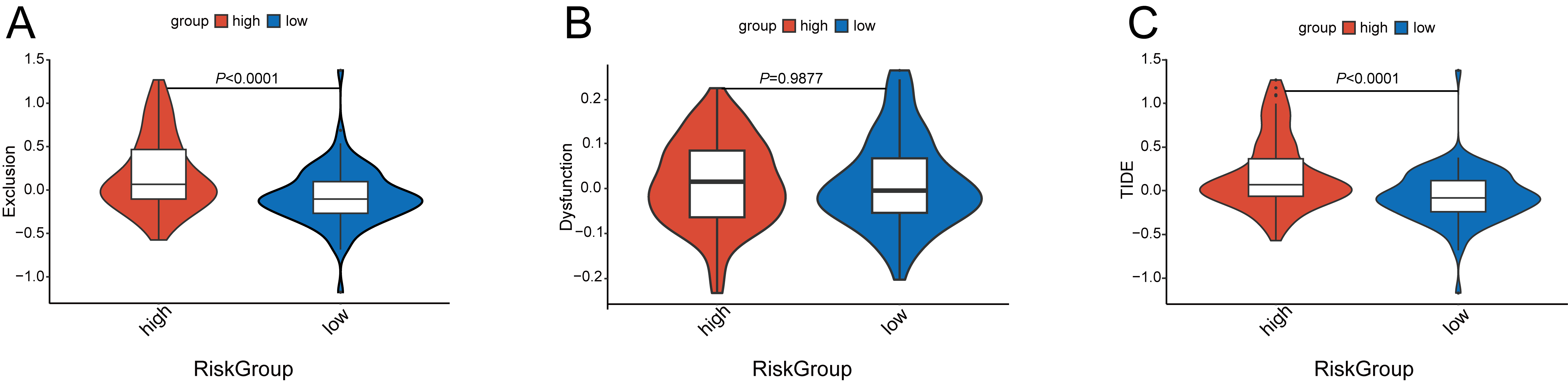

Supplement: Supplementary file 1 — Additional file 1: Figure S1. Immune analysis. T cell exclusion (A), T cell dysfunction (B) and TIDE score (C) in the FNDC3B high and low groups. [file 40001_2024_1823_MOESM1_ESM.tif]
