# Supplementary material for: Identification of fibronectin type III domain containing 3B as a potential prognostic and therapeutic target for pancreatic cancer: a preliminary analysis
Source: Eur J Med Res. 2024 Apr 5;29:221. doi: 10.1186/s40001-024-01823-6 (PMC10996089; doi:10.1186/s40001-024-01823-6)
Supplement: Supplementary file 2 — Additional file 2: Table S1. The baseline characteristics of patients in TCGA-PAAD cohort. [file 40001_2024_1823_MOESM2_ESM.docx]

**Table S1.** The baseline characteristics of patients in TCGA-PAAD cohort

| **Characteristic** | **Variables** | **Overall*** |
| --- | --- | --- |
| n |  | 178 |
| T stage, n (%) | T1 | 7 (3.9%) |
|  | T2 | 24 (13.5%) |
|  | T3 | 142 (79.8%) |
|  | T4 | 3 (1.7%) |
| N stage, n (%) | N0 | 49 (28.3%) |
|  | N1 | 124 (69.7%) |
| M stage, n (%) | M0 | 80 (44.9%) |
|  | M1 | 4 (2.2%) |
| Pathologic stage, n (%) | I | 21 (11.8%) |
|  | II | 147 (82.6%) |
|  | III | 3 (1.7%) |
|  | IV | 4 (2.2%) |
| Radiation therapy, n (%) | No | 118 (66.3%) |
|  | Yes | 45 (25.3%) |
| Primary therapy outcome, n (%) | PD | 50 (28.1%) |
|  | SD | 9 (5.1%) |
|  | PR | 10 (5.6%) |
|  | CR | 71 (39.9%) |
| Age, n (%) | <=65 | 94 (52.8%) |
|  | >65 | 84 (47.2%) |
| Race, n (%) | Asian | 11 (6.2%) |
|  | Black or African American | 6 (3.4%) |
|  | White | 157 (88.2%) |
| Gender, n (%) | Female | 80 (44.9%) |
|  | Male | 98 (55.1%) |
| Histologic grade, n (%) | G1 | 31 (17.4%) |
|  | G2 | 95 (53.4%) |
|  | G3 | 48 (27%) |
|  | G4 | 2 (1.1%) |
| Residual tumor, n (%) | R0 | 106 (59.6%) |
|  | R1 | 53 (29.8%) |
|  | R2 | 5 (2.8%) |
| Anatomic neoplasm subdivision, n (%) | Head of Pancreas | 139 (78.1%) |
|  | Other | 39 (21.9%) |
| Alcohol history, n (%) | No | 64 (38.6%) |
|  | Yes | 102 (61.4%) |
| Smoker, n (%) | No | 66 (45.5%) |
|  | Yes | 79 (54.5%) |
| History of diabetes, n (%) | No | 109 (61.2%) |
|  | Yes | 38 (21.3%) |
| History of chronic pancreatitis, n (%) | No | 129 (72.5%) |
|  | Yes | 13 (7.3%) |

*The total patient number does not equal to 178 for all variables owing to a lack of patient information in some cases. CR, complete response; G1: well-differentiated; G2: moderately-differentiated; G3: poorly-differentiated; G4: undifferentiated; M: metastasis; N: lymph node; PAAD, pancreatic adenocarcinoma; PR, partial response; PD, progressive disease; SD, stable disease; T: tumor.
