# Supplementary material for: Identification of fibronectin type III domain containing 3B as a potential prognostic and therapeutic target for pancreatic cancer: a preliminary analysis
Source: Eur J Med Res. 2024 Apr 5;29:221. doi: 10.1186/s40001-024-01823-6 (PMC10996089; doi:10.1186/s40001-024-01823-6)
Supplement: Supplementary file 4 — Additional file 4. Table S3. The detailed result of GO and KEGG enrichment analysis. [file 40001_2024_1823_MOESM4_ESM.pdf]

ONTOLOGY	ID	Description	GeneRatio	BgRatio	pvalue	p.adjust
BP	GO:0007586	digestion	18/224	137/18800	4.7376E-14	1.0432E-10
BP	GO:0035821	modulation of	6/224	25/18800	3.9215E-07	0.00043176
BP	GO:0019730	antimicrobial	10/224	122/18800	2.0312E-06	0.00149092
BP	GO:0061844	antimicrobial	8/224	79/18800	4.5209E-06	0.00201191
BP	GO:0032094	response to fc	6/224	37/18800	4.5684E-06	0.00201191
BP	GO:0060402	calcium ion tr	11/224	171/18800	6.6836E-06	0.00245289
BP	GO:0060401	cytosolic calci	11/224	190/18800	1.808E-05	0.00568747
BP	GO:0048172	regulation of s	4/224	15/18800	2.4159E-05	0.00604342
BP	GO:0031667	response to n	17/224	446/18800	2.6644E-05	0.00604342
BP	GO:0048168	regulation of r	6/224	50/18800	2.7445E-05	0.00604342
BP	GO:0010522	regulation of c	8/224	103/18800	3.2261E-05	0.00645816
BP	GO:0010524	positive regula	6/224	54/18800	4.2872E-05	0.00786709
BP	GO:0006816	calcium ion tr	16/224	424/18800	5.199E-05	0.00880626
BP	GO:0009991	response to ex	17/224	479/18800	6.4791E-05	0.01019077
BP	GO:0002385	mucosal immu	5/224	38/18800	8.366E-05	0.01228125
BP	GO:0072503	cellular divale	17/224	494/18800	9.4327E-05	0.01297112
BP	GO:0044241	lipid digestion	4/224	21/18800	0.00010014	0.01297112
BP	GO:0006959	humoral immu	13/224	317/18800	0.00011661	0.01406154
BP	GO:0002251	organ or tissu	5/224	41/18800	0.00012133	0.01406154
BP	GO:0007204	positive regula	13/224	325/18800	0.00014925	0.01643269
BP	GO:0022600	digestive syste	7/224	104/18800	0.00024776	0.02597889
BP	GO:0051480	regulation of c	13/224	356/18800	0.00036088	0.03612082
BP	GO:0006874	cellular calciu	15/224	456/18800	0.00039322	0.03764654
BP	GO:0002526	acute inflamm	7/224	113/18800	0.00041158	0.03776253
BP	GO:0030157	pancreatic jui	3/224	13/18800	0.00043702	0.03849247
BP	GO:0046883	regulation of h	10/224	231/18800	0.00046843	0.03967221
BP	GO:0055074	calcium ion hc	15/224	468/18800	0.00051521	0.04201846
BP	GO:0014733	regulation of s	3/224	14/18800	0.00055133	0.04335788
BP	GO:1902656	calcium ion im	4/224	33/18800	0.00061214	0.04642051
BP	GO:0048167	regulation of s	9/224	198/18800	0.00063243	0.04642051
BP	GO:0045666	positive regula	6/224	91/18800	0.00076785	0.05283756
BP	GO:0070509	calcium ion im	6/224	91/18800	0.00076785	0.05283756
BP	GO:0051924	regulation of c	10/224	251/18800	0.0008892	0.05933362
BP	GO:0009311	oligosaccharic	5/224	63/18800	0.00092045	0.05961246
BP	GO:1904062	regulation of c	12/224	352/18800	0.00109884	0.06913273
BP	GO:0010959	regulation of r	13/224	403/18800	0.00113911	0.0696758
BP	GO:0090276	regulation of p	8/224	177/18800	0.00130432	0.0776249
BP	GO:0002791	regulation of p	8/224	180/18800	0.00145149	0.08411027
BP	GO:0050830	defense respo	6/224	104/18800	0.00153837	0.08685892
BP	GO:0090087	regulation of p	8/224	183/18800	0.00161156	0.08871628
CC	GO:0045095	keratin filame	8/248	102/19594	4.6318E-05	0.00721348
CC	GO:0005796	Golgi lumen	8/248	104/19594	5.3236E-05	0.00721348
CC	GO:0042589	zymogen gran	3/248	11/19594	0.00030661	0.02321485
CC	GO:0043195	terminal bout	5/248	48/19594	0.00034265	0.02321485
CC	GO:0042588	zymogen gran	3/248	14/19594	0.00065766	0.03564491
CC	GO:0008328	ionotropic glu	4/248	40/19594	0.00160062	0.07229489
CC	GO:0098878	neurotransmit	4/248	45/19594	0.00248396	0.09616492
MF	GO:0008236	serine-type pe	16/228	191/18410	2.0925E-09	5.2478E-07
MF	GO:0017171	serine hydrola	16/228	195/18410	2.829E-09	5.2478E-07

MF	GO:0004252	serine-type er	15/228	174/18410	4.5643E-09	5.6446E-07
MF	GO:0004806	triglyceride lip	5/228	26/18410	1.4833E-05	0.00137579
MF	GO:0004867	serine-type er	8/228	98/18410	2.957E-05	0.00219408
MF	GO:0031406	carboxylic acid	10/228	173/18410	6.0965E-05	0.00324215
MF	GO:0042834	peptidoglycan	4/228	18/18410	6.1173E-05	0.00324215
MF	GO:0004175	endopeptidase	16/228	432/18410	0.0001017	0.00471634
MF	GO:0004553	hydrolase acti	7/228	94/18410	0.00016731	0.00622423
MF	GO:0005539	glycosaminogl	11/228	234/18410	0.00016777	0.00622423
MF	GO:0019865	immunoglobu	4/228	24/18410	0.00020039	0.00675849
MF	GO:0004181	metallocarbox	4/228	29/18410	0.00042669	0.0131918
MF	GO:0031404	chloride ion b	3/228	13/18410	0.00048916	0.01395986
MF	GO:0070492	oligosaccharic	3/228	15/18410	0.00076409	0.02023052
MF	GO:0005179	hormone activ	7/228	122/18410	0.00081795	0.02023052
MF	GO:0004866	endopeptidase	8/228	180/18410	0.00184855	0.04169443
MF	GO:0030246	carbohydrate	10/228	270/18410	0.00204061	0.04169443
MF	GO:0004180	carboxypeptic	4/228	44/18410	0.00210968	0.04169443
MF	GO:0016798	hydrolase acti	7/228	144/18410	0.00213529	0.04169443
MF	GO:0030414	peptidase inhi	8/228	187/18410	0.00234499	0.04349953
MF	GO:0052689	carboxylic este	7/228	149/18410	0.00258789	0.04571945
MF	GO:0061135	endopeptidase	8/228	194/18410	0.00294049	0.04958731
MF	GO:0048018	receptor ligan	14/228	489/18410	0.00322225	0.05197628
MF	GO:0005104	fibroblast grov	3/228	25/18410	0.00352574	0.05423887
MF	GO:0030546	signaling rece	14/228	496/18410	0.00365491	0.05423887
MF	GO:0016298	lipase activity	6/228	130/18410	0.0056393	0.08046848
MF	GO:0005432	calcium:sodiu	2/228	10/18410	0.00643628	0.08528072
MF	GO:0015643	toxic substanc	2/228	10/18410	0.00643628	0.08528072
MF	GO:0015368	calcium:cation	2/228	11/18410	0.00780269	0.09921274
MF	GO:0061134	peptidase reg	8/228	230/18410	0.00806876	0.09921274
MF	GO:0043177	organic acid b	6/228	141/18410	0.00829001	0.09921274
KEGG	hsa04972	Pancreatic sec	21/102	102/8164	1.8004E-20	3.2407E-18
KEGG	hsa04974	Protein digest	14/102	103/8164	2.4992E-11	2.2493E-09
KEGG	hsa04975	Fat digestion a	6/102	43/8164	1.3754E-05	0.00082526
KEGG	hsa00500	Starch and suc	5/102	36/8164	7.6465E-05	0.00344094
KEGG	hsa04080	Neuroactive li	14/102	362/8164	0.00015015	0.00540522
KEGG	hsa04973	Carbohydrate	5/102	47/8164	0.00027907	0.008372
KEGG	hsa04640	Hematopoieti	6/102	99/8164	0.00143944	0.0370143
KEGG	hsa04971	Gastric acid se	5/102	76/8164	0.00252642	0.05684438
KEGG	hsa04020	Calcium signal	9/102	240/8164	0.00299926	0.05998525

qvalue	geneID	Count
1.0069E-10	AMY2A/ARX/(	18
0.00041671	DEFA5/DEFA6	6
0.00143896	DEFA5/DEFA6	10
0.00194179	DEFA5/DEFA6	8
0.00194179	CLPS/CLPSL1/	6
0.00236741	CD19/EPO/F2,	11
0.00548927	CD19/EPO/F2,	11
0.00583281	SHISA7/SHISA	4
0.00583281	FGF23/ALB/BI	17
0.00583281	CAMK2B/NEU	6
0.0062331	CD19/EPO/F2,	8
0.00759293	CD19/F2/GRII	6
0.00849937	BHLHA15/CA(	16
0.00983563	FGF23/ALB/BI	17
0.01185327	DEFA5/DEFA6	5
0.0125191	CCKBR/CD19/	17
0.0125191	ARX/CEL/PNLI	4
0.01357151	DEFA5/DEFA6	13
0.01357151	DEFA5/DEFA6	5
0.01586004	CCKBR/CD19/	13
0.02507356	CCKBR/CEL/M	7
0.03486206	CCKBR/CD19/	13
0.03633461	CCKBR/CD19/	15
0.03644655	IL20RB/C2CD4	7
0.03715106	CEL/SPINK1/V	3
0.03828968	FGF23/BLK/BI	10
0.04055418	CCKBR/CD19/	15
0.04184692	CAMK2B/MIR	3
0.04480281	MIR208B/MS4	4
0.04480281	CAMK2B/CPL)	9
0.05099624	ATOH1/BRINP	6
0.05099624	MIR208B/MS4	6
0.05726592	CAMK2B/CD1	10
0.05753504	AMY1A/AMY1	5
0.06672354	CACNG7/CD1!	12
0.06724769	CAMK2B/CD1	13
0.07491978	BLK/BRSK2/CI	8
0.08117914	BLK/BRSK2/CI	8
0.083832	DEFA5/DEFA6	6
0.08562463	BLK/BRSK2/CI	8
0.0066405	CASP14/KRT1	8
0.0066405	FGF23/MUC1(	8
0.02137083	CUZD1/GP2/Z	3
0.02137083	CPLX2/GHRH/	5
0.03281353	CUZD1/GP2/Z	3
0.06655229	CACNG7/GRII	4
0.08852626	CACNG7/GRII	4
4.72E-07	MMP1/CELA2	16
4.72E-07	MMP1/CELA2	16

5.0768E-07	MMP1/CELA2	15
0.00123741	LIPK/CEL/PNLI	5
0.00197339	A2ML1/ITIH1/	8
0.00291605	SIGLEC6/AFP/	10
0.00291605	REG1A/REG3A	4
0.00424196	CASP14/MMP	16
0.00559819	AMY1A/AMY1	7
0.00559819	COL11A1/EPY	11
0.00607871	CD22/FCER2/I	4
0.01186495	CPA1/CPA2/C	4
0.01255575	AMY1A/AMY1	3
0.0181957	REG1A/REG3A	3
0.0181957	EPO/GAST/GH	7
0.03750073	A2ML1/ITIH1/	8
0.03750073	SIGLEC6/CD22	10
0.03750073	CPA1/CPA2/C	4
0.03750073	AMY1A/AMY1	7
0.03912427	A2ML1/ITIH1/	8
0.04112091	LIPK/CEL/PLA2	7
0.04459973	A2ML1/ITIH1/	8
0.04674842	FGF23/EREG/I	14
0.04878343	FGF23/FGF19/	3
0.04878343	FGF23/EREG/I	14
0.07237483	LIPK/CEL/PLA2	6
0.07670304	SLC24A5/SLC8	2
0.07670304	ALB/GUCY2C	2
0.08923376	SLC24A5/SLC8	2
0.08923376	A2ML1/ITIH1/	8
0.08923376	FTCD/GNMT/	6
3.146E-18	AMY1A/AMY1	21
2.1835E-09	COL11A1/CEL	14
0.00080113	CEL/CLPS/PLA	6
0.00334032	AMY1A/AMY1	5
0.00524718	CCKBR/F2/GH	14
0.00812721	AMY1A/AMY1	5
0.03593201	CD19/CD22/E	6
0.05518226	ATP4A/CALMI	5
0.05823129	FGF23/FGF19/	9
