# Supplementary material for: Identification of fibronectin type III domain containing 3B as a potential prognostic and therapeutic target for pancreatic cancer: a preliminary analysis
Source: Eur J Med Res. 2024 Apr 5;29:221. doi: 10.1186/s40001-024-01823-6 (PMC10996089; doi:10.1186/s40001-024-01823-6)
Supplement: Supplementary file 5 — Additional file 5. Table S4. The detailed result of GSEA analysis. [file 40001_2024_1823_MOESM5_ESM.pdf]

ID	Description	setSize
PID_INTEGRIN1_PATHWAY	PID_INTEGRIN1_PATHWAY	66
REACTOME_INTEGRIN_CELL_SURFACE_INTERACTIONS	REACTOME_INTEGRIN_CELL_	84
REACTOME_COLLAGEN_FORMATION	REACTOME_COLLAGEN_FOR	90
REACTOME_ASSEMBLY_OF_COLLAGEN_FIBRILS_AND_OTH	REACTOME_ASSEMBLY_OF_	61
REACTOME_LAMININ_INTERACTIONS	REACTOME_LAMININ_INTER	30
KEGG_ECM_RECEPTOR_INTERACTION	KEGG_ECM_RECEPTOR_INTE	83
REACTOME_MET_ACTIVATES_PTK2_SIGNALING	REACTOME_MET_ACTIVATES	30
REACTOME_ECM_PROTEOGLYCANS	REACTOME_ECM_PROTEOG	76
REACTOME_NON_INTEGRIN_MEMBRANE_ECM_INTERACT	REACTOME_NON_INTEGRIN_	59
REACTOME_ELASTIC_FIBRE_FORMATION	REACTOME_ELASTIC_FIBRE_	44
PID_INTEGRIN3_PATHWAY	PID_INTEGRIN3_PATHWAY	43
WP_GLUCOCORTICOID_RECEPTOR_PATHWAY	WP_GLUCOCORTICOID_RECI	70
REACTOME_MET_PROMOTES_CELL_MOTILITY	REACTOME_MET_PROMOTE	41
PID_AVB3_INTEGRIN_PATHWAY	PID_AVB3_INTEGRIN_PATHV	74
REACTOME_SYNDECAN_INTERACTIONS	REACTOME_SYNDECAN_INTI	27
REACTOME_MOLECULES_ASSOCIATED_WITH_ELASTIC_FIE	REACTOME_MOLECULES_AS	37
REACTOME_COLLAGEN_DEGRADATION	REACTOME_COLLAGEN_DEC	64
REACTOME_SIGNALING_BY_PDGF	REACTOME_SIGNALING_BY_	58
PID_SYNDECAN_1_PATHWAY	PID_SYNDECAN_1_PATHWA	46
REACTOME_COLLAGEN_BIOSYNTHESIS_AND_MODIFYING_	REACTOME_COLLAGEN_BIO	67
REACTOME_SIGNALING_BY_TGF_BETA_RECEPTOR_COMP	REACTOME_SIGNALING_BY_	93
KEGG_SMALL_CELL_LUNG_CANCER	KEGG_SMALL_CELL_LUNG_C	84
KEGG_FOCAL_ADHESION	KEGG_FOCAL_ADHESION	199
PID_FAK_PATHWAY	PID_FAK_PATHWAY	59
WP_BURN_WOUND_HEALING	WP_BURN_WOUND_HEALIN	108
REACTOME_DEGRADATION_OF_THE_EXTRACELLULAR_MA	REACTOME_DEGRADATION_	140
WP_HAIR_FOLLICLE_DEVELOPMENT_CYTODIFFERENTIATI(	WP_HAIR_FOLLICLE_DEVELC	86
WP_TGFBETA_SIGNALING_PATHWAY	WP_TGFBETA_SIGNALING_P	132
WP_SMALL_CELL_LUNG_CANCER	WP_SMALL_CELL_LUNG_CAI	96
REACTOME_SIGNALING_BY_TGFB_FAMILY_MEMBERS	REACTOME_SIGNALING_BY_	122
WP_FOCAL_ADHESION	WP_FOCAL_ADHESION	198
REACTOME_DIGESTION	REACTOME_DIGESTION	23
WP_EBOLA_VIRUS_INFECTION_IN_HOST	WP_EBOLA_VIRUS_INFECTIC	129
NABA_ECM_GLYCOPROTEINS	NABA_ECM_GLYCOPROTEIN	196
KEGG_AXON_GUIDANCE	KEGG_AXON_GUIDANCE	129
KEGG_RIBOSOME	KEGG_RIBOSOME	88
REACTOME_EUKARYOTIC_TRANSLATION_ELONGATION	REACTOME_EUKARYOTIC_TI	94
REACTOME_DIGESTION_AND_ABSORPTION	REACTOME_DIGESTION_ANI	27
WP_CYTOPLASMIC_RIBOSOMAL_PROTEINS	WP_CYTOPLASMIC_RIBOSOI	88
REACTOME_RESPONSE_OF_EIF2AK4_GCN2_TO_AMINO_A	REACTOME_RESPONSE_OF_	102
REACTOME_SRP_DEPENDENT_COTRANSLATIONAL_PROTE	REACTOME_SRP_DEPENDEN	113
REACTOME_EUKARYOTIC_TRANSLATION_INITIATION	REACTOME_EUKARYOTIC_TI	120
REACTOME_SELENOAMINO_ACID_METABOLISM	REACTOME_SELENOAMINO_	118
REACTOME_NONSENSE_MEDIATED_DECAY_NMD	REACTOME_NONSENSE_MEI	116
REACTOME_IMMUNOREGULATORY_INTERACTIONS_BETW	REACTOME_IMMUNOREGUI	186
KEGG_REGULATION_OF_ACTIN_CYTOSKELETON	KEGG_REGULATION_OF_ACT	213
REACTOME_CELLULAR_RESPONSE_TO_STARVATION	REACTOME_CELLULAR_RESP	156
REACTOME_INFLUENZA_INFECTION	REACTOME_INFLUENZA_INF	157
REACTOME_RRNA_PROCESSING	REACTOME_RRNA_PROCESS	203

REACTOME_METABOLISM_OF_AMINO_ACIDS_AND_DERIV	REACTOME_METABOLISM_C	373
REACTOME_TRANSLATION	REACTOME_TRANSLATION	293
NABA_BASEMENT_MEMBRANES	NABA_BASEMENT_MEMBRA	40
WP_EPITHELIAL_TO_MESENCHYMAL_TRANSITION_IN_COI	WP_EPITHELIAL_TO_MESEN	158
REACTOME_SIGNALING_BY_MET	REACTOME_SIGNALING_BY_	79
REACTOME_REGULATION_OF_EXPRESSION_OF_SLITS_ANI	REACTOME_REGULATION_O	172
WP_HIPPOMERLIN_SIGNALING_DYSREGULATION	WP_HIPPOMERLIN_SIGNALI	120
WP_MIR5093P_ALTERATION_OF_YAP1ECM_AXIS	WP_MIR5093P_ALTERATION	18
REACTOME_RAC1_GTPASE_CYCLE	REACTOME_RAC1_GTPASE_(	184
KEGG_TGF_BETA_SIGNALING_PATHWAY	KEGG_TGF_BETA_SIGNALING	85
WP_HYPOTHESIZED_PATHWAYS_IN_PATHOGENESIS_OF_(	WP_HYPOTHESIZED_PATHW	25
PID_PDGFRB_PATHWAY	PID_PDGFRB_PATHWAY	129
REACTOME_DISEASES_OF_GLYCOSYLATION	REACTOME_DISEASES_OF_G	143
REACTOME_RHOA_GTPASE_CYCLE	REACTOME_RHOA_GTPASE_	148
REACTOME_RAC3_GTPASE_CYCLE	REACTOME_RAC3_GTPASE_(	94
PID_NECTIN_PATHWAY	PID_NECTIN_PATHWAY	30
WP_ONCOSTATIN_M_SIGNALING_PATHWAY	WP_ONCOSTATIN_M_SIGNA	65
KEGG_ADHERENS_JUNCTION	KEGG_ADHERENS_JUNCTION	73
WP_ENDOCHONDRAL_OSSIFICATION	WP_ENDOCHONDRAL_OSSIF	63
WP_ENDOCHONDRAL_OSSIFICATION_WITH_SKELETAL_DY	WP_ENDOCHONDRAL_OSSIF	63
PID_AP1_PATHWAY	PID_AP1_PATHWAY	70
REACTOME_RHOQ_GTPASE_CYCLE	REACTOME_RHOQ_GTPASE_	59
PID_P53_DOWNSTREAM_PATHWAY	PID_P53_DOWNSTREAM_PA	137
NABA_COLLAGENS	NABA_COLLAGENS	44
REACTOME_COLLAGEN_CHAIN_TRIMERIZATION	REACTOME_COLLAGEN_CHA	44
REACTOME_CELL_CELL_COMMUNICATION	REACTOME_CELL_CELL_COM	128
PID_MET_PATHWAY	PID_MET_PATHWAY	79
WP_EGFEGFR_SIGNALING_PATHWAY	WP_EGFEGFR_SIGNALING_P	161
REACTOME_ESR_MEDIATED_SIGNALING	REACTOME_ESR_MEDIATED_	218
PID_FRA_PATHWAY	PID_FRA_PATHWAY	37
REACTOME_TGF_BETA_RECEPTOR_SIGNALING_ACTIVATES	REACTOME_TGF_BETA_RECE	46
PID_A6B1_A6B4_INTEGRIN_PATHWAY	PID_A6B1_A6B4_INTEGRIN_	46
REACTOME_FCGAMMA_RECEPTOR_FCGR_DEPENDENT_PI	REACTOME_FCGAMMA_REC	143
REACTOME_RAC2_GTPASE_CYCLE	REACTOME_RAC2_GTPASE_(	88
REACTOME_DIGESTION_OF_DIETARY_CARBOHYDRATE	REACTOME_DIGESTION_OF_	11
WP_PRIMARY_FOCAL_SEGMENTAL_GLOMERULOSCLEROS	WP_PRIMARY_FOCAL_SEGM	72
PID_DELTA_NP63_PATHWAY	PID_DELTA_NP63_PATHWAY	47
REACTOME_INTERFERON_SIGNALING	REACTOME_INTERFERON_SI	200
WP_CCL18_SIGNALING_PATHWAY	WP_CCL18_SIGNALING_PAT	51
REACTOME_CELL_SURFACE_INTERACTIONS_AT_THE_VAS(	REACTOME_CELL_SURFACE_	194
REACTOME_RESPIRATORY_ELECTRON_TRANSPORT_ATP_S	REACTOME_RESPIRATORY_E	114
PID_INTEGRIN_CS_PATHWAY	PID_INTEGRIN_CS_PATHWAY	26
REACTOME_RHOF_GTPASE_CYCLE	REACTOME_RHOF_GTPASE_	42
WP_CLOCKCONTROLLED_AUTOPHAGY_IN_BONE_METAB(	WP_CLOCKCONTROLLED_AU	80
PID_UPA_UPAR_PATHWAY	PID_UPA_UPAR_PATHWAY	42
REACTOME_ACTIVATION_OF_THE_MRNA_UPON_BINDING	REACTOME_ACTIVATION_OF	60
NABA_ECM_REGULATORS	NABA_ECM_REGULATORS	238
WP_DEVELOPMENT_OF_URETERIC_COLLECTION_SYSTEM	WP_DEVELOPMENT_OF_URI	60
PID_SMAD2_3NUCLEAR_PATHWAY	PID_SMAD2_3NUCLEAR_PAT	82
REACTOME_ROLE_OF_LAT2_NTAL_LAB_ON_CALCIUM_M(	REACTOME_ROLE_OF_LAT2_	71

WP_REGULATION_OF_ACTIN_CYTOSKELETON	WP_REGULATION_OF_ACTIN	150
PID_SHP2_PATHWAY	PID_SHP2_PATHWAY	57
WP_ELECTRON_TRANSPORT_CHAIN_OXPHOS_SYSTEM_IN	WP_ELECTRON_TRANSPORT	90
WP_ANGIOGENESIS	WP_ANGIOGENESIS	24
PID_ERBB1_DOWNSTREAM_PATHWAY	PID_ERBB1_DOWNSTREAM_	105
WP_NRP1TRIGGERED_SIGNALING_PATHWAYS_IN_PANCR	WP_NRP1TRIGGERED_SIGN	54
WP_DNA_DAMAGE_RESPONSE_ONLY_ATM_DEPENDENT	WP_DNA_DAMAGE_RESPON	109
WP_MATRIX_METALLOPROTEINASES	WP_MATRIX_METALLOPROT	30
REACTOME_TYPE_I_HEMIDESMOSOME_ASSEMBLY	REACTOME_TYPE_I_HEMIDE	11
WP_AGERAGE_PATHWAY	WP_AGERAGE_PATHWAY	66
REACTOME_RHOC_GTPASE_CYCLE	REACTOME_RHOC_GTPASE_	74
WP_TYPE_I_COLLAGEN_SYNTHESIS_IN_THE_CONTEXT_OF	WP_TYPE_I_COLLAGEN_SYN	33
REACTOME_SEMAPHORIN_INTERACTIONS	REACTOME_SEMAPHORIN_I	64
REACTOME_EXTRA_NUCLEAR_ESTROGEN_SIGNALING	REACTOME_EXTRA_NUCLEA	75
REACTOME_CELL_JUNCTION_ORGANIZATION	REACTOME_CELL_JUNCTION	90
REACTOME_FCERI_MEDIATED_CA_2_MOBILIZATION	REACTOME_FCERI_MEDIATE	86
REACTOME_SIGNALING_BY_ROBO_RECEPTORS	REACTOME_SIGNALING_BY_	218
REACTOME_CDC42_GTPASE_CYCLE	REACTOME_CDC42_GTPASE_	154
REACTOME_FCERI_MEDIATED_MAPK_ACTIVATION	REACTOME_FCERI_MEDIATE	87
REACTOME_O_GLYCOSYLATION_OF_TSR_DOMAIN_CONT	REACTOME_O_GLYCOSYLATI	39
PID_INTEGRIN5_PATHWAY	PID_INTEGRIN5_PATHWAY	17
PID_TGFBR_PATHWAY	PID_TGFBR_PATHWAY	54
WP_MFAP5_EFFECT_ON_PERMEABILITY_AND_MOTILITY_	WP_MFAP5_EFFECT_ON_PE	18
WP_TGFBETA_RECEPTOR_SIGNALING_IN_SKELETAL_DYSP	WP_TGFBETA_RECEPTOR_SI	59
WP_NEOVASCULARISATION_PROCESSES	WP_NEOVASCULARISATION_	37
WP_CANONICAL_AND_NONCANONICAL_TGFB_SIGNALING	WP_CANONICAL_AND_NON	17
REACTOME_SARS_COV_2_MODULATES_HOST_TRANSLATI	REACTOME_SARS_COV_2_M	51
PID_INTEGRIN4_PATHWAY	PID_INTEGRIN4_PATHWAY	11
PID_CDC42_PATHWAY	PID_CDC42_PATHWAY	70
REACTOME_RND1_GTPASE_CYCLE	REACTOME_RND1_GTPASE_	42
WP_TGFBETA_RECEPTOR_SIGNALING	WP_TGFBETA_RECEPTOR_SI	55
PID_ANGIOPOIETIN_RECEPTOR_PATHWAY	PID_ANGIOPOIETIN_RECEPT	49
PID_VEGFR1_2_PATHWAY	PID_VEGFR1_2_PATHWAY	69
PID_LYMPH_ANGIOGENESIS_PATHWAY	PID_LYMPH_ANGIOGENESIS_	25
REACTOME_CROSSLINKING_OF_COLLAGEN_FIBRILS	REACTOME_CROSSLINKING_	18
WP_INTEGRINMEDIATED_CELL_ADHESION	WP_INTEGRINMEDIATED_CE	102
WP_VITAMIN_D_RECEPTOR_PATHWAY	WP_VITAMIN_D_RECEPTOR_	184
REACTOME_RHOJ_GTPASE_CYCLE	REACTOME_RHOJ_GTPASE_	55
KEGG_OXIDATIVE_PHOSPHORYLATION	KEGG_OXIDATIVE_PHOSPHC	118
WP_GASTRIN_SIGNALING_PATHWAY	WP_GASTRIN_SIGNALING_P	115
WP_REGULATION_OF_WNT_BCATENIN_SIGNALING_BY_SI	WP_REGULATION_OF_WNT_	17
WP_OVERVIEW_OF_NANOPARTICLE_EFFECTS	WP_OVERVIEW_OF_NANOP	19
WP_MIRNA_TARGETS_IN_ECM_AND_MEMBRANE_RECEPT	WP_MIRNA_TARGETS_IN_E	39
REACTOME_RESPIRATORY_ELECTRON_TRANSPORT	REACTOME_RESPIRATORY_E	92
WP_ALPHA_6_BETA_4_SIGNALING_PATHWAY	WP_ALPHA_6_BETA_4_SIGN	33
REACTOME_ANCHORING_FIBRIL_FORMATION	REACTOME_ANCHORING_FI	15
PID_FOXO_PATHWAY	PID_FOXO_PATHWAY	48
KEGG_PANCREATIC_CANCER	KEGG_PANCREATIC_CANCER	70
REACTOME_DISEASES_ASSOCIATED_WITH_GLYCOSAMINC	REACTOME_DISEASES_ASSO	41
KEGG_CHRONIC_MYELOID_LEUKEMIA	KEGG_CHRONIC_MYELOID_L	73

BIOCARTA_TGFB_PATHWAY	BIOCARTA_TGFB_PATHWAY	19
REACTOME_PARASITE_INFECTION	REACTOME_PARASITE_INFE(	116
WP_EXTRACELLULAR_VESICLEMEDIATED_SIGNALING_IN_I	WP_EXTRACELLULAR_VESICI	30
KEGG_PROSTATE_CANCER	KEGG_PROSTATE_CANCER	89
REACTOME_BINDING_AND_UPTAKE_OF_LIGANDS_BY_SC/	REACTOME_BINDING_AND_	98
REACTOME_RHOB_GTPASE_CYCLE	REACTOME_RHOB_GTPASE_	70
KEGG_GLYCINE_SERINE_AND_THREONINE_METABOLISM	KEGG_GLYCINE_SERINE_ANI	31
SIG_INSULIN_RECEPTOR_PATHWAY_IN_CARDIAC_MYOCY	SIG_INSULIN_RECEPTOR_PA	51
PID_TCPTP_PATHWAY	PID_TCPTP_PATHWAY	42
REACTOME_RHOD_GTPASE_CYCLE	REACTOME_RHOD_GTPASE_	53
REACTOME_DEFECTS_OF_CONTACT_ACTIVATION_SYSTEN	REACTOME_DEFECTS_OF_C(	15
BIOCARTA_HER2_PATHWAY	BIOCARTA_HER2_PATHWAY	23
WP_TGFBETA_SIGNALING_IN_THYROID_CELLS_FOR_EPITH	WP_TGFBETA_SIGNALING_II	17
PID_WNT_NONCANONICAL_PATHWAY	PID_WNT_NONCANONICAL_	32
WP_BREAST_CANCER_PATHWAY	WP_BREAST_CANCER_PATH	154
WP_TYPE_I_INTERFERON_INDUCTION_AND_SIGNALING_I	WP_TYPE_I_INTERFERON_IN	31
KEGG_RENAL_CELL_CARCINOMA	KEGG_RENAL_CELL_CARCIN(	70
KEGG_MELANOMA	KEGG_MELANOMA	71
REACTOME_INTERLEUKIN_4_AND_INTERLEUKIN_13_SIGN	REACTOME_INTERLEUKIN_4_	111
WP_MESODERMAL_COMMITMENT_PATHWAY	WP_MESODERMAL_COMMI	152
PID_ECADHERIN_NASCENT_AJ_PATHWAY	PID_ECADHERIN_NASCENT_/	39
WP_HEPATITIS_C_AND_HEPATOCELLULAR_CARCINOMA	WP_HEPATITIS_C_AND_HEP.	50
PID_GMCSF_PATHWAY	PID_GMCSF_PATHWAY	36
WP_GLIOBLASTOMA_SIGNALING_PATHWAYS	WP_GLIOBLASTOMA_SIGNA	82
REACTOME_SCAVENGING_BY_CLASS_A_RECEPTORS	REACTOME_SCAVENGING_B	19
PID_RET_PATHWAY	PID_RET_PATHWAY	39
PID_CXCR4_PATHWAY	PID_CXCR4_PATHWAY	100
WP_HOSTPATHOGEN_INTERACTION_OF_HUMAN_CORON	WP_HOSTPATHOGEN_INTER	33
REACTOME_DOWNSTREAM_SIGNAL_TRANSDUCTION	REACTOME_DOWNSTREAM_	29
REACTOME_TRANSCRIPTIONAL_ACTIVITY_OF_SMAD2_SM	REACTOME_TRANSCRIPTION	51
PID_BETA_CATENIN_NUC_PATHWAY	PID_BETA_CATENIN_NUC_P/	79
REACTOME_ROLE_OF_PHOSPHOLIPIDS_IN_PHAGOCYTOSI	REACTOME_ROLE_OF_PHOS	82
WP_RAC1PAK1P38MMP2_PATHWAY	WP_RAC1PAK1P38MMP2_P/	68
REACTOME_THE_CITRIC_ACID_TCA_CYCLE_AND_RESPIRA	REACTOME_THE_CITRIC_ACI	165
REACTOME_NEGATIVE_REGULATION_OF_THE_PI3K_AKT_	REACTOME_NEGATIVE_REGI	113
KEGG_SYSTEMIC_LUPUS_ERYTHEMATOSUS	KEGG_SYSTEMIC_LUPUS_ER	132
REACTOME_ANTIGEN_PRESENTATION_FOLDING_ASSEMB	REACTOME_ANTIGEN_PRESI	29
WP_PHOTODYNAMIC_THERAPYINDUCED_NFKB_SURVIVA	WP_PHOTODYNAMIC_THER/	35
REACTOME_INTERFERON_GAMMA_SIGNALING	REACTOME_INTERFERON_G/	91
WP_RETINOBLASTOMA_GENE_IN_CANCER	WP_RETINOBLASTOMA_GEN	87
WP_PHYSICOCHEMICAL_FEATURES_AND_TOXICITYASSOCI	WP_PHYSICOCHEMICAL_FEA	66
PID_AVB3_OPN_PATHWAY	PID_AVB3_OPN_PATHWAY	31
WP_LEPTIN_SIGNALING_PATHWAY	WP_LEPTIN_SIGNALING_PA	76
REACTOME_GLYCOSAMINOGLYCAN_METABOLISM	REACTOME_GLYCOSAMINO(	124
REACTOME_TOLL_LIKE_RECEPTOR_CASCADES	REACTOME_TOLL_LIKE_RECI	166
PID_TOLL_ENDOGENOUS_PATHWAY	PID_TOLL_ENDOGENOUS_P/	24
REACTOME_PI3K_AKT_SIGNALING_IN_CANCER	REACTOME_PI3K_AKT_SIGN.	105
WP_COMPLEMENT_SYSTEM_IN_NEURONAL_DEVELOPME	WP_COMPLEMENT_SYSTEM	105
WP_HEPATITIS_B_INFECTION	WP_HEPATITIS_B_INFECTIOI	150
PID_SYNDECAN_2_PATHWAY	PID_SYNDECAN_2_PATHWA	33

REACTOME_SIGNALING_BY_ALK_IN_CANCER	REACTOME_SIGNALING_BY_	55
WP_INFLAMMATORY_RESPONSE_PATHWAY	WP_INFLAMMATORY_RESPC	30
BIOCARTA_PTEN_PATHWAY	BIOCARTA_PTEN_PATHWAY	18
BIOCARTA_ALK_PATHWAY	BIOCARTA_ALK_PATHWAY	37
REACTOME_DISEASES_ASSOCIATED_WITH_O_GLYCOSYLA1	REACTOME_DISEASES_ASSO	68
REACTOME_O_LINKED_GLYCOSYLATION	REACTOME_O_LINKED_GLY(	111
WP_ADIPOGENESIS	WP_ADIPOGENESIS	131
PID_IL1_PATHWAY	PID_IL1_PATHWAY	34
KEGG_LEISHMANIA_INFECTION	KEGG_LEISHMANIA_INFECTI	70
REACTOME_SIGNALING_BY_VEGF	REACTOME_SIGNALING_BY_	106
REACTOME_FCGR_ACTIVATION	REACTOME_FCGR_ACTIVATI	69
BIOCARTA_G1_PATHWAY	BIOCARTA_G1_PATHWAY	28
WP_PDGF_PATHWAY	WP_PDGF_PATHWAY	39
PID_HNF3B_PATHWAY	PID_HNF3B_PATHWAY	44
WP_PI3KAKTMTOR_VITAMIN_D3_SIGNALING	WP_PI3KAKTMTOR_VITAMII	22
WP_PDGFRBETA_PATHWAY	WP_PDGFRBETA_PATHWAY	29
REACTOME_CONSTITUTIVE_SIGNALING_BY_ABERRANT_PI	REACTOME_CONSTITUTIVE_	78
REACTOME_ANTIMICROBIAL_PEPTIDES	REACTOME_ANTIMICROBIAL	84
KEGG_CELL_ADHESION_MOLECULES_CAMS	KEGG_CELL_ADHESION_MOI	130
REACTOME_ANTIVIRAL_MECHANISM_BY_IFN_STIMULATE	REACTOME_ANTIVIRAL_ME(	82
WP_PTDINS45P2_IN_CYTOKINESIS_PATHWAY	WP_PTDINS45P2_IN_CYTOK	12
PID_ECADHERIN_KERATINOCYTE_PATHWAY	PID_ECADHERIN_KERATINO(	21
WP_HEPATOCYTE_GROWTH_FACTOR_RECEPTOR_SIGNALI	WP_HEPATOCYTE_GROWTH	34
BIOCARTA_MET_PATHWAY	BIOCARTA_MET_PATHWAY	33
KEGG_APOPTOSIS	KEGG_APOPTOSIS	87
PID_PI3K_PLC_TRK_PATHWAY	PID_PI3K_PLC_TRK_PATHW/	36
REACTOME_MITOTIC_TELOPHASE_CYTOKINESIS	REACTOME_MITOTIC_TELOF	13
WP_NEURAL_CREST_CELL_MIGRATION_DURING_DEVELOI	WP_NEURAL_CREST_CELL_N	40
WP_NEURAL_CREST_CELL_MIGRATION_IN_CANCER	WP_NEURAL_CREST_CELL_N	43
WP_CLEAR_CELL_RENAL_CELL_CARCINOMA_PATHWAYS	WP_CLEAR_CELL_RENAL_CE	85
PID_VEGFR1_PATHWAY	PID_VEGFR1_PATHWAY	26
KEGG_NATURAL_KILLER_CELL_MEDIATED_CYTOTOXICITY	KEGG_NATURAL_KILLER_CEL	131
PID_AR_PATHWAY	PID_AR_PATHWAY	61
REACTOME_CREATION_OF_C4_AND_C2_ACTIVATORS	REACTOME_CREATION_OF_(	71
PID_FOXM1_PATHWAY	PID_FOXM1_PATHWAY	40
REACTOME_RND3_GTPASE_CYCLE	REACTOME_RND3_GTPASE_	42
BIOCARTA_FCER1_PATHWAY	BIOCARTA_FCER1_PATHWAY	39
WP_EMBRYONIC_STEM_CELL_PLURIPOTENCY_PATHWAYS	WP_EMBRYONIC_STEM_CEL	116
KEGG_PARKINSONS_DISEASE	KEGG_PARKINSONS_DISEASI	116
PID_TNF_PATHWAY	PID_TNF_PATHWAY	46
NABA_PROTEOGLYCANS	NABA_PROTEOGLYCANS	35
PID_ARF6_TRAFFICKING_PATHWAY	PID_ARF6_TRAFFICKING_PA1	49
WP_OXIDATIVE_PHOSPHORYLATION	WP_OXIDATIVE_PHOSPHOR'	52
PID_FGF_PATHWAY	PID_FGF_PATHWAY	55
PID_MYC_REPRESS_PATHWAY	PID_MYC_REPRESS_PATHW/	63
BIOCARTA_IL1R_PATHWAY	BIOCARTA_IL1R_PATHWAY	31
WP_IL10_ANTIINFLAMMATORY_SIGNALING_PATHWAY	WP_IL10_ANTIINFLAMMATC	12
REACTOME_RHOV_GTPASE_CYCLE	REACTOME_RHOV_GTPASE_	37
PID_NCADHERIN_PATHWAY	PID_NCADHERIN_PATHWAY	33
REACTOME_PROGRAMMED_CELL_DEATH	REACTOME_PROGRAMMED_	208

WP_GASTRIC_CANCER_NETWORK_1	WP_GASTRIC_CANCER_NET\	27
REACTOME_DAP12_INTERACTIONS	REACTOME_DAP12_INTERA(	40
PID_IL6_7_PATHWAY	PID_IL6_7_PATHWAY	47
BIOCARTA_ECM_PATHWAY	BIOCARTA_ECM_PATHWAY	19
REACTOME_G2_M_DNA_DAMAGE_CHECKPOINT	REACTOME_G2_M_DNA_DA	93
WP_TYROBP_CAUSAL_NETWORK_IN_MICROGLIA	WP_TYROBP_CAUSAL_NETW	60
REACTOME_FC_EPSILON_RECEPTOR_FCERI_SIGNALING	REACTOME_FC_EPSILON_RE	186
WP_NANOPARTICLEMEDIATED_ACTIVATION_OF_RECEPTC	WP_NANOPARTICLEMEDIAT	28
WP_WNTBETACATENIN_SIGNALING_PATHWAY_IN_LEUKE	WP_WNTBETACATENIN_SIGI	26
BIOCARTA_FAS_PATHWAY	BIOCARTA_FAS_PATHWAY	30
WP_CHROMOSOMAL_AND_MICROSATELLITE_INSTABILITY	WP_CHROMOSOMAL_AND_	73
REACTOME_POTASSIUM_CHANNELS	REACTOME_POTASSIUM_CH	102
REACTOME_FCGR3A_MEDIATED_IL10_SYNTHESIS	REACTOME_FCGR3A_MEDIA	95
KEGG_ARGININE_AND_PROLINE_METABOLISM	KEGG_ARGININE_AND_PROL	54
KEGG_COLORECTAL_CANCER	KEGG_COLORECTAL_CANCEI	62
WP_MAPK_SIGNALING_PATHWAY	WP_MAPK_SIGNALING_PATI	246
REACTOME_TRANSCRIPTIONAL_REGULATION_BY_RUNX2	REACTOME_TRANSCRIPTION	120
KEGG_HEDGEHOG_SIGNALING_PATHWAY	KEGG_HEDGEHOG_SIGNALIN	56
WP_AMPLIFICATION_AND_EXPANSION_OF_ONCOGENIC_	WP_AMPLIFICATION_AND_E	17
REACTOME_SUMOYLATION	REACTOME_SUMOYLATION	187
PID_ILK_PATHWAY	PID_ILK_PATHWAY	45
REACTOME_COMPLEX_I_BIOGENESIS	REACTOME_COMPLEX_I_BIC	51
REACTOME_FORMATION_OF_THE_CORNIFIED_ENVELOPE	REACTOME_FORMATION_OI	128
WP_ANDROGEN_RECEPTOR_SIGNALING_PATHWAY	WP_ANDROGEN_RECEPTOR_	90
WP_INTERLEUKIN11_SIGNALING_PATHWAY	WP_INTERLEUKIN11_SIGNAL	44
KEGG_CARDIAC_MUSCLE_CONTRACTION	KEGG_CARDIAC_MUSCLE_C(	74
REACTOME_UB_SPECIFIC_PROCESSING_PROTEASES	REACTOME_UB_SPECIFIC_PI	200
REACTOME_COHESIN_LOADING_ONTO_CHROMATIN	REACTOME_COHESIN_LOAD	10
REACTOME_SIGNALING_BY_HIPPO	REACTOME_SIGNALING_BY_	20
REACTOME_TOLL_LIKE_RECEPTOR_9_TLR9_CASCADE	REACTOME_TOLL_LIKE_RECI	106
WP_PHOTODYNAMIC_THERAPYINDUCED_AP1_SURVIVAL_	WP_PHOTODYNAMIC_THER/	51
PID_INTEGRIN_A4B1_PATHWAY	PID_INTEGRIN_A4B1_PATHV	33
KEGG_ARRHYTHMOGENIC_RIGHT_VENTRICULAR_CARDIO	KEGG_ARRHYTHMOGENIC_F	74
WP_4249_HEDGEHOG_SIGNALING_PATHWAY	WP_4249_HEDGEHOG_SIGN	43
WP_TRANSCRIPTION_COFACTORS_SKI_AND_SKIL_PROTEII	WP_TRANSCRIPTION_COFAC	18
WP_RELATIONSHIP_BETWEEN_INFLAMMATION_COX2_AN	WP_RELATIONSHIP_BETWEE	25
SIG_PIP3_SIGNALING_IN_CARDIAC_MYOCTES	SIG_PIP3_SIGNALING_IN_CA	67
REACTOME_CHONDROITIN_SULFATE_DERMATAN_SULFAT	REACTOME_CHONDROITIN_	50
REACTOME_SIGNALING_BY_EGFR	REACTOME_SIGNALING_BY_	50
BIOCARTA_IGF1_PATHWAY	BIOCARTA_IGF1_PATHWAY	21
PID_EPHB_FWD_PATHWAY	PID_EPHB_FWD_PATHWAY	40
REACTOME_APOPTOSIS	REACTOME_APOPTOSIS	179
WP_IL1_SIGNALING_PATHWAY	WP_IL1_SIGNALING_PATHW	55
KEGG_ENDOCYTOSIS	KEGG_ENDOCYTOSIS	181
REACTOME_COBALAMIN_CBL_VITAMIN_B12_TRANSPORT	REACTOME_COBALAMIN_CE	22
REACTOME_SEMA4D_IN_SEMAPHORIN_SIGNALING	REACTOME_SEMA4D_IN_SEI	24
KEGG_TOLL_LIKE_RECEPTOR_SIGNALING_PATHWAY	KEGG_TOLL_LIKE_RECEPTOR	101
REACTOME_RUNX2_REGULATES_BONE_DEVELOPMENT	REACTOME_RUNX2_REGUL/	31
REACTOME_CIRCADIAN_CLOCK	REACTOME_CIRCADIAN_CLC	70
REACTOME_RUNX2_REGULATES_OSTEOBLAST_DIFFERENT	REACTOME_RUNX2_REGUL/	24

BIOCARTA_VITCB_PATHWAY	BIOCARTA_VITCB_PATHWAY	11
REACTOME_INITIAL_TRIGGERING_OF_COMPLEMENT	REACTOME_INITIAL_TRIGGE	79
WP_EGFR_TYROSINE_KINASE_INHIBITOR_RESISTANCE	WP_EGFR_TYROSINE_KINASI	84
WP_MEASLES_VIRUS_INFECTION	WP_MEASLES_VIRUS_INFECT	135
WP_ARRHYTHMOGENIC_RIGHT_VENTRICULAR_CARDIOM	WP_ARRHYTHMOGENIC_RIG	75
REACTOME_SIGNALING_BY_FLT3_FUSION_PROTEINS	REACTOME_SIGNALING_BY_	19
PID_IL5_PATHWAY	PID_IL5_PATHWAY	14
REACTOME_ESTABLISHMENT_OF_SISTER_CHROMATID_CO	REACTOME_ESTABLISHMENT	11
REACTOME_INTRINSIC_PATHWAY_OF_FIBRIN_CLOT_FORI	REACTOME_INTRINSIC_PATH	22
KEGG_STARCH_AND_SUCROSE_METABOLISM	KEGG_STARCH_AND_SUCRO	51
REACTOME_MYD88_INDEPENDENT_TLR4_CASCADE	REACTOME_MYD88_INDEPE	108
WP_PANCREATIC_ADENOCARCINOMA_PATHWAY	WP_PANCREATIC_ADENOCA	89
WP_ACUTE_VIRAL_MYOCARDITIS	WP_ACUTE_VIRAL_MYOCAR	85
WP_TOLLLIKE_RECEPTOR_SIGNALING_RELATED_TO_MYD8	WP_TOLLLIKE_RECEPTOR_SI	31
WP_OSTEOBLAST_DIFFERENTIATION_AND_RELATED_DISE	WP_OSTEOBLAST_DIFFEREN	118
PID_ECADHERIN_STABILIZATION_PATHWAY	PID_ECADHERIN_STABILIZAT	41
WP_HEAD_AND_NECK_SQUAMOUS_CELL_CARCINOMA	WP_HEAD_AND_NECK_SQU/	74
WP_BONE_MORPHOGENIC_PROTEIN_SIGNALING_AND_R	WP_BONE_MORPHOGENIC_	12
BIOCARTA_IL10_PATHWAY	BIOCARTA_IL10_PATHWAY	13
REACTOME_RHO_GTPASES_ACTIVATE_ROCKS	REACTOME_RHO_GTPASES_	19
WP_H19_ACTION_RBE2F1_SIGNALING_AND_CDKBETACAT	WP_H19_ACTION_RBE2F1_S	15
BIOCARTA_NPP1_PATHWAY	BIOCARTA_NPP1_PATHWAY	10
REACTOME_KERATAN_SULFATE_DEGRADATION	REACTOME_KERATAN_SULF/	13
PID_AJDISS_2PATHWAY	PID_AJDISS_2PATHWAY	48
WP_SIGNAL_TRANSDUCTION_THROUGH_IL1R	WP_SIGNAL_TRANSDUCTIOI	33
KEGG_MATURITY_ONSET_DIABETES_OF_THE_YOUNG	KEGG_MATURITY_ONSET_DI	25
REACTOME_MET_ACTIVATES_RAP1_AND_RAC1	REACTOME_MET_ACTIVATES	11
BIOCARTA_PDGF_PATHWAY	BIOCARTA_PDGF_PATHWAY	28
PID_SYNDECAN_4_PATHWAY	PID_SYNDECAN_4_PATHWA	32
WP_MECHANOREGULATION_AND_PATHOLOGY_OF_YAPT	WP_MECHANOREGULATION	46
WP_NETRINUNC5B_SIGNALING_PATHWAY	WP_NETRINUNC5B_SIGNALI	52
WP_TOLLLIKE_RECEPTOR_SIGNALING_PATHWAY	WP_TOLLLIKE_RECEPTOR_SI	102
KEGG_CELL_CYCLE	KEGG_CELL_CYCLE	124
PID_ERBB1_RECEPTOR_PROXIMAL_PATHWAY	PID_ERBB1_RECEPTOR_PRO	35
WP_PROSTAGLANDIN_SYNTHESIS_AND_REGULATION	WP_PROSTAGLANDIN_SYNT	45
REACTOME_EARLY_SARS_COV_2_INFECTION_EVENTS	REACTOME_EARLY_SARS_CC	37
REACTOME_SYNTHESIS_OF_PIPS_AT_THE_PLASMA_MEM	REACTOME_SYNTHESIS_OF_	53
REACTOME_RHOG_GTPASE_CYCLE	REACTOME_RHOG_GTPASE_	74
WP_REGULATORY_CIRCUITS_OF_THE_STAT3_SIGNALING_	WP_REGULATORY_CIRCUITS	78
WP_HIPPO_SIGNALING_REGULATION_PATHWAYS	WP_HIPPO_SIGNALING_REG	97
WP_BIOMARKERS_FOR_UREA_CYCLE_DISORDERS	WP_BIOMARKERS_FOR_URE	12
WP_UREA_CYCLE_AND_METABOLISM_OF_AMINO_GROU	WP_UREA_CYCLE_AND_MET	21
WP_FACTORS_AND_PATHWAYS_AFFECTING_INSULINLIKE_	WP_FACTORS_AND_PATHW	36
WP_TYPE_III_INTERFERON_SIGNALING	WP_TYPE_III_INTERFERON_S	10
WP_GENES_CONTROLLING_NEPHROGENESIS	WP_GENES_CONTROLLING_	44
PID_ATF2_PATHWAY	PID_ATF2_PATHWAY	59
WP_PATHWAYS_AFFECTED_IN_ADENOID_CYSTIC_CARCIN	WP_PATHWAYS_AFFECTED_	65
KEGG_NOD_LIKE_RECEPTOR_SIGNALING_PATHWAY	KEGG_NOD_LIKE_RECEPTOR	62
REACTOME_OTHER_SEMAPHORIN_INTERACTIONS	REACTOME_OTHER_SEMAPH	19
PID_NETRIN_PATHWAY	PID_NETRIN_PATHWAY	32

REACTOME_NEURONAL_SYSTEM	REACTOME_NEURONAL_SYS	409
REACTOME_REMOVAL_OF_AMINOTERMINAL_PROPEPTID	REACTOME_REMOVAL_OF_/	10
KEGG_ACUTE_MYELOID_LEUKEMIA	KEGG_ACUTE_MYELOID_LEU	57
WP_IMMUNE_RESPONSE_TO_TUBERCULOSIS	WP_IMMUNE_RESPONSE_T(	23
PID_HIF1_TFPATHWAY	PID_HIF1_TFPATHWAY	66
KEGG_GRAFT_VERSUS_HOST_DISEASE	KEGG_GRAFT_VERSUS_HOST	37
WP_HAIR_FOLLICLE_DEVELOPMENT_ORGANOGENESIS_P/	WP_HAIR_FOLLICLE_DEVELC	32
REACTOME_APOPTOTIC_CLEAVAGE_OF_CELL_ADHESION_	REACTOME_APOPTOTIC_CLE	11
WP_AEROBIC_GLYCOLYSIS	WP_AEROBIC_GLYCOLYSIS	11
PID_TAP63_PATHWAY	PID_TAP63_PATHWAY	54
PID_NFAT_TFPATHWAY	PID_NFAT_TFPATHWAY	45
WP_LNCRNA_IN_CANONICAL_WNT_SIGNALING_AND_COI	WP_LNCRNA_IN_CANONICA	94
BIOCARTA_RAC1_PATHWAY	BIOCARTA_RAC1_PATHWAY	21
WP_INTEGRATED_CANCER_PATHWAY	WP_INTEGRATED_CANCER_I	44
REACTOME_GLYOXYLATE_METABOLISM_AND_GLYCINE_D	REACTOME_GLYOXYLATE_M	31
KEGG_LINOLEIC_ACID_METABOLISM	KEGG_LINOLEIC_ACID_MET/	29
BIOCARTA_PITX2_PATHWAY	BIOCARTA_PITX2_PATHWAY	16
WP_PHOTODYNAMIC_THERAPYINDUCED_HIF1_SURVIVAL	WP_PHOTODYNAMIC_THER/	38
WP_OVERLAP_BETWEEN_SIGNAL_TRANSDUCTION_PATH\	WP_OVERLAP_BETWEEN_SI(	56
PID_HEDGEHOG_2PATHWAY	PID_HEDGEHOG_2PATHWAY	22
REACTOME_RHOBTB_GTPASE_CYCLE	REACTOME_RHOBTB_GTPAS	35
WP_PLATELETMEDIATED_INTERACTIONS_WITH_VASCULA	WP_PLATELETMEDIATED_IN	17
REACTOME_DOWNREGULATION_OF_SMAD2_3_SMAD4_1	REACTOME_DOWNREGULAT	31
REACTOME_ATTACHMENT_AND_ENTRY	REACTOME_ATTACHMENT_/	19
REACTOME_GAMMA_CARBOXYLATION_TRANSPORT_AND	REACTOME_GAMMA_CARB(	11
REACTOME_ESTROGEN_DEPENDENT_NUCLEAR_EVENTS_I	REACTOME_ESTROGEN_DEP	24
REACTOME_GPVI_MEDIATED_ACTIVATION_CASCADE	REACTOME_GPVI_MEDIATEI	35
KEGG_HYPERTROPHIC_CARDIOMYOPATHY_HCM	KEGG_HYPERTROPHIC_CARD	83
REACTOME_MITOTIC_PROPHASE	REACTOME_MITOTIC_PROPI	140
PID_WNT_SIGNALING_PATHWAY	PID_WNT_SIGNALING_PATH	27
KEGG_ALZHEIMERS_DISEASE	KEGG_ALZHEIMERS_DISEASE	159
WP_RANKLRANK_SIGNALING_PATHWAY	WP_RANKLRANK_SIGNALIN(	55
REACTOME_REGULATION_OF_INSULIN_LIKE_GROWTH_F/	REACTOME_REGULATION_O	124
WP_ENDODERM_DIFFERENTIATION	WP_ENDODERM_DIFFERENT	143
REACTOME_APOPTOTIC_EXECUTION_PHASE	REACTOME_APOPTOTIC_EXE	52
REACTOME_INTERLEUKIN_6_FAMILY_SIGNALING	REACTOME_INTERLEUKIN_6_	24
REACTOME_CASPASE_ACTIVATION_VIA_EXTRINSIC_APOP	REACTOME_CASPASE_ACTIV	26
WP_CELL_CYCLE	WP_CELL_CYCLE	120
REACTOME_SIGNALING_BY_FGFR1	REACTOME_SIGNALING_BY_	50
REACTOME_TRANSCRIPTIONAL_REGULATION_BY_THE_AP	REACTOME_TRANSCRIPTION	38
WP_THYMIC_STROMAL_LYMPHOPOIETIN_TSLP_SIGNALIN	WP_THYMIC_STROMAL_LYM	47
KEGG_NEUROACTIVE_LIGAND_RECEPTOR_INTERACTION	KEGG_NEUROACTIVE_LIGAN	270
REACTOME_SYNTHESIS_OF_PIPS_AT_THE_EARLY_ENDOS(	REACTOME_SYNTHESIS_OF_	16
REACTOME_UPTAKE_OF_DIETARY_COBALAMINS_INTO_EI	REACTOME_UPTAKE_OF_DIE	10
REACTOME_GAB1_SIGNALOSOME	REACTOME_GAB1_SIGNALO	17
WP_DNA_IRDAMAGE_AND_CELLULAR_RESPONSE_VIA_AT	WP_DNA_IRDAMAGE_AND_	81
REACTOME_TRAFFICKING_AND_PROCESSING_OF_ENDOS(	REACTOME_TRAFFICKING_A	13
WP_METHIONINE_METABOLISM_LEADING_TO_SULFUR_A	WP_METHIONINE_METABOI	11
KEGG_LEUKOCYTE_TRANSENDOTHELIAL_MIGRATION	KEGG_LEUKOCYTE_TRANSEN	115
REACTOME_APOPTOTIC_CLEAVAGE_OF_CELLULAR_PROTI	REACTOME_APOPTOTIC_CLE	38

REACTOME_RHOU_GTPASE_CYCLE	REACTOME_RHOU_GTPASE_	40
WP_EXERCISEINDUCED_CIRCADIAN_REGULATION	WP_EXERCISEINDUCED_CIR(	48
REACTOME_SIGNALING_BY_EGFR_IN_CANCER	REACTOME_SIGNALING_BY_	25
REACTOME_CELL_EXTRACELLULAR_MATRIX_INTERACTION	REACTOME_CELL_EXTRACEL	18
REACTOME_SIGNALING_BY_FGFR1_IN_DISEASE	REACTOME_SIGNALING_BY_	38
WP_NEPHROTIC_SYNDROME	WP_NEPHROTIC_SYNDROMI	45
PID_ALK1_PATHWAY	PID_ALK1_PATHWAY	26
WP_MET_IN_TYPE_1_PAPILLARY_RENAL_CELL_CARCINOM	WP_MET_IN_TYPE_1_PAPILL	59
REACTOME_SIGNALING_BY_FLT3_ITD_AND_TKD_MUTANT	REACTOME_SIGNALING_BY_	16
WP_SPINAL_CORD_INJURY	WP_SPINAL_CORD_INJURY	117
REACTOME_REGULATION_OF_HSF1_MEDIATED_HEAT_SH	REACTOME_REGULATION_O	82
WP_MYD88_DISTINCT_INPUTOUTPUT_PATHWAY	WP_MYD88_DISTINCT_INPU	18
PID_RHOA_PATHWAY	PID_RHOA_PATHWAY	45
WP_WNT_SIGNALING	WP_WNT_SIGNALING	113
WP_TRANSSULFURATION_ONECARBON_METABOLISM_AN	WP_TRANSSULFURATION_O	66
WP_MFAP5MEDIATED_OVARIAN_CANCER_CELL_MOTILIT	WP_MFAP5MEDIATED_OVA	13
WP_SARSCOV2_INNATE_IMMUNITY_EVASION_AND_CELL	WP_SARSCOV2_INNATE_IMI	66
WP_NCRNAS_INVOLVED_IN_WNT_SIGNALING_IN_HEPAT(	WP_NCRNAS_INVOLVED_IN_	85
REACTOME_CARDIAC_CONDUCTION	REACTOME_CARDIAC_CONC	130
WP_ANGIOTENSIN_II_RECEPTOR_TYPE_1_PATHWAY	WP_ANGIOTENSIN_II_RECEP	28
WP_BARDETBIEDL_SYNDROME	WP_BARDETBIEDL_SYNDROI	84
REACTOME_OLFACTORY_SIGNALING_PATHWAY	REACTOME_OLFACTORY_SIG	333
REACTOME_TFAP2_AP_2_FAMILY_REGULATES_TRANSCRI	REACTOME_TFAP2_AP_2_FA	15
REACTOME_PROTEIN_UBIQUITINATION	REACTOME_PROTEIN_UBIQU	79
WP_TYPE_2_PAPILLARY_RENAL_CELL_CARCINOMA	WP_TYPE_2_PAPILLARY_REN	34
REACTOME_FORMATION_OF_THE_BETA_CATENIN_TCF_T	REACTOME_FORMATION_OI	89
WP_2586_ARYL_HYDROCARBON_RECEPTOR_PATHWAY	WP_2586_ARYL_HYDROCAR	46
KEGG_PRIMARY_IMMUNODEFICIENCY	KEGG_PRIMARY_IMMUNOD	35
REACTOME_CELLULAR_SENESCENCE	REACTOME_CELLULAR_SENE	193
REACTOME_SIGNALING_BY_CYTOSOLIC_FGFR1_FUSION_I	REACTOME_SIGNALING_BY_	18
REACTOME_SMAD2_SMAD3_SMAD4_HETEROTRIMER_RE	REACTOME_SMAD2_SMAD3	36
PID_PDGFRA_PATHWAY	PID_PDGFRA_PATHWAY	22
REACTOME_OVARIAN_TUMOR_DOMAIN_PROTEASES	REACTOME_OVARIAN_TUM(	39
WP_CELL_MIGRATION_AND_INVASION_THROUGH_P75NT	WP_CELL_MIGRATION_AND_	30
PID_INTEGRIN_A9B1_PATHWAY	PID_INTEGRIN_A9B1_PATHV	25
SIG_CHEMOTAXIS	SIG_CHEMOTAXIS	45
REACTOME_REGULATION_OF_BETA_CELL_DEVELOPMENT	REACTOME_REGULATION_O	42
REACTOME_RHO_GTPASES_ACTIVATE_PKNS	REACTOME_RHO_GTPASES_	91
PID_BMP_PATHWAY	PID_BMP_PATHWAY	42
BIOCARTA_INTEGRIN_PATHWAY	BIOCARTA_INTEGRIN_PATH\	34
WP_APOPTOSISRELATED_NETWORK_DUE_TO_ALTERED_I	WP_APOPTOSISRELATED_NE	53
REACTOME_L1CAM_INTERACTIONS	REACTOME_L1CAM_INTERA(	121
WP_OSTEOBLAST_SIGNALING	WP_OSTEOBLAST_SIGNALIN	14
BIOCARTA_ERK5_PATHWAY	BIOCARTA_ERK5_PATHWAY	14
WP_MITOCHONDRIAL_COMPLEX_I_ASSEMBLY_MODEL_O	WP_MITOCHONDRIAL_COM	50
PID_CMYB_PATHWAY	PID_CMYB_PATHWAY	84
BIOCARTA_RACCYCD_PATHWAY	BIOCARTA_RACCYCD_PATHV	26
REACTOME_SIGNALING_BY_NTRK3_TRKC	REACTOME_SIGNALING_BY_	17
SA_PTEN_PATHWAY	SA_PTEN_PATHWAY	17
WNT_SIGNALING	WNT_SIGNALING	88

REACTOME_SIGNALING_BY_KIT_IN_DISEASE	REACTOME_SIGNALING_BY_	20
KEGG_HUNTINGTONS_DISEASE	KEGG_HUNTINGTONS_DISEA	175
BIOCARTA_ATM_PATHWAY	BIOCARTA_ATM_PATHWAY	20
REACTOME_PROCESSING_OF_DNA_DOUBLE_STRAND_BRI	REACTOME_PROCESSING_OI	96
REACTOME_E3_UBIQUITIN_LIGASES_UBIQUITINATE_TARG	REACTOME_E3_UBIQUITIN_	59
REACTOME_SARS_COV_2_TARGETS_HOST_INTRACELLULA	REACTOME_SARS_COV_2_TA	12
REACTOME_CLEC7A_DECTIN_1_INDUCES_NFAT_ACTIVATI	REACTOME_CLEC7A_DECTIN	11
WP_22Q112_COPY_NUMBER_VARIATION_SYNDROME	WP_22Q112_COPY_NUMBE	125
PID_NFKAPPAB_CANONICAL_PATHWAY	PID_NFKAPPAB_CANONICAL	23
WP_2Q13_COPY_NUMBER_VARIATION_SYNDROME	WP_2Q13_COPY_NUMBER_	60
REACTOME_DNA_DAMAGE_TELOMERE_STRESS_INDUCED	REACTOME_DNA_DAMAGE_	79
WP_GDNFRET_SIGNALING_AXIS	WP_GDNFRET_SIGNALING_A	23
KEGG_GLYCOSAMINOGLYCAN_BIOSYNTHESIS_CHONDROI	KEGG_GLYCOSAMINOGLYCA	22
KEGG_BASAL_CELL_CARCINOMA	KEGG_BASAL_CELL_CARCINC	55
WP_SMC1SMC3_ROLE_IN_DNA_DAMAGE_CORNELIA_DE_	WP_SMC1SMC3_ROLE_IN_D	11
WP_ESTROGEN_SIGNALING_PATHWAY	WP_ESTROGEN_SIGNALING_	23
REACTOME_G_ALPHA_12_13_SIGNALLING_EVENTS	REACTOME_G_ALPHA_12_1	80
PID_AMB2_NEUTROPHILS_PATHWAY	PID_AMB2_NEUTROPHILS_P	41
REACTOME_NONHOMOLOGOUS_END_JOINING_NHEJ	REACTOME_NONHOMOLOG	67
WP_WNT_SIGNALING_PATHWAY_AND_PLURIPOTENCY	WP_WNT_SIGNALING_PATH	101
REACTOME_SEMA4D_INDUCED_CELL_MIGRATION_AND_(	REACTOME_SEMA4D_INDUC	20
WP_MICROTUBULE_CYTOSKELETON_REGULATION	WP_MICROTUBULE_CYTOSK	46
REACTOME_SIGNALING_BY_PDGFR_IN_DISEASE	REACTOME_SIGNALING_BY_	20
WP_OSTEOCLAST_SIGNALING	WP_OSTEOCLAST_SIGNALIN	16
REACTOME_ESTROGEN_DEPENDENT_GENE_EXPRESSION	REACTOME_ESTROGEN_DEP	147
BIOCARTA_GCR_PATHWAY	BIOCARTA_GCR_PATHWAY	17
REACTOME_NCAM_SIGNALING_FOR_NEURITE_OUT_GRO	REACTOME_NCAM_SIGNALI	63
REACTOME_TRANSMISSION_ACROSS_CHEMICAL_SYNAPSI	REACTOME_TRANSMISSION_	269
WP_LUNG_FIBROSIS	WP_LUNG_FIBROSIS	63
PID_PI3KCI_AKT_PATHWAY	PID_PI3KCI_AKT_PATHWAY	35
WP_NONGENOMIC_ACTIONS_OF_125_DIHYDROXYVITAM	WP_NONGENOMIC_ACTION	75
REACTOME_HCMV_EARLY_EVENTS	REACTOME_HCMV_EARLY_E	135
PID_INSULIN_PATHWAY	PID_INSULIN_PATHWAY	44
REACTOME_TRANSCRIPTIONAL_REGULATION_OF_GRANU	REACTOME_TRANSCRIPTION	87
REACTOME_DOWNSTREAM_SIGNALING_OF_ACTIVATED_I	REACTOME_DOWNSTREAM_	30
REACTOME_INTERFERON_ALPHA_BETA_SIGNALING	REACTOME_INTERFERON_AI	72
WP_MITOCHONDRIAL_COMPLEX_IV_ASSEMBLY	WP_MITOCHONDRIAL_COM	31
REACTOME_EPH_EPHRIN_SIGNALING	REACTOME_EPH_EPHRIN_SI	92
WP_PROLACTIN_SIGNALING_PATHWAY	WP_PROLACTIN_SIGNALING_	76
WP_ONECARBON_METABOLISM_AND_RELATED_PATHWA	WP_ONECARBON_METABOL	51
WP_PROSTAGLANDIN_SIGNALING	WP_PROSTAGLANDIN_SIGNA	33
REACTOME_INTERLEUKIN_2_FAMILY_SIGNALING	REACTOME_INTERLEUKIN_2_	44
WP_FAS_LIGAND_PATHWAY_AND_STRESS_INDUCTION_O	WP_FAS_LIGAND_PATHWAY	43
REACTOME_NUCLEOTIDE_BINDING_DOMAIN_LEUCINE_RI	REACTOME_NUCLEOTIDE_BI	56
WP_IL4_SIGNALING_PATHWAY	WP_IL4_SIGNALING_PATHW	54
WP_PLURIPOTENT_STEM_CELL_DIFFERENTIATION_PATHV	WP_PLURIPOTENT_STEM_CI	48
KEGG_JAK_STAT_SIGNALING_PATHWAY	KEGG_JAK_STAT_SIGNALING	154
WP_DNA_IRDOUBLE_STRAND_BREAKS_AND_CELLULAR_R	WP_DNA_IRDOUBLE_STRAN	55
KEGG_P53_SIGNALING_PATHWAY	KEGG_P53_SIGNALING_PATI	68
KEGG_NEUROTROPHIN_SIGNALING_PATHWAY	KEGG_NEUROTROPHIN_SIGN	126

WP_PI3KAKTMTOR_SIGNALING_PATHWAY_AND_THERAPI	WP_PI3KAKTMTOR_SIGNALI	30
WP_GLYCOLYSIS_IN_SENESCENCE	WP_GLYCOLYSIS_IN_SENESC	11
KEGG_VIRAL_MYOCARDITIS	KEGG_VIRAL_MYOCARDITIS	68
BIOCARTA_GSK3_PATHWAY	BIOCARTA_GSK3_PATHWAY	20
REACTOME_TRANSCRIPTIONAL_REGULATION_BY_RUNX3	REACTOME_TRANSCRIPTION	96
REACTOME_SARS_COV_2_ACTIVATES_MODULATES_INNA	REACTOME_SARS_COV_2_A	125
REACTOME_SIGNALING_BY_FGFR	REACTOME_SIGNALING_BY_	87
REACTOME_RND2_GTPASE_CYCLE	REACTOME_RND2_GTPASE_	43
BIOCARTA_RANKL_PATHWAY	BIOCARTA_RANKL_PATHWA	14
REACTOME_PASSIVE_TRANSPORT_BY_AQUAPORINS	REACTOME_PASSIVE_TRANS	13
REACTOME_SIGNAL_TRANSDUCTION_BY_L1	REACTOME_SIGNAL_TRANSI	21
WP_HYPERTROPHY_MODEL	WP_HYPERTROPHY_MODEL	20
REACTOME_REGULATION_OF_GENE_EXPRESSION_IN_BET	REACTOME_REGULATION_O	21
PID_TRKR_PATHWAY	PID_TRKR_PATHWAY	61
KEGG_ANTIGEN_PROCESSING_AND_PRESENTATION	KEGG_ANTIGEN_PROCESSIN	80
REACTOME_DEATH_RECEPTOR_SIGNALLING	REACTOME_DEATH_RECEPT	141
REACTOME_ION_CHANNEL_TRANSPORT	REACTOME_ION_CHANNEL_	183
WP_BMP_SIGNALING_IN_EYELID_DEVELOPMENT	WP_BMP_SIGNALING_IN_EY	20
KEGG_OLFACTORY_TRANSDUCTION	KEGG_OLFACTORY_TRANSD	320
REACTOME_EGR2_AND_SOX10_MEDIATED_INITIATION_C	REACTOME_EGR2_AND_SO	28
WP_IL3_SIGNALING_PATHWAY	WP_IL3_SIGNALING_PATHW	49
WP_FIBRIN_COMPLEMENT_RECEPTOR_3_SIGNALING_PA	WP_FIBRIN_COMPLEMENT_	43
REACTOME_POTENTIAL_THERAPEUTICS_FOR_SARS	REACTOME_POTENTIAL_THE	154
KEGG_ENDOMETRIAL_CANCER	KEGG_ENDOMETRIAL_CANC	52
REACTOME_CHONDROITIN_SULFATE_BIOSYNTHESIS	REACTOME_CHONDROITIN_	20
BIOCARTA_P53HYPOXIA_PATHWAY	BIOCARTA_P53HYPOXIA_PA	21
WP_PATHOGENIC_ESCHERICHIA_COLI_INFECTION	WP_PATHOGENIC_ESCHERIC	55
REACTOME_SIGNALING_BY_NTRK2_TRKB	REACTOME_SIGNALING_BY_	25
KEGG_NITROGEN_METABOLISM	KEGG_NITROGEN_METABOL	23
REACTOME_KERATAN_SULFATE_KERATIN_METABOLISM	REACTOME_KERATAN_SULF	34
REACTOME_FORMATION_OF_FIBRIN_CLOT_CLOTTING_CA	REACTOME_FORMATION_OI	38
KEGG_PROXIMAL_TUBULE_BICARBONATE_RECLAMATION	KEGG_PROXIMAL_TUBULE_I	23
WP_DOPAMINERGIC_NEUROGENESIS	WP_DOPAMINERGIC_NEUR	30
KEGG_DILATED_CARDIOMYOPATHY	KEGG_DILATED_CARDIOMY	90
PID_PLK1_PATHWAY	PID_PLK1_PATHWAY	46
REACTOME_SIGNALING_BY_PDGFRA_TRANSMEMBRANE_	REACTOME_SIGNALING_BY_	12
REACTOME_PROTEIN_LOCALIZATION	REACTOME_PROTEIN_LOCAI	161
REACTOME_SIGNALING_BY_SCF_KIT	REACTOME_SIGNALING_BY_	43
REACTOME_ONCOGENIC_MAPK_SIGNALING	REACTOME_ONCOGENIC_M	81
WP_CARDIAC_PROGENITOR_DIFFERENTIATION	WP_CARDIAC_PROGENITOR	53
WP_OSTEOPONTIN_SIGNALING	WP_OSTEOPONTIN_SIGNALI	13
REACTOME_RHO_GTPASES_ACTIVATE_FORMINS	REACTOME_RHO_GTPASES_	140
PID_IL2_1PATHWAY	PID_IL2_1PATHWAY	55
REACTOME_FCERI_MEDIATED_NF_KB_ACTIVATION	REACTOME_FCERI_MEDIATE	136
REACTOME_CASPASE_ACTIVATION_VIA_DEATH_RECEPTO	REACTOME_CASPASE_ACTIV	16
WP_OSX_AND_MIRNAS_IN_TOOTH_DEVELOPMENT	WP_OSX_AND_MIRNAS_IN_	26
REACTOME_INTERLEUKIN_RECEPTOR_SHC_SIGNALING	REACTOME_INTERLEUKIN_R	27
WP_LTF_DANGER_SIGNAL_RESPONSE_PATHWAY	WP_LTF_DANGER_SIGNAL_F	19
PID_IGF1_PATHWAY	PID_IGF1_PATHWAY	29
BIOCARTA_MAPK_PATHWAY	BIOCARTA_MAPK_PATHWAY	81

PID_NOTCH_PATHWAY	PID_NOTCH_PATHWAY	59
PID_REG_GR_PATHWAY	PID_REG_GR_PATHWAY	82
WP_MICROGLIA_PATHOGEN_PHAGOCYTOSIS_PATHWAY	WP_MICROGLIA_PATHOGEN	40
REACTOME_REGULATION_OF_LOCALIZATION_OF_FOXO_1	REACTOME_REGULATION_O	12
PID_E2F_PATHWAY	PID_E2F_PATHWAY	73
BIOCARTA_HIVNEF_PATHWAY	BIOCARTA_HIVNEF_PATHWA	56
REACTOME_MITOCHONDRIAL_TRANSLATION	REACTOME_MITOCHONDRIA	94
PID_P73PATHWAY	PID_P73PATHWAY	79
KEGG_GLYCEROLIPID_METABOLISM	KEGG_GLYCEROLIPID_META	49
REACTOME_EPHB_MEDIATED_FORWARD_SIGNALING	REACTOME_EPHB_MEDIATE	42
REACTOME_MET_ACTIVATES_RAS_SIGNALING	REACTOME_MET_ACTIVATES	11
KEGG_WNT_SIGNALING_PATHWAY	KEGG_WNT_SIGNALING_PAT	150
WP_NAD_METABOLISM_SIRTUINS_AND_AGING	WP_NAD_METABOLISM_SIR	11
BIOCARTA_EDG1_PATHWAY	BIOCARTA_EDG1_PATHWAY	22
REACTOME_POSTMITOTIC_NUCLEAR_PORE_COMPLEX_NI	REACTOME_POSTMITOTIC_N	27
PID_NFAT_3PATHWAY	PID_NFAT_3PATHWAY	53
WP_ATM_SIGNALING_IN_DEVELOPMENT_AND_DISEASE	WP_ATM_SIGNALING_IN_DE	45
REACTOME_SIGNALING_BY_NTRKS	REACTOME_SIGNALING_BY_	134
REACTOME_INTERLEUKIN_6_SIGNALING	REACTOME_INTERLEUKIN_6	11
REACTOME_SIGNALING_BY_FGFR3	REACTOME_SIGNALING_BY_	40
REACTOME_FLT3_SIGNALING_IN_DISEASE	REACTOME_FLT3_SIGNALING	28
REACTOME_SIGNALING_BY_TYPE_1_INSULIN_LIKE_GROW	REACTOME_SIGNALING_BY_	54
WP_HEMATOPOIETIC_STEM_CELL_GENE_REGULATION_BY	WP_HEMATOPOIETIC_STEM	21
BIOCARTA_P38MAPK_PATHWAY	BIOCARTA_P38MAPK_PATHW	36
PID_S1P_S1P3_PATHWAY	PID_S1P_S1P3_PATHWAY	29
REACTOME_IL_6_TYPE_CYTOKINE_RECEPTOR_LIGAND_IN	REACTOME_IL_6_TYPE_CYTO	17
REACTOME_MECP2_REGULATES_NEURONAL_RECEPTORS_	REACTOME_MECP2_REGULA	18
REACTOME_TIE2_SIGNALING	REACTOME_TIE2_SIGNALING	18
REACTOME_PHENYLALANINE_AND_TYROSINE_METABOLIS	REACTOME_PHENYLALANINE	11
REACTOME_INTEGRATION_OF_ENERGY_METABOLISM	REACTOME_INTEGRATION_O	108
WP_BLOOD_CLOTTING_CASCADE	WP_BLOOD_CLOTTING_CASC	22
REACTOME_SENESCENCE_ASSOCIATED_SECRETORY_PHEN	REACTOME_SENESCENCE_AS	109
REACTOME_REGULATION_OF_INSULIN_SECRETION	REACTOME_REGULATION_O	78
REACTOME_RHO_GTPASES_ACTIVATE_WASPS_AND_WAV	REACTOME_RHO_GTPASES_	36
BIOCARTA_INSULIN_PATHWAY	BIOCARTA_INSULIN_PATHW	21
REACTOME_DOWNSTREAM_SIGNALING_OF_ACTIVATED_I	REACTOME_DOWNSTREAM_	25
REACTOME_DOWNSTREAM_SIGNALING_OF_ACTIVATED_I	REACTOME_DOWNSTREAM_	31
BIOCARTA_BCELLSURVIVAL_PATHWAY	BIOCARTA_BCELLSURVIVAL_	15
REACTOME_DEFECTIVE_EXT2_CAUSES_EXOSTOSES_2	REACTOME_DEFECTIVE_EXT	14
REACTOME_SHC1_EVENTS_IN_EGFR_SIGNALING	REACTOME_SHC1_EVENTS_I	14
REACTOME_CELLULAR_RESPONSE_TO_HEAT_STRESS	REACTOME_CELLULAR_RESP	101
PID_KIT_PATHWAY	PID_KIT_PATHWAY	52
REACTOME_ATTENUATION_PHASE	REACTOME_ATTENUATION_	28
REACTOME_PLATELET_CALCIUM_HOMEOSTASIS	REACTOME_PLATELET_CALC	28
WP_INTERLEUKIN1_IL1_STRUCTURAL_PATHWAY	WP_INTERLEUKIN1_IL1_STRU	49
BIOCARTA_TPO_PATHWAY	BIOCARTA_TPO_PATHWAY	24
REACTOME_HDACS_DEACETYLATE_HISTONES	REACTOME_HDACS_DEACET	91
WP_OVERVIEW_OF_INTERFERONSMEDIATED_SIGNALING_	WP_OVERVIEW_OF_INTERFE	36
PID_IL3_PATHWAY	PID_IL3_PATHWAY	26
REACTOME_ERYTHROCYTES_TAKE_UP_CARBON_DIOXIDE_	REACTOME_ERYTHROCYTES_	13

REACTOME_RUNX3_REGULATES_P14_ARF	REACTOME_RUNX3_REGULA	10
REACTOME_SEMA3A_PLEXIN_REPULSION_SIGNALING_BY_	REACTOME_SEMA3A_PLEXIN	14
REACTOME_DEADENYLATION_OF_MRNA	REACTOME_DEADENYLATIO	25
PID_BARD1_PATHWAY	PID_BARD1_PATHWAY	29
PID_INTEGRIN2_PATHWAY	PID_INTEGRIN2_PATHWAY	28
WP_UREA_CYCLE_AND_ASSOCIATED_PATHWAYS	WP_UREA_CYCLE_AND_ASS	24
WP_PKCGAMMA_CALCIUM_SIGNALING_PATHWAY_IN_AT	WP_PKCGAMMA_CALCIUM_	22
REACTOME_SARS_COV_1_INFECTION	REACTOME_SARS_COV_1_IN	51
KEGG_ALANINE_ASPARTATE_AND_GLUTAMATE_METABO	KEGG_ALANINE_ASPARTATE	32
REACTOME_YAP1_AND_WWTR1_TAZ_STIMULATED_GEN	REACTOME_YAP1_AND_WW	15
WP_SYNAPTIC_VESICLE_PATHWAY	WP_SYNAPTIC_VESICLE_PAT	50
WP_CYSTEINE_AND_METHIONINE_CATABOLISM	WP_CYSTEINE_AND_METHIO	15
REACTOME_AFLATOXIN_ACTIVATION_AND_DETOXIFICATI	REACTOME_AFLATOXIN_ACT	19
BIOCARTA_LAIR_PATHWAY	BIOCARTA_LAIR_PATHWAY	17
WP_CELLS_AND_MOLECULES_INVOLVED_IN_LOCAL_ACU	WP_CELLS_AND_MOLECULE	17
REACTOME_FOXO_MEDIATED_TRANSCRIPTION	REACTOME_FOXO_MEDIATE	65
REACTOME_KERATAN_SULFATE_BIOSYNTHESIS	REACTOME_KERATAN_SULF	28
WP_IL6_SIGNALING_PATHWAY	WP_IL6_SIGNALING_PATHW	43
REACTOME_MUSCLE_CONTRACTION	REACTOME_MUSCLE_CONT	203
REACTOME_DEFENSINS	REACTOME_DEFENSINS	39
REACTOME_STAT3_NUCLEAR_EVENTS_DOWNSTREAM_O	REACTOME_STAT3_NUCLEA	10
BIOCARTA_IL7_PATHWAY	BIOCARTA_IL7_PATHWAY	16
WP_NOVEL_INTRACELLULAR_COMPONENTS_OF_RIGILIKE	WP_NOVEL_INTRACELLULAR	60
REACTOME_NCAM1_INTERACTIONS	REACTOME_NCAM1_INTERA	42
REACTOME_ION_HOMEOSTASIS	REACTOME_ION_HOMEOST	54
WP_P53_TRANSCRIPTIONAL_GENE_NETWORK	WP_P53_TRANSCRIPTIONAL	92
WP_NONALCOHOLIC_FATTY_LIVER_DISEASE	WP_NONALCOHOLIC_FATTY	148
REACTOME_RHO_GTPASES_ACTIVATE_CIT	REACTOME_RHO_GTPASES_	19
REACTOME_AQUAPORIN_MEDIATED_TRANSPORT	REACTOME_AQUAPORIN_M	52
BIOCARTA_LYM_PATHWAY	BIOCARTA_LYM_PATHWAY	14
KEGG_ALPHA_LINOLENIC_ACID_METABOLISM	KEGG_ALPHA_LINOLENIC_A	19
WP_PTF1A_RELATED_REGULATORY_PATHWAY	WP_PTF1A_RELATED_REGU	11
REACTOME_CONDENSATION_OF_PROPHASE_CHROMOSO	REACTOME_CONDENSATION	71
REACTOME_IRS_MEDIATED_SIGNALLING	REACTOME_IRS_MEDIATED_	48
REACTOME_TANDEM_PORE_DOMAIN_POTASSIUM_CHAN	REACTOME_TANDEM_PORE_	12
BIOCARTA_ERK_PATHWAY	BIOCARTA_ERK_PATHWAY	27
BIOCARTA_EGF_PATHWAY	BIOCARTA_EGF_PATHWAY	27
WP_SUDDEN_INFANT_DEATH_SYNDROME_SIDS_SUSCEPT	WP_SUDDEN_INFANT_DEAT	157
REACTOME_CONSTITUTIVE_SIGNALING_BY_OVEREXPRESS	REACTOME_CONSTITUTIVE_	11
PID_IFNG_PATHWAY	PID_IFNG_PATHWAY	40
WP_ENERGY_METABOLISM	WP_ENERGY_METABOLISM	48
REACTOME_MHC_CLASS_II_ANTIGEN_PRESENTATION	REACTOME_MHC_CLASS_II_	124
REACTOME_INSULIN_RECEPTOR_SIGNALLING_CASCADE	REACTOME_INSULIN_RECEP	54
REACTOME_BASIGIN_INTERACTIONS	REACTOME_BASIGIN_INTER	25
PID_CDC42_REG_PATHWAY	PID_CDC42_REG_PATHWAY	30
REACTOME_GOLGI_CISTERNAE_PERICENTRIOLAR_STACK_	REACTOME_GOLGI_CISTERN	14
PID_FCER1_PATHWAY	PID_FCER1_PATHWAY	60
REACTOME_RUNX1_REGULATES_GENES_INVOLVED_IN_M	REACTOME_RUNX1_REGULA	95
WP_IL1_AND_MEGAKARYOCYTES_IN_OBESITY	WP_IL1_AND_MEGAKARYOC	24
REACTOME_RHOBTB1_GTPASE_CYCLE	REACTOME_RHOBTB1_GTPA	23

REACTOME_RESOLUTION_OF_SISTER_CHROMATID_COHE	REACTOME_RESOLUTION_O	126
BIOCARTA_HCMV_PATHWAY	BIOCARTA_HCMV_PATHWAY	17
WP_TLR4_SIGNALING_AND_TOLERANCE	WP_TLR4_SIGNALING_AND_	28
WP_TCELL_RECEPTOR_SIGNALING_PATHWAY	WP_TCELL_RECEPTOR_SIGN	90
KEGG_BLADDER_CANCER	KEGG_BLADDER_CANCER	42
REACTOME_MAPK_TARGETS_NUCLEAR_EVENTS_MEDIATI	REACTOME_MAPK_TARGETS	31
REACTOME_SIGNALING_BY_ERBB2_ECD_MUTANTS	REACTOME_SIGNALING_BY_	16
WP_MRNA_VACCINE_ACTIVATION_OF_DENDRITIC_CELL_	WP_MRNA_VACCINE_ACTIV	10
REACTOME_PI_METABOLISM	REACTOME_PI_METABOLISM	84
REACTOME_RHO_GTPASES_ACTIVATE_PAKS	REACTOME_RHO_GTPASES_	21
WP_ENVELOPE_PROTEINS_AND_THEIR_POTENTIAL_ROLE	WP_ENVELOPE_PROTEINS_	45
BIOCARTA_VDR_PATHWAY	BIOCARTA_VDR_PATHWAY	24
REACTOME_CLATHRIN_MEDIATED_ENDOCYTOSIS	REACTOME_CLATHRIN_MED	145
REACTOME_SIGNALING_BY_FGFR2_IN_DISEASE	REACTOME_SIGNALING_BY_	43
WP_COMMON_PATHWAYS_UNDERLYING_DRUG_ADDICT	WP_COMMON_PATHWAYS_	41
REACTOME_DEGRADATION_OF_CYSTEINE_AND_HOMOCY	REACTOME_DEGRADATION_	14
PID_ERBB2_ERBB3_PATHWAY	PID_ERBB2_ERBB3_PATHW	44
REACTOME_SCAVENGING_OF_HEME_FROM_PLASMA	REACTOME_SCAVENGING_O	69
REACTOME_SIGNALING_BY_FGFR2	REACTOME_SIGNALING_BY_	73
REACTOME_INTERLEUKIN_3_INTERLEUKIN_5_AND_GM_C	REACTOME_INTERLEUKIN_3	48
WP_HIPPOYAP_SIGNALING_PATHWAY	WP_HIPPOYAP_SIGNALING_	22
WP_LIPID_PARTICLES_COMPOSITION	WP_LIPID_PARTICLES_COMP	10
REACTOME_SENSORY_PROCESSING_OF_SOUND	REACTOME_SENSORY_PROC	77
REACTOME_ASPIRIN_ADME	REACTOME_ASPIRIN_ADME	44
KEGG_CALCIUM_SIGNALING_PATHWAY	KEGG_CALCIUM_SIGNALING	178
WP_PROTEOGLYCAN_BIOSYNTHESIS	WP_PROTEOGLYCAN_BIOSY	18
WP_VITAMIN_D_IN_INFLAMMATORY_DISEASES	WP_VITAMIN_D_IN_INFLAM	22
PID_CIRCADIAN_PATHWAY	PID_CIRCADIAN_PATHWAY	16
WP_IL5_SIGNALING_PATHWAY	WP_IL5_SIGNALING_PATHW	40
PID_TELOMERASE_PATHWAY	PID_TELOMERASE_PATHWA	67
KEGG_LONG_TERM_POTENTIATION	KEGG_LONG_TERM_POTENT	70
REACTOME_SULFUR_AMINO_ACID_METABOLISM	REACTOME_SULFUR_AMINO	27
WP_BRAINDERIVED_NEUROTROPHIC_FACTOR_BDNF_SIGI	WP_BRAINDERIVED_NEURO	144
REACTOME_PI_3K_CASCADE_FGFR2	REACTOME_PI_3K_CASCADE	23
REACTOME_INTRINSIC_PATHWAY_FOR_APOPTOSIS	REACTOME_INTRINSIC_PATH	55
REACTOME_SYNTHESIS_OF_PIPS_AT_THE_LATE_ENDOSO	REACTOME_SYNTHESIS_OF_	11
PID_HIF2PATHWAY	PID_HIF2PATHWAY	34
WP_METAPATHWAY_BIOTRANSFORMATION_PHASE_I_AN	WP_METAPATHWAY_BIOTR	183
BIOCARTA_GLEEVEC_PATHWAY	BIOCARTA_GLEEVEC_PATHW	23
REACTOME_RECEPTOR_TYPE_TYROSINE_PROTEIN_PHOSP	REACTOME_RECEPTOR_TYP	20
REACTOME_SIGNALING_BY_FGFR_IN_DISEASE	REACTOME_SIGNALING_BY_	63
REACTOME_POST_TRANSLATIONAL_MODIFICATION_SYNT	REACTOME_POST_TRANSLA	92
WP_ALLOGRAFT_REJECTION	WP_ALLOGRAFT_REJECTION	89
WP_PROSTAGLANDIN_AND_LEUKOTRIENE_METABOLISM_	WP_PROSTAGLANDIN_AND_	31
REACTOME_VEGFR2_MEDIATED_CELL_PROLIFERATION	REACTOME_VEGFR2_MEDIA	19
REACTOME_CARGO_RECOGNITION_FOR_CLATHRIN_MEDI	REACTOME_CARGO_RECOGI	105
WP_INTERLEUKIN1_INDUCED_ACTIVATION_OF_NFKB	WP_INTERLEUKIN1_INDUCE	10
WP_FOXA2_PATHWAY	WP_FOXA2_PATHWAY	21
PID_P75_NTR_PATHWAY	PID_P75_NTR_PATHWAY	68
REACTOME_SIGNALING_BY_LEPTIN	REACTOME_SIGNALING_BY_	11

PID_RAC1_REG_PATHWAY	PID_RAC1_REG_PATHWAY	38
REACTOME_UREA_CYCLE	REACTOME_UREA_CYCLE	10
REACTOME_HATS_ACETYLATE_HISTONES	REACTOME_HATS_ACETYLAT	139
REACTOME_XENOBIOTICS	REACTOME_XENOBIOTICS	23
BIOCARTA_ARAP_PATHWAY	BIOCARTA_ARAP_PATHWAY	17
PID_PTP1B_PATHWAY	PID_PTP1B_PATHWAY	52
REACTOME_TRAFFICKING_OF_AMPA_RECEPTORS	REACTOME_TRAFFICKING_O	31
REACTOME_NRAGE_SIGNALS_DEATH_THROUGH_JNK	REACTOME_NRAGE_SIGNALS	59
BIOCARTA_AGR_PATHWAY	BIOCARTA_AGR_PATHWAY	33
REACTOME_DNA_DOUBLE_STRAND_BREAK_RESPONSE	REACTOME_DNA_DOUBLE_S	77
REACTOME_PHASE_II_CONJUGATION_OF_COMPOUNDS	REACTOME_PHASE_II_CONJU	108
REACTOME_DEPOSITION_OF_NEW_CENPA_CONTAINING_	REACTOME_DEPOSITION_OF	72
KEGG_UBIQUITIN_MEDIATED_PROTEOLYSIS	KEGG_UBIQUITIN_MEDIATEI	134
REACTOME_GABA_SYNTHESIS_RELEASE_REUPTAKE_AND_	REACTOME_GABA_SYNTHES	19
REACTOME_SIGNALING_BY_WNT_IN_CANCER	REACTOME_SIGNALING_BY_	33
REACTOME_DOWNSTREAM_SIGNALING_OF_ACTIVATED_I	REACTOME_DOWNSTREAM_	27
REACTOME_RESPONSE_TO_METAL_IONS	REACTOME_RESPONSE_TO_	14
REACTOME_NETRIN_1_SIGNALING	REACTOME_NETRIN_1_SIGN	50
WP_SEROTONIN_RECEPTOR_467_AND_NR3C_SIGNALING	WP_SEROTONIN_RECEPTOR_	19
REACTOME_SIGNALING_BY_ERBB2_IN_CANCER	REACTOME_SIGNALING_BY_	26
REACTOME_RAB_GEFS_EXCHANGE_GTP_FOR_GDP_ON_R	REACTOME_RAB_GEFS_EXCH	90
WP_FGF23_SIGNALING_IN_HYPOPHOSPHATEMIC_RICKETS	WP_FGF23_SIGNALING_IN_I	23
REACTOME_MITOCHONDRIAL_PROTEIN_IMPORT	REACTOME_MITOCHONDRIA	62
WP_FOLATE_METABOLISM	WP_FOLATE_METABOLISM	69
REACTOME_CYP2E1_REACTIONS	REACTOME_CYP2E1_REACTI	11
REACTOME_FRS_MEDIATED_FGFR2_SIGNALING	REACTOME_FRS_MEDIATED_	25
REACTOME_STAT5_ACTIVATION_DOWNSTREAM_OF_FLT3	REACTOME_STAT5_ACTIVAT	10
PID_TCR_CALCIUM_PATHWAY	PID_TCR_CALCIUM_PATHWA	28
REACTOME_CHYLOMICRON_REMODELING	REACTOME_CHYLOMICRON_	10
WP_VITAMIN_K_METABOLISM_AND_ACTIVATION_OF_DE	WP_VITAMIN_K_METABOLIS	12
REACTOME_REDUCTION_OF_CYTOSOLIC_CA_LEVELS	REACTOME_REDUCTION_OF	12
REACTOME_ENDOSOMAL_VACUOLAR_PATHWAY	REACTOME_ENDOSOMAL_V	11
REACTOME_CHK1_CHK2_CDS1_MEDIATED_INACTIVATION	REACTOME_CHK1_CHK2_CD	13
PID_EPHA2_FWD_PATHWAY	PID_EPHA2_FWD_PATHWAY	19
WP_MIRNAS_INVOLVEMENT_IN_THE_IMMUNE_RESPONS	WP_MIRNAS_INVOLVEMENT	53
BIOCARTA_MONOCYTE_PATHWAY	BIOCARTA_MONOCYTE_PAT	11
PID_ERBB1_INTERNALIZATION_PATHWAY	PID_ERBB1_INTERNALIZATIC	41
REACTOME_MITOTIC_PROMETAPHASE	REACTOME_MITOTIC_PROM	203
REACTOME_CARGO_CONCENTRATION_IN_THE_ER	REACTOME_CARGO_CONCEI	33
BIOCARTA_UCALPAIN_PATHWAY	BIOCARTA_UCALPAIN_PATH	14
WP_INTERACTIONS_OF_NATURAL_KILLER_CELLS_IN_PAN(	WP_INTERACTIONS_OF_NAT	28
REACTOME_REGULATION_OF_LIPID_METABOLISM_BY_PF	REACTOME_REGULATION_O	118
KEGG_PATHOGENIC_ESCHERICHIA_COLI_INFECTION	KEGG_PATHOGENIC_ESCHEF	56
WP_CORTICOTROPINRELEASING_HORMONE_SIGNALING_	WP_CORTICOTROPINRELEAS	92
WP_GLYOXYLATE_METABOLISM	WP_GLYOXYLATE_METABOL	11
BIOCARTA_NTHI_PATHWAY	BIOCARTA_NTHI_PATHWAY	23
WP_MICRORNAS_IN_CARDIOMYOCYTE_HYPERTROPHY	WP_MICRORNAS_IN_CARDI(	94
REACTOME_SEMA3A_PAK_DEPENDENT_AXON_REPULSIOI	REACTOME_SEMA3A_PAK_I	16
REACTOME_MITOCHONDRIAL_FATTY_ACID_BETA_OXIDAT	REACTOME_MITOCHONDRIA	11
REACTOME_ACTIVATED_PKN1_STIMULATES_TRANSCRIPTI	REACTOME_ACTIVATED_PKI	64

REACTOME_FGFR1_MUTANT_RECEPTOR_ACTIVATION	REACTOME_FGFR1_MUTANT	31
WP_AIRWAY_SMOOTH_MUSCLE_CELL_CONTRACTION	WP_AIRWAY_SMOOTH_MUS	16
REACTOME_MEIOTIC_RECOMBINATION	REACTOME_MEIOTIC_RECOI	84
PID_TRAIL_PATHWAY	PID_TRAIL_PATHWAY	28
KEGG_TIGHT_JUNCTION	KEGG_TIGHT_JUNCTION	130
PID_S1P_S1P1_PATHWAY	PID_S1P_S1P1_PATHWAY	21
REACTOME_PHASE_0_RAPID_DEPOLARISATION	REACTOME_PHASE_0_RAPID	32
REACTOME_CONSTITUTIVE_SIGNALING_BY_EGFRVIII	REACTOME_CONSTITUTIVE_	15
KEGG_DRUG_METABOLISM_CYTOCHROME_P450	KEGG_DRUG_METABOLISM_	71
REACTOME_CREATINE_METABOLISM	REACTOME_CREATINE_MET/	11
REACTOME_FLT3_SIGNALING	REACTOME_FLT3_SIGNALIN(	38
PID_MYC_ACTIV_PATHWAY	PID_MYC_ACTIV_PATHWAY	79
REACTOME_HSF1_ACTIVATION	REACTOME_HSF1_ACTIVATI(	31
WP_FOXP3_IN_COVID19	WP_FOXP3_IN_COVID19	15
REACTOME_TRANSCRIPTIONAL_REGULATION_BY_E2F6	REACTOME_TRANSCRIPTION	34
REACTOME_STIMULI_SENSING_CHANNELS	REACTOME_STIMULI_SENSIN	106
BIOCARTA_TID_PATHWAY	BIOCARTA_TID_PATHWAY	19
REACTOME_SIGNALING_BY_ERBB2	REACTOME_SIGNALING_BY_	49
REACTOME_TP53_REGULATES_TRANSCRIPTION_OF_CELL_	REACTOME_TP53_REGULATI	49
REACTOME_METABOLISM_OF_STEROID_HORMONES	REACTOME_METABOLISM_(	35
WP_NEURAL_CREST_DIFFERENTIATION	WP_NEURAL_CREST_DIFFER	100
WP_BLADDER_CANCER	WP_BLADDER_CANCER	40
REACTOME_HCMV_LATE_EVENTS	REACTOME_HCMV_LATE_EV	113
BIOCARTA_NFKB_PATHWAY	BIOCARTA_NFKB_PATHWAY	21
PID_AURORA_B_PATHWAY	PID_AURORA_B_PATHWAY	39
REACTOME_SIGNALING_BY_CTNNB1_PHOSPHO_SITE_MU	REACTOME_SIGNALING_BY_	15
REACTOME_FGFR2_LIGAND_BINDING_AND_ACTIVATION	REACTOME_FGFR2_LIGAND_	20
WP_IMATINIB_AND_CHRONIC_MYELOID_LEUKEMIA	WP_IMATINIB_AND_CHRON	20
WP_OVERVIEW_OF_PROINFLAMMATORY_AND_PROFIBR(	WP_OVERVIEW_OF_PROINF	126
REACTOME_GPCR_LIGAND_BINDING	REACTOME_GPCR_LIGAND_	459
REACTOME_MATURATION_OF_NUCLEOPROTEIN	REACTOME_MATURATION_(	15
REACTOME_ERK_MAPK_TARGETS	REACTOME_ERK_MAPK_TAF	22
REACTOME_TOLL_LIKE_RECEPTOR_TLR1_TLR2_CASCADE	REACTOME_TOLL_LIKE_RECI	115
BIOCARTA_IL2RB_PATHWAY	BIOCARTA_IL2RB_PATHWAY	37
WP_INTERACTOME_OF_POLYCOMB_REPRESSIVE_COMPLI	WP_INTERACTOME_OF_POL	16
REACTOME_SIGNALING_BY_BRAF_AND_RAF1_FUSIONS	REACTOME_SIGNALING_BY_	65
BIOCARTA_ARF_PATHWAY	BIOCARTA_ARF_PATHWAY	17
REACTOME_LONG_TERM_POTENTIATION	REACTOME_LONG_TERM_P(	23
BIOCARTA_RAS_PATHWAY	BIOCARTA_RAS_PATHWAY	22
REACTOME_DISEASES_OF_PROGRAMMED_CELL_DEATH	REACTOME_DISEASES_OF_P	100
BIOCARTA_PLATELETAPP_PATHWAY	BIOCARTA_PLATELETAPP_PA	14
REACTOME_GASTRIN_CREB_SIGNALLING_PATHWAY_VIA_	REACTOME_GASTRIN_CREB_	18
REACTOME_RORA_ACTIVATES_GENE_EXPRESSION	REACTOME_RORA_ACTIVATI	18
WP_INTERACTIONS_BETWEEN_IMMUNE_CELLS_AND_MI(	WP_INTERACTIONS_BETWEE	39
REACTOME_SUMOYLATION_OF_DNA_DAMAGE_RESPONS	REACTOME_SUMOYLATION_	77
REACTOME_INTERLEUKIN_20_FAMILY_SIGNALING	REACTOME_INTERLEUKIN_2(	26
REACTOME_PURINERGIC_SIGNALING_IN_LEISHMANIASIS_	REACTOME_PURINERGIC_SI(	26
REACTOME_TCF_DEPENDENT_SIGNALING_IN_RESPONSE_	REACTOME_TCF_DEPENDEN	230
REACTOME_PI_3K_CASCADE_FGFR3	REACTOME_PI_3K_CASCADE	18
BIOCARTA_INFLAM_PATHWAY	BIOCARTA_INFLAM_PATHW/	27

REACTOME_NOD1_2_SIGNALING_PATHWAY	REACTOME_NOD1_2_SIGNA	36
REACTOME_EPIGENETIC_REGULATION_OF_GENE_EXPRES	REACTOME_EPIGENETIC_RE	142
REACTOME_NEUROTRANSMITTER_RELEASE_CYCLE	REACTOME_NEUROTRANSM	51
KEGG_COMPLEMENT_AND_COAGULATION_CASCADES	KEGG_COMPLEMENT_AND_	69
KEGG_T_CELL_RECEPTOR_SIGNALING_PATHWAY	KEGG_T_CELL_RECEPTOR_SI	108
REACTOME_SIGNALING_BY_FGFR4_IN_DISEASE	REACTOME_SIGNALING_BY_	11
PID_ER_NONGENOMIC_PATHWAY	PID_ER_NONGENOMIC_PAT	40
PID_RXR_VDR_PATHWAY	PID_RXR_VDR_PATHWAY	26
WP_PYRIMIDINE_METABOLISM_AND_RELATED_DISEASES	WP_PYRIMIDINE_METABOLI	17
REACTOME_PRE_NOTCH_EXPRESSION_AND_PROCESSING	REACTOME_PRE_NOTCH_EX	113
BIOCARTA_CASPASE_PATHWAY	BIOCARTA_CASPASE_PATHW	22
REACTOME_HCMV_INFECTION	REACTOME_HCMV_INFECTI	159
WP_KIT_RECEPTOR_SIGNALING_PATHWAY	WP_KIT_RECEPTOR_SIGNALI	59
REACTOME_PHOSPHOLIPASE_C_MEDIATED_CASCADE_FG	REACTOME_PHOSPHOLIPASI	18
BIOCARTA_CALCINEURIN_PATHWAY	BIOCARTA_CALCINEURIN_PA	18
BIOCARTA_P53_PATHWAY	BIOCARTA_P53_PATHWAY	16
REACTOME_HDR_THROUGH_SINGLE_STRAND_ANNEALIN	REACTOME_HDR_THROUGH	37
REACTOME_PHASE_2_PLATEAU_PHASE	REACTOME_PHASE_2_PLATE	15
PID_TCR_PATHWAY	PID_TCR_PATHWAY	64
PID_ERBB4_PATHWAY	PID_ERBB4_PATHWAY	38
WP_ANGIOPOIETINLIKE_PROTEIN_8_REGULATORY_PATH	WP_ANGIOPOIETINLIKE_PRO	131
WP_THYROID_STIMULATING_HORMONE_TSH_SIGNALING	WP_THYROID_STIMULATING	66
REACTOME_INTERLEUKIN_27_SIGNALING	REACTOME_INTERLEUKIN_2	11
REACTOME_NEGATIVE_REGULATION_OF_FGFR2_SIGNALI	REACTOME_NEGATIVE_REGU	34
REACTOME_ELEVATION_OF_CYTOSOLIC_CA2_LEVELS	REACTOME_ELEVATION_OF_	16
WP_GLYCEROLIPIDS_AND_GLYCEROPHOSPHOLIPIDS	WP_GLYCEROLIPIDS_AND_G	22
REACTOME_METALLOTHIONEINS_BIND_METALS	REACTOME_METALLOTHION	11
WP_P38_MAPK_SIGNALING_PATHWAY	WP_P38_MAPK_SIGNALING_	34
WP_MELANOMA	WP_MELANOMA	68
WP_ALANINE_AND_ASPARTATE_METABOLISM	WP_ALANINE_AND_ASPART	12
REACTOME_INTERLEUKIN_17_SIGNALING	REACTOME_INTERLEUKIN_1	71
REACTOME_SIGNALING_BY_FGFR4	REACTOME_SIGNALING_BY_	41
BIOCARTA_IL6_PATHWAY	BIOCARTA_IL6_PATHWAY	21
REACTOME_HDL_REMODELING	REACTOME_HDL_REMODELI	10
REACTOME_NEUROTRANSMITTER_RECEPTORS_AND_POS	REACTOME_NEUROTRANSM	205
REACTOME_DNA_METHYLATION	REACTOME_DNA_METHYLA	62
REACTOME_EICOSANOIDS	REACTOME_EICOSANOIDS	12
KEGG_CIRCADIAN_RHYTHM_MAMMAL	KEGG_CIRCADIAN_RHYTHM_	13
REACTOME_ABACAVIR_ADME	REACTOME_ABACAVIR_ADM	10
REACTOME_TRANSPORT_OF_ORGANIC_ANIONS	REACTOME_TRANSPORT_OF	11
REACTOME_INTERLEUKIN_1_FAMILY_SIGNALING	REACTOME_INTERLEUKIN_1	152
REACTOME_ASSEMBLY_OF_THE_ORC_COMPLEX_AT_THE_	REACTOME_ASSEMBLY_OF_	66
KEGG_CHEMOKINE_SIGNALING_PATHWAY	KEGG_CHEMOKINE_SIGNALI	188
REACTOME_BIOLOGICAL_OXIDATIONS	REACTOME_BIOLOGICAL_OX	219
REACTOME_N_GLYCAN_ANTENNAE_ELONGATION	REACTOME_N_GLYCAN_ANT	15
REACTOME_PEPTIDE_LIGAND_BINDING_RECEPTORS	REACTOME_PEPTIDE_LIGAN	195
KEGG_ARACHIDONIC_ACID_METABOLISM	KEGG_ARACHIDONIC_ACID_	58
WP_COMPLEMENT_AND_COAGULATION_CASCADES	WP_COMPLEMENT_AND_CC	58
REACTOME_TRANSCRIPTIONAL_REGULATION_BY_RUNX1	REACTOME_TRANSCRIPTION	236
REACTOME_CA2_PATHWAY	REACTOME_CA2_PATHWAY	62

REACTOME_GRB2_SOS_PROVIDES_LINKAGE_TO_MAPK_S	REACTOME_GRB2_SOS_PRO	15
PID_RB_1PATHWAY	PID_RB_1PATHWAY	65
REACTOME_ERCC6_CSB_AND_EHMT2_G9A_POSITIVELY_F	REACTOME_ERCC6_CSB_ANI	73
WP_COHESIN_COMPLEX_CORNELIA_DE_LANGE_SYNDROI	WP_COHESIN_COMPLEX_CC	34
SIG_IL4RECEPTOR_IN_B_LYPHOCYTES	SIG_IL4RECEPTOR_IN_B_LYP	27
WP_WNT_SIGNALING_PATHWAY	WP_WNT_SIGNALING_PATH	51
BIOCARTA_EIF4_PATHWAY	BIOCARTA_EIF4_PATHWAY	24
PID_PS1_PATHWAY	PID_PS1_PATHWAY	46
WP_INFLUENCE_OF_LAMINOPATHIES_ON_WNT_SIGNALII	WP_INFLUENCE_OF_LAMINC	36
REACTOME_CLASS_A_1_RHODOPSIN_LIKE_RECEPTORS	REACTOME_CLASS_A_1_RHC	327
WP_STATIN_INHIBITION_OF_CHOLESTEROL_PRODUCTION	WP_STATIN_INHIBITION_OF_	31
KEGG_BASAL_TRANSCRIPTION_FACTORS	KEGG_BASAL_TRANSCRIPTIC	35
REACTOME_DISSOLUTION_OF_FIBRIN_CLOT	REACTOME_DISSOLUTION_C	13
KEGG_METABOLISM_OF_XENOBIOTICS_BY_CYTOCHROMI	KEGG_METABOLISM_OF_XE	69
WP_INSULIN_SIGNALING	WP_INSULIN_SIGNALING	159
REACTOME_REGULATION_OF_RUNX1_EXPRESSION_AND_	REACTOME_REGULATION_O	17
REACTOME_FOXO_MEDIATED_TRANSCRIPTION_OF_CELL_	REACTOME_FOXO_MEDIATE	17
WP_RESISTIN_AS_A_REGULATOR_OF_INFLAMMATION	WP_RESISTIN_AS_A_REGUL/	33
WP_FATTY_ACID_TRANSPORTERS	WP_FATTY_ACID_TRANSPOF	18
REACTOME_MAPK3_ERK1_ACTIVATION	REACTOME_MAPK3_ERK1_A	10
PID_SMAD2_3PATHWAY	PID_SMAD2_3PATHWAY	16
WP_TYPE_II_INTERFERON_SIGNALING	WP_TYPE_II_INTERFERON_S	37
REACTOME_BMAL1_CLOCK_NPAS2_ACTIVATES_CIRCADIA	REACTOME_BMAL1_CLOCK_	27
WP_AMINO_ACID_METABOLISM	WP_AMINO_ACID_METABOI	91
REACTOME_SPHINGOLIPID_METABOLISM	REACTOME_SPHINGOLIPID_I	90
WP_FATTY_ACID_OMEGAOXIDATION	WP_FATTY_ACID_OMEGAO>	15
WP_FATTY_ACID_BETAOXIDATION	WP_FATTY_ACID_BETAOXID.	34
REACTOME_SIGNALLING_TO_ERKS	REACTOME_SIGNALLING_TC	34
PID_IL2_PI3K_PATHWAY	PID_IL2_PI3K_PATHWAY	34
REACTOME_PRC2_METHYLATES_HISTONES_AND_DNA	REACTOME_PRC2_METHYLA	70
REACTOME_P75_NTR_RECEPTOR_MEDIATED_SIGNALLING	REACTOME_P75_NTR_RECEI	97
WP_GENETIC_CAUSES_OF_PSVDINCPH	WP_GENETIC_CAUSES_OF_F	37
REACTOME_CRMPS_IN_SEMA3A_SIGNALING	REACTOME_CRMPS_IN_SEM	16
REACTOME_METABOLISM_OF_FAT_SOLUBLE_VITAMINS	REACTOME_METABOLISM_C	48
REACTOME_HEDGEHOG_ON_STATE	REACTOME_HEDGEHOG_ON	86
BIOCARTA_MTOR_PATHWAY	BIOCARTA_MTOR_PATHWA'	22
REACTOME_REVERSIBLE_HYDRATION_OF_CARBON_DIOXI	REACTOME_REVERSIBLE_HY	12
REACTOME_UNBLOCKING_OF_NMDA_RECEPTORS_GLUT/	REACTOME_UNBLOCKING_C	21
REACTOME_INTEGRIN_SIGNALING	REACTOME_INTEGRIN_SIGN.	27
REACTOME_SIGNALING_BY_BMP	REACTOME_SIGNALING_BY_	28
PID_ARF6_PATHWAY	PID_ARF6_PATHWAY	35
REACTOME_VOLTAGE_GATED_POTASSIUM_CHANNELS	REACTOME_VOLTAGE_GATE	42
WP_GASTRIC_ACID_PRODUCTION	WP_GASTRIC_ACID_PRODU(	11
REACTOME_PRESYNAPTIC_DEPOLARIZATION_AND_CALCII	REACTOME_PRESYNAPTIC_C	11
WP_RESOLVIN_E1_AND_RESOLVIN_D1_SIGNALING_PATH	WP_RESOLVIN_E1_AND_RES	12
REACTOME_TRAFFICKING_OF_GLUR2_CONTAINING_AMP,	REACTOME_TRAFFICKING_O	17
REACTOME_SUMOYLATION_OF_INTRACELLULAR_RECEPT(	REACTOME_SUMOYLATION_	30
REACTOME_PHASE_4_RESTING_MEMBRANE_POTENTIAL	REACTOME_PHASE_4_RESTI	19
REACTOME_PEROXISOMAL_PROTEIN_IMPORT	REACTOME_PEROXISOMAL_	63
BIOCARTA_VEGF_PATHWAY	BIOCARTA_VEGF_PATHWAY	27

BIOCARTA_IGF1MTOR_PATHWAY	BIOCARTA_IGF1MTOR_PATH	19
WP_METABOLISM_OF_SPINGOLIPIDS_IN_ER_AND_GOLGI	WP_METABOLISM_OF_SPIN	21
REACTOME_SUMOYLATION_OF_TRANSCRIPTION_FACTOR	REACTOME_SUMOYLATION_	20
REACTOME_FRS_MEDIATED_FGFR3_SIGNALING	REACTOME_FRS_MEDIATED_	20
REACTOME_PLASMA_LIPOPROTEIN_REMODELING	REACTOME_PLASMA_LIPOP	34
REACTOME_CRISTAE_FORMATION	REACTOME_CRISTAE_FORM	29
WP_BDNFTRKB_SIGNALING	WP_BDNFTRKB_SIGNALING	33
PID_MAPK_TRK_PATHWAY	PID_MAPK_TRK_PATHWAY	34
KEGG_PEROXISOME	KEGG_PEROXISOME	78
REACTOME_CELL_DEATH_SIGNALLING_VIA_NRAGE_NRIF_	REACTOME_CELL_DEATH_SI	76
REACTOME_RHOBTB2_GTPASE_CYCLE	REACTOME_RHOBTB2_GTPA	23
WP_OXYSTEROLS_DERIVED_FROM_CHOLESTEROL	WP_OXYSTEROLS_DERIVED_	46
WP_FAMILIAL_HYPERLIPIDEMIA_TYPE_4	WP_FAMILIAL_HYPERLIPIDE	22
WP_GLUTATHIONE_METABOLISM	WP_GLUTATHIONE_METABO	19
REACTOME_SIGNALING_BY_ALK	REACTOME_SIGNALING_BY_	27
REACTOME_RESOLUTION_OF_D_LOOP_STRUCTURES_THR	REACTOME_RESOLUTION_O	27
BIOCARTA_CTCF_PATHWAY	BIOCARTA_CTCF_PATHWAY	24
REACTOME_HEME_SIGNALING	REACTOME_HEME_SIGNALIN	48
REACTOME_MEIOSIS	REACTOME_MEIOSIS	116
REACTOME_ACTIVATION_OF_BH3_ONLY_PROTEINS	REACTOME_ACTIVATION_OF	30
REACTOME_HOMOLOGOUS_DNA_PAIRING_AND_STRAND	REACTOME_HOMOLOGOUS_	43
WP_EPO_RECEPTOR_SIGNALING	WP_EPO_RECEPTOR_SIGNAL	26
REACTOME_SIRT1_NEGATIVELY_REGULATES_RRNA_EXPR	REACTOME_SIRT1_NEGATIV	65
WP_CANONICAL_AND_NONCANONICAL_NOTCH_SIGNALI	WP_CANONICAL_AND_NON	27
KEGG_GLYOXYLATE_AND_DICARBOXYLATE_METABOLISM	KEGG_GLYOXYLATE_AND_DI	16
REACTOME_NICOTINATE_METABOLISM	REACTOME_NICOTINATE_M	31
REACTOME_NUCLEAR_PORE_COMPLEX_NPC_DISASSEMB	REACTOME_NUCLEAR_PORE	36
REACTOME_CARGO_TRAFFICKING_TO_THE_PERICILIARY_	REACTOME_CARGO_TRAFFIC	51
KEGG_CYSTEINE_AND_METHIONINE_METABOLISM	KEGG_CYSTEINE_AND_METH	34
REACTOME_FORMATION_OF_ATP_BY_CHEMIOSMOTIC_C	REACTOME_FORMATION_OI	16
WP_MECP2_AND_ASSOCIATED_RETT_SYNDROME	WP_MECP2_AND_ASSOCIAT	73
WP_NUCLEOTIDEBINDING_OLIGOMERIZATION_DOMAIN_	WP_NUCLEOTIDEBINDING_C	41
KEGG_TYPE_II_DIABETES_MELLITUS	KEGG_TYPE_II_DIABETES_M	47
BIOCARTA_NKCELLS_PATHWAY	BIOCARTA_NKCELLS_PATHW	20
REACTOME_HORMONE_LIGAND_BINDING_RECEPTORS	REACTOME_HORMONE_LIG	12
REACTOME_CARBOXYTERMINAL_POST_TRANSLATIONAL_	REACTOME_CARBOXYTERMI	45
WP_MITOCHONDRIAL_COMPLEX_III_ASSEMBLY	WP_MITOCHONDRIAL_COM	15
REACTOME_CHYLOMICRON_ASSEMBLY	REACTOME_CHYLOMICRON_	10
REACTOME_THE_NLRP3_INFLAMMASOME	REACTOME_THE_NLRP3_INF	16
REACTOME_FGFR1_LIGAND_BINDING_AND_ACTIVATION	REACTOME_FGFR1_LIGAND_	16
REACTOME_DEFECTIVE_B4GALT7_CAUSES_EDS_PROGERC	REACTOME_DEFECTIVE_B4G	20
REACTOME_CELL_CELL_JUNCTION_ORGANIZATION	REACTOME_CELL_CELL_JUN	63
REACTOME_DERMATAN_SULFATE_BIOSYNTHESIS	REACTOME_DERMATAN_SU	11
WP_ZINC_HOMEOSTASIS	WP_ZINC_HOMEOSTASIS	37
WP_MAMMARY_GLAND_DEVELOPMENT_PATHWAY_PUB	WP_MAMMARY_GLAND_DE	13
WP_15Q133_COPY_NUMBER_VARIATION_SYNDROME	WP_15Q133_COPY_NUMBE	20
REACTOME_INTERLEUKIN_10_SIGNALING	REACTOME_INTERLEUKIN_1	45
WP_INHIBITION_OF_EXOSOME_BIOGENESIS_AND_SECRET	WP_INHIBITION_OF_EXOSO	18
REACTOME_SHC_MEDIATED_CASCADE_FGFR3	REACTOME_SHC_MEDIATED	18
REACTOME_EPH_EPHRIN_MEDIATED_REPULSION_OF_CEL	REACTOME_EPH_EPHRIN_M	51

WP_OLIGODENDROCYTE_SPECIFICATION_AND_DIFFERENT	WP_OLIGODENDROCYTE_SP	29
WP_PPARALPHA_PATHWAY	WP_PPARALPHA_PATHWAY	26
SA_TRKA_RECEPTOR	SA_TRKA_RECEPTOR	17
REACTOME_PROLONGED_ERK_ACTIVATION_EVENTS	REACTOME_PROLONGED_EI	14
BIOCARTA_TNFR1_PATHWAY	BIOCARTA_TNFR1_PATHWA\	29
REACTOME_REGULATION_OF_TP53_ACTIVITY	REACTOME_REGULATION_O	160
REACTOME_DRUG_ADME	REACTOME_DRUG_ADME	97
BIOCARTA_GHRELIN_PATHWAY	BIOCARTA_GHRELIN_PATHW	13
REACTOME_ACTIVATED_NTRK2_SIGNALS_THROUGH_FRS2	REACTOME_ACTIVATED_NTI	11
REACTOME_B_WICH_COMPLEX_POSITIVELY_REGULATES_	REACTOME_B_WICH_COMP	85
BIOCARTA_RACC_PATHWAY	BIOCARTA_RACC_PATHWAY	15
REACTOME_INWARDLY_RECTIFYING_K_CHANNELS	REACTOME_INWARDLY_REC	35
WP_CALCIUM_REGULATION_IN_CARDIAC_CELLS	WP_CALCIUM_REGULATION	152
BIOCARTA_HSWI_SNF_PATHWAY	BIOCARTA_HSWI_SNF_PATH	12
REACTOME_SIGNALING_BY_ERYTHROPOIETIN	REACTOME_SIGNALING_BY_	25
REACTOME_REGULATION_OF_CHOLESTEROL_BIOSYNTHES	REACTOME_REGULATION_O	55
KEGG_ERBB_SIGNALING_PATHWAY	KEGG_ERBB_SIGNALING_PA	87
KEGG_TAURINE_AND_HYPOTAURINE_METABOLISM	KEGG_TAURINE_AND_HYPO	10
REACTOME_PRESYNAPTIC_NICOTINIC_ACETYLCHOLINE_RI	REACTOME_PRESYNAPTIC_N	12
WP_VALPROIC_ACID_PATHWAY	WP_VALPROIC_ACID_PATHV	12
REACTOME_SIGNALING_BY_INSULIN_RECEPTOR	REACTOME_SIGNALING_BY_	78
REACTOME_CONDENSATION_OF_PROMETAPHASE_CHROI	REACTOME_CONDENSATION	11
REACTOME_GAMMA_CARBOXYLATION_HYPUSINE_FORM	REACTOME_GAMMA_CARB(	42
BIOCARTA_SPRY_PATHWAY	BIOCARTA_SPRY_PATHWAY	17
REACTOME_CTLA4_INHIBITORY_SIGNALING	REACTOME_CTLA4_INHIBIT(	21
REACTOME_TRANS_GOLGI_NETWORK_VESICLE_BUDDING	REACTOME_TRANS_GOLGI_I	72
WP_SEROTONIN_AND_ANXIETY	WP_SEROTONIN_AND_ANXI	17
BIOCARTA_PDZS_PATHWAY	BIOCARTA_PDZS_PATHWAY	17
KEGG_FC_GAMMA_R_MEDIATED_PHAGOCYTOSIS	KEGG_FC_GAMMA_R_MEDI	96
PID_P53_REGULATION_PATHWAY	PID_P53_REGULATION_PATI	59
BIOCARTA_FIBRINOLYSIS_PATHWAY	BIOCARTA_FIBRINOLYSIS_PA	12
WP_STEROID_BIOSYNTHESIS	WP_STEROID_BIOSYNTHESIS	10
REACTOME_NUCLEAR_ENVELOPE_BREAKDOWN	REACTOME_NUCLEAR_ENVE	53
REACTOME_PI_3K_CASCADE_FGFR1	REACTOME_PI_3K_CASCADE	21
WP_STING_PATHWAY_IN_KAWASAKILIKE_DISEASE_AND_	WP_STING_PATHWAY_IN_K/	21
REACTOME_BIOSYNTHESIS_OF_SPECIALIZED_PRORESOLVI	REACTOME_BIOSYNTHESIS_(	19
REACTOME_ALK_MUTANTS_BIND_TKIS	REACTOME_ALK_MUTANTS_	12
REACTOME_INTERACTION_WITH_CUMULUS_CELLS_AND_	REACTOME_INTERACTION_V	11
REACTOME_PI_3K_CASCADE_FGFR4	REACTOME_PI_3K_CASCADE	20
BIOCARTA_NGF_PATHWAY	BIOCARTA_NGF_PATHWAY	20
REACTOME_ION_TRANSPORT_BY_P_TYPE_ATPASES	REACTOME_ION_TRANSPOR	55
WP_CHEMOKINE_SIGNALING_PATHWAY	WP_CHEMOKINE_SIGNALIN(	165
REACTOME_CONSTITUTIVE_SIGNALING_BY_LIGAND_RESP	REACTOME_CONSTITUTIVE_	19
WP_ACQUIRED_PARTIAL_LIPODYSTROPHY_BARRAQUERSI	WP_ACQUIRED_PARTIAL_LII	10
BIOCARTA_EPONFKB_PATHWAY	BIOCARTA_EPONFKB_PATHV	11
REACTOME_DNA_DOUBLE_STRAND_BREAK_REPAIR	REACTOME_DNA_DOUBLE_S	167
REACTOME_NEGATIVE_REGULATION_OF_FGFR1_SIGNALII	REACTOME_NEGATIVE_REGI	33
REACTOME_DCC_MEDIATED_ATTRACTIVE_SIGNALING	REACTOME_DCC_MEDIATED	14
WP_EXTRAFOLLICULAR_B_CELL_ACTIVATION_BY_SARSCO	WP_EXTRAFOLLICULAR_B_C	70
REACTOME_EPHA_MEDIATED_GROWTH_CONE_COLLAPSI	REACTOME_EPHA_MEDIATE	29

BIOCARTA_NFAT_PATHWAY	BIOCARTA_NFAT_PATHWAY	51
REACTOME_INTERLEUKIN_15_SIGNALING	REACTOME_INTERLEUKIN_1	14
WP_QUERCETIN_AND_NFKB_AP1_INDUCED_APOPTOSIS	WP_QUERCETIN_AND_NFKB	15
KEGG_THYROID_CANCER	KEGG_THYROID_CANCER	29
KEGG_ASCORBATE_AND_ALDARATE_METABOLISM	KEGG_ASCORBATE_AND_AL	25
REACTOME_MEIOTIC_SYNAPSIS	REACTOME_MEIOTIC_SYNAP	78
WP_ENDOMETRIAL_CANCER	WP_ENDOMETRIAL_CANCER	63
REACTOME_SODIUM_CALCIUM_EXCHANGERS	REACTOME_SODIUM_CALCIU	11
WP_PHOSPHOINOSITIDES_METABOLISM	WP_PHOSPHOINOSITIDES_M	49
WP_WNT_SIGNALING_IN_KIDNEY_DISEASE	WP_WNT_SIGNALING_IN_KI	36
REACTOME_MITOTIC_SPINDLE_CHECKPOINT	REACTOME_MITOTIC_SPIND	111
WP_NUCLEOTIDE_GPCRS	WP_NUCLEOTIDE_GPCRS	10
REACTOME_RECOGNITION_AND_ASSOCIATION_OF_DNA_	REACTOME_RECOGNITION_	55
PID_RETINOIC_ACID_PATHWAY	PID_RETINOIC_ACID_PATHW	30
KEGG_MTOR_SIGNALING_PATHWAY	KEGG_MTOR_SIGNALING_P	52
BIOCARTA_AKT_PATHWAY	BIOCARTA_AKT_PATHWAY	22
REACTOME_ACETYLCHOLINE_BINDING_AND_DOWNSTRE	REACTOME_ACETYLCHOLINE	14
REACTOME_HOMOLOGY_DIRECTED_REPAIR	REACTOME_HOMOLOGY_DI	137
REACTOME_EGFR_DOWNREGULATION	REACTOME_EGFR_DOWNRE	31
WP_APOPTOSIS	WP_APOPTOSIS	85
REACTOME_OXIDATIVE_STRESS_INDUCED_SENESCENCE	REACTOME_OXIDATIVE_STR	121
REACTOME_GLUCOCORTICOID_BIOSYNTHESIS	REACTOME_GLUCOCORTICO	10
REACTOME_VISUAL_PHOTOTRANSDUCTION	REACTOME_VISUAL_PHOTO	100
WP_NICOTINE_EFFECT_ON_DOPAMINERGIC_NEURONS	WP_NICOTINE_EFFECT_ON_	21
REACTOME_SYNTHESIS_SECRETION_AND_INACTIVATION_	REACTOME_SYNTHESIS_SEC	21
REACTOME_CLASS_B_2_SECRETIN_FAMILY_RECEPTORS	REACTOME_CLASS_B_2_SEC	94
REACTOME_REGULATION_OF_FOXO_TRANSCRIPTIONAL_	REACTOME_REGULATION_O	10
WP_SEROTONIN_RECEPTOR_2_AND_ELKSRFGATA4_SIGN	WP_SEROTONIN_RECEPTOR_	20
SA_MMP_CYTOKINE_CONNECTION	SA_MMP_CYTOKINE_CONNE	15
REACTOME_SYNTHESIS_OF_BILE_ACIDS_AND_BILE_SALTS	REACTOME_SYNTHESIS_OF_	15
WP_MTHFR_DEFICIENCY	WP_MTHFR_DEFICIENCY	24
BIOCARTA_TOLL_PATHWAY	BIOCARTA_TOLL_PATHWAY	25
BIOCARTA_INTRINSIC_PATHWAY	BIOCARTA_INTRINSIC_PATH	23
BIOCARTA_SHH_PATHWAY	BIOCARTA_SHH_PATHWAY	16
REACTOME_REGULATION_OF_KIT_SIGNALING	REACTOME_REGULATION_O	16
WP_LIPID_METABOLISM_IN_SENESCENT_CELLS	WP_LIPID_METABOLISM_IN_	20
BIOCARTA_FMLP_PATHWAY	BIOCARTA_FMLP_PATHWAY	34
REACTOME_MAP2K_AND_MAPK_ACTIVATION	REACTOME_MAP2K_AND_M	40
REACTOME_A_TETRASACCHARIDE_LINKER_SEQUENCE_IS_	REACTOME_A_TETRASACCH	26
REACTOME_ACTIVATION_OF_SMO	REACTOME_ACTIVATION_OF	18
KEGG_LONG_TERM_DEPRESSION	KEGG_LONG_TERM_DEPRES	70
REACTOME_G2_M_CHECKPOINTS	REACTOME_G2_M_CHECKP	166
REACTOME_P130CAS_LINKAGE_TO_MAPK_SIGNALING_F	REACTOME_P130CAS_LINKA	15
REACTOME_NOTCH4_INTRACELLULAR_DOMAIN_REGULA	REACTOME_NOTCH4_INTRA	20
REACTOME_SIGNALING_BY_ACTIVIN	REACTOME_SIGNALING_BY_	15
SIG_REGULATION_OF_THE_ACTIN_CYTOSKELETON_BY_R	SIG_REGULATION_OF_THE_	35
REACTOME_RIPK1_MEDIATED_REGULATED_NECROSIS	REACTOME_RIPK1_MEDIATE	29
PID_ATR_PATHWAY	PID_ATR_PATHWAY	39
WP_VITAMIN_B12_METABOLISM	WP_VITAMIN_B12_METABO	51
BIOCARTA_PS1_PATHWAY	BIOCARTA_PS1_PATHWAY	14

WP_ERBB_SIGNALING_PATHWAY	WP_ERBB_SIGNALING_PATH	91
BIOCARTA_RHO_PATHWAY	BIOCARTA_RHO_PATHWAY	21
REACTOME_DAP12_SIGNALING	REACTOME_DAP12_SIGNALI	29
WP_TNFRELATED_WEAK_INDUCER_OF_APOPTOSIS_TWE/	WP_TNFRELATED_WEAK_INI	42
REACTOME_FGFR2_MUTANT_RECEPTOR_ACTIVATION	REACTOME_FGFR2_MUTAN	33
KEGG_LYSOSOME	KEGG_LYSOSOME	121
REACTOME_REGULATION_OF_SIGNALING_BY_NODAL	REACTOME_REGULATION_O	12
REACTOME_TRANSPORT_OF_NUCLEOSIDES_AND_FREE_P	REACTOME_TRANSPORT_OF	12
BIOCARTA_TOB1_PATHWAY	BIOCARTA_TOB1_PATHWAY	19
REACTOME_RHOBTB3_ATPASE_CYCLE	REACTOME_RHOBTB3_ATPA	10
REACTOME_AKT_PHOSPHORYLATES_TARGETS_IN_THE_NI	REACTOME_AKT_PHOSPHOF	10
REACTOME_PEPTIDE_HORMONE_METABOLISM	REACTOME_PEPTIDE_HORM	91
KEGG_STEROID_HORMONE_BIOSYNTHESIS	KEGG_STEROID_HORMONE_	55
REACTOME_FGFR2B_LIGAND_BINDING_AND_ACTIVATION	REACTOME_FGFR2B_LIGANE	10
BIOCARTA_ACTINY_PATHWAY	BIOCARTA_ACTINY_PATHWA	12
PID_RAC1_PATHWAY	PID_RAC1_PATHWAY	54
REACTOME_REGULATION_OF_IFNG_SIGNALING	REACTOME_REGULATION_O	14
BIOCARTA_EFP_PATHWAY	BIOCARTA_EFP_PATHWAY	16
REACTOME_ADORA2B_MEDIATED_ANTI_INFLAMMATORY	REACTOME_ADORA2B_MED	133
WP_CONGENITAL_GENERALIZED_LIPODYSTROPHY	WP_CONGENITAL_GENERAL	18
REACTOME_PLATELET_ADHESION_TO_EXPOSED_COLLAGI	REACTOME_PLATELET_ADHE	14
REACTOME_VXPX_CARGO_TARGETING_TO_CILIUM	REACTOME_VXPX_CARGO_T	21
WP_GENE_REGULATORY_NETWORK_MODELLING_SOMIT	WP_GENE_REGULATORY_NE	11
REACTOME_GLUTAMATE_NEUROTRANSMITTER_RELEASE_	REACTOME_GLUTAMATE_NI	24
REACTOME_MET_RECEPTOR_RECYCLING	REACTOME_MET_RECEPTOF	10
REACTOME_HIGHLY_CALCIUM_PERMEABLE_POSTSYNAPT	REACTOME_HIGHLY_CALCIU	11
REACTOME_ACTIVATION_OF_BAD_AND_TRANSLOCATION	REACTOME_ACTIVATION_OF	15
SA_B_CELL_RECEPTOR_COMPLEXES	SA_B_CELL_RECEPTOR_COM	24
REACTOME_LGI_ADAM_INTERACTIONS	REACTOME_LGI_ADAM_INTI	14
WP_NUCLEAR_RECEPTORS	WP_NUCLEAR_RECEPTORS	38
PID_P38_ALPHA_BETA_DOWNSTREAM_PATHWAY	PID_P38_ALPHA_BETA_DOW	38
REACTOME_REGULATION_OF_TP53_ACTIVITY_THROUGH_	REACTOME_REGULATION_O	92
WP_ANTIVIRAL_AND_ANTIINFLAMMATORY_EFFECTS_OF_	WP_ANTIVIRAL_AND_ANTIIN	32
REACTOME_GLUCAGON_TYPE_LIGAND_RECEPTORS	REACTOME_GLUCAGON_TYF	33
WP_GPCRS_CLASS_B_SECRETINLIKE	WP_GPCRS_CLASS_B_SECRE	24
WP_HEREDITARY_LEIOMYOMATOSIS_AND_RENAL_CELL_(	WP_HEREDITARY_LEIOMYOI	20
REACTOME_INITIATION_OF_NUCLEAR_ENVELOPE_NE_REI	REACTOME_INITIATION_OF_	19
REACTOME_TP53_REGULATES_TRANSCRIPTION_OF_DEAT	REACTOME_TP53_REGULATI	12
REACTOME_INHIBITION_OF_DNA_RECOMBINATION_AT_1	REACTOME_INHIBITION_OF_	67
WP_CANNABINOID_RECEPTOR_SIGNALING	WP_CANNABINOID_RECEPT(	29
WP_CORI_CYCLE	WP_CORI_CYCLE	17
REACTOME_ANDROGEN_BIOSYNTHESIS	REACTOME_ANDROGEN_BIC	11
REACTOME_SIGNALLING_TO_RAS	REACTOME_SIGNALLING_TC	20
REACTOME_REGULATION_BY_C_FLIP	REACTOME_REGULATION_B	11
REACTOME_P2Y_RECEPTORS	REACTOME_P2Y_RECEPTORS	12
REACTOME_GOLGI_ASSOCIATED_VESICLE_BIOGENESIS	REACTOME_GOLGI_ASSOCIA	56
REACTOME_FGFRL1_MODULATION_OF_FGFR1_SIGNALIN(	REACTOME_FGFRL1_MODUL	13
BIOCARTA_AMI_PATHWAY	BIOCARTA_AMI_PATHWAY	20
REACTOME_MYOGENESIS	REACTOME_MYOGENESIS	29
REACTOME_G0_AND_EARLY_G1	REACTOME_G0_AND_EARLY	27

REACTOME_NF_KB_ACTIVATION_THROUGH_FADD_RIP_1	REACTOME_NF_KB_ACTIVAT	13
BIOCARTA_LEPTIN_PATHWAY	BIOCARTA_LEPTIN_PATHWA	11
BIOCARTA_RELA_PATHWAY	BIOCARTA_RELA_PATHWAY	15
WP_LEUKOCYTEINTRINSIC_HIPPO_PATHWAY_FUNCTIONS	WP_LEUKOCYTEINTRINSIC_H	33
REACTOME_CYTOPROTECTION_BY_HMOX1	REACTOME_CYTOPROTECTIC	60
WP_INTERACTIONS_BETWEEN_LOXL4_AND_OXIDATIVE_S	WP_INTERACTIONS_BETWEE	18
WP_SMALL_LIGAND_GPCRS	WP_SMALL_LIGAND_GPCRS	19
KEGG_GLUTATHIONE_METABOLISM	KEGG_GLUTATHIONE_META	49
REACTOME_ASSEMBLY_AND_CELL_SURFACE_PRESENTATI	REACTOME_ASSEMBLY_AND	44
REACTOME_SMOOTH_MUSCLE_CONTRACTION	REACTOME_SMOOTH_MUSC	43
NABA_ECM_AFFILIATED	NABA_ECM_AFFILIATED	169
REACTOME_DOWNREGULATION_OF_TGF_BETA_RECEPTO	REACTOME_DOWNREGULAT	26
BIOCARTA_AHSP_PATHWAY	BIOCARTA_AHSP_PATHWAY	13
REACTOME_FGFR1C_LIGAND_BINDING_AND_ACTIVATION	REACTOME_FGFR1C_LIGAND	12
KEGG_GNRH_SIGNALING_PATHWAY	KEGG_GNRH_SIGNALING_PA	101
REACTOME_GAP_JUNCTION_DEGRADATION	REACTOME_GAP_JUNCTION_	12
WP_NOTCH1_REGULATION_OF_ENDOTHELIAL_CELL_CALC	WP_NOTCH1_REGULATION_	17

enrichmentS	NES	pvalue	p.adjust	qvalue	rank	leading_edge
0.75590662	3.52382571	1E-10	4.8784E-09	3.1414E-09	2203	tags=62%, list
0.67911068	3.37568681	1E-10	4.8784E-09	3.1414E-09	3991	tags=61%, list
0.66099957	3.34653142	1E-10	4.8784E-09	3.1414E-09	4273	tags=63%, list
0.72652709	3.28972178	1E-10	4.8784E-09	3.1414E-09	3699	tags=70%, list
0.83229603	3.27087584	1E-10	4.8784E-09	3.1414E-09	1873	tags=70%, list
0.64754118	3.22804119	1E-10	4.8784E-09	3.1414E-09	3743	tags=59%, list
0.81887848	3.21814563	1E-10	4.8784E-09	3.1414E-09	1873	tags=70%, list
0.67294544	3.18674036	1E-10	4.8784E-09	3.1414E-09	2620	tags=57%, list
0.71915107	3.16193966	1E-10	4.8784E-09	3.1414E-09	3743	tags=63%, list
0.75330203	3.15536851	1E-10	4.8784E-09	3.1414E-09	5049	tags=70%, list
0.74325336	3.11456753	1E-10	4.8784E-09	3.1414E-09	4647	tags=70%, list
0.65392526	3.08812577	1E-10	4.8784E-09	3.1414E-09	4367	tags=51%, list
0.75980528	3.08483227	1E-10	4.8784E-09	3.1414E-09	3743	tags=63%, list
0.65011878	3.08073892	1E-10	4.8784E-09	3.1414E-09	5140	tags=61%, list
0.79967821	3.02592264	1E-10	4.8784E-09	3.1414E-09	1391	tags=59%, list
0.7420084	3.02185345	1E-10	4.8784E-09	3.1414E-09	3401	tags=59%, list
0.63999626	2.95177392	1E-10	4.8784E-09	3.1414E-09	3056	tags=59%, list
0.66151165	2.9267274	1E-10	4.8784E-09	3.1414E-09	4731	tags=55%, list
0.68354061	2.89627948	1E-10	4.8784E-09	3.1414E-09	2375	tags=50%, list
0.61217685	2.88206568	1E-10	4.8784E-09	3.1414E-09	4731	tags=63%, list
0.56804073	2.86185142	1E-10	4.8784E-09	3.1414E-09	4967	tags=40%, list
0.57458	2.85609136	1E-10	4.8784E-09	3.1414E-09	5133	tags=44%, list
0.51232562	2.82937053	1E-10	4.8784E-09	3.1414E-09	5133	tags=47%, list
0.64049021	2.81608619	1E-10	4.8784E-09	3.1414E-09	8715	tags=63%, list
0.55109337	2.80868298	1E-10	4.8784E-09	3.1414E-09	3318	tags=39%, list
0.51844681	2.77823515	1E-10	4.8784E-09	3.1414E-09	3991	tags=49%, list
0.54421666	2.72449513	1E-10	4.8784E-09	3.1414E-09	3554	tags=36%, list
0.51390467	2.70438904	1E-10	4.8784E-09	3.1414E-09	6753	tags=49%, list
0.51645817	2.66081975	1E-10	4.8784E-09	3.1414E-09	4567	tags=35%, list
0.50151144	2.61481926	1E-10	4.8784E-09	3.1414E-09	4460	tags=37%, list
0.46927646	2.58786971	1E-10	4.8784E-09	3.1414E-09	4571	tags=41%, list
-0.895316	-2.5443777	1E-10	4.8784E-09	3.1414E-09	732	tags=57%, list
0.48412999	2.53753254	1E-10	4.8784E-09	3.1414E-09	5398	tags=41%, list
0.45750952	2.52315034	1E-10	4.8784E-09	3.1414E-09	3856	tags=40%, list
0.47587316	2.49425493	1E-10	4.8784E-09	3.1414E-09	5334	tags=44%, list
-0.7081099	-2.4936183	1E-10	4.8784E-09	3.1414E-09	9137	tags=91%, list
-0.7031116	-2.4922399	1E-10	4.8784E-09	3.1414E-09	9137	tags=90%, list
-0.8540638	-2.4776901	1E-10	4.8784E-09	3.1414E-09	732	tags=48%, list
-0.6951643	-2.4480303	1E-10	4.8784E-09	3.1414E-09	9004	tags=89%, list
-0.674546	-2.411278	1E-10	4.8784E-09	3.1414E-09	9137	tags=82%, list
-0.6599067	-2.3702689	1E-10	4.8784E-09	3.1414E-09	9137	tags=75%, list
-0.6543053	-2.3628719	1E-10	4.8784E-09	3.1414E-09	9137	tags=72%, list
-0.6475851	-2.3314467	1E-10	4.8784E-09	3.1414E-09	9429	tags=75%, list
-0.6373194	-2.2938642	1E-10	4.8784E-09	3.1414E-09	9429	tags=72%, list
0.40736581	2.20638463	1E-10	4.8784E-09	3.1414E-09	6490	tags=46%, list
0.39309953	2.18228344	1E-10	4.8784E-09	3.1414E-09	5116	tags=38%, list
-0.5891582	-2.181546	1E-10	4.8784E-09	3.1414E-09	9137	tags=61%, list
-0.5572653	-2.0640321	1E-10	4.8784E-09	3.1414E-09	9137	tags=57%, list
-0.5395198	-2.0318269	1E-10	4.8784E-09	3.1414E-09	10829	tags=59%, list

-0.5105909	-1.9703943	1E-10	4.8784E-09	3.1414E-09	8443	tags=46%, list
-0.5069875	-1.9391662	1E-10	4.8784E-09	3.1414E-09	10697	tags=51%, list
0.69523677	2.8680295	1.8423E-10	8.8145E-09	5.6759E-09	3743	tags=60%, list
0.42223955	2.27326256	2.0125E-10	9.4474E-09	6.0835E-09	4591	tags=37%, list
0.53872304	2.63259203	2.6922E-10	1.2404E-08	7.9874E-09	5515	tags=54%, list
-0.5408167	-2.0194447	3.2381E-10	1.4648E-08	9.4323E-09	6617	tags=54%, list
0.46619123	2.42909711	4.5224E-10	2.0092E-08	1.2938E-08	5263	tags=45%, list
0.86200467	2.87306274	7.7421E-10	3.3794E-08	2.1761E-08	1929	tags=67%, list
0.39060203	2.08427765	8.7905E-10	3.7708E-08	2.4282E-08	6937	tags=44%, list
0.51677589	2.56508898	9.173E-10	3.8682E-08	2.4909E-08	6555	tags=52%, list
0.7895406	2.97069038	9.6055E-10	3.9831E-08	2.5648E-08	5514	tags=76%, list
0.43413393	2.27548176	1.1228E-09	4.5795E-08	2.9489E-08	7143	tags=47%, list
0.41808196	2.23446766	1.3352E-09	5.358E-08	3.4502E-08	4895	tags=34%, list
0.41988011	2.2294078	1.9705E-09	7.7818E-08	5.011E-08	7692	tags=45%, list
0.48636145	2.45132811	2.2619E-09	8.793E-08	5.6621E-08	7077	tags=52%, list
0.73458336	2.8868706	2.5739E-09	9.852E-08	6.344E-08	5992	tags=57%, list
0.56504764	2.62210179	2.8072E-09	1.0582E-07	6.8143E-08	5586	tags=48%, list
0.52853131	2.50845887	5.8951E-09	2.1891E-07	1.4096E-07	6377	tags=48%, list
0.55463955	2.55149417	6.6699E-09	2.405E-07	1.5487E-07	3688	tags=38%, list
0.55463955	2.55149417	6.6699E-09	2.405E-07	1.5487E-07	3688	tags=38%, list
0.53812888	2.54128377	8.7501E-09	3.11E-07	2.0027E-07	5067	tags=46%, list
0.57726511	2.53810015	9.2892E-09	3.2442E-07	2.089E-07	6422	tags=53%, list
0.4141929	2.18412235	9.3882E-09	3.2442E-07	2.089E-07	5238	tags=35%, list
0.6315921	2.64556014	1.2406E-08	4.1711E-07	2.6859E-07	2994	tags=59%, list
0.6315921	2.64556014	1.2406E-08	4.1711E-07	2.6859E-07	2994	tags=59%, list
0.41555731	2.17694131	1.337E-08	4.4353E-07	2.856E-07	5282	tags=44%, list
0.49724904	2.42991997	1.391E-08	4.4945E-07	2.8941E-07	8646	tags=62%, list
0.38363964	2.0992457	1.3879E-08	4.4945E-07	2.8941E-07	7047	tags=47%, list
0.34137243	1.92005932	1.5064E-08	4.8051E-07	3.0942E-07	5938	tags=35%, list
0.66443575	2.70593629	1.9225E-08	6.0546E-07	3.8988E-07	6708	tags=65%, list
0.61532524	2.60723916	2.0037E-08	6.2316E-07	4.0127E-07	4012	tags=43%, list
0.61415146	2.60226567	2.2458E-08	6.8981E-07	4.4419E-07	4253	tags=48%, list
0.39454125	2.10865272	2.3637E-08	7.1718E-07	4.6181E-07	7078	tags=41%, list
0.48167258	2.4055203	2.4621E-08	7.3804E-07	4.7525E-07	7141	tags=50%, list
-0.9239666	-2.1968507	2.5244E-08	7.4771E-07	4.8148E-07	358	tags=64%, list
0.51865929	2.46653173	2.6671E-08	7.8069E-07	5.0271E-07	3743	tags=43%, list
0.59740562	2.60254888	3.6216E-08	1.0366E-06	6.675E-07	6205	tags=53%, list
0.35087197	1.95155595	3.6248E-08	1.0366E-06	6.675E-07	6879	tags=40%, list
0.58220428	2.55996331	4.5359E-08	1.2824E-06	8.258E-07	4817	tags=55%, list
0.34837226	1.91946116	5.9716E-08	1.6694E-06	1.075E-06	5617	tags=40%, list
-0.5554096	-1.9985392	6.5197E-08	1.8023E-06	1.1606E-06	8524	tags=56%, list
0.72908462	2.75454734	7.7958E-08	2.1314E-06	1.3725E-06	3963	tags=65%, list
0.61781372	2.55718196	1.0302E-07	2.7859E-06	1.7939E-06	6937	tags=71%, list
0.47658264	2.35329657	1.0697E-07	2.8618E-06	1.8428E-06	7010	tags=44%, list
0.61553613	2.54775484	1.123E-07	2.9618E-06	1.9072E-06	3963	tags=60%, list
-0.6398017	-2.1366267	1.1309E-07	2.9618E-06	1.9072E-06	9061	tags=65%, list
0.32309078	1.84306937	1.201E-07	3.1125E-06	2.0042E-06	4131	tags=34%, list
0.53877225	2.41738867	1.2729E-07	3.2649E-06	2.1024E-06	3750	tags=47%, list
0.46785582	2.3124595	1.301E-07	3.3029E-06	2.1269E-06	8495	tags=55%, list
0.48935374	2.32471226	1.8071E-07	4.5414E-06	2.9244E-06	5024	tags=41%, list

0.36697976	1.9152471	1.9158E-07	4.7666E-06	3.0694E-06	5084	tags=38%, list
0.53968351	2.37721909	2.1655E-07	5.3344E-06	3.435E-06	5727	tags=54%, list
-0.5765602	-2.0360804	2.4186E-07	5.8995E-06	3.7989E-06	8524	tags=62%, list
0.73287548	2.71795057	2.4703E-07	5.967E-06	3.8423E-06	5401	tags=62%, list
0.42069092	2.14242603	2.6246E-07	6.2788E-06	4.0431E-06	7109	tags=50%, list
0.54710576	2.44002464	2.6826E-07	6.2966E-06	4.0546E-06	4475	tags=44%, list
0.41757406	2.11433889	2.6766E-07	6.2966E-06	4.0546E-06	7143	tags=50%, list
0.67159358	2.63932437	2.7885E-07	6.4838E-06	4.1751E-06	3496	tags=50%, list
0.88661656	2.5877954	2.8216E-07	6.5002E-06	4.1857E-06	3699	tags=91%, list
0.51048459	2.37973667	2.8513E-07	6.5082E-06	4.1908E-06	7143	tags=48%, list
0.48675485	2.30660095	3.1644E-07	7.1574E-06	4.6089E-06	7692	tags=51%, list
0.64193354	2.48858798	3.2207E-07	7.1872E-06	4.6281E-06	4273	tags=48%, list
0.50385226	2.32385416	3.2643E-07	7.1872E-06	4.6281E-06	4367	tags=30%, list
0.48565195	2.30147527	3.2407E-07	7.1872E-06	4.6281E-06	5473	tags=45%, list
0.44460866	2.25098007	3.4715E-07	7.5765E-06	4.8787E-06	4469	tags=40%, list
0.45846097	2.29517904	3.7953E-07	8.211E-06	5.2873E-06	5030	tags=37%, list
-0.46341	-1.7557378	4.2276E-07	9.0674E-06	5.8388E-06	6672	tags=38%, list
0.36584447	1.95414753	4.4982E-07	9.5655E-06	6.1595E-06	6659	tags=42%, list
0.44430692	2.22574817	4.6147E-07	9.73E-06	6.2655E-06	5024	tags=37%, list
0.60561764	2.47094335	4.9013E-07	1.0247E-05	6.5987E-06	5747	tags=62%, list
0.79059464	2.64110508	5.1012E-07	1.0577E-05	6.8106E-06	2803	tags=76%, list
0.53716609	2.39569494	5.2009E-07	1.0694E-05	6.8863E-06	7143	tags=59%, list
0.78228034	2.60734144	5.6027E-07	1.1426E-05	7.3574E-06	4402	tags=61%, list
0.52316374	2.30022901	6.2401E-07	1.2622E-05	8.1278E-06	4770	tags=42%, list
0.61069747	2.4870854	7.434E-07	1.4916E-05	9.6048E-06	4402	tags=41%, list
0.78308032	2.61600231	7.6766E-07	1.5279E-05	9.8389E-06	7047	tags=82%, list
-0.6462721	-2.1011792	8.908E-07	1.759E-05	1.1327E-05	8062	tags=73%, list
0.8731171	2.54839408	9.4853E-07	1.8498E-05	1.1912E-05	3743	tags=82%, list
0.47985925	2.26610866	9.5167E-07	1.8498E-05	1.1912E-05	5376	tags=43%, list
0.58397558	2.41712311	1.0022E-06	1.918E-05	1.2351E-05	4069	tags=45%, list
0.52545088	2.33630958	9.9685E-07	1.918E-05	1.2351E-05	4770	tags=44%, list
0.54893597	2.39401118	1.0827E-06	2.0563E-05	1.3241E-05	7047	tags=51%, list
0.47128916	2.17943373	1.1382E-06	2.1453E-05	1.3814E-05	5586	tags=38%, list
0.70101571	2.63761056	1.2287E-06	2.2984E-05	1.48E-05	4402	tags=56%, list
0.76753477	2.55819443	1.3087E-06	2.4298E-05	1.5646E-05	4062	tags=78%, list
0.4073954	2.07704028	1.4923E-06	2.7502E-05	1.7709E-05	5992	tags=39%, list
0.33123376	1.76748474	1.5233E-06	2.7868E-05	1.7945E-05	3992	tags=28%, list
0.51702201	2.29883236	1.5582E-06	2.8092E-05	1.8089E-05	6422	tags=53%, list
-0.5234061	-1.8843754	1.5483E-06	2.8092E-05	1.8089E-05	8524	tags=53%, list
0.39323709	2.03192135	1.6293E-06	2.9163E-05	1.8779E-05	7143	tags=51%, list
0.76896511	2.56884826	1.7221E-06	3.0604E-05	1.9707E-05	4548	tags=71%, list
0.74430078	2.52089693	1.8129E-06	3.1543E-05	2.0311E-05	646	tags=37%, list
0.58380346	2.38194066	1.7929E-06	3.1543E-05	2.0311E-05	2203	tags=51%, list
-0.5519316	-1.9506644	1.8026E-06	3.1543E-05	2.0311E-05	8524	tags=55%, list
0.61302213	2.37650693	1.9082E-06	3.297E-05	2.123E-05	5376	tags=55%, list
0.80186928	2.54982135	2.0019E-06	3.435E-05	2.2119E-05	1873	tags=73%, list
0.54205672	2.38139367	2.2044E-06	3.7565E-05	2.4189E-05	7305	tags=54%, list
0.46554316	2.19850173	2.5396E-06	4.2984E-05	2.7679E-05	7047	tags=53%, list
0.57549749	2.3365371	2.7467E-06	4.6174E-05	2.9733E-05	4865	tags=51%, list
0.44875206	2.1298191	2.8018E-06	4.6784E-05	3.0126E-05	7390	tags=47%, list

0.7350338	2.48951029	2.9027E-06	4.8146E-05	3.1003E-05	6377	tags=74%, list
0.38391382	1.98752716	2.9403E-06	4.8446E-05	3.1196E-05	7078	tags=40%, list
0.63640288	2.50102695	3.0095E-06	4.926E-05	3.172E-05	5074	tags=57%, list
0.41067457	2.07924761	3.1972E-06	5.1991E-05	3.3478E-05	7008	tags=52%, list
0.39447498	2.02626786	3.3031E-06	5.3364E-05	3.4363E-05	5024	tags=38%, list
0.45992367	2.17196401	3.6114E-06	5.7969E-05	3.7328E-05	7692	tags=57%, list
-0.7056394	-2.0983615	3.7036E-06	5.9067E-05	3.8035E-05	6026	tags=55%, list
0.5165552	2.27130303	3.7515E-06	5.9451E-05	3.8282E-05	7015	tags=57%, list
0.56209723	2.3265668	3.8657E-06	6.0781E-05	3.9139E-05	5515	tags=55%, list
0.50798477	2.27150104	3.8843E-06	6.0781E-05	3.9139E-05	6386	tags=58%, list
-0.8147428	-2.0884093	4.0028E-06	6.2244E-05	4.0081E-05	2658	tags=53%, list
0.68967185	2.51864868	4.0383E-06	6.2374E-05	4.0165E-05	5586	tags=57%, list
0.75385785	2.51838008	4.0613E-06	6.2374E-05	4.0165E-05	4817	tags=65%, list
0.61391238	2.38040727	4.1815E-06	6.3826E-05	4.1099E-05	7143	tags=59%, list
0.34511286	1.84341029	4.2645E-06	6.4695E-05	4.166E-05	4975	tags=33%, list
0.62808915	2.46595746	4.6374E-06	6.9926E-05	4.5028E-05	6591	tags=71%, list
0.45472432	2.14741038	5.0107E-06	7.51E-05	4.8359E-05	5586	tags=43%, list
0.44404966	2.1094918	5.0452E-06	7.5164E-05	4.84E-05	4571	tags=42%, list
0.38730666	1.97994997	5.2564E-06	7.7845E-05	5.0127E-05	4817	tags=37%, list
0.34399526	1.82015749	5.3663E-06	7.9002E-05	5.0872E-05	7002	tags=45%, list
0.56276185	2.29609006	5.4208E-06	7.9334E-05	5.1086E-05	7560	tags=59%, list
0.52105507	2.28410376	5.495E-06	7.995E-05	5.1483E-05	7047	tags=50%, list
0.57063841	2.30741101	6.3674E-06	9.1572E-05	5.8966E-05	5586	tags=47%, list
0.4217582	2.08461394	6.3389E-06	9.1572E-05	5.8966E-05	4571	tags=35%, list
0.72103759	2.44210606	6.4133E-06	9.1703E-05	5.9051E-05	1657	tags=42%, list
0.55609778	2.26890042	6.9905E-06	9.9016E-05	6.3759E-05	7143	tags=51%, list
0.39125435	2.0090933	7.0043E-06	9.9016E-05	6.3759E-05	5386	tags=38%, list
0.58875838	2.28244349	7.3658E-06	0.00010354	6.6671E-05	7047	tags=58%, list
0.61468512	2.42619229	7.4943E-06	0.00010475	6.7453E-05	5140	tags=48%, list
0.5062359	2.22592887	7.5401E-06	0.0001048	6.7486E-05	4899	tags=37%, list
0.42150066	2.05975836	8.1634E-06	0.00011284	7.2659E-05	4407	tags=34%, list
0.41792772	2.0656811	8.2565E-06	0.00011349	7.3081E-05	5024	tags=35%, list
0.45128899	2.09196273	8.4765E-06	0.00011588	7.4616E-05	5573	tags=34%, list
-0.4741606	-1.7698713	8.8518E-06	0.00012035	7.7494E-05	8524	tags=46%, list
0.37199783	1.91164982	9.5419E-06	0.00012902	8.3082E-05	2957	tags=26%, list
0.33908922	1.78443439	9.7429E-06	0.00013103	8.4374E-05	5386	tags=33%, list
0.60922441	2.40463861	1.0011E-05	0.00013391	8.6226E-05	6482	tags=52%, list
0.57682098	2.32757476	1.0333E-05	0.00013748	8.8528E-05	4453	tags=40%, list
0.39950301	2.01777149	1.0686E-05	0.00014142	9.1067E-05	6851	tags=43%, list
0.4092524	2.05014314	1.1033E-05	0.00014524	9.3523E-05	8264	tags=44%, list
0.45621866	2.12676406	1.1274E-05	0.00014763	9.5066E-05	4402	tags=33%, list
0.61136071	2.40027948	1.1443E-05	0.0001478	9.5174E-05	7143	tags=55%, list
0.4241681	2.00865261	1.1384E-05	0.0001478	9.5174E-05	7143	tags=51%, list
0.35930839	1.87245508	1.1465E-05	0.0001478	9.5174E-05	4291	tags=31%, list
0.324691	1.75732815	1.1555E-05	0.00014819	9.5427E-05	6736	tags=39%, list
0.67117997	2.48914591	1.1631E-05	0.00014822	9.5442E-05	2678	tags=33%, list
0.37570229	1.91331528	1.1676E-05	0.00014822	9.5442E-05	3568	tags=30%, list
0.37382312	1.90374532	1.3381E-05	0.00016899	0.00010882	5133	tags=40%, list
0.32570028	1.69981175	1.3551E-05	0.00017028	0.00010965	7047	tags=40%, list
0.57558907	2.23138994	1.3657E-05	0.00017074	0.00010995	4744	tags=48%, list

0.48582213	2.16010846	1.3908E-05	0.00017302	0.00011141	4549	tags=38%, list
0.60555435	2.37979399	1.5658E-05	0.00019382	0.00012481	2732	tags=40%, list
0.72386513	2.41264347	1.6061E-05	0.00019782	0.00012739	4897	tags=56%, list
0.55905413	2.27676621	1.629E-05	0.00019965	0.00012856	6555	tags=57%, list
0.44026218	2.04084765	1.6478E-05	0.00020097	0.00012941	2237	tags=29%, list
0.37463981	1.91519577	1.6957E-05	0.0002048	0.00013188	5044	tags=39%, list
0.33451736	1.74086208	1.6942E-05	0.0002048	0.00013188	4875	tags=34%, list
0.56833132	2.26932154	1.7105E-05	0.0002056	0.00013239	7047	tags=53%, list
0.43086882	2.03475409	1.7977E-05	0.00021503	0.00013847	6292	tags=44%, list
0.37543945	1.91157374	1.8353E-05	0.00021849	0.00014069	5376	tags=35%, list
0.43144859	1.99519463	1.8958E-05	0.00022461	0.00014463	5024	tags=35%, list
0.60579998	2.35399514	2.0986E-05	0.00024746	0.00015935	7961	tags=61%, list
0.53470198	2.1816047	2.1391E-05	0.00025105	0.00016166	8837	tags=62%, list
-0.6157303	-1.9476112	2.1613E-05	0.00025246	0.00016257	5222	tags=48%, list
0.67015674	2.41668242	2.2316E-05	0.00025774	0.00016597	5386	tags=50%, list
0.59332273	2.3418739	2.2376E-05	0.00025774	0.00016597	7047	tags=55%, list
0.41491968	2.00181195	2.2192E-05	0.00025774	0.00016597	2957	tags=33%, list
-0.5340783	-1.8587011	2.2661E-05	0.00025862	0.00016653	3901	tags=25%, list
0.34319588	1.79414409	2.262E-05	0.00025862	0.00016653	5843	tags=35%, list
0.40091641	1.98159975	2.3024E-05	0.00026157	0.00016844	9118	tags=54%, list
0.80440709	2.38691316	2.3673E-05	0.00026772	0.00017239	5446	tags=75%, list
0.67974179	2.41547884	2.3914E-05	0.00026801	0.00017258	7560	tags=62%, list
0.55951708	2.23412668	2.3869E-05	0.00026801	0.00017258	6081	tags=50%, list
0.56346222	2.18437769	2.4219E-05	0.00027021	0.000174	6081	tags=55%, list
0.39842375	1.9958972	2.4761E-05	0.00027502	0.00017709	7366	tags=43%, list
0.54801827	2.21594509	2.5722E-05	0.00028442	0.00018315	7015	tags=56%, list
0.79573703	2.39892063	2.6325E-05	0.00028853	0.0001858	5783	tags=85%, list
0.52885529	2.18166335	2.6256E-05	0.00028853	0.0001858	5311	tags=45%, list
0.52274692	2.1905459	2.8748E-05	0.00031233	0.00020112	5311	tags=44%, list
0.39717417	1.97142924	2.8691E-05	0.00031233	0.00020112	4993	tags=34%, list
0.62898701	2.37636955	3.0484E-05	0.00032976	0.00021234	5140	tags=58%, list
0.327126	1.70239666	3.1962E-05	0.00034425	0.00022167	6360	tags=39%, list
0.45321628	2.05216775	3.2435E-05	0.00034784	0.00022399	6806	tags=51%, list
0.41320807	1.96297646	3.3439E-05	0.00035707	0.00022993	5024	tags=38%, list
0.52341378	2.15921571	3.3617E-05	0.00035744	0.00023016	6896	tags=52%, list
0.52072082	2.15530643	3.534E-05	0.0003728	0.00024006	4145	tags=45%, list
0.52228146	2.1309285	3.5362E-05	0.0003728	0.00024006	6081	tags=41%, list
0.35643296	1.84525834	3.5535E-05	0.00037304	0.00024022	5170	tags=39%, list
-0.4895519	-1.7620136	3.6769E-05	0.00038437	0.00024751	8524	tags=53%, list
0.50126914	2.12396382	3.6925E-05	0.00038439	0.00024752	6961	tags=46%, list
0.55307438	2.23175302	3.7277E-05	0.00038539	0.00024816	3641	tags=49%, list
0.48878305	2.13167319	3.733E-05	0.00038539	0.00024816	6075	tags=53%, list
-0.5868313	-1.9123027	3.7858E-05	0.00038922	0.00025063	9335	tags=65%, list
0.46890596	2.08489421	3.8408E-05	0.00039325	0.00025322	4402	tags=42%, list
0.43851272	2.0172789	4.0601E-05	0.000414	0.00026659	6683	tags=40%, list
0.58580199	2.29993272	4.3769E-05	0.00044448	0.00028622	7047	tags=52%, list
0.79277213	2.3523888	4.4764E-05	0.00045274	0.00029153	4549	tags=50%, list
0.54212279	2.20781275	4.528E-05	0.00045586	0.00029354	6447	tags=59%, list
0.55271197	2.14270212	4.544E-05	0.00045586	0.00029354	7873	tags=61%, list
0.2819741	1.56497326	4.5823E-05	0.00045786	0.00029483	7972	tags=39%, list

0.59717618	2.25967007	4.7519E-05	0.00047242	0.0003042	7778	tags=56%, list
0.51721094	2.13362739	4.7849E-05	0.00047242	0.0003042	4567	tags=38%, list
0.48415255	2.10917111	4.7675E-05	0.00047242	0.0003042	5376	tags=43%, list
0.68014826	2.30361664	5.1399E-05	0.00050545	0.00032548	5992	tags=63%, list
0.37936177	1.91126614	5.2947E-05	0.00051863	0.00033396	7850	tags=40%, list
0.45086807	2.02297604	5.6713E-05	0.00055335	0.00035632	7960	tags=53%, list
0.29856393	1.61708924	5.725E-05	0.0005564	0.00035828	7921	tags=38%, list
0.58258427	2.26378441	6.1179E-05	0.00059227	0.00038138	5992	tags=50%, list
0.61230772	2.31335372	6.4277E-05	0.00061985	0.00039914	7668	tags=62%, list
0.58055262	2.28153859	6.4825E-05	0.00062032	0.00039945	7275	tags=47%, list
0.39883275	1.89289739	6.4667E-05	0.00062032	0.00039945	7305	tags=47%, list
-0.4925765	-1.7607973	6.59E-05	0.00062819	0.00040451	7176	tags=43%, list
0.36777032	1.87376637	6.7576E-05	0.00064171	0.00041322	5122	tags=32%, list
-0.5717449	-1.8748831	7.279E-05	0.0006886	0.00044341	9343	tags=50%, list
0.44059284	2.01630376	7.3841E-05	0.0006959	0.00044811	7143	tags=47%, list
0.25840062	1.50254582	7.6318E-05	0.00071652	0.00046139	5681	tags=32%, list
0.34613202	1.8035266	7.8065E-05	0.00073017	0.00047018	6683	tags=32%, list
0.44938233	1.99902101	7.9569E-05	0.00074146	0.00047745	5470	tags=43%, list
0.68573526	2.29080593	8.2022E-05	0.00076146	0.00049033	2266	tags=41%, list
0.29144933	1.56344575	8.8168E-05	0.00081547	0.00052511	7668	tags=35%, list
0.48928486	2.05668429	8.9167E-05	0.00082166	0.00052909	5518	tags=47%, list
-0.5809671	-1.8888576	9.378E-05	0.00086098	0.00055441	8524	tags=59%, list
0.32626998	1.7092001	9.7742E-05	0.00089405	0.00057571	2899	tags=26%, list
0.3681093	1.86367649	0.00010758	0.00098044	0.00063134	7890	tags=47%, list
0.48220535	2.01982139	0.00010943	0.00099365	0.00063985	6853	tags=48%, list
-0.5396966	-1.851921	0.00011021	0.0009971	0.00064207	7419	tags=51%, list
0.2854294	1.58756325	0.00011103	0.00100091	0.00064452	8224	tags=36%, list
0.80343289	2.31509966	0.00011343	0.00101376	0.0006528	5783	tags=90%, list
0.66465119	2.28899851	0.00011305	0.00101376	0.0006528	4005	tags=50%, list
0.35161418	1.79026587	0.00011368	0.00101376	0.0006528	8397	tags=45%, list
0.45407797	1.99658946	0.0001159	0.00102984	0.00066315	7047	tags=49%, list
0.53097129	2.05841992	0.00012393	0.00109725	0.00070655	8502	tags=58%, list
0.40494252	1.91891423	0.00012681	0.00111878	0.00072042	5274	tags=43%, list
0.4963163	2.07978964	0.00012846	0.00112937	0.00072724	5470	tags=47%, list
0.67545864	2.25130456	0.00012907	0.00113068	0.00072808	7322	tags=72%, list
0.60754665	2.28592804	0.0001326	0.00115598	0.00074437	5080	tags=52%, list
0.40459962	1.90481343	0.00013288	0.00115598	0.00074437	7305	tags=51%, list
0.46478785	2.03745006	0.00014344	0.0012392	0.00079796	3333	tags=34%, list
0.46415494	2.03467562	0.00014344	0.0012392	0.00079796	5367	tags=52%, list
0.64186041	2.28086645	0.00014734	0.00126846	0.00081681	7047	tags=62%, list
0.49797262	2.05426444	0.00014968	0.00128413	0.0008269	5586	tags=52%, list
0.2883107	1.55724579	0.00015407	0.00131726	0.00084823	7972	tags=39%, list
0.44018629	1.95719806	0.00015515	0.00132199	0.00085127	7047	tags=44%, list
0.29001843	1.54836671	0.0001658	0.0014079	0.00090659	6422	tags=36%, list
-0.6932945	-1.9401038	0.00017121	0.00144885	0.00093296	2482	tags=32%, list
0.61260521	2.27191485	0.00017462	0.00147274	0.00094834	3695	tags=29%, list
0.34366481	1.7584665	0.00017862	0.00150138	0.00096679	7047	tags=41%, list
0.54793075	2.15124543	0.00018011	0.00150378	0.00096833	4969	tags=48%, list
0.39442728	1.86266093	0.0001797	0.00150378	0.00096833	8835	tags=64%, list
0.61164728	2.26836227	0.00018677	0.00155411	0.00100075	4969	tags=54%, list

0.77804746	2.27091136	0.00019115	0.00158524	0.00102079	1480	tags=55%, list
0.37375494	1.82643809	0.00019365	0.00160068	0.00103073	5081	tags=38%, list
0.37194155	1.84882705	0.00019702	0.00162313	0.00104519	4715	tags=38%, list
0.31047386	1.63599673	0.00020149	0.00165445	0.00106536	7100	tags=39%, list
0.39184743	1.85694132	0.00021386	0.00175026	0.00112705	4469	tags=39%, list
0.65149233	2.20656093	0.00021577	0.00176013	0.00113341	7615	tags=68%, list
0.73156852	2.29371934	0.00022098	0.00179675	0.00115699	5140	tags=50%, list
0.77365108	2.25807951	0.00023136	0.0018689	0.00120345	6426	tags=82%, list
-0.6880816	-1.9255161	0.00023063	0.0018689	0.00120345	5195	tags=50%, list
-0.5606547	-1.8228173	0.00024234	0.00195127	0.00125649	1633	tags=22%, list
0.34172568	1.74162704	0.00024617	0.0019757	0.00127222	8397	tags=44%, list
0.34674795	1.7555868	0.00024728	0.00197823	0.00127385	7305	tags=46%, list
0.3607901	1.79083182	0.00025158	0.00200617	0.00129184	7028	tags=39%, list
0.54054807	2.12226011	0.00025612	0.00203591	0.00131099	6961	tags=52%, list
0.33272483	1.72858002	0.00025807	0.00204482	0.00131672	3765	tags=30%, list
0.483953	1.96486373	0.00026176	0.00206748	0.00133132	5727	tags=54%, list
0.39104412	1.85305344	0.00026802	0.00211024	0.00135885	7008	tags=49%, list
0.7550966	2.24059438	0.00027201	0.00213492	0.00137474	7871	tags=83%, list
0.73569976	2.21792534	0.00027739	0.00217027	0.00139751	4549	tags=38%, list
0.64574143	2.18708299	0.00028639	0.00223368	0.00143834	4106	tags=37%, list
0.70517198	2.24233875	0.00029674	0.00229505	0.00147786	6683	tags=67%, list
0.77648341	2.23744447	0.00029615	0.00229505	0.00147786	3688	tags=60%, list
0.73395843	2.21267574	0.00029703	0.00229505	0.00147786	7689	tags=69%, list
0.45035976	1.9785455	0.00030098	0.00231836	0.00149287	6377	tags=54%, list
0.51143461	1.98268191	0.000309	0.00237283	0.00152794	7047	tags=48%, list
-0.6609197	-1.8900951	0.00031936	0.00244484	0.00157432	6358	tags=56%, list
0.76676047	2.23796769	0.00032475	0.00245626	0.00158167	5476	tags=55%, list
0.5446115	2.11623121	0.00032395	0.00245626	0.00158167	7047	tags=54%, list
0.52179954	2.0232454	0.0003248	0.00245626	0.00158167	992	tags=28%, list
0.4502074	1.90760642	0.0003234	0.00245626	0.00158167	5456	tags=46%, list
0.42527016	1.90219917	0.00034229	0.00257492	0.00165807	5586	tags=37%, list
0.33371651	1.70140026	0.00034256	0.00257492	0.00165807	7047	tags=40%, list
0.31870803	1.66087545	0.00034942	0.00261852	0.00168615	7650	tags=40%, list
0.50913573	2.05445279	0.00036065	0.0026946	0.00173514	5992	tags=54%, list
0.45619834	1.91760677	0.00036473	0.00271692	0.00174951	6746	tags=47%, list
0.49961955	2.03471693	0.0003714	0.00275719	0.00177545	6742	tags=49%, list
0.42465163	1.89886915	0.00037538	0.00275719	0.00177545	7048	tags=51%, list
0.38592528	1.82879663	0.00037345	0.00275719	0.00177545	6542	tags=42%, list
0.37070567	1.78849803	0.00037568	0.00275719	0.00177545	6606	tags=42%, list
0.33997706	1.73068536	0.00037504	0.00275719	0.00177545	5263	tags=36%, list
-0.788974	-1.9158539	0.00038969	0.00285162	0.00183625	3651	tags=58%, list
-0.6847855	-1.8957172	0.00039156	0.00285687	0.00183964	8241	tags=67%, list
0.48941144	1.97896483	0.00040488	0.00294543	0.00189666	6961	tags=61%, list
0.76700839	2.21014212	0.0004112	0.00298267	0.00192064	4051	tags=60%, list
0.45089294	1.88866262	0.00042463	0.00307117	0.00197763	4280	tags=55%, list
0.41266441	1.81438923	0.00042761	0.00308375	0.00198573	7305	tags=49%, list
0.3966667	1.84073055	0.00043414	0.00312176	0.0020102	6377	tags=43%, list
0.40383214	1.84807421	0.00043729	0.00313541	0.002019	6034	tags=42%, list
0.63397426	2.14722838	0.00043913	0.00313554	0.00201908	2464	tags=32%, list
0.51597207	2.00064977	0.00044109	0.00313554	0.00201908	5586	tags=50%, list

-0.3648988	-1.4138676	0.00044018	0.00313554	0.00201908	6597	tags=31%, list
-0.8030862	-1.8771115	0.00046729	0.0033123	0.0021329	4649	tags=60%, list
0.41544685	1.82997659	0.00047386	0.00334933	0.00215674	7854	tags=51%, list
0.59142481	2.15985518	0.00048774	0.00343767	0.00221363	6700	tags=65%, list
0.38466114	1.79318287	0.00049016	0.00344497	0.00221833	4413	tags=41%, list
0.49305961	2.00800137	0.00050397	0.00352659	0.00227089	7795	tags=46%, list
0.51050403	1.97944779	0.00050461	0.00352659	0.00227089	3991	tags=44%, list
0.75306958	2.19800767	0.00051619	0.0035874	0.00231005	4503	tags=64%, list
0.75305874	2.19797601	0.00051619	0.0035874	0.00231005	3982	tags=45%, list
0.42453289	1.89336466	0.00051841	0.00359275	0.00231349	6614	tags=46%, list
0.44478236	1.86962027	0.00052362	0.0036188	0.00233026	5901	tags=42%, list
0.34166419	1.72203414	0.00054823	0.0037784	0.00243303	4292	tags=31%, list
0.60921615	2.1648643	0.00055629	0.00382335	0.00246198	6853	tags=62%, list
0.443404	1.85729357	0.0005646	0.00386975	0.00249186	6344	tags=48%, list
-0.6244028	-1.856788	0.00057852	0.00395425	0.00254627	6026	tags=42%, list
-0.6349628	-1.860695	0.00058842	0.00401091	0.00258276	2759	tags=34%, list
0.65550764	2.16168526	0.00059697	0.00404786	0.00260655	4407	tags=50%, list
0.47392618	1.9265769	0.00059709	0.00404786	0.00260655	4413	tags=50%, list
0.40895198	1.81917163	0.00062432	0.00422092	0.00271799	6782	tags=41%, list
0.59023525	2.12847394	0.0006298	0.00423741	0.00272861	5148	tags=59%, list
0.49792213	2.00920391	0.00063016	0.00423741	0.00272861	8449	tags=54%, list
0.63276404	2.11384727	0.00063398	0.00425161	0.00273775	2678	tags=41%, list
0.52215903	2.05006241	0.00064098	0.00428699	0.00276054	4899	tags=45%, list
0.62359248	2.11206599	0.00065236	0.00435139	0.002802	6742	tags=63%, list
-0.7735852	-1.8392995	0.00066643	0.00443337	0.00285479	4649	tags=55%, list
0.57960399	2.14952614	0.0006686	0.00443593	0.00285644	7008	tags=62%, list
0.49616582	2.00211689	0.00068844	0.00455543	0.00293339	5140	tags=37%, list
0.34487911	1.71924815	0.00069745	0.00460279	0.00296389	5274	tags=37%, list
0.29829973	1.59851843	0.0007025	0.00462388	0.00297747	7963	tags=39%, list
0.53029083	2.00658091	0.000725	0.00475934	0.00306469	4150	tags=41%, list
-0.4313938	-1.6002355	0.00073814	0.0048329	0.00311206	8524	tags=42%, list
0.41298257	1.83624232	0.00074071	0.00483697	0.00311469	8683	tags=47%, list
0.30972186	1.61404602	0.00075579	0.00492252	0.00316977	3820	tags=31%, list
0.28940399	1.54673944	0.00077803	0.00505412	0.00325452	6981	tags=43%, list
0.40357522	1.80515943	0.00078284	0.00507217	0.00326614	8086	tags=52%, list
0.5764252	2.13773726	0.00079127	0.00508705	0.00327572	5532	tags=58%, list
0.55418978	2.09377888	0.00079127	0.00508705	0.00327572	6147	tags=50%, list
0.31428742	1.63759977	0.00079047	0.00508705	0.00327572	7650	tags=39%, list
0.42595219	1.86720957	0.00079737	0.00511302	0.00329244	5312	tags=42%, list
0.46563155	1.89285805	0.00080018	0.00511787	0.00329556	4747	tags=39%, list
0.42681981	1.85940571	0.00080466	0.00513331	0.00330551	7504	tags=45%, list
-0.384343	-1.4655737	0.00081463	0.00518362	0.0033379	3807	tags=21%, list
0.64645445	2.1318303	0.00082284	0.00522253	0.00336296	7048	tags=50%, list
-0.7844762	-1.8336131	0.00083968	0.00531583	0.00342304	1484	tags=50%, list
0.6209245	2.07429544	0.00085774	0.00540265	0.00347894	5290	tags=65%, list
0.35487039	1.75538271	0.00085735	0.00540265	0.00347894	8015	tags=42%, list
0.70322114	2.12001155	0.00088314	0.00554865	0.00357296	5398	tags=62%, list
-0.7650216	-1.8189384	0.0009031	0.00565974	0.00364449	1624	tags=45%, list
0.31736905	1.63989861	0.00090711	0.00567057	0.00365147	5843	tags=37%, list
0.46273923	1.88110038	0.00092252	0.00575243	0.00370418	7237	tags=53%, list

0.4576912	1.88809327	0.00092553	0.00575551	0.00370616	5518	tags=48%, list
0.4287881	1.88377568	0.00092764	0.00575551	0.00370616	7260	tags=48%, list
0.55266667	2.07943906	0.00094855	0.00586602	0.00377733	4367	tags=56%, list
0.62213676	2.07358265	0.00095016	0.00586602	0.00377733	5282	tags=56%, list
0.46196842	1.8779669	0.00095517	0.00588233	0.00378783	4549	tags=37%, list
0.43174173	1.81480463	0.0009609	0.00590302	0.00380115	3743	tags=33%, list
0.54658355	2.06504186	0.00097529	0.00597664	0.00384855	4402	tags=38%, list
0.39419166	1.73316884	0.00098491	0.00602076	0.00387696	5586	tags=39%, list
0.63784724	2.10344606	0.00101645	0.00619835	0.00399132	7292	tags=62%, list
0.30634325	1.596094	0.00101983	0.00620375	0.0039948	5668	tags=36%, list
0.34628628	1.71158073	0.00102333	0.00620988	0.00399874	7650	tags=35%, list
0.61895481	2.06297721	0.00102618	0.00621199	0.0040001	6961	tags=72%, list
0.42869697	1.80200617	0.00103411	0.0062297	0.00401151	7143	tags=49%, list
0.31066234	1.59645449	0.00103204	0.0062297	0.00401151	7293	tags=47%, list
-0.4977605	-1.6779474	0.00104976	0.00630867	0.00406236	7428	tags=36%, list
0.69579098	2.09761174	0.00110071	0.00658312	0.00423909	4402	tags=46%, list
0.36882985	1.71938181	0.00110038	0.00658312	0.00423909	6989	tags=45%, list
0.33756805	1.67556597	0.00110661	0.00660251	0.00425157	4292	tags=32%, list
-0.4268625	-1.5524522	0.00111938	0.00666272	0.00429034	5819	tags=34%, list
0.5092661	1.97888735	0.00113477	0.00673818	0.00433894	5140	tags=57%, list
0.34330784	1.70649613	0.0011496	0.00681	0.00438519	7465	tags=39%, list
-0.36378	-1.4013521	0.00115398	0.00681969	0.00439142	15373	tags=68%, list
0.65861697	2.09430098	0.00119872	0.00706736	0.00455091	4747	tags=73%, list
0.34509181	1.68636925	0.00121762	0.0071618	0.00461172	8259	tags=43%, list
0.46419881	1.85352509	0.00127123	0.00745946	0.00480339	5401	tags=35%, list
0.32224879	1.63154743	0.00127686	0.00747487	0.00481332	3930	tags=25%, list
0.41799459	1.77111516	0.00128735	0.00751858	0.00484146	5586	tags=43%, list
-0.582481	-1.762005	0.00129444	0.0075423	0.00485673	6492	tags=37%, list
0.26217931	1.43851642	0.00130722	0.00759896	0.00489322	8264	tags=39%, list
0.60985833	2.03265865	0.00134226	0.00778449	0.00501269	8385	tags=56%, list
0.46260163	1.87055773	0.00135127	0.0078185	0.00503459	4402	tags=31%, list
0.56232641	2.02783064	0.00135705	0.00783374	0.0050444	4253	tags=36%, list
0.4405594	1.79749935	0.00137674	0.00792901	0.00510575	5848	tags=41%, list
0.50709676	1.9928613	0.00140454	0.00807041	0.0051968	2717	tags=33%, list
0.54452757	2.04881523	0.00141524	0.00809338	0.00521159	4280	tags=48%, list
0.4203067	1.76673808	0.00141846	0.00809338	0.00521159	5791	tags=49%, list
-0.5458846	-1.7053109	0.00142155	0.00809338	0.00521159	5464	tags=38%, list
0.33093598	1.67145969	0.00141583	0.00809338	0.00521159	4106	tags=26%, list
0.42683538	1.76670686	0.0014833	0.0084257	0.00542558	6555	tags=48%, list
0.46134897	1.84214579	0.00148683	0.00842649	0.00542609	7366	tags=44%, list
0.39310446	1.75780308	0.00149629	0.00844165	0.00543586	6961	tags=40%, list
0.30088187	1.57165476	0.00149305	0.00844165	0.00543586	5779	tags=31%, list
0.67318891	2.11067915	0.00151036	0.00848257	0.00546221	1070	tags=50%, list
0.67306814	2.11030049	0.00151036	0.00848257	0.00546221	4402	tags=50%, list
-0.5316571	-1.7202007	0.00156668	0.00877904	0.00565311	10508	tags=68%, list
0.33730863	1.67667561	0.00157228	0.00879063	0.00566058	3961	tags=30%, list
0.52856065	1.99694972	0.00166433	0.00928443	0.00597855	10157	tags=69%, list
0.59825623	1.99856852	0.00168111	0.00931538	0.00599848	4253	tags=53%, list
0.59818974	1.99834643	0.00168111	0.00931538	0.00599848	7008	tags=53%, list
0.32793321	1.63773074	0.00167641	0.00931538	0.00599848	4407	tags=26%, list

0.5848694	2.01423724	0.0016882	0.00933387	0.00601038	4549	tags=50%, list
-0.4015276	-1.5007567	0.00171869	0.00948136	0.00610536	9093	tags=42%, list
0.58283573	2.00723348	0.00173292	0.00953872	0.0061423	10337	tags=90%, list
0.31951464	1.64615631	0.00177318	0.00973879	0.00627113	7889	tags=36%, list
0.3815284	1.67749145	0.00177781	0.0097427	0.00627364	8919	tags=49%, list
0.6892789	2.04529384	0.00180058	0.00984583	0.00634005	7015	tags=58%, list
0.71941991	2.09979333	0.00180949	0.00987286	0.00635746	7293	tags=73%, list
-0.4285691	-1.5508186	0.00186756	0.01016735	0.0065471	8084	tags=34%, list
0.55101067	2.01226467	0.00189863	0.01031395	0.0066415	10157	tags=83%, list
0.38647046	1.73403382	0.00193028	0.01046302	0.00673748	5151	tags=37%, list
0.3353139	1.63858726	0.00195509	0.01057447	0.00680925	6219	tags=33%, list
0.54958385	2.00705399	0.00197028	0.01062431	0.00684134	3405	tags=52%, list
0.55073608	1.9860342	0.00197284	0.01062431	0.00684134	1445	tags=32%, list
0.38801299	1.72522021	0.00211337	0.01135651	0.00731283	5415	tags=40%, list
0.71512615	2.08726098	0.00211968	0.0113659	0.00731888	8998	tags=82%, list
0.54700255	1.99762722	0.00216133	0.01156428	0.00744662	8683	tags=61%, list
0.32582101	1.60885734	0.00217497	0.0116123	0.00747754	4596	tags=26%, list
0.43435844	1.76350831	0.00219436	0.01166575	0.00751196	7143	tags=46%, list
0.35435243	1.6682548	0.00219088	0.01166575	0.00751196	7749	tags=39%, list
0.30594888	1.56548135	0.00220303	0.01168685	0.00752555	6782	tags=40%, list
0.57380703	1.97613946	0.00222484	0.01177744	0.00758388	3695	tags=30%, list
0.40640701	1.7220166	0.00224242	0.01184534	0.0076276	5668	tags=46%, list
0.57287454	1.97292805	0.00226956	0.01196326	0.00770354	4567	tags=50%, list
0.60994068	2.01141785	0.00228116	0.0119737	0.00771026	8201	tags=62%, list
0.27527223	1.46811756	0.00227931	0.0119737	0.00771026	6739	tags=35%, list
0.58731445	1.9620158	0.00230279	0.01206175	0.00776696	4367	tags=41%, list
0.36295724	1.66970291	0.00232743	0.01213971	0.00781716	4772	tags=41%, list
-0.373702	-1.4253507	0.002324	0.01213971	0.00781716	5477	tags=23%, list
0.36219043	1.66617541	0.00235059	0.01223485	0.00787842	4304	tags=35%, list
0.46055487	1.85842042	0.00242789	0.01261082	0.00812053	7028	tags=46%, list
0.34310302	1.6259445	0.00244727	0.01268501	0.00816829	4661	tags=29%, list
0.27908373	1.47059098	0.00247553	0.01280485	0.00824546	6316	tags=30%, list
0.40640361	1.70230943	0.00249291	0.01286797	0.00828611	7008	tags=45%, list
0.32488792	1.62752066	0.00250271	0.0128918	0.00830145	6219	tags=34%, list
0.48708173	1.91420334	0.00251268	0.01291641	0.00831731	4253	tags=47%, list
0.34125735	1.62288055	0.00254344	0.01304759	0.00840177	6606	tags=40%, list
-0.5900695	-1.7546909	0.00255954	0.01308794	0.00842776	9093	tags=68%, list
0.32682672	1.64293193	0.00256183	0.01308794	0.00842776	5649	tags=33%, list
0.33233033	1.57375383	0.00257118	0.01310881	0.00844119	7504	tags=46%, list
-0.5154058	-1.6757027	0.00261549	0.01330744	0.0085691	7428	tags=35%, list
0.45399108	1.75999021	0.00263046	0.01334881	0.00859574	5080	tags=45%, list
0.40408345	1.69259094	0.00263435	0.01334881	0.00859574	4567	tags=34%, list
0.42711359	1.78979901	0.00266646	0.01348404	0.00868282	7275	tags=35%, list
0.3770439	1.67723257	0.00274129	0.01383434	0.00890839	7028	tags=46%, list
0.38428109	1.71384659	0.00275763	0.01388865	0.00894336	7292	tags=46%, list
0.40054655	1.75970334	0.00276948	0.01392012	0.00896362	5013	tags=48%, list
0.27208335	1.45332528	0.00279947	0.0140425	0.00904243	7053	tags=41%, list
0.38126996	1.69523871	0.00282478	0.01414096	0.00910583	7650	tags=42%, list
0.34735615	1.61017912	0.00290053	0.01449099	0.00933123	6939	tags=43%, list
0.28466755	1.50738677	0.00301685	0.01504191	0.00968598	7143	tags=41%, list

0.48133106	1.89160354	0.00303013	0.01507791	0.00970917	4729	tags=47%, list
0.70395774	2.05466341	0.00308473	0.01531896	0.00986438	6515	tags=64%, list
0.34662592	1.60679415	0.00309885	0.01535844	0.00988981	6834	tags=40%, list
0.553148	1.90499163	0.00312343	0.01544948	0.00994843	6961	tags=60%, list
0.30826618	1.58820366	0.00313834	0.01549244	0.00997609	6805	tags=34%, list
0.28226445	1.47664631	0.003149	0.0155143	0.00999017	6591	tags=35%, list
0.31931371	1.59959676	0.00315558	0.01551598	0.00999125	5312	tags=31%, list
0.4223179	1.7697029	0.0032031	0.01571855	0.01012169	6422	tags=47%, list
0.6454433	2.02368712	0.00323937	0.01583409	0.01019609	8397	tags=79%, list
-0.7135042	-1.7700156	0.00323819	0.01583409	0.01019609	4338	tags=62%, list
0.5506403	1.95671358	0.00330319	0.01608285	0.01035628	1746	tags=33%, list
0.5515051	1.89933362	0.00330319	0.01608285	0.01035628	7092	tags=75%, list
-0.6348765	-1.7575523	0.00331832	0.01610293	0.01036921	4648	tags=52%, list
0.36711911	1.66231893	0.0033371	0.01610293	0.01036921	7292	tags=46%, list
0.31653348	1.56299684	0.00333968	0.01610293	0.01036921	6834	tags=36%, list
0.2707644	1.46700319	0.00333775	0.01610293	0.01036921	7175	tags=34%, list
-0.385456	-1.4470766	0.0033309	0.01610293	0.01036921	5774	tags=33%, list
0.55028287	1.8951244	0.00334813	0.01611247	0.01037535	4527	tags=50%, list
-0.3481138	-1.3381781	0.00336176	0.01614685	0.01039749	15373	tags=64%, list
0.47747609	1.85535892	0.00338166	0.01617993	0.01041879	2539	tags=39%, list
0.39125722	1.70634503	0.0033811	0.01617993	0.01041879	7292	tags=39%, list
0.41969844	1.75872619	0.00340434	0.01625718	0.01046854	4453	tags=30%, list
0.26950214	1.43953782	0.00341111	0.0162583	0.01046926	5024	tags=28%, list
0.3723652	1.66555954	0.00350736	0.01668509	0.01074408	7008	tags=50%, list
0.54764082	1.88602539	0.00361777	0.01717753	0.01106118	3333	tags=45%, list
0.54696072	1.94363813	0.00366272	0.01732478	0.011156	7650	tags=57%, list
0.3730825	1.65883482	0.00366174	0.01732478	0.011156	7143	tags=45%, list
0.51204399	1.92659398	0.00369072	0.01742412	0.01121996	5140	tags=44%, list
-0.6203077	-1.7628379	0.00372178	0.0175375	0.01129297	5850	tags=39%, list
0.43801555	1.74897649	0.00374266	0.01760251	0.01133484	4542	tags=35%, list
-0.5491702	-1.6907649	0.00375501	0.0176273	0.0113508	3327	tags=37%, list
-0.6193261	-1.7600484	0.00381311	0.01786635	0.01150473	5448	tags=43%, list
-0.5760516	-1.7037515	0.00392797	0.0183699	0.01182899	2388	tags=33%, list
0.31366487	1.58803332	0.00395569	0.01846485	0.01189013	2324	tags=22%, list
0.39162582	1.65938616	0.00396771	0.01848627	0.01190392	7850	tags=52%, list
0.65804364	1.95260962	0.00399122	0.01854785	0.01194357	4567	tags=75%, list
-0.3975946	-1.4797139	0.00399584	0.01854785	0.01194357	10828	tags=39%, list
0.41572835	1.74208969	0.00401103	0.0185837	0.01196666	5140	tags=40%, list
0.32399341	1.60264832	0.00402651	0.01862072	0.01199049	7015	tags=40%, list
0.37187288	1.66286407	0.00405689	0.01872642	0.01205856	2269	tags=28%, list
0.65370252	1.97072701	0.00412599	0.01901013	0.01224125	5586	tags=46%, list
0.27163969	1.45565355	0.00415087	0.01908941	0.0122923	7532	tags=37%, list
0.37073528	1.64839838	0.00416392	0.01911407	0.01230818	5586	tags=35%, list
0.27574009	1.43709976	0.00420172	0.01925208	0.01239705	6345	tags=29%, list
0.584815	1.92856021	0.0042364	0.019343	0.01245559	6147	tags=50%, list
0.50246955	1.89837519	0.00423711	0.019343	0.01245559	2207	tags=31%, list
0.47030631	1.77960397	0.00425294	0.01937971	0.01247924	4567	tags=41%, list
0.55894434	1.89310708	0.00427397	0.01943994	0.01251802	5566	tags=47%, list
0.4687768	1.85028498	0.00435979	0.01977887	0.01273627	8502	tags=48%, list
0.32234211	1.59448009	0.00436439	0.01977887	0.01273627	7047	tags=37%, list

0.36295336	1.59582132	0.00438409	0.01983204	0.01277051	5332	tags=34%, list
0.31242536	1.5442172	0.00441894	0.01995341	0.01284866	7323	tags=40%, list
0.41830478	1.72561418	0.00445398	0.02007519	0.01292708	4999	tags=35%, list
0.65420869	1.94123019	0.00458802	0.02060745	0.01326982	7015	tags=67%, list
0.32166188	1.52663726	0.00458864	0.02060745	0.01326982	8998	tags=53%, list
0.36762048	1.63531367	0.00462813	0.02072955	0.01334844	7275	tags=38%, list
-0.4250638	-1.5066755	0.00463249	0.02072955	0.01334844	10697	tags=46%, list
0.31681492	1.54818782	0.00464392	0.0207434	0.01335736	6708	tags=39%, list
-0.5064632	-1.634648	0.00465229	0.02074355	0.01335746	4203	tags=18%, list
0.39517435	1.63565924	0.00468225	0.02083978	0.01341942	5617	tags=36%, list
0.69101009	2.01687268	0.00474521	0.0210447	0.01355137	5779	tags=64%, list
0.26555441	1.38591379	0.00473769	0.0210447	0.01355137	7293	tags=40%, list
0.68988593	2.01359156	0.00481448	0.02131392	0.01372474	7124	tags=82%, list
0.52135828	1.88009361	0.00483702	0.02137569	0.01376451	4647	tags=41%, list
0.46650047	1.76520295	0.00486414	0.0214574	0.01381713	9118	tags=44%, list
0.36590172	1.63616347	0.00490857	0.02157468	0.01389265	8917	tags=55%, list
0.3888845	1.63465646	0.00491674	0.02157468	0.01389265	7650	tags=47%, list
0.27523307	1.44468101	0.00490787	0.02157468	0.01389265	5724	tags=35%, list
0.68814577	2.00851252	0.00495302	0.02169563	0.01397053	6606	tags=73%, list
0.41592509	1.71579736	0.00497414	0.02174986	0.01400545	5312	tags=42%, list
0.46489461	1.80647027	0.00503489	0.02197126	0.01414802	7889	tags=61%, list
0.36974755	1.64902879	0.00504244	0.02197126	0.01414802	4571	tags=41%, list
0.53719625	1.90893983	0.00510348	0.02219835	0.01429425	8105	tags=48%, list
0.42572542	1.72144652	0.00515701	0.02239205	0.01441898	6081	tags=39%, list
0.4611368	1.82012953	0.00525763	0.02278919	0.01467471	4819	tags=45%, list
0.55196626	1.84392965	0.00532038	0.02302105	0.01482402	5532	tags=59%, list
0.55540078	1.85115156	0.00542221	0.02342091	0.0150815	3455	tags=44%, list
0.55427482	1.84739873	0.00551037	0.02372621	0.01527809	5573	tags=67%, list
-0.7200065	-1.7119091	0.00551196	0.02372621	0.01527809	4766	tags=55%, list
-0.4142885	-1.4857905	0.00559054	0.0240229	0.01546914	4789	tags=24%, list
-0.6049032	-1.692751	0.00561522	0.02404591	0.01548395	3286	tags=50%, list
0.28966337	1.46667764	0.00561066	0.02404591	0.01548395	6219	tags=31%, list
-0.4493473	-1.5514094	0.0056293	0.02406475	0.01549609	4789	tags=29%, list
0.42152173	1.70444867	0.00582048	0.02483937	0.01599489	9056	tags=58%, list
0.53120949	1.88766575	0.00600673	0.02554142	0.01644696	6081	tags=48%, list
0.49011227	1.84407464	0.00600048	0.02554142	0.01644696	4253	tags=48%, list
0.45295732	1.77836773	0.00601578	0.02554142	0.01644696	5140	tags=48%, list
0.59991752	1.90764573	0.00604222	0.02560996	0.0164911	5133	tags=40%, list
0.61287084	1.92156122	0.00610126	0.02581621	0.01662391	6742	tags=71%, list
0.61272534	1.92110502	0.00618315	0.02611831	0.01681844	4253	tags=64%, list
0.28556468	1.46117933	0.00621616	0.02621322	0.01687956	6879	tags=31%, list
0.36223089	1.62022959	0.00627894	0.02643317	0.01702119	5140	tags=42%, list
0.4547372	1.767001	0.00629326	0.02644872	0.0170312	6694	tags=43%, list
-0.5772583	-1.6783139	0.00631663	0.02645755	0.01703689	4846	tags=36%, list
0.37395295	1.63087789	0.00631307	0.02645755	0.01703689	7047	tags=43%, list
0.51180662	1.898092	0.00642443	0.02681877	0.01726949	5586	tags=42%, list
0.30174486	1.52402399	0.00642116	0.02681877	0.01726949	5938	tags=30%, list
0.41789477	1.68978282	0.00648573	0.02702931	0.01740506	4661	tags=36%, list
0.48617135	1.83679915	0.00662363	0.02755786	0.01774542	7292	tags=46%, list
-0.6880764	-1.706936	0.00670119	0.02783401	0.01792324	5121	tags=38%, list

0.67080361	1.93292709	0.00677958	0.02785869	0.01793913	6683	tags=60%, list
0.6081118	1.90664	0.00675642	0.02785869	0.01793913	2147	tags=21%, list
0.48172917	1.81253276	0.00675643	0.02785869	0.01793913	10162	tags=64%, list
0.45398903	1.79191696	0.00675978	0.02785869	0.01793913	9053	tags=55%, list
0.45346774	1.76206817	0.00679671	0.02785869	0.01793913	5001	tags=50%, list
-0.5979958	-1.7120398	0.00678763	0.02785869	0.01793913	10268	tags=50%, list
-0.5975845	-1.6722706	0.00672896	0.02785869	0.01793913	4368	tags=41%, list
0.35859767	1.57676079	0.00677806	0.02785869	0.01793913	6412	tags=37%, list
-0.5470809	-1.6344677	0.00685131	0.02803628	0.01805349	5798	tags=38%, list
0.59492844	1.89178121	0.00689926	0.02818614	0.01814999	2652	tags=47%, list
-0.495068	-1.6018149	0.00691552	0.02820624	0.01816293	4405	tags=32%, list
-0.6508137	-1.6682141	0.0069518	0.0283078	0.01822833	9135	tags=67%, list
-0.6253656	-1.6975852	0.00697547	0.02835779	0.01826052	8394	tags=63%, list
0.53980572	1.80330547	0.00705055	0.02856966	0.01839695	5001	tags=53%, list
0.53980572	1.80330547	0.00705055	0.02856966	0.01839695	5001	tags=53%, list
0.33870632	1.57176562	0.00708439	0.02866009	0.01845518	7305	tags=46%, list
0.45132814	1.75375418	0.00719948	0.0290784	0.01872454	4542	tags=39%, list
0.39500338	1.65524273	0.00724876	0.0291827	0.0187917	7028	tags=49%, list
-0.3744129	-1.4100357	0.00723894	0.0291827	0.0187917	5448	tags=29%, list
-0.5302229	-1.6414489	0.00726311	0.02919323	0.01879848	380	tags=13%, list
0.6670479	1.92210499	0.00747697	0.02995606	0.01928969	4549	tags=50%, list
0.56096336	1.84990403	0.0074667	0.02995606	0.01928969	6377	tags=56%, list
0.34726819	1.55813922	0.00760852	0.03043406	0.0195975	7047	tags=35%, list
0.38116419	1.57767003	0.00769973	0.03072772	0.01978659	2620	tags=36%, list
-0.4759675	-1.560807	0.00771898	0.03072772	0.01978659	5819	tags=33%, list
0.30576386	1.53705059	0.00770953	0.03072772	0.01978659	6472	tags=35%, list
-0.3922748	-1.4439769	0.00774103	0.03076628	0.01981142	8524	tags=38%, list
0.53767393	1.8210656	0.00778901	0.03089263	0.01989278	4241	tags=32%, list
-0.4781695	-1.5582072	0.00779766	0.03089263	0.01989278	7176	tags=35%, list
0.60335253	1.89171804	0.00790295	0.03124517	0.0201198	1575	tags=43%, list
-0.6220978	-1.6887145	0.00791176	0.03124517	0.0201198	2759	tags=32%, list
-0.7111254	-1.6907932	0.00793918	0.03130378	0.02015754	2388	tags=36%, list
0.31425193	1.49287776	0.00795617	0.03132111	0.0201687	6219	tags=34%, list
0.36898462	1.6210437	0.0079703	0.03132716	0.02017259	3568	tags=33%, list
-0.6990656	-1.6975306	0.00803516	0.03140021	0.02021963	2199	tags=33%, list
0.44773425	1.69419295	0.00803605	0.03140021	0.02021963	6081	tags=41%, list
0.4477229	1.69415001	0.00803605	0.03140021	0.02021963	7047	tags=52%, list
-0.3817123	-1.4138084	0.00803936	0.03140021	0.02021963	5687	tags=26%, list
0.65757684	1.91929002	0.008145	0.03176295	0.02045321	4367	tags=64%, list
0.40054988	1.6523707	0.00816785	0.03180222	0.0204785	6759	tags=52%, list
0.36621845	1.60889123	0.00840901	0.03267018	0.0210374	7584	tags=50%, list
0.272226	1.41864477	0.00841703	0.03267018	0.0210374	7795	tags=42%, list
0.35812427	1.59719036	0.00845957	0.03278412	0.02111077	4567	tags=37%, list
0.47646692	1.79273326	0.00855182	0.03309009	0.0213078	2559	tags=36%, list
0.44957795	1.76681566	0.00858867	0.03318106	0.02136638	7692	tags=53%, list
0.60085794	1.88389664	0.00864162	0.03330753	0.02144782	8647	tags=64%, list
0.34378667	1.5425182	0.00864818	0.03330753	0.02144782	5586	tags=37%, list
0.29202474	1.48784747	0.00866669	0.03332725	0.02146051	5938	tags=31%, list
0.4956607	1.83821306	0.00871527	0.03342947	0.02152634	3659	tags=46%, list
0.50217782	1.83392944	0.00873092	0.03342947	0.02152634	8449	tags=61%, list

0.26956316	1.4274052	0.00873358	0.03342947	0.02152634	7210	tags=38%, list
0.53229486	1.77821426	0.00878073	0.0335583	0.0216093	5586	tags=59%, list
0.44441162	1.72687823	0.00881052	0.03362052	0.02164936	6961	tags=36%, list
0.29680137	1.50265623	0.00895319	0.03411261	0.02196623	7047	tags=39%, list
0.37772643	1.56344084	0.00901589	0.03429899	0.02208625	4402	tags=31%, list
0.43777097	1.71874423	0.00905071	0.03437887	0.02213769	8397	tags=48%, list
0.55124839	1.81786671	0.00939051	0.03561524	0.02293383	4367	tags=62%, list
0.66029741	1.90265338	0.00943352	0.03570985	0.02299475	3789	tags=60%, list
0.30152564	1.49880741	0.00944416	0.03570985	0.02299475	7048	tags=37%, list
0.51351416	1.82478495	0.0095312	0.03598424	0.02317144	4834	tags=43%, list
0.37055126	1.5575936	0.00958484	0.03613193	0.02326654	7143	tags=44%, list
0.49140848	1.82244325	0.00961169	0.03617835	0.02329643	8119	tags=62%, list
0.26034729	1.38717054	0.00980723	0.03685861	0.02373447	5367	tags=32%, list
0.38672349	1.62054624	0.00982302	0.03686224	0.02373681	3568	tags=30%, list
-0.4997868	-1.5622307	0.00988791	0.03704987	0.02385763	5783	tags=29%, list
-0.667935	-1.6797665	0.00991616	0.03707975	0.02387688	7979	tags=64%, list
0.37438361	1.56818675	0.00992753	0.03707975	0.02387688	6079	tags=39%, list
0.31644594	1.46337538	0.00994059	0.03707975	0.02387688	5024	tags=36%, list
0.30479446	1.44658289	0.00998658	0.03719554	0.02395144	3568	tags=22%, list
0.36183822	1.58964774	0.01001766	0.03725551	0.02399005	5140	tags=31%, list
0.49359525	1.7799761	0.01009216	0.03747657	0.0241324	6805	tags=64%, list
-0.7121079	-1.6644614	0.01025136	0.03801099	0.02447653	5786	tags=60%, list
-0.4451866	-1.5378931	0.0103038	0.03814861	0.02456515	7711	tags=38%, list
-0.5023772	-1.5890652	0.01036803	0.03832937	0.02468155	5152	tags=41%, list
-0.3721814	-1.3928499	0.01043598	0.03852332	0.02480644	5975	tags=24%, list
0.52800454	1.75983985	0.01054356	0.0388628	0.02502504	4865	tags=44%, list
0.48902815	1.76350645	0.01064768	0.0391885	0.02523477	7293	tags=45%, list
0.54177442	1.78662414	0.01079466	0.03961228	0.02550765	7535	tags=56%, list
0.39238325	1.61868128	0.01078152	0.03961228	0.02550765	6081	tags=42%, list
0.32011364	1.50706213	0.01103777	0.04044473	0.02604369	8397	tags=46%, list
-0.4430574	-1.5051469	0.0110851	0.04055843	0.02611691	4928	tags=23%, list
-0.5681071	-1.6481127	0.01119331	0.0408942	0.02633312	7979	tags=48%, list
0.25893606	1.381871	0.01125658	0.04106507	0.02644315	5586	tags=32%, list
0.49430438	1.80517601	0.0113433	0.04132084	0.02660785	3568	tags=43%, list
0.3490659	1.55204993	0.011411	0.04150667	0.02672751	7497	tags=42%, list
0.63916915	1.86556293	0.01148647	0.04166291	0.02682812	7003	tags=55%, list
0.39735736	1.58663015	0.01148744	0.04166291	0.02682812	6377	tags=44%, list
-0.3652772	-1.3713215	0.01163107	0.04212243	0.02712402	6716	tags=31%, list
0.49281793	1.79974755	0.0117311	0.04231294	0.0272467	5586	tags=35%, list
0.50129874	1.72642746	0.01170969	0.04231294	0.0272467	3891	tags=55%, list
0.32643835	1.50170599	0.01175171	0.04231294	0.0272467	3568	tags=24%, list
0.29582413	1.48708437	0.01173784	0.04231294	0.0272467	3274	tags=27%, list
0.27979274	1.41659218	0.01177738	0.04234411	0.02726677	6120	tags=35%, list
0.42599462	1.67250876	0.01187313	0.04262678	0.02744879	5114	tags=39%, list
0.51308272	1.73777682	0.01189781	0.04265382	0.0274662	8020	tags=58%, list
0.26877315	1.36876401	0.01201015	0.04299462	0.02768565	5367	tags=32%, list
0.65019908	1.87355496	0.01208883	0.04316474	0.0277952	6961	tags=50%, list
-0.600199	-1.6615533	0.01209237	0.04316474	0.0277952	7462	tags=48%, list
0.3154831	1.46243075	0.01211755	0.04319264	0.02781317	7161	tags=38%, list
0.63757366	1.86090613	0.01218261	0.04336243	0.0279225	6606	tags=55%, list

0.38351689	1.55905035	0.01226228	0.04358366	0.02806496	8986	tags=53%, list
-0.7031633	-1.6435545	0.01228127	0.04358886	0.0280683	3651	tags=50%, list
0.26024335	1.38972432	0.0124252	0.0440369	0.02835681	6884	tags=29%, list
-0.578395	-1.6437272	0.01245195	0.04406891	0.02837742	8981	tags=57%, list
0.5197582	1.73633358	0.01260589	0.04453659	0.02867858	6509	tags=47%, list
0.34772216	1.55533323	0.01261989	0.04453659	0.02867858	4549	tags=35%, list
-0.5408262	-1.6082562	0.0126771	0.04467509	0.02876776	5137	tags=29%, list
0.33950352	1.49271786	0.0127963	0.0450314	0.0289972	6061	tags=32%, list
0.40739269	1.57934194	0.01296243	0.04555158	0.02933216	4834	tags=42%, list
0.29313031	1.394704	0.01301387	0.04566786	0.02940704	7889	tags=39%, list
-0.3954437	-1.4182063	0.01312207	0.0459827	0.02960978	5845	tags=29%, list
0.30807914	1.46509854	0.01323755	0.04632213	0.02982835	4378	tags=25%, list
0.26362658	1.38375927	0.01340831	0.04685377	0.03017069	6763	tags=33%, list
-0.6046068	-1.6412344	0.01345528	0.04688945	0.03019367	4405	tags=47%, list
0.40307779	1.56261433	0.01345622	0.04688945	0.03019367	7179	tags=45%, list
0.43082489	1.63020922	0.01347673	0.04689527	0.03019741	3568	tags=41%, list
-0.6564377	-1.6508524	0.01357444	0.0471693	0.03037387	3733	tags=43%, list
0.35675469	1.56387451	0.0136583	0.0473945	0.03051888	5650	tags=42%, list
0.5060024	1.71379625	0.01368692	0.04742767	0.03054024	6081	tags=53%, list
0.45716505	1.72721073	0.01371306	0.04745215	0.030556	4367	tags=46%, list
0.28455259	1.44064268	0.01382364	0.04776834	0.03075961	8205	tags=47%, list
0.48928172	1.78683349	0.01386398	0.0478413	0.03080659	253	tags=17%, list
-0.4594273	-1.5363735	0.01396886	0.04813646	0.03099666	9320	tags=55%, list
-0.4432984	-1.5047981	0.01401032	0.04821256	0.03104566	5050	tags=28%, list
-0.6902674	-1.6412005	0.01412736	0.04854817	0.03126177	5183	tags=64%, list
0.45751369	1.72142068	0.01414935	0.04855668	0.03126725	3485	tags=40%, list
0.64265216	1.85180843	0.01418514	0.04861243	0.03130315	7292	tags=50%, list
0.42644826	1.65707685	0.01426768	0.04882804	0.03144199	7293	tags=36%, list
-0.6973724	-1.6300191	0.01431119	0.04890965	0.03149454	5786	tags=60%, list
-0.6722539	-1.6324243	0.0143663	0.04896349	0.0315292	4649	tags=42%, list
-0.6713107	-1.6301339	0.0143663	0.04896349	0.0315292	4846	tags=42%, list
0.63206508	1.84482807	0.01441164	0.04898383	0.0315423	6470	tags=45%, list
0.60120326	1.81245669	0.0144115	0.04898383	0.0315423	7850	tags=62%, list
0.50443006	1.70847087	0.01458148	0.04947018	0.03185548	9423	tags=58%, list
0.341137	1.52542572	0.0145945	0.04947018	0.03185548	3070	tags=23%, list
0.62906304	1.8360659	0.01469147	0.04973112	0.03202351	3963	tags=55%, list
0.38378018	1.55815905	0.01473003	0.0497939	0.03206394	5458	tags=44%, list
0.22946918	1.26929154	0.01493892	0.05043153	0.03247452	7873	tags=36%, list
0.400824	1.55387704	0.01518449	0.05119108	0.03296363	4769	tags=36%, list
0.57858014	1.81404808	0.01522555	0.05126004	0.03300803	7366	tags=57%, list
0.42373088	1.64651777	0.01527956	0.05137234	0.03308035	3985	tags=29%, list
0.27442708	1.42571016	0.01538192	0.05164673	0.03325703	7667	tags=40%, list
0.33514451	1.49084836	0.01550313	0.05191358	0.03342887	7143	tags=45%, list
0.2903969	1.45980212	0.0154952	0.05191358	0.03342887	7143	tags=39%, list
-0.6876347	-1.634941	0.01555024	0.05200133	0.03348537	5849	tags=64%, list
0.48295302	1.76372138	0.01560907	0.05205813	0.03352194	6961	tags=52%, list
0.28017526	1.41212156	0.0156033	0.05205813	0.03352194	5586	tags=30%, list
0.52785303	1.74071521	0.01570922	0.05232199	0.03369186	4367	tags=38%, list
-0.6863715	-1.6319376	0.01575351	0.05233347	0.03369924	5873	tags=45%, list
0.31097892	1.43428878	0.01575473	0.05233347	0.03369924	3930	tags=27%, list

0.41248	1.61944866	0.01579444	0.05239542	0.03373914	4549	tags=29%, list
0.52769319	1.74018808	0.01588474	0.05255482	0.03384178	4819	tags=50%, list
0.29176537	1.45029161	0.01587354	0.05255482	0.03384178	4346	tags=23%, list
0.41941176	1.62973468	0.01629143	0.0538288	0.03466214	7047	tags=43%, list
0.25051331	1.30962232	0.01633793	0.05391083	0.03471496	5843	tags=32%, list
0.48934888	1.73891306	0.0164298	0.05408921	0.03482983	4647	tags=43%, list
-0.5241112	-1.5658431	0.01643547	0.05408921	0.03482983	7168	tags=47%, list
0.55541253	1.76612668	0.01662273	0.05463324	0.03518015	4367	tags=67%, list
-0.4352529	-1.4818766	0.01692437	0.05555124	0.03577128	7146	tags=42%, list
-0.6804938	-1.6179626	0.01697312	0.05563783	0.03582703	2669	tags=55%, list
0.37284303	1.51565961	0.01718545	0.05625158	0.03622224	5677	tags=37%, list
0.28968501	1.41561134	0.01720557	0.05625158	0.03622224	4502	tags=25%, list
0.4068961	1.59752556	0.0173194	0.05654942	0.03641404	5482	tags=29%, list
0.55139947	1.75336577	0.01748401	0.05693745	0.0366639	6000	tags=40%, list
0.38452789	1.53540266	0.01746281	0.05693745	0.0366639	10036	tags=59%, list
-0.3978308	-1.4261692	0.01751159	0.05695273	0.03667374	9103	tags=35%, list
0.49213558	1.66683025	0.01762298	0.05724019	0.03685884	4661	tags=32%, list
0.34431606	1.50162596	0.01770555	0.0573586	0.0369351	5320	tags=35%, list
0.34424327	1.50130849	0.01770555	0.0573586	0.0369351	9524	tags=47%, list
-0.5146956	-1.556954	0.01775149	0.05743264	0.03698277	6684	tags=37%, list
0.27389881	1.40647191	0.01793316	0.05794508	0.03731275	3405	tags=23%, list
0.37708374	1.55556686	0.01799231	0.0580608	0.03738726	6205	tags=42%, list
0.26898951	1.38230309	0.01803707	0.05812983	0.03743172	6412	tags=29%, list
0.48526132	1.72438781	0.01806369	0.0581403	0.03743846	10157	tags=67%, list
0.37029653	1.51082413	0.01810917	0.05821138	0.03748423	8852	tags=49%, list
0.55015144	1.74939721	0.01817642	0.05835218	0.03757489	5312	tags=47%, list
0.47937809	1.65093474	0.01842677	0.05899518	0.03798894	1585	tags=35%, list
0.47882699	1.64903679	0.01842677	0.05899518	0.03798894	7305	tags=55%, list
0.25654918	1.35849287	0.01844785	0.05899518	0.03798894	4307	tags=29%, list
-0.3226555	-1.2541367	0.01849267	0.05906259	0.03803235	5935	tags=25%, list
0.54738073	1.74058679	0.01852263	0.05908244	0.03804513	6846	tags=53%, list
0.47024435	1.69576935	0.01861014	0.05928555	0.03817592	6081	tags=41%, list
0.2701451	1.39588462	0.01881351	0.0598568	0.03854377	7047	tags=37%, list
0.3897418	1.5872362	0.01901472	0.0604197	0.03890624	6853	tags=41%, list
0.51668056	1.70387144	0.01922654	0.06101484	0.03928947	9952	tags=62%, list
0.31623263	1.46747653	0.01926595	0.06106203	0.03931986	7015	tags=37%, list
0.4999738	1.67024069	0.01939547	0.0613943	0.03953382	7254	tags=53%, list
-0.5644462	-1.6040865	0.01946546	0.06153757	0.03962607	4215	tags=39%, list
0.46712757	1.68452979	0.01953965	0.06169373	0.03972663	7143	tags=50%, list
0.27049655	1.38900132	0.01957836	0.0617376	0.03975488	6219	tags=29%, list
0.55977196	1.75507795	0.02034597	0.06407693	0.04126125	1480	tags=50%, list
-0.5991706	-1.6099884	0.02066565	0.06500144	0.04185657	151	tags=11%, list
0.49881421	1.66254845	0.02083511	0.06531802	0.04206043	8821	tags=72%, list
0.36619569	1.49409253	0.0208323	0.06531802	0.04206043	2992	tags=31%, list
0.28181975	1.34088873	0.02084506	0.06531802	0.04206043	9118	tags=44%, list
0.43486607	1.64296316	0.02112395	0.06594277	0.04246273	5140	tags=42%, list
0.43297819	1.6358306	0.02112395	0.06594277	0.04246273	6961	tags=50%, list
0.22003769	1.23891595	0.02109703	0.06594277	0.04246273	3930	tags=17%, list
0.49552405	1.65158234	0.02119126	0.06607	0.04254465	3568	tags=44%, list
0.4198848	1.58881274	0.0212972	0.066298	0.04269147	5386	tags=41%, list

0.38346017	1.55054444	0.02131957	0.066298	0.04269147	8215	tags=53%, list
0.24709718	1.33343604	0.02134433	0.066298	0.04269147	7624	tags=33%, list
-0.4561827	-1.4831548	0.02157519	0.06676501	0.04299219	2923	tags=33%, list
-0.4301181	-1.4600568	0.0215722	0.06676501	0.04299219	3286	tags=29%, list
0.26542381	1.35274962	0.02155924	0.06676501	0.04299219	5958	tags=31%, list
0.60514546	1.76625692	0.02168723	0.0669452	0.04310822	3568	tags=64%, list
0.37046982	1.52828274	0.02166951	0.0669452	0.04310822	5290	tags=42%, list
0.43169129	1.63096855	0.02192865	0.06752287	0.0434802	7228	tags=46%, list
-0.5962716	-1.5779082	0.02191371	0.06752287	0.0434802	4526	tags=35%, list
0.26489104	1.36124155	0.02263282	0.06960501	0.04482096	6721	tags=35%, list
0.46141667	1.66393545	0.02288922	0.07030663	0.04527276	8215	tags=41%, list
0.2415624	1.30212526	0.02292013	0.07031476	0.045278	6412	tags=28%, list
0.32417199	1.42530869	0.02325002	0.07123897	0.04587312	5586	tags=34%, list
0.49158288	1.63844642	0.02332815	0.07130274	0.04591418	1585	tags=33%, list
0.49091793	1.63623015	0.02332815	0.07130274	0.04591418	6705	tags=44%, list
0.50808906	1.67553903	0.0234598	0.07152979	0.04606039	7650	tags=56%, list
0.38295858	1.55961133	0.02348871	0.07152979	0.04606039	9053	tags=43%, list
-0.5984833	-1.534077	0.02349767	0.07152979	0.04606039	4797	tags=53%, list
0.30101025	1.38831158	0.02351743	0.07152979	0.04606039	5796	tags=39%, list
0.35958986	1.4617836	0.02357993	0.0715914	0.04610007	4567	tags=34%, list
0.23554268	1.22578787	0.02359524	0.0715914	0.04610007	5586	tags=30%, list
0.30339762	1.41435505	0.02399738	0.07272287	0.04682866	7123	tags=42%, list
0.59800892	1.74542728	0.02420571	0.07326498	0.04717774	4652	tags=55%, list
0.37616072	1.50199291	0.02426264	0.07334804	0.04723122	2505	tags=24%, list
-0.5944252	-1.5529981	0.02451925	0.07403386	0.04767284	384	tags=31%, list
-0.553292	-1.5483232	0.02455788	0.0740606	0.04769006	8420	tags=41%, list
-0.6652238	-1.5816562	0.02471736	0.07439158	0.04790319	3733	tags=82%, list
0.37446718	1.49523069	0.02473374	0.07439158	0.04790319	6081	tags=35%, list
0.29985713	1.38999608	0.02475733	0.07439158	0.04790319	4567	tags=34%, list
-0.6485011	-1.5747456	0.02489186	0.07464932	0.04806916	2397	tags=50%, list
0.28813012	1.36878413	0.02490311	0.07464932	0.04806916	7266	tags=39%, list
0.36838366	1.49564873	0.02511094	0.07518174	0.048412	4402	tags=34%, list
0.47507176	1.68817897	0.02608918	0.07801668	0.05023751	8759	tags=57%, list
-0.6729475	-1.5729288	0.02631578	0.07859983	0.05061302	5786	tags=50%, list
-0.3535621	-1.3328579	0.02647658	0.07898528	0.05086123	5180	tags=20%, list
0.31599413	1.44609738	0.02653557	0.07906648	0.05091351	3930	tags=26%, list
0.57705422	1.71229012	0.02697176	0.08027003	0.05168852	784	tags=25%, list
0.57594688	1.73631589	0.02702354	0.08032804	0.05172587	7535	tags=62%, list
0.60944599	1.75612452	0.02710593	0.08047681	0.05182167	3403	tags=40%, list
0.59558947	1.73836554	0.02728386	0.08090853	0.05209967	9003	tags=82%, list
0.23813016	1.26000105	0.02770344	0.08205496	0.05283789	7047	tags=31%, list
0.29872256	1.39256118	0.02777642	0.0820757	0.05285125	3930	tags=23%, list
0.2288087	1.22896753	0.02777398	0.0820757	0.05285125	5114	tags=28%, list
-0.3434876	-1.301133	0.02784748	0.08218807	0.05292361	7146	tags=29%, list
0.52841634	1.68028292	0.02821648	0.08317845	0.05356135	1887	tags=27%, list
-0.3543534	-1.3335873	0.02845528	0.08378313	0.05395072	3807	tags=24%, list
-0.4358506	-1.448748	0.02857143	0.08392646	0.05404302	3451	tags=24%, list
-0.4348607	-1.4454575	0.02857143	0.08392646	0.05404302	3286	tags=29%, list
0.21229221	1.20060429	0.02869938	0.08420291	0.05422103	6721	tags=30%, list
0.31487224	1.44096324	0.02882638	0.08447589	0.05439681	7523	tags=42%, list

0.5256619	1.67152423	0.0289089	0.08461806	0.05448836	5992	tags=47%, list
0.30658518	1.42270754	0.0289922	0.08476216	0.05458115	6708	tags=38%, list
0.28120284	1.33461488	0.02931961	0.08561875	0.05513274	3930	tags=21%, list
0.36602828	1.46153452	0.0294448	0.08588353	0.05530324	7181	tags=47%, list
0.40833088	1.54509356	0.02956774	0.08614113	0.05546912	5466	tags=37%, list
0.32478015	1.42806451	0.02991298	0.08704502	0.05605116	7143	tags=49%, list
0.43597538	1.6168634	0.02997566	0.08712553	0.056103	6853	tags=54%, list
0.33883992	1.43572318	0.03003267	0.08718937	0.05614411	6736	tags=41%, list
0.37496963	1.51621244	0.03011739	0.08723174	0.05617139	6782	tags=47%, list
-0.3247267	-1.2491571	0.03009027	0.08723174	0.05617139	3899	tags=20%, list
-0.517636	-1.5392952	0.03017765	0.08730464	0.05621834	5786	tags=35%, list
0.38052142	1.53547128	0.03049492	0.08812005	0.05674341	8677	tags=43%, list
0.5702284	1.71907631	0.03058349	0.08827345	0.05684219	2771	tags=46%, list
-0.4175068	-1.417247	0.03066813	0.08841518	0.05693345	7146	tags=41%, list
0.2371114	1.27813247	0.03072982	0.08849051	0.05698196	7117	tags=39%, list
0.4746796	1.58574146	0.03092765	0.08885451	0.05721635	8458	tags=71%, list
0.47418437	1.58408707	0.03092765	0.08885451	0.05721635	7305	tags=53%, list
0.37848144	1.4672615	0.03101519	0.08900321	0.0573121	7008	tags=33%, list
-0.5738786	-1.5420282	0.03128262	0.08966722	0.05773968	4408	tags=28%, list
0.6003996	1.73005727	0.0313331	0.08970856	0.0577663	5668	tags=60%, list
0.49203498	1.62259705	0.03157358	0.09029318	0.05814276	8300	tags=56%, list
0.37290778	1.51867911	0.0317229	0.09061606	0.05835067	6606	tags=46%, list
0.40451629	1.53065944	0.03204891	0.09133755	0.05881526	6806	tags=56%, list
-0.3992882	-1.4111794	0.03201708	0.09133755	0.05881526	4379	tags=19%, list
0.26811059	1.35739952	0.03211667	0.09142595	0.05887219	5456	tags=33%, list
-0.5852575	-1.5001755	0.03303685	0.09393792	0.06048972	9343	tags=73%, list
-0.4911006	-1.4761496	0.03317536	0.09411797	0.06060566	8397	tags=44%, list
0.35857974	1.43179286	0.03321366	0.09411797	0.06060566	5586	tags=41%, list
0.35806918	1.42975419	0.03321366	0.09411797	0.06060566	7292	tags=50%, list
0.29148429	1.37651839	0.03357584	0.09503605	0.06119685	3930	tags=21%, list
0.26295056	1.3385747	0.03364054	0.09511099	0.06124511	6061	tags=27%, list
0.37095064	1.51070861	0.03372116	0.09523069	0.06132218	4834	tags=35%, list
0.48556311	1.60125459	0.034043	0.09603059	0.06183726	4207	tags=31%, list
-0.455947	-1.4623564	0.03424658	0.09649545	0.0621366	5786	tags=23%, list
0.26945822	1.34898042	0.03431934	0.09659109	0.06219819	5918	tags=28%, list
0.44526288	1.60568256	0.03442666	0.09670369	0.0622707	7080	tags=50%, list
-0.6286654	-1.526579	0.03443709	0.09670369	0.0622707	4436	tags=33%, list
-0.5677445	-1.5717083	0.03447784	0.09670897	0.0622741	8064	tags=38%, list
0.40188751	1.52071232	0.03453008	0.09674644	0.06229822	5992	tags=37%, list
0.38678158	1.50294154	0.03477751	0.09722175	0.06260429	6555	tags=54%, list
0.37187647	1.50058739	0.03481695	0.09722175	0.06260429	2382	tags=29%, list
-0.4711169	-1.471741	0.03480278	0.09722175	0.06260429	5730	tags=43%, list
-0.6442184	-1.5317131	0.0353395	0.09845988	0.06340156	3172	tags=36%, list
-0.6350006	-1.5097966	0.03532609	0.09845988	0.06340156	3980	tags=55%, list
0.56128771	1.66550621	0.03543354	0.09861146	0.06349917	4715	tags=58%, list
-0.5761852	-1.5247537	0.03548796	0.09865256	0.06352564	5137	tags=35%, list
0.39791539	1.56378475	0.03553587	0.0986755	0.06354041	7228	tags=43%, list
-0.5636197	-1.529973	0.03647799	0.10113921	0.06512687	2691	tags=37%, list
-0.425973	-1.4273473	0.03650442	0.10113921	0.06512687	8223	tags=33%, list
0.39997954	1.51349272	0.03701125	0.10242936	0.06595765	7100	tags=37%, list

0.45067329	1.52640026	0.03722151	0.10289681	0.06625865	7100	tags=53%, list
0.45137459	1.60397049	0.03776889	0.10406312	0.06700967	5456	tags=57%, list
0.45142626	1.55467117	0.03776889	0.10406312	0.06700967	6980	tags=55%, list
0.45105581	1.55339537	0.03776889	0.10406312	0.06700967	3485	tags=40%, list
-0.4853333	-1.4588143	0.03791469	0.10413515	0.06705606	9039	tags=53%, list
-0.4975558	-1.4580375	0.03789731	0.10413515	0.06705606	10407	tags=52%, list
0.36890433	1.43013385	0.03796245	0.10413515	0.06705606	5650	tags=42%, list
0.35312692	1.41002	0.03792474	0.10413515	0.06705606	6081	tags=44%, list
-0.4059909	-1.4017176	0.03808487	0.10435591	0.06719821	8223	tags=31%, list
0.26711216	1.26491252	0.03820717	0.10457585	0.06733984	6061	tags=28%, list
0.45203898	1.65082478	0.03838714	0.10495297	0.06758268	9918	tags=61%, list
-0.4501541	-1.440076	0.0385051	0.10515991	0.06771594	5153	tags=26%, list
-0.5444238	-1.5235065	0.03865337	0.1054491	0.06790215	9339	tags=59%, list
-0.5600769	-1.5203558	0.03899371	0.10626044	0.0684246	6467	tags=37%, list
0.3979041	1.50563939	0.0390789	0.10626044	0.0684246	4567	tags=41%, list
0.39788737	1.50557611	0.0390789	0.10626044	0.0684246	9877	tags=63%, list
0.42431336	1.5736135	0.03922977	0.10655423	0.06861378	6205	tags=54%, list
0.32284888	1.41835759	0.03928585	0.10659019	0.06863694	8400	tags=69%, list
0.25009683	1.29475476	0.03979351	0.10784996	0.06944815	6219	tags=28%, list
0.39174887	1.53955067	0.04016179	0.10872964	0.0700146	7497	tags=47%, list
0.34238725	1.43475736	0.0402284	0.10879159	0.07005449	9629	tags=47%, list
0.40037014	1.51263443	0.04083919	0.11032345	0.07104091	4549	tags=42%, list
0.29922364	1.38854636	0.04097778	0.11057778	0.07120468	6219	tags=34%, list
0.39460368	1.49315086	0.04156008	0.1120276	0.07213827	2266	tags=26%, list
-0.5690489	-1.4866998	0.04171934	0.1123352	0.07233634	9470	tags=50%, list
0.3766031	1.47859142	0.04181551	0.11247243	0.07242471	2974	tags=26%, list
0.35990328	1.45529071	0.04187029	0.11249815	0.07244127	9118	tags=44%, list
0.31373132	1.37948258	0.04230784	0.11355115	0.07311933	6187	tags=27%, list
-0.4785159	-1.4383224	0.04265403	0.11435692	0.07363819	2295	tags=24%, list
-0.5650696	-1.4763037	0.04298357	0.11500225	0.07405374	10407	tags=69%, list
0.27360233	1.29854213	0.04298718	0.11500225	0.07405374	6772	tags=42%, list
0.35086063	1.42450471	0.04313915	0.11528486	0.07423573	6852	tags=44%, list
-0.4412012	-1.4100569	0.04342857	0.11593378	0.07465359	5242	tags=30%, list
0.4403228	1.51643185	0.04363573	0.11636194	0.07492929	6852	tags=45%, list
-0.6196835	-1.5047684	0.04370861	0.1164315	0.07497408	6493	tags=42%, list
-0.4524653	-1.4402969	0.04441913	0.11819765	0.07611137	4693	tags=22%, list
-0.5678121	-1.4554584	0.04447268	0.11821371	0.07612171	12561	tags=87%, list
-0.6487685	-1.5164136	0.04469141	0.11866834	0.07641446	5786	tags=50%, list
0.46471478	1.53250249	0.04502421	0.11929737	0.07681951	6961	tags=50%, list
0.46353431	1.52860962	0.04502421	0.11929737	0.07681951	1585	tags=31%, list
0.43592477	1.50128545	0.04510244	0.11937753	0.07687113	4291	tags=40%, list
0.28512066	1.31163331	0.04519361	0.1194917	0.07694465	3991	tags=27%, list
0.55962195	1.63338602	0.04527593	0.11958228	0.07700298	2483	tags=45%, list
-0.476459	-1.4592505	0.04572098	0.1206297	0.07767744	4299	tags=22%, list
0.54450126	1.64151631	0.04585801	0.12073517	0.07774536	1401	tags=31%, list
0.43437739	1.4959564	0.0458358	0.12073517	0.07774536	6731	tags=35%, list
0.32241896	1.3552719	0.04592232	0.12077668	0.07777209	5013	tags=38%, list
0.45171349	1.50556166	0.04617087	0.12104651	0.07794584	4991	tags=33%, list
0.4514065	1.50453846	0.04617087	0.12104651	0.07794584	4253	tags=44%, list
0.31142543	1.36934354	0.04612502	0.12104651	0.07794584	5649	tags=31%, list

-0.4920645	-1.4419459	0.04645477	0.12166259	0.07834256	4498	tags=31%, list
-0.504734	-1.457927	0.04651163	0.12168342	0.07835597	1612	tags=19%, list
0.44950063	1.50162719	0.04670856	0.12207027	0.07860507	7143	tags=53%, list
0.51956454	1.62901384	0.04687708	0.12238214	0.0788059	8080	tags=64%, list
0.3859578	1.52339433	0.04756567	0.12391979	0.07979604	9874	tags=55%, list
0.22576338	1.21274249	0.04754079	0.12391979	0.07979604	8759	tags=39%, list
-0.3801556	-1.3529958	0.0477707	0.12432375	0.08005616	8178	tags=36%, list
-0.5944638	-1.4747077	0.04792746	0.12460138	0.08023494	1771	tags=31%, list
0.5551163	1.62023526	0.04808806	0.12488841	0.08041977	6800	tags=64%, list
0.26044112	1.29273571	0.04814564	0.12490755	0.08043209	5938	tags=29%, list
0.49732428	1.58141495	0.04836157	0.12532203	0.08069899	3389	tags=33%, list
-0.4716365	-1.4267003	0.04840614	0.12532203	0.08069899	9080	tags=49%, list
-0.3587802	-1.325033	0.04902962	0.12680426	0.08165345	4846	tags=22%, list
0.54650643	1.62164581	0.04936438	0.12753747	0.08212558	7323	tags=50%, list
0.40835457	1.53645675	0.04948571	0.12764947	0.0821977	5074	tags=44%, list
0.3080868	1.36984479	0.04951034	0.12764947	0.0821977	6806	tags=36%, list
0.25247601	1.2647744	0.04997202	0.12870641	0.0828783	7047	tags=38%, list
-0.6220218	-1.4538966	0.05020353	0.12916895	0.08317615	8141	tags=50%, list
-0.6050383	-1.4692055	0.05033113	0.12922997	0.08321544	1467	tags=33%, list
-0.6043821	-1.4676121	0.05033113	0.12922997	0.08321544	5765	tags=42%, list
0.2736594	1.32029085	0.05067071	0.12996777	0.08369053	4567	tags=27%, list
0.54964798	1.60427468	0.0509002	0.13042193	0.08398298	8759	tags=64%, list
-0.4529016	-1.4148375	0.05104408	0.13065605	0.08413374	5473	tags=31%, list
0.44437454	1.48450266	0.05162516	0.1320076	0.08500405	7332	tags=65%, list
0.43609927	1.54968926	0.05170266	0.13207005	0.08504426	5312	tags=33%, list
0.27470718	1.30639513	0.05176331	0.13208936	0.08505669	7288	tags=39%, list
-0.5566259	-1.4729942	0.05196451	0.13233133	0.08521251	5975	tags=41%, list
-0.5553798	-1.4696965	0.05196451	0.13233133	0.08521251	7824	tags=47%, list
0.24878077	1.28173167	0.05236999	0.13322754	0.08578961	7368	tags=35%, list
0.29864671	1.31307996	0.05334609	0.13557207	0.08729933	7650	tags=39%, list
0.54280893	1.61067422	0.05360422	0.13608363	0.08762874	1534	tags=50%, list
-0.6425279	-1.5018271	0.05371147	0.13608363	0.08762874	6650	tags=70%, list
0.30306975	1.35520449	0.05368623	0.13608363	0.08762874	7850	tags=36%, list
0.43355077	1.54063313	0.05390274	0.13629067	0.08776206	3568	tags=38%, list
0.4333606	1.53995735	0.05390274	0.13629067	0.08776206	8683	tags=52%, list
-0.5427665	-1.4733659	0.05408805	0.13662037	0.08797437	1999	tags=47%, list
0.54154788	1.60693233	0.05420991	0.1367893	0.08808314	5122	tags=50%, list
-0.6089625	-1.4478876	0.05434783	0.13699837	0.08821777	5829	tags=55%, list
0.42934127	1.47861246	0.0546361	0.13744653	0.08850635	3568	tags=40%, list
0.42907946	1.4777108	0.0546361	0.13744653	0.08850635	7047	tags=40%, list
-0.4182955	-1.3694134	0.05487122	0.13775943	0.08870784	5724	tags=27%, list
0.22115489	1.21658284	0.05481942	0.13775943	0.08870784	5586	tags=30%, list
0.42687406	1.44579385	0.05528973	0.13867022	0.08929433	4367	tags=53%, list
-0.6173408	-1.4429553	0.05563094	0.13938547	0.0897549	1537	tags=20%, list
-0.6072915	-1.4439146	0.05570652	0.13943443	0.08978643	295	tags=27%, list
0.22805381	1.23189637	0.05576505	0.13944064	0.08979043	7749	tags=30%, list
0.35416768	1.3730042	0.0563525	0.14076809	0.09064522	2505	tags=21%, list
0.50603583	1.58659665	0.05685078	0.14187034	0.09135499	5107	tags=50%, list
-0.3997513	-1.3580279	0.05708013	0.14229997	0.09163164	2525	tags=26%, list
0.38132889	1.50512381	0.05732252	0.14276119	0.09192864	4568	tags=38%, list

0.30524766	1.34217977	0.05757659	0.14325055	0.09224376	7161	tags=49%, list
0.50504533	1.58349112	0.0581806	0.14460873	0.09311833	7979	tags=57%, list
0.48913311	1.55536827	0.05879916	0.14600031	0.09401441	4280	tags=27%, list
0.38043443	1.50159332	0.05894866	0.14622559	0.09415948	7153	tags=55%, list
0.40171493	1.51147475	0.05945939	0.14734557	0.09488067	1884	tags=28%, list
0.26812293	1.29357971	0.05963877	0.14749627	0.09497771	6505	tags=33%, list
0.27933114	1.28499994	0.05962488	0.14749627	0.09497771	5586	tags=35%, list
-0.6023663	-1.4322041	0.05978261	0.14770519	0.09511225	2314	tags=27%, list
0.30726077	1.340021	0.06039764	0.14907672	0.09599542	8094	tags=39%, list
0.34198464	1.38283563	0.06053223	0.1492011	0.09607551	3750	tags=28%, list
0.24896636	1.27274064	0.06056797	0.1492011	0.09607551	7394	tags=36%, list
0.54679115	1.57558401	0.06070632	0.14939398	0.09619971	5264	tags=60%, list
0.30468607	1.35472411	0.0611195	0.15026217	0.09675877	3930	tags=24%, list
0.37010797	1.45450317	0.06127997	0.15050795	0.09691703	7047	tags=47%, list
0.2949703	1.3193784	0.06161297	0.15117659	0.0973476	5192	tags=33%, list
0.4165148	1.50201279	0.06171304	0.15127295	0.09740964	4367	tags=27%, list
-0.5622505	-1.4139842	0.06177606	0.15127839	0.09741314	1467	tags=29%, list
0.23702692	1.24989053	0.06188798	0.15140343	0.09749366	7889	tags=31%, list
0.35962754	1.41194321	0.0620432	0.15148525	0.09754635	5367	tags=45%, list
0.2545476	1.26348243	0.06203037	0.15148525	0.09754635	7572	tags=32%, list
0.23882942	1.24752415	0.06213686	0.1515652	0.09759783	6219	tags=27%, list
-0.6135146	-1.4340122	0.0624152	0.15194619	0.09784317	2900	tags=50%, list
-0.371568	-1.3245108	0.06236786	0.15194619	0.09784317	6650	tags=24%, list
-0.5229668	-1.4477484	0.06265664	0.15223606	0.09802982	1476	tags=48%, list
-0.5224556	-1.4463334	0.06265664	0.15223606	0.09802982	4167	tags=29%, list
-0.3745775	-1.3277223	0.06276596	0.15235288	0.09810504	5084	tags=34%, list
0.54496093	1.57031019	0.06297565	0.15271287	0.09833686	6697	tags=60%, list
0.41906926	1.44323658	0.06343642	0.15368045	0.09895991	6964	tags=55%, list
0.48372272	1.53816406	0.06367004	0.15379714	0.09903505	1836	tags=20%, list
0.48326476	1.53670782	0.06367004	0.15379714	0.09903505	4979	tags=40%, list
-0.5112606	-1.4637201	0.06364749	0.15379714	0.09903505	1624	tags=21%, list
0.39900986	1.50129679	0.06406263	0.15401505	0.09917537	7047	tags=44%, list
-0.5139562	-1.4606002	0.06411837	0.15401505	0.09917537	3286	tags=43%, list
0.44247473	1.45916086	0.06416802	0.15401505	0.09917537	5904	tags=56%, list
0.44237134	1.45881992	0.06416802	0.15401505	0.09917537	5137	tags=44%, list
-0.5257878	-1.4411467	0.06390977	0.15401505	0.09917537	4404	tags=30%, list
0.3378256	1.34892249	0.06391473	0.15401505	0.09917537	6961	tags=38%, list
0.32208465	1.32868154	0.06419357	0.15401505	0.09917537	5992	tags=32%, list
0.38442344	1.45238637	0.06429076	0.15409964	0.09922984	3290	tags=31%, list
0.43514126	1.45032642	0.06478207	0.15497865	0.09979586	5148	tags=44%, list
-0.3971451	-1.3491743	0.064764	0.15497865	0.09979586	4928	tags=26%, list
0.23020178	1.24592326	0.06495388	0.1552404	0.09996442	7921	tags=31%, list
0.47980552	1.52570799	0.06506172	0.15534891	0.10003429	5992	tags=40%, list
0.41655989	1.43459456	0.06563651	0.15657107	0.10082128	6377	tags=40%, list
0.47917655	1.52370796	0.06575756	0.15670959	0.10091047	6344	tags=53%, list
0.34080126	1.37519341	0.06611699	0.15741537	0.10136495	5791	tags=43%, list
0.37270555	1.47108706	0.06626632	0.15762007	0.10149676	6147	tags=45%, list
0.32413701	1.32249153	0.06655363	0.15815227	0.10183946	7460	tags=38%, list
-0.4176637	-1.3579207	0.06666667	0.15826972	0.10191509	5050	tags=27%, list
0.50092238	1.57056424	0.06682449	0.15849317	0.10205898	6010	tags=50%, list

0.25650865	1.29554925	0.0669929	0.15874128	0.10221875	7047	tags=38%, list
0.42541474	1.51172154	0.06710323	0.15885141	0.10228966	7143	tags=57%, list
0.37046762	1.46225385	0.06789246	0.16041448	0.10329618	4567	tags=31%, list
0.31437698	1.30123225	0.06785791	0.16041448	0.10329618	7047	tags=29%, list
0.34706153	1.34545571	0.06832089	0.16127359	0.10384939	1585	tags=18%, list
0.2368714	1.23729641	0.0687208	0.16206385	0.10435826	9251	tags=45%, list
-0.5778128	-1.4030942	0.06887417	0.1621182	0.10439326	3500	tags=33%, list
-0.5761855	-1.3991427	0.06887417	0.1621182	0.10439326	7318	tags=42%, list
0.41552598	1.40735867	0.06902165	0.16231178	0.10451791	3851	tags=32%, list
0.53702381	1.54743932	0.06978365	0.16379408	0.10547241	9649	tags=80%, list
0.53579229	1.54389067	0.06978365	0.16379408	0.10547241	7008	tags=60%, list
-0.373934	-1.3215718	0.07043757	0.16517311	0.10636041	5695	tags=31%, list
0.29742988	1.32246095	0.07068673	0.1656013	0.10663614	2068	tags=22%, list
0.53409574	1.53900206	0.07091832	0.16598756	0.10688486	1481	tags=40%, list
0.5270871	1.56402293	0.07121703	0.16653005	0.10723419	5791	tags=58%, list
0.29956723	1.33603314	0.07140416	0.16681084	0.107415	5828	tags=39%, list
0.49626614	1.55596532	0.07149855	0.16687467	0.1074561	6700	tags=57%, list
0.43446744	1.43275501	0.07196738	0.16765434	0.10795816	5668	tags=38%, list
-0.3508954	-1.2768692	0.07194995	0.16765434	0.10795816	7317	tags=30%, list
0.42246758	1.40808502	0.07265606	0.16910035	0.10888929	3699	tags=33%, list
0.49252459	1.54423426	0.07283493	0.16935824	0.10905536	3164	tags=50%, list
0.42294917	1.50296009	0.07297012	0.16951416	0.10915576	9096	tags=52%, list
-0.59374	-1.411694	0.07336957	0.1702831	0.1096509	1766	tags=27%, list
-0.5008277	-1.4338512	0.07343941	0.17028635	0.109653	4405	tags=50%, list
0.53014653	1.52762237	0.07432233	0.17217314	0.11086796	5151	tags=40%, list
-0.5908625	-1.4048524	0.07472826	0.17295248	0.1113698	1467	tags=36%, list
0.47310145	1.50439005	0.07482406	0.17301326	0.11140895	7293	tags=47%, list
0.39041457	1.44789604	0.07561227	0.17467347	0.11247801	6081	tags=38%, list
-0.5531275	-1.3910411	0.07593308	0.17492731	0.11264146	7824	tags=57%, list
0.32323493	1.31399565	0.07588017	0.17492731	0.11264146	6441	tags=45%, list
0.32266185	1.31166601	0.07588017	0.17492731	0.11264146	8232	tags=47%, list
0.25457204	1.27971337	0.07612807	0.17521428	0.11282625	8998	tags=41%, list
0.35076679	1.36007652	0.07620626	0.17523215	0.11283776	4304	tags=28%, list
-0.4571244	-1.3735715	0.07629108	0.1752652	0.11285904	4764	tags=30%, list
-0.4984805	-1.4271312	0.07711138	0.17698627	0.1139673	7208	tags=42%, list
0.40578159	1.39747507	0.07738474	0.17744999	0.1142659	4280	tags=35%, list
0.41030627	1.38967988	0.07771582	0.17804507	0.1146491	8292	tags=47%, list
0.52246093	1.5502957	0.07852117	0.17972463	0.11573062	5906	tags=58%, list
0.26651035	1.25470334	0.07924814	0.18122184	0.11669472	3930	tags=21%, list
-0.466463	-1.3669233	0.0794621	0.18154427	0.11690234	9018	tags=41%, list
0.42667537	1.42537582	0.07976166	0.18206147	0.11723538	4132	tags=35%, list
-0.5863614	-1.3941506	0.08016304	0.18280995	0.11771735	6616	tags=45%, list
0.40301053	1.38793177	0.08033265	0.18302896	0.11785839	5376	tags=40%, list
0.51746129	1.51033041	0.08082535	0.18398305	0.11847275	6147	tags=55%, list
0.51361372	1.52404344	0.08095589	0.18411174	0.11855562	5631	tags=58%, list
0.28588941	1.27174324	0.08127764	0.18467468	0.11891812	7288	tags=41%, list
0.50692604	1.5282377	0.08171024	0.1854882	0.11944197	2036	tags=38%, list
0.40240591	1.38584951	0.0818066	0.18553767	0.11947382	1480	tags=35%, list
0.35942067	1.41865101	0.08252781	0.1870029	0.12041733	7035	tags=59%, list
0.36290424	1.37320254	0.08269947	0.18722137	0.12055801	8905	tags=52%, list

0.50576242	1.52472973	0.08301757	0.18775511	0.12090171	17467	tags=100%, li
0.51548	1.50454756	0.08308616	0.18775511	0.12090171	7304	tags=45%, list
0.4670806	1.48524469	0.08321532	0.1878551	0.1209661	11090	tags=87%, list
0.34065148	1.32060583	0.08328142	0.1878551	0.1209661	5476	tags=48%, list
-0.3997745	-1.3350525	0.0839779	0.18925454	0.12186724	9093	tags=37%, list
0.41569721	1.38551935	0.08416558	0.18950585	0.12202907	5032	tags=39%, list
-0.5183647	-1.4071261	0.08553459	0.19235496	0.1238637	1571	tags=21%, list
-0.4130023	-1.3329957	0.08558559	0.19235496	0.1238637	9494	tags=37%, list
-0.4268973	-1.3503153	0.08591065	0.19291129	0.12422194	4693	tags=20%, list
0.31044073	1.300887	0.08635431	0.19373266	0.12475085	5117	tags=37%, list
-0.3356954	-1.2556551	0.08655804	0.19401479	0.12493252	3415	tags=18%, list
0.37357444	1.41139789	0.0869577	0.19473515	0.12539638	6344	tags=42%, list
-0.5609108	-1.3914717	0.0880829	0.19707757	0.12690475	9318	tags=46%, list
0.5053106	1.49940561	0.08826005	0.19727132	0.12702951	1585	tags=33%, list
-0.3580511	-1.2798247	0.08832808	0.19727132	0.12702951	5340	tags=20%, list
0.49975442	1.4829188	0.08947741	0.199659	0.12856702	7717	tags=50%, list
0.42073648	1.40553604	0.08964339	0.19985013	0.12869009	6319	tags=47%, list

**core_enrichment**
COL11A1/FN1/THBS2/COL5A2/COL6A3/FBN1/THBS1/PLAU/COL3A1/COL1A2/ITGB1/ITGA2/TGM
FN1/IBSP/COL10A1/COL5A2/COL8A1/COL6A3/FBN1/THBS1/COL3A1/COL1A2/ITGB6/ITGB1/ITGA
COL11A1/MMP13/LOX/COL12A1/COL10A1/COL5A2/PLOD2/COL8A1/COL6A3/COL3A1/LOXL2/C
COL11A1/MMP13/LOX/COL12A1/COL10A1/COL5A2/COL8A1/COL6A3/COL3A1/LOXL2/COL1A2/
ITGB1/ITGA2/LAMA3/ITGAV/LAMC2/NID2/LAMA2/COL7A1/COL4A1/NID1/HSPG2/LAMA4/COL4
COL11A1/FN1/IBSP/THBS2/COL5A2/COL6A3/THBS1/COL3A1/COL1A2/ITGB6/ITGB1/ITGA2/ITGA
COL11A1/FN1/COL5A2/COL3A1/COL1A2/ITGB1/ITGA2/MET/LAMA3/COL5A1/LAMC2/COL1A1/L
FN1/VCAN/IBSP/TGFB2/COL5A2/COL6A3/COL3A1/COL1A2/ITGB6/ITGB1/ITGA2/SERPINE1/DMP
COL11A1/FN1/COL10A1/COL5A2/THBS1/COL3A1/COL1A2/ITGB1/ITGA2/LAMA3/ITGAV/COL5A1
FBN3/FN1/LOX/TGFB2/FBN1/LOXL2/ITGB6/ITGB1/ITGAV/LTBP2/LTBP1/MFAP5/MFAP3/ITGB8/I
FN1/IBSP/FBN1/THBS1/PLAU/COL1A2/ITGAV/EDIL3/COL1A1/TGFBI/COL4A1/TNC/L1CAM/ITGB3
ANKRD1/SNAI2/ADGRF4/ANGPTL4/FGFBP1/PTGS2/B3GNT5/AMIGO2/IL11/NAV3/ABHD2/BIRC3/
COL11A1/FN1/COL5A2/COL3A1/COL1A2/ITGB1/ITGA2/MET/LAMA3/COL5A1/LAMC2/COL1A1/L
COL11A1/FN1/COL12A1/COL10A1/COL5A2/COL8A1/COL6A3/COL3A1/COL1A2/ITGAV/COL5A1/E
FN1/COL5A2/THBS1/COL3A1/COL1A2/ITGB1/ITGA2/ITGAV/COL5A1/FGF2/COL1A1/TNC/ITGB3/I
FBN3/FN1/TGFB2/FBN1/ITGB6/ITGB1/ITGAV/LTBP2/LTBP1/MFAP5/MFAP3/ITGB8/ITGB3/EFEM
MMP1/COL11A1/MMP13/COL12A1/COL10A1/COL5A2/COL8A1/COL6A3/COL3A1/COL1A2/MM
PLAT/THBS2/COL5A2/COL6A3/THBS1/COL3A1/COL5A1/PDGFC/COL4A1/PDGFRB/RASA1/PIK3CA
MMP1/COL11A1/COL12A1/COL10A1/COL5A2/COL8A1/COL6A3/COL3A1/COL1A2/MET/COL5A1/
COL11A1/COL12A1/COL10A1/COL5A2/PLOD2/COL8A1/COL6A3/COL3A1/COL1A2/COL5A1/COL1
TGFB2/FBN1/COL1A2/ITGB6/ITGB1/SERPINE1/ITGAV/LTBP2/LTBP1/SKIL/ITGB8/ITGB3/ITGB5/CD
FN1/ITGB1/ITGA2/LAMA3/ITGAV/LAMC2/PTGS2/LAMA2/COL4A1/PIK3CA/PIK3CG/LAMA4/RARB
COL11A1/FN1/IBSP/MYL2/THBS2/COL5A2/COL6A3/THBS1/COL3A1/COL1A2/ITGB6/ITGB1/ITGA
ITGB1/ITGAV/MMP14/RASA1/ITGB5/ITGA5/PIK3CA/ASAP1/VCL/NCK1/PIK3R1/ETS1/RAP1B/KLF
MMP1/MMP13/FN1/FOXE1/INHBA/TGFB2/ADAM12/FBN1/SNAI2/COL1A2/SCEL/COL1A1/MMP
FBN3/MMP1/COL11A1/MMP13/FN1/COL12A1/COL10A1/COL5A2/COL8A1/COL6A3/FBN1/COL3
CASP14/KLK5/FOXE1/DKK1/GAS1/IGFBP5/WNT5A/KLK4/GJB6/KLK6/S100A4/NOTCH2/ADAMTS2
MMP1/FN1/THBS1/COL1A2/ITGB1/ITGA2/MET/SKIL/RUNX2/CAV1/TNC/ITGB3/ZEB1/ZEB2/MM
FN1/ITGB1/ITGA2/LAMA3/ITGAV/LAMC2/PTGS2/LAMA2/COL4A1/PIK3CA/LAMA4/COL4A5/RAR
INHBA/TGFB2/FBN1/COL1A2/ITGB6/ITGB1/SERPINE1/ITGAV/NOG/LTBP2/LTBP1/SKIL/ITGB8/FST
FN1/IBSP/MYL2/THBS2/COL5A2/THBS1/COL1A2/ITGB6/ITGB1/ITGA2/MET/ITGA11/LAMA3/ITGA
GUCA2A/AMY1C/PNLIPRP2/GUCY2C/SI/PNLIPRP1/CEL/AMY2B/AMY1A/AMY2A/PNLIP/CLPS/AM
HAVCR1/CLEC6A/ITGB1/ITGA2/ITGAV/AXL/CAV1/ITGB3/CAV2/ADAM17/ITGA5/PIK3CA/REL/TLR
FBN3/FN1/IBSP/ZPLD1/THBS2/POSTN/HMCN1/FBN1/THBS1/MXRA5/CCN4/ANOS1/DMP1/LAM
SEMA3C/ITGB1/MET/SEMA3D/EPHA3/SEMA3A/SEMA7A/NFAT5/UNC5D/L1CAM/RASA1/NTNG1
RPL36AL/RPL41/RPS25/RPL10/RPL5/RPS4Y1/RPS16/RPL35A/RPS23/RPL10A/RPS8/RPL11/RPS3/
RPL36AL/RPL41/RPS25/RPL10/RPL5/RPS4Y1/RPS16/RPL35A/RPS23/RPL10A/EEF1D/RPS8/RPL11
GUCA2A/AMY1C/PNLIPRP2/GUCY2C/SI/PNLIPRP1/CEL/AMY2B/AMY1A/AMY2A/PNLIP/CLPS/AM
RPL41/RPS25/RPL10/RPL5/RPS4Y1/RPS16/RPL35A/RPS23/RPL10A/RPS6KA6/RPS8/RPL11/RPS3/
RPL36AL/RPL41/RPS25/RPL10/RPL5/ATF4/RPS4Y1/RPS16/RPL35A/RPS23/RPL10A/RPS8/RPL11/
RPL36AL/RPL41/RPS25/RPL10/RPL5/SRPRB/RPS4Y1/RPS16/RPL35A/RPS23/RPL10A/RPS8/RPL11
RPL36AL/EIF3B/RPL41/RPS25/RPL10/RPL5/RPS4Y1/RPS16/RPL35A/RPS23/EIF3K/RPL10A/EIF3F/
RPL4/SCLY/RPL36AL/RPL41/RPS25/RPL10/RPL5/RPS4Y1/RPS16/RPL35A/RPS23/AHCY/RPL10A/P
RPL4/UPF3A/RPL36AL/RPL41/RPS25/RPL10/RPL5/RPS4Y1/RPS16/RPL35A/RPS23/RPL10A/RPS8/
SIGLEC6/COL3A1/COL1A2/ITGB1/COL1A1/TREM1/IGKV1D-33/SIGLEC8/IGKV2D-28/ICAM5/FCGR
FGF23/FGF19/FN1/MYL2/ITGB6/ITGB1/ITGA2/ITGA11/ITGAV/PDGFC/FGF2/F2R/ITGB8/FGF16/IT
RPL36AL/RPL41/RPS25/RPL10/KPTN/RPL5/ATF4/RPS4Y1/RPS16/ATP6V1G2/RPL35A/RPS23/MLS
RPL36AL/RPL41/POLR2E/RPS25/RPL10/CLTA/RPL5/CPSF4/NUP88/RPS4Y1/RPS16/RPL35A/RPS2
EXOSC7/EXOSC1/EXOSC6/RRP7A/RPL15/RPP38/NOP56/IMP4/RRP9/EXOSC8/ELAC2/RPL30/TBL3

RPL5/NDUFAB1/ASPG/SLC5A5/CKM/DBH/OAZ1/PSMB3/PYCR3/RPS4Y1/RPS16/SUOX/RPL35A/R
MRPS7/EEF1E1/ERAL1/EEF2/FARSA/RPL15/MRPS11/MRPL28/SEC61G/SARS2/QARS1/SSR2/RPL3
HMCN1/COL6A3/LAMA3/LAMC2/NID2/LAMA2/COL4A1/NID1/NTNG1/COL6A1/HSPG2/LAMA4/
FN1/TGFB2/SNAI2/CLDN6/WNT2/WNT5A/WNT7A/MMP2/SPARC/NRP2/COL4A1/NOTCH2/CDH2
COL11A1/FN1/COL5A2/COL3A1/COL1A2/ITGB1/ITGA2/MET/LAMA3/COL5A1/LAMC2/COL1A1/L
PSMD4/RPL4/UPF3A/RPL36AL/RPL41/RPS25/RPL10/RPL5/PSMB3/RPS4Y1/RPS16/RPL35A/RPS2
ITGB6/ITGB1/ITGA2/MET/CDH11/ITGA11/ITGAV/CDH3/ITGB8/ITGB3/PDGFRB/CDH2/ITGB5/KIT/
FN1/THBS2/SNAI2/COL3A1/EDNRA/COL5A1/COL1A1/SPARC/YAP1/TWIST1/TEAD1/GPC6
ITGB1/ARHGAP20/ARHGAP42/AMIGO2/CAV1/PIK3CA/ECT2/ARHGAP29/PREX2/IQGAP1/RASGR
INHBA/THBS2/TGFB2/THBS1/NOG/LTBP1/BMP5/BMP8A/BMPR2/DCN/GDF6/CDKN2B/BMP8B/T
FBN3/POSTN/FBN1/SERPINE1/LTBP2/LTBP1/RUNX2/FBN2/CCN2/TGFBR2/TGFBR1/ANGPT2/SM
ITGAV/ITGB3/PDGFRB/LRP1/RASA1/PIK3CA/PIK3CG/EPS8/IQGAP1/STAT1/ACTN4/DOCK4/NCK1/
MUC16/VCAN/THBS2/ADAMTS12/THBS1/OMD/KERA/ADAMTS2/LUM/NOTCH2/ADAMTS20/CH
PCDH7/ARHGAP20/ANLN/ARHGAP42/CAV1/ECT2/ARHGAP29/PREX2/IQGAP1/RASGRF2/ARHGA
ITGB1/SLITRK3/ARHGAP42/AMIGO2/CAV1/EPHA2/DSG2/ARHGAP5/TRIO/FERMT2/ARAP2/NCF2
ITGAV/ITGB3/PDGFRB/CLDN1/PIK3CA/NECTIN3/IQGAP1/PIK3R1/PDGFB/RAP1B/CTNNB1/CDH1/
MMP1/MMP13/SERPINE1/TIMP3/OSMR/HIF1A/STAT1/IRS1/IL6ST/KRAS/PIAS3/CCN1/PIK3R1/O
SNAI2/MET/NECTIN3/IQGAP1/VCL/FER/EGFR/ACTN4/TGFBR2/LEF1/TGFBR1/SNAI1/LMO7/TJP1/
MMP13/PLAT/TGFB2/COL10A1/PLAU/TIMP3/FGF2/CTSV/DDR2/CHST11/RUNX2/ADAMTS5/SER
MMP13/PLAT/TGFB2/COL10A1/PLAU/TIMP3/FGF2/CTSV/DDR2/CHST11/RUNX2/ADAMTS5/SER
MMP1/PLAU/COL1A2/DMP1/PENK/CXCL8/GJA1/HIF1A/CSF2/TH/FOSL1/NR3C1/MAF/FOSL2/CC
CAV1/GJA1/IQGAP1/ARHGAP5/CPNE8/ARL13B/ARHGAP21/DIAPH3/PAK2/ITSN1/VANGL1/ARHG
VCAN/S100A2/DKK1/SNAI2/SERPINE1/MET/MMP2/CAV1/TGFA/IGFBP3/PRDM1/HGF/EDN2/EP
COL11A1/COL12A1/COL10A1/COL5A2/COL8A1/COL6A3/COL3A1/COL1A2/COL5A1/COL1A1/COL
COL11A1/COL12A1/COL10A1/COL5A2/COL8A1/COL6A3/COL3A1/COL1A2/COL5A1/COL1A1/COL
KRT14/ITGB1/CDH11/KRT5/CLDN6/LAMA3/LAMC2/CDH3/KIRREL1/COL17A1/CDH2/CLDN1/LIM
MET/PIK3CA/HGF/F2RL2/PRKCI/NCK1/CBL/PAK2/SNAI1/PIK3R1/ETS1/RAP1B/CTNNB1/SOS1/GA
PXDN/CAV1/CAV2/PTPRR/RASA1/GJA1/ASAP1/EPS8/IQGAP1/ERRFI1/TWIST1/NEDD4/STAT1/EG
EREG/AREG/H2BC7/H3C14/MMP2/H2BC8/CAV1/CAV2/TGFA/H2BC6/GNB4/PIK3CA/H3C12/ZNF
MMP1/PLAU/COL1A2/LAMA3/MMP2/IVL/CXCL8/DCN/GJA1/FOSL1/PLAUR/FOSL2/EP300/ITGB4
TGFB2/FBN1/ITGB6/ITGB1/ITGAV/LTBP2/LTBP1/ITGB8/ITGB3/ITGB5/TGFBR2/PMEPA1/TGFB3/S
ITGB1/MET/LAMA3/LAMC2/LAMA2/COL17A1/PIK3CA/LAMA4/IL1A/PMP22/ITGA6/LAMC1/LAM
PLPP4/IGKV1D-33/IGKV2D-28/FCGR2A/FCGR3A/IGKV5-2/PIK3CA/IGLV3-25/ITPR2/IGKV2-28/NC
ITGB1/ARHGAP42/CAV1/PIK3CA/IQGAP1/EPHA2/DSG2/TRIO/NCF2/DOCK4/NCKAP1/ARHGAP21
CHIA/AMY1C/SI/AMY2B/AMY1A/AMY2A/AMY1B
DKK1/ITGB1/ITGAV/MME/PODXL/WT1/ITGB3/CDH2/CLDN1/LIMS1/TLR4/MYO1E/COL4A5/ITGA
KRT14/KRT5/COL5A1/AXL/TBXT/RRAD/IGFBP3/IL1A/YAP1/ITGA3/RUNX1/VDR/FOSL2/TOP2A/HE
RSAD2/GBP4/IFIT2/GBP6/OAS3/VCAM1/GBP1/IFIT3/NEDD4/ICAM1/FLNB/STAT1/CD44/TRIM6/
LINC02582/FN1/COL1A2/MMP2/SLC2A1/LINC00974/CDH2/CCR8/ZEB2/HIF1A/CSF2/VCAM1/TW
MMP1/FN1/COL1A2/ITGB1/ITGAV/COL1A1/TREM1/IGKV1D-33/IGKV2D-28/CAV1/L1CAM/ITGB3
NDUFS5/ATP5F1A/NDUFAB1/NDUFAF5/TACO1/SLC25A14/NDUFAF4/NDUFC2/NDUFA8/NDUFA
ITGB6/ITGB1/ITGA2/ITGA11/ITGAV/ITGB8/ITGB3/ITGB5/ITGA5/ITGA6/ITGA3/ITGA4/ITGA1/ITGA
CAV1/ARHGAP5/AKAP12/ARHGAP21/DIAPH3/SOWAHC/VANGL1/ARHGAP32/PIK3R1/RHOF/SLC
DKK1/WNT5A/SEMA3A/CTHRC1/BMP5/MEPE/RUNX2/CTSK/BMPR2/RORB/BHLHE41/SOST/BHL
MMP13/FN1/FPR2/PLAU/ITGB1/SERPINE1/ITGAV/KLK4/ITGB3/PDGFRB/LRP1/ITGB5/ITGA5/HGF
EIF3B/RPS25/RPS4Y1/RPS16/RPS23/EIF3K/EIF3F/RPS8/RPS3/FAU/RPS10/RPS15A/RPS4X/RPS14/
MMP1/A2ML1/TGM5/MMP13/PLAT/LOX/ADAM12/ADAMTS12/PLOD2/SULF1/PLAU/CD109/LO
TGFB2/ITGB1/ANOS1/BMP5/WT1/FAT4/BMPR2/SLIT2/RARB/SIX1/GLI2/GLI3/ROBO1/GREB1L/L
COL1A2/SERPINE1/SKIL/RUNX2/ITGB5/AR/LAMC1/CDKN2B/NR3C1/RUNX1/GATA3/FOXO1/VDR/
IGKV1D-33/IGKV2D-28/MS4A2/IGKV5-2/PIK3CA/IGLV3-25/IGKV2-28/IGHV2-70/IGLV7-43/IGHV3

FGF23/FGF19/FN1/FGF2/F2R/FGF16/PDGFRB/FGF11/MYLK/FGF7/RDX/PIK3CA/PIK3CG/PDGFRA/
PDGFRB/PIK3CA/BDNF/IL2RA/STAT1/EGFR/IRS1/ANGPT1/NTF3/IL6ST/FRS2/KRAS/PAG1/NTRK1/
NDUFS5/ATP5F1A/NDUFAB1/SLC25A14/NDUFC2/NDUFA8/ATP5MF/UQCRC1/ATP5PF/COX17/CO
TIMP3/FGF2/TIMP2/PIK3CA/PDGFRA/HIF1A/ANGPT1/FLT1/PDGFB/SMAD1/VEGFA/MAPK1/TEK/
PIK3CA/EPS8/IQGAP1/F2RL2/STAT1/EGFR/NCKAP1/ACTR2/DIAPH3/KRAS/MAP3K2/NRAS/PIK3R1
TGFB2/SNAI2/COL1A2/MET/COL1A1/MMP2/SEMA3A/REL/HGF/NRP1/CCN2/EGFR/TGFBR2/PLXN
PLAU/WNT2/WNT5A/WNT7A/PIK3CA/PIK3CG/WNT7B/FOSL1/WNT10A/CCNG2/IRS1/PMAIP1/LE
MMP1/MMP13/MMP20/TIMP3/MMP8/MMP2/MMP11/MMP16/MMP14/TIMP2/MMP12/MMI
KRT14/KRT5/LAMA3/LAMC2/COL17A1/ITGA6/LAMB3/DST/PLEC/ITGB4
MMP13/MMP2/MSR1/MMP14/HIF1A/MSN/STAT1/EGFR/IRS1/ROCK1/FOXO1/INHBB/MMP7/SF
ANLN/CAV1/IQGAP1/ARHGAP5/ROCK1/ARHGAP21/DIAPH3/ARHGEF12/VANGL1/ERBIN/ARHGAF
LOX/PLOD2/COL1A2/COL1A1/ADAMTS2/P4HA1/SERPINH1/PLOD1/BMP1/FZD1/P3H2/MBTPS2/I
ITGB1/MET/SEMA3A/SEMA7A/SEMA3E/NRP1/ITGA1/DPYSL3/ROCK1/PLXNA1/MYH9/MYH10/AF
EREG/AREG/MMP2/CAV1/CAV2/TGFA/GNB4/PIK3CA/GNG12/EGFR/GNGT1/KRAS/AKT3/STRN/EI
KRT14/ITGB1/CDH11/KRT5/CLDN6/LAMA3/LAMC2/CDH3/COL17A1/CDH2/CLDN1/LIMS1/CLDN1
IGKV1D-33/IGKV2D-28/MS4A2/IGKV5-2/IGLV3-25/ITPR2/IGKV2-28/ITPR3/IGHV2-70/IGLV7-43/P
ARHGAP39/RPS8/ELOB/RPL11/RPS3/RPL23/PSMB6/FAU/RPL27/RPL7/RPS10/RPL6/RPL37/RPS15
ARHGAP20/ARHGAP42/CAV1/PLEKHG4B/ECT2/ARHGAP29/PREX2/IQGAP1/RASGRF2/SH3PXD2A
IGKV1D-33/IGKV2D-28/MS4A2/IGKV5-2/IGLV3-25/IGKV2-28/IGHV2-70/IGLV7-43/KRAS/IGHV3-3
THBS2/ADAMTS12/THBS1/ADAMTS2/ADAMTS20/SPON1/ADAMTS5/THSD7B/ADAMTS3/THSD4/
FN1/FBN1/PLAU/ITGB6/ITGAV/EDIL3/ITGB8/ITGB5/VCAM1/ITGA4/PLAUR/TGFBR1/CCN1
TGFB2/SKIL/CAV1/YAP1/CCN2/TGFBR2/TGFB3/SMURF2/TGFBR1/DAB2/TAB2/RNF111/SPTBN1/C
MYL2/ITGAV/MFAP5/ITGB3/MYLK/VCL/LPP/TJP1/CREB1/ACTN1/MAPK1
INHBA/FBN1/THBS1/ITGB6/SERPINE1/NOG/LTBP1/SKIL/RUNX2/ZEB2/STAT1/TGFBR2/LEF1/TGFE
TGFB2/KIT/REL/HIF1A/ANGPT1/NOTCH3/JAG1/TGFB3/KITLG/EPHB2/TGFBR1/PDGFB/SMAD1/SM
LOX/LOXL2/GREM1/BMPR2/BMP1/TGFBR2/TGFBR1/SMAD3/LOXL4/LOXL1/MAPK14/BMPR1A/S
RPS4Y1/SNRPD1/RPS16/RPS23/SNRPD2/SNRPF/GEMIN7/RPS8/RPS3/FAU/RPS10/RPS15A/RPS4X
LAMA3/LAMC2/LAMA2/ITGA6/LAMC1/LAMB1/LAMB3/ITGB4/LAMB2
MYL2/PIK3CA/EPS8/IQGAP1/F2RL2/ENAH/ACTR2/DIAPH3/CBL/PAK2/PIK3R1/LIMK1/ACTR3/CTN
CAV1/RASAL2/FAM83B/CCDC88A/EPHA2/ARHGAP5/KIF14/PTPN13/PLXNA1/DST/FRS2/KIDINS22
INHBA/THBS1/ITGB6/SERPINE1/NOG/LTBP1/SKIL/RUNX2/ZEB2/STAT1/TGFBR2/LEF1/TGFBR1/SN
FN1/ITGB1/FGF2/MMP2/RASA1/ITGA5/PIK3CA/FOXO1/NCK1/ANGPT1/PIK3R1/ANGPT2/ETS1/EL
ITGAV/MYOF/CAV1/ITGB3/PIK3CA/IQGAP1/VCL/NEDD4/ROCK1/NCK1/CBL/PAK2/PRKAA2/FLT1/
FN1/COL1A2/ITGB1/ITGA2/COL1A1/ITGA5/PIK3CA/ITGA4/ITGA1/PIK3R1/CREB1/SOS1/SHC1/MA
LOX/LOXL2/COL1A2/COL1A1/PXDN/COL4A1/LOXL3/COL4A5/COL4A4/COL4A2/BMP1/PCOLCE/TI
ITGB6/ITGB1/ITGA2/ITGA11/ITGAV/ITGB8/CAV1/ITGB3/CAV2/ITGB5/ITGA5/ITGA6/ITGA3/VCL/IT
CASP14/FGF23/CYP24A1/S100A2/TGFB2/GXYLT2/TIMP3/IGFBP5/IL1RL1/TNFSF4/TREM1/ADRA1
CAV1/GJA1/ARHGAP5/TRIO/CPNE8/ARL13B/ARHGAP21/DIAPH3/PAK2/VANGL1/ARHGAP32/PIK3
NDUFS5/ATP5F1A/NDUFAB1/NDUFC2/ATP6V1G2/NDUFA8/TCIRG1/ATP5MF/UQCRC1/ATP5PF/C
ITGB1/SERPINE1/PTGS2/CXCL8/CLDN1/KIT/TCF4/PIK3CA/BIRC3/SERPINB2/EGFR/IRS1/CD44/ROC
LRP1/TCF4/SFRP4/DKK3/FZD1/LEF1/FZD7/CSNK1A1/CTNNB1/GSK3B/APC/MBOAT1
FN1/LAMA3/PTGS2/COL4A1/CDH3/CXCL8/CRP
FN1/THBS2/COL5A2/COL6A3/THBS1/COL3A1/COL1A2/ITGB6/ITGA11/COL5A1/COL4A1/MIR548A
NDUFS5/NDUFAB1/NDUFAF5/TACO1/NDUFAF4/NDUFC2/NDUFA8/NDUFAF6/UQCRC1/COX7C/N
LAMA3/LAMC2/LAMA2/ITGA6/LAMC1/LAMB1/IRS1/LAMB3/PIK3R1/SOS1/GAB1/ITGB4/LAMB2/
COL1A2/LAMA3/LAMC2/COL1A1/COL7A1/COL4A1/COL4A5/COL4A4/COL4A2/BMP1/LAMB3
FBXO32/FOXO1/CSNK1A1/BCL6/RALB/YWHAZ/SOD2/EP300/YWHAG/FOXO3/CTNNB1/RALA/CDI
TGFB2/TGFA/PIK3CA/PIK3CG/STAT1/CDK6/EGFR/TGFBR2/TGFB3/KRAS/AKT3/TGFBR1/RALB/PIK3
VCAN/OMD/KERA/LUM/CHST3/DCN/CHSY1/HSPG2/PRELP/SLC26A2/B4GALT1/GPC6/AGRN/BGN
TGFB2/PIK3CA/PIK3CG/CDK6/RUNX1/TGFBR2/TGFB3/KRAS/AKT3/CBL/TGFBR1/NRAS/PIK3R1/SO

TGFB2/SKIL/TGFBR2/TGFB3/TGFBR1/EP300/SMAD3/SMAD7/CDH1/APC/MAP2K1/MAP3K7/SMA

IGKV1D-33/IGKV2D-28/FCGR3A/IGKV5-2/IGLV3-25/IGKV2-28/NCK1/NCKAP1/IGHV2-70/IGLV7-43

TGFB2/MET/WNT5A/TGFA/HGF/EGFR/TGFBR2/TGFB3/KRAS/TGFBR1/NRAS/CTNNB1/MFGE8/SM

PDGFC/PDGFRB/TGFA/AR/PIK3CA/PIK3CG/PDGFRA/CREB5/EGFR/FOXO1/LEF1/KRAS/AKT3/NRAS

COL3A1/COL1A2/SCGB3A2/COL1A1/IGKV1D-33/SPARC/CD163/COL4A1/MSR1/IGKV2D-28/LRP1/

PCDH7/ANLN/CAV1/ECT2/ARHGAP5/ROCK1/ARHGAP21/DIAPH3/SOWAHC/ARHGEF12/VANGL1/

GLDC/AMT/DAO/ALAS2/PHGDH/PSAT1/BHMT/SARDH/GLYCTK/PIPOX/GATM/AGXT2/CBS/GAMT

PIK3CA/F2RL2/IRS1/NPY4R/FOXO1/CYTH3/AKT3/CBL/PIK3R1/YWHAZ/CAP1/YWHAG/PTEN/SOS1

ITGB1/MET/PDGFRB/PIK3CA/HGF/STAT1/ITGA1/EGFR/EIF2AK2/PIK3R1/PDGFB/CSF1R/SOS1/JAK

CAV1/ARHGAP5/CPNE8/PLXNA1/AKAP12/ARHGAP21/DIAPH3/VANGL1/ARHGAP32/PIK3R1/VRK2

F11/TPST2/F9/F12/KLKB1/GP1BA/F2/GP9

PIK3CA/PIK3CG/EGFR/GRIP1/IL6ST/PIK3R1/EP300/SOS1/SHC1/MAPK1/STAT3/ESR1/MAP2K1

FN1/SNAI2/RUNX2/TNC/CDH2/CDH6/SNAI1/SMAD3/CDH1/MAPK1/VIM

WNT5A/CTHRC1/FZD6/ROCK1/FZD7/CSNK1A1/TAB2/FLNA/ROR2/YES1/CDC42/NFATC2/DVL3/D

FGF23/FGF19/FGF2/WNT2/WNT5A/WNT7A/NOTCH2/FGF16/FGF7/KIT/PIK3CA/FZD6/WNT7B/SF

TLR4/OAS3/STAT1/TLR7/TLR3/TREML4/EIF2AK2/OAS2/TLR2/DDX58/IFIH1/ACE2/JAK1/STAT2/TR

TGFB2/MET/SLC2A1/TGFA/PIK3CA/PIK3CG/HIF1A/HGF/TGFB3/KRAS/AKT3/PAK2/NRAS/PIK3R1/

FGF23/FGF19/MET/PDGFC/FGF2/FGF16/PDGFRB/FGF11/MITF/FGF7/PIK3CA/PIK3CG/PDGFRA/H

MMP1/FN1/COL1A2/ITGB1/FGF2/PTGS2/MMP2/ANXA1/CXCL8/F13A1/ZEB1/HIF1A/HGF/IL1A/V

INHBA/HMGA2/DKK1/ZFHX4/NOG/ADAM19/BMPR2/TCF4/CCDC88A/TWSG1/TET1/ZNF281/YAP

PIK3CA/IQGAP1/ENAH/NCKAP1/PIK3R1/TJP1/RAP1B/CTNND1/CTNNB1/KLHL20/CDH1/WASF2/C

MMP1/PTGS2/MYOF/PODXL/CXCL8/HIF1A/BIRC3/COL4A2/CD44/LEF1/TGFBR1/SOS1/JAK1/SMA

PIK3CA/CSF2/STAT1/KRAS/NRAS/PIK3R1/YWHAZ/PIM1/OSM/SOS1/CSF2RA/SHC1/MAPK1/JAK2/

MET/PDGFRB/PIK3CA/PIK3CG/PDGFRA/ERRFI1/CDKN2B/CDK6/EGFR/IRS1/PRKCI/FOXO1/KRAS/

COL3A1/COL1A2/SCGB3A2/COL1A1/COL4A1/MSR1/COL4A2/COLEC12

RASA1/PIK3CA/DOK6/IRS1/NCK1/FRS2/GDNF/PIK3R1/CREB1/SOS1/GAB1/DOK5/SHC1/MAPK1/F

ITGB1/ITGA2/ITGA11/ITGAV/ITGA5/PIK3CA/PIK3CG/ITGA6/ITGA3/STAT1/ITGA4/ITGA1/SSH1/FO

OAS3/STAT1/TLR7/EIF2AK2/OAS2/DDX58/IFIH1/JAK1/STAT2/TRAF6/OAS1/MAPK14/MYD88/MA

PDGFRB/RASA1/PIK3CA/PDGFRA/STAT1/NCK1/KRAS/NRAS/PIK3R1/PDGFB/SOS1/STAT3/PIK3CB/

COL1A2/SERPINE1/SKIL/CDKN2B/STAT1/WWTR1/SMURF2/CCNK/EP300/RNF111/USP9X/SP1/SM

VCAN/IGF2BP1/DKK1/SNAI2/TBXT/MMP2/CXCL8/MITF/MYOG/AR/TCF4/MDFIC/ADCY7/LEF1/NC

PLPP4/IGKV1D-33/IGKV2D-28/FCGR2A/FCGR3A/IGKV5-2/PIK3CA/IGLV3-25/ITPR2/IGKV2-28/ITPI

FN1/ITGB1/MMP2/RASA1/PIK3CA/YAP1/EGFR/FOXO1/NCK1/ANGPT1/KRAS/NRAS/PIK3R1/ANGI

NDUFS5/ATP5F1A/NDUFAB1/NDUFAF5/TACO1/PDK2/SLC25A14/SUCLG1/FH/NDUFAF4/NDUFC2

FGF23/EREG/FGF19/AREG/IL1RAP/MET/FGF2/IL1RL1/FGF16/PDGFRB/TGFA/FGF7/KIT/PIK3CA/PI

H2BW1/H2BC7/H3C14/H2AC13/H2BC8/FCGR2A/H2BC18/FCGR3A/H2BC6/H2BW2/H3C12/H2AC

SEC23A/SEC24A/SEC24C/HLA-G/PIK3R4/SEC24D/SEC31A/SEC24B/ERAP1/ERAP2/CANX/TAP1/HS

MMP1/PTGS2/MMP2/CXCL8/REL/CSF2/IL1A/VCAM1/BIRC3/ICAM1/MMP3/BIRC2/VEGFA/TRAF6

GBP4/GBP6/OAS3/VCAM1/GBP1/ICAM1/STAT1/CD44/TRIM6/GBP5/OAS2/TRIM17/HLA-G/JAK1/

ANLN/CDK6/PRKDC/WEE1/TOP2A/NPAT/E2F3/MCM4/RB1/CDK2/PLK4/SMC2/CCDC6/CCNE2/SM

FN1/TGFA/MYLK/PIK3CG/FZD6/EGFR/FZD1/MYL1/NCK1/ACTR2/FZD7/NEFH/CBL/ACTR3/CTNNB1

PLAU/ITGAV/MMP2/ITGB3/PIK3CA/CD44/PIK3R1/SPP1/ROCK2/MAPK1/MAP3K1/CDC42/ILK/PIP

REL/LEPR/STAT1/IRS1/ROCK1/FOXO1/PRKAA2/PIK3R1/IL1RN/KPNA4/PTEN/CREB1/SOS1/JAK1/R

VCAN/OMD/KERA/CHST11/CHSY3/LUM/DSE/CEMIP/CHST3/CSGALNACT2/DCN/CHSY1/HSPG2/D

CTSV/TICAM2/CTSK/TLR8/TLR4/IRAK3/IRAK2/BIRC3/SFTPA1/EEA1/TLR7/NOD2/RIPK2/MEF2A/TL

VCAN/TLR4/IRAK2/SFTPA1/TLR3/TLR1/BGN/TLR2

FGF23/EREG/FGF19/AREG/MET/FGF2/FGF16/PDGFRB/TGFA/FGF7/KIT/PIK3CA/PDGFRA/HGF/FG

TGFB2/ITGAV/ATP8B3/AXL/ITGB3/FCN1/C5AR2/PLSCR4/C1S/COLEC12/ATP10A/PRKCI/C5AR1/CI

TGFB2/CXCL8/TICAM2/PIK3CA/HSPG2/TLR4/CREB5/STAT1/TGFBR2/EGR2/TLR3/TGFB3/KRAS/AK

FN1/ITGB1/ITGA2/LAMA3/MMP2/CXCL8/CAV2/RASA1/ITGA5/CSF2/EPHB2/NF1/SDCBP/MAPK1/

FN1/PIK3CA/PPFIBP1/MSN/VCL/HIP1/TPM4/IRS1/MYH9/FRS2/STRN/LMO7/PIK3R1/RNF213/GC(
FN1/THBS1/COL3A1/COL1A2/LAMC2/COL1A1/IL2RA/LAMC1/LAMB1/CD80/CD86/IL5RA
ITGB1/PIK3CA/EGFR/PIK3R1/FOXO3/PTEN/SOS1/SHC1/MAPK1/ILK
MYL2/TGFB2/NOG/BMP5/BMPR2/FZD1/TGFBR2/ACVR1/TGFB3/TGFBR1/SMAD1/CTNNB1/ATF2,
MUC16/THBS2/ADAMTS12/THBS1/ADAMTS2/NOTCH2/ADAMTS20/SPON1/ADAMTS5/THSD7B/I
MUC16/THBS2/ADAMTS12/THBS1/GALNT9/GALNT15/ADAMTS2/B3GNT5/ADAMTS20/POMK/SF
SPOCK1/PTGIS/SERPINE1/KLF7/HIF1A/NRIP1/TWIST1/SFRP4/RETN/NR3C1/KLF6/RORA/PLIN2/ST
IL1RAP/TICAM2/IL1R1/PIK3CA/IL1A/IRAK3/PRKCI/PIK3R1/IL1RN/TAB2/ERC1/TRAF6/MYD88/MA
TGFB2/ITGB1/PTGS2/FCGR2A/FCGR3A/TLR4/IL1A/STAT1/ITGA4/NCF2/TGFB3/TLR2/FCGR3B/ITG
ITGAV/AXL/NRP2/CAV1/ITGB3/RASA1/PIK3CA/ITPR2/NRP1/NCF2/ROCK1/NCK1/ITPR3/NCKAP1/
IGKV1D-33/IGKV2D-28/FCGR2A/FCGR3A/IGKV5-2/IGLV3-25/IGKV2-28/IGHV2-70/IGLV7-43/IGHV
TGFB2/CDKN2B/CDK6/TGFB3/SMAD3/RB1/GSK3B/CDK2/ATR/CDK1/CCNE1/CDKN2A/ABL1/CCNI
PDGFRB/RASA1/STAT1/PIK3R1/PDGFB/SOS1/JAK1/SHC1/MAPK1/MAP3K1/STAT3/CDC42/WASL/
ACADVL/INS/TTR/CPT1B/UCP2/DLK1/APOA1/HNF1A/BDH1/ALDOB/FOXA3/PDX1/GCK/KCNJ11/T
CYP24A1/SLC2A3/PIK3CA/LDHA/CD80/CD86/VDR/PRKAA2/GSK3B/MTOR/HLA-DRA
PDGFRB/RASA1/PIK3CA/STAT1/EIF2AK2/PIK3R1/SOS1/JAK1/SHC1/JAK2/MAP3K1/STAT3/MAP2K
FGF23/EREG/FGF19/AREG/MET/FGF2/FGF16/PDGFRB/TGFA/FGF7/KIT/PIK3CA/PDGFRA/HGF/FG
CHGA/LEAP2/ELANE/BPIFB1/PRTN3/HTN3/PGLYRP2/CLU/DEFB134/BPIFA1/PLA2G2A/PRSS3/ITLI
VCAN/ITGB1/CLDN6/ITGAV/NCAM2/CNTNAP2/CDH3/ITGB8/L1CAM/CDH2/CLDN1/CLDN11/NEC
OAS3/NEDD4/FLNB/STAT1/EIF2AK2/OAS2/DDX58/IFIT1/KPNA4/NUP160/JAK1/NUP153/EIF4G3/
MYL2/ANLN/RDX/MSN/AFAP1/KIF23/SEPTIN2/EZR/RACGAP1
PIK3CA/FMN1/EGFR/PIK3R1/CTNND1/CTNNB1/CDH1/CTNNA1/AJUBA/PIP5K1A/AKT2/RHOA/JUI
ITGB1/MET/RASA1/PIK3CA/HGF/ITGA1/RAP1B/PTEN/SOS1/GAB1/DOCK1/MAPK1/STAT3/PTPN1
ITGB1/MET/RASA1/PIK3CA/PIK3CG/HGF/PIK3R1/RAP1B/PTEN/SOS1/GAB1/DOCK1/MAPK1/STAT
IL1RAP/IL1R1/PIK3CA/PIK3CG/IL1A/IRAK3/IRAK2/BIRC3/ENDOD1/PPP3CA/AKT3/PPP3R2/TNFRSI
PIK3CA/TRPC3/KRAS/NTRK1/NRAS/PIK3R1/YWHAZ/YWHAG/FOXO3/CREB1/SOS1/GAB1/GSK3B/
STAG1/KIF20A/KIF23/STAG2/RAD21/PDS5B/SMC3/SMC1A/NIPBL/PDS5A/WAPL
CDH11/MMP8/MMP2/PIK3CA/PIK3CG/BDNF/EPHB6/F2RL2/TWIST1/TRIO/AKT3/PAK2/EPHB2/PI
CDH11/MMP8/MMP2/PIK3CA/PIK3CG/BDNF/EPHB6/F2RL2/TWIST1/TRIO/AKT3/KIDINS220/PAK
TGFB2/PLOD2/SLC2A1/PDGFRB/SSPN/ZEB1/PDGFRA/HIF1A/BHLHE41/LDHA/EGFR/HK2/CDH13/
NRP2/CAV1/RASA1/PIK3CA/HIF1A/NRP1/CD2AP/NCK1/CBL/FLT1/PIK3R1/VEGFA/HSP90AA1/MA
NFAT5/FCGR3A/ULBP3/KIR2DL3/PIK3CA/PIK3CG/MICB/CSF2/KIR3DL2/KIR2DL1/ICAM1/RAET1E/
AR/TCF4/LATS2/NRIP1/HIP1/CDK6/PRKDC/PIAS3/NCOA2/CTDSP2/CTNNB1/TMF1/MED1/SVIL/KI
IGKV1D-33/IGKV2D-28/CRP/IGKV5-2/IGLV3-25/FCN1/C1S/IGKV2-28/IGHV2-70/IGLV7-43/IGHV3-
GAS1/MMP2/TGFA/LAMA4/CENPF/EP300/NEK2/SP1/RB1/NFATC3/BRCA2/CDK2/ESR1/MAP2K1,
CAV1/RASAL2/FAM83B/TMOD3/CCDC88A/EPHA2/ARHGAP5/RND3/PTPN13/ROCK1/DST/PICALN
MS4A2/PIK3CA/PIK3CG/PPP3CA/PAK2/PIK3R1/FCER1G/SOS1/FCER1A/NFATC3/SHC1/MAPK1/M
FGF23/FGF19/FGF2/WNT2/WNT5A/NOG/WNT7A/FGF16/PDGFRB/FGF11/BMPR2/FGF7/PDGFRA
ATP5PD/UBE2J2/NDUFA4/NDUFS5/ATP5F1A/NDUFAB1/NDUFC2/NDUFA8/UQCRC1/ATP5PF/UQ
NRK/CAV1/ADAM17/BIRC3/MAP4K5/STAT1/PRKCI/MAP4K4/TAB2/TNIK/TNFAIP3/MAP4K3/BIRC
EPYC/VCAN/SPOCK1/OMD/KERA/ASPN/HAPLN1/LUM/DCN/HSPG2/PRELP/PODNL1/BGN/PODN/
ITGB1/ITGA2/ITGA11/ITGAV/ASAP2/ITGA5/IL2RA/ITGA6/ITGA3/ITGA4/ITGA1/SPAG9/CTNND1/E
ATP5PD/NDUFA4/NDUFS5/ATP5F1A/NDUFAB1/NDUFC2/NDUFA8/ATP5MF/ATP5PF/NDUFV3/NI
FGF23/FGF19/PLAU/MET/FGF2/RUNX2/CDH2/PIK3CA/HGF/STAT1/PLAUR/SSH1/FRS2/CBL/PIK3F
DKK1/COL1A2/ITGB1/WNT5A/PDGFRB/ITGA6/CDKN2B/ZFP36L1/SLC11A1/EP300/LGALS1/FOXO
TGFB2/IL1RAP/IL1R1/IL1A/IRAK3/IRAK2/TGFB3/IL1RN/IFNA1/TRAF6/MAP3K1/MAPK14/MYD88/
IL1A/STAT1/JAK1/STAT2/HMOX1/STAT3
IQGAP1/EPHA2/TPM4/NCK1/DST/PEAK1/PAK2/VANGL1/PARD6B/PIK3R1/MYO9A/SPTBN1/USP9
MYL2/CDH2/GJA1/PIK3CA/FER/ROCK1/PIK3R1/CTNND1/CTNNB1/CTNNA1/CDC42/PTPN11/PTPN
DSG3/TICAM2/UNC5B/UNC5A/TLR4/IL1A/BIRC3/DSG2/UACA/PMAIP1/ROCK1/CLSPN/AKT3/PAK

E2F7/ECT2/KIF20B/CENPF/TOP2A/MCM4/ESM1/ACTL6A/APC/SMOC2/NUP107/INO80D/TPX2/LI
TREM1/CLEC5A/PIK3CA/CD300E/SIRPB1/KRAS/NRAS/PIK3R1/KIR2DS4/SOS1/LCP2/KLRD1/CD30(
CRP/MITF/PIK3CA/STAT1/FOXO1/IL6ST/PIAS3/PIK3R1/PTPRE/SOS1/JAK1/FGG/GAB1/A2M/TNFS
MYL2/ITGB1/PIK3CA/PIK3CG/ARHGAP5/ROCK1/PIK3R1/SHC1/MAPK1/DIAPH1/MAP2K1/TLN1
H2BC7/H2BC8/H2BC6/H2BC14/H2BC4/H2BU1/WEE1/H4C8/YWHAZ/H2BC21/NBN/YWHAG/H2B(
DPYD/LOXL3/NCF2/MAF/SAMSN1/CD84/LHFPL2/TGFBR1/PPP1R18/ITGAM/C1QC/SPP1/ITGB2/E
IGKV1D-33/IGKV2D-28/MS4A2/IGKV5-2/PIK3CA/IGLV3-25/ITPR2/IGKV2-28/ITPR3/IGHV2-70/IGL
FN1/AREG/ITGB1/COL1A1/ITGA1/EGFR/KRAS/AKT3/NRAS/SOS1/MAPK1/MAPK14/MAP2K1/TLN
DKK1/PYGO1/FZD6/LEF1/CSNK1A1/SALL4/CTNNB1/ZBTB16/GSK3B/APC/PPARD/RUNX1T1/LRP6,
RIPK2/PTPN13/PRKDC/PAK2/RB1/MAP3K1/FAS/LMNB1/MAP3K7/CASP8/CFLAR/SPTAN1/MAPK8
TGFB2/PTGS2/REL/TGFBR2/PMAIP1/LEF1/TGFB3/KRAS/AKT3/TGFBR1/CSNK1A1/RALB/POLK/CTI
GNG13/KCNA5/GNG2/KCNA1/KCNMB2/KCNN1/HCN3/KCNMA1/KCNH5/GABBR2/KCNH8/KCNJ4,
IGKV1D-33/IGKV2D-28/FCGR2A/FCGR3A/IGKV5-2/IGLV3-25/ITPR2/IGKV2-28/ADCY7/ITPR3/IGH\
ALDH2/SRM/GOT1/CKM/PYCR3/PYCR2/ASL/ACY1/ODC1/DAO/NOS1/AGMAT/ARG1/SAT2/CKMT
TGFB2/PIK3CA/PIK3CG/TGFBR2/LEF1/TGFB3/KRAS/AKT3/TGFBR1/PIK3R1/CTNNB1/SMAD3/GSK
FGF23/FGF19/HSPA6/TGFB2/FGF2/FGF16/PDGFRB/FGF11/PTPRR/IL1R1/RASA1/FGF7/BDNF/IL1/
MMP13/ITGBL1/COL1A1/RUNX2/AR/ITGA5/ZNF521/YAP1/GLI2/TWIST1/NR3C1/GLI3/STAT1/WV
GAS1/WNT2/WNT5A/WNT7A/HHIP/BMP5/RAB23/BMP8A/WNT7B/GLI2/GLI3/WNT10A/BMP8B/
POSTN/WNT2/TNC/PIK3CG/VCAM1/LEF1/JAG1
FOXL2/MITF/AR/TFAP2C/NRIP1/NR3C1/RORA/PHC3/STAG1/VDR/H4C8/TOP2A/PIAS3/NCOA2/EI
LIMS1/ZEB1/IQGAP1/SNAI1/PPP1R14C/TNS1/PARVA/CTNNB1/CREB1/ACTN1/CKAP5/PPP1R12A/
NDUFS5/NDUFAB1/NDUFAF5/NDUFAF4/NDUFC2/NDUFA8/NDUFAF6/NDUFV3/NDUFS3/NDUFC1
CASP14/KRT14/KRT81/LIPK/TGM5/KLK5/KRT5/LIPN/IVL/KRT6A/DSG3/SPRR2E/KRT16/KRT7/RPTI
RUNX2/CAV1/AR/EGFR/ROCK1/FOXO1/PIAS3/NCOA2/PIK3R1/EP300/CTNNB1/PTEN/CREB1/ROC
IBSP/ITGA2/RUNX2/IL11/ICAM1/STAT1/IL6ST/PIAS3/PIK3R1/CREB1/JAK1/ATF1/YES1/MAPK1/JA
UQCRC1/CACNG1/UQCRHL/COX7C/CACNA2D3/CYC1/UQCRB/UQCR10/COX7A2/COX7B/COX6C/(
H2BC7/H2AC13/H2BC8/H2BC18/USP37/H2BC6/AR/HIF1A/USP26/USP34/H2AC7/BIRC3/H2BC14/
STAG1/STAG2/RAD21/PDS5B/SMC3/SMC1A/NIPBL/PDS5A/WAPL
LATS2/YAP1/WWTR1/STK3/AMOTL1/TJP1/LATS1/AMOTL2/SAV1/MOB1A
TICAM2/TLR4/IRAK2/EEA1/TLR7/NOD2/RIPK2/MEF2A/IKBIP/TAB2/DUSP7/CREB1/PIK3R4/APP/A
MMP2/FGF7/PDGFRA/EGFR/TNFSF10/ATF2/RB1/NFE2L2/HSP90AA1/MCL1/TRAF6/BCL2L11/FAS
FN1/THBS2/THBS1/ITGB1/VCAM1/ITGA4/YWHAZ/SPP1/DOCK1/PRKAR1A/ARF6/TLN1/ABI1/PRK.
ITGB6/ITGB1/ITGA2/ITGA11/ITGAV/LAMA2/ITGB8/ITGB3/CDH2/ITGB5/GJA1/ITGA5/SGCD/ITGA(
GAS1/HHIP/EVC/GLI2/GLI3/GLI1/GPR161/SMURF2/CSNK1A1/SPOPL/BOC/SMO/EVC2/LRP2/CDO
SKIL/LATS2/TEAD1/STK3/NF1/LATS1/MERTK/SKI/SATB2/TEAD3/TEAD2/SIN3A/PRMT5
MMP1/PTGS2/PIK3CA/PIK3CG/EGFR/PTGER3/KRAS/AKT3/NRAS/MAPK1/PIK3CB/ESR1/PTGER2
MET/PIK3CA/F2RL2/CREB5/IRS1/CYTH3/AKT3/PAK2/YWHAZ/YWHAG/FOXO3/PTEN/CREB1/SOS1
VCAN/CHST11/CHSY3/DSE/CHST3/CSGALNACT2/DCN/CHSY1/HSPG2/DSEL/XYLT1/GPC6/ARSB/A(
EREG/ADAM12/AREG/ADAM17/TGFA/PIK3CA/ADAM10/EGFR/KRAS/PTPN12/CBL/PAG1/EPGN/N
RASA1/PIK3CA/PIK3CG/IRS1/PIK3R1/SOS1/SHC1/IGF1R/PTPN11/MAP2K1/ELK1/IGF1/MAPK8
RASA1/PIK3CA/EFNA5/MAP4K4/ROCK1/NCK1/KRAS/ITSN1/EPHB2/EFNB2/NRAS/PIK3R1/RAP1B/
DSG3/TICAM2/UNC5B/UNC5A/TLR4/DSG2/UACA/PMAIP1/ROCK1/CLSPN/AKT3/PAK2/TNFRSF10
IL1RAP/IL1R1/REL/IL1A/IRAK3/IRAK2/MAP3K2/PIK3R1/TAB2/ATF2/PELI1/MAPK1/TRAF6/MAP3K
HSPA6/MET/RAB31/F2R/ASAP2/KIT/PSD2/PDGFRA/PSD3/ASAP1/CXCR2/IL2RA/NEDD4/EEA1/EG
MMAB/CD320/AMN/PRSS3/PRSS1/CTRB1/CTRB2
MET/ROCK1/MYH9/MYH10/ARHGEF12/LIMK1/ROCK2
CXCL8/TICAM2/CTSK/PIK3CA/PIK3CG/TLR8/TLR4/CXCL9/STAT1/TLR7/CD80/CD86/TLR3/TLR1/AK
COL1A1/RUNX2/AR/ZNF521/YAP1/GLI2/GLI3/WWTR1/MAF/SMAD1/RB1/YES1/MAPK1/SATB2/C
SERPINE1/HIF1A/ARNTL2/BHLHE41/NRIP1/NR3C1/RORA/NAMPT/BHLHE40/CHD9/NCOA2/EP30(
COL1A1/RUNX2/AR/ZNF521/YAP1/GLI3/WWTR1/MAF/RB1/YES1/MAPK1/SATB2/CBFB

SLC2A1/COL4A1/SLC2A3/COL4A5/COL4A4/COL4A2
IGKV1D-33/IGKV2D-28/CRP/IGKV5-2/IGLV3-25/FCN1/C1S/IGKV2-28/IGHV2-70/IGLV7-43/IGHV3-
MET/AXL/PDGFC/FGF2/PDGFRB/TGFA/PIK3CA/PDGFRA/HGF/EGFR/KRAS/AKT3/NRAS/PIK3R1/PI
HSPA6/PIK3CA/TLR4/IL1A/OAS3/IL2RA/MSN/STAT1/CDK6/TLR7/HSPA1A/CSNK2A3/EIF2AK2/OAS
ITGB6/ITGB1/ITGA2/ITGA11/ITGAV/LAMA2/ITGB8/ITGB3/CDH2/ITGB5/GJA1/ITGA5/SGCD/ITGA
NOX4/PIK3CA/KRAS/NRAS/PIK3R1/PIM1/SPTBN1/SOS1/TRIP11/GOLGB1/ETV6/GAB2/MYO18A
PIK3CA/IL5RA/PIK3R1/PIM1/SDCBP/JAK2/PTPN11
STAG1/STAG2/RAD21/PDS5B/SMC3/SMC1A/PDS5A/WAPL/ESCO2
C1QBP/PROC/F11/F9/KNG1/F12/SERPINC1/KLKB1/GP1BA/F2/GP9
GBA3/UGT2B4/GCK/UGT2B17/GYS2/AMY1C/SI/AMY2B/AMY1A/AMY2A/AMY1B
TICAM2/TLR4/IRAK2/BIRC3/NOD2/RIPK2/MEF2A/IKBIP/TAB2/DUSP7/CREB1/APP/UBE2D1/ATF2
TGFB2/TGFA/PIK3CA/STAT1/CDK6/EGFR/TGFBR2/TGFB3/KRAS/AKT3/PAK2/TGFBR1/RALB/POLK/
LAMA2/CAV1/RASA1/SGCD/TLR4/STAT1/NOD2/CD80/TLR3/ABL2/PIK3R1/EDN1/CREB1/SOS1/CC
TICAM2/REL/TLR8/TLR4/TLR7/TLR3/TLR1/TLR2/MAPK1/TRAF6/TLR9/TLR6/MYD88/TLR5/TBK1/N
FGF2/WNT2/WNT5A/WNT7A/NOTCH2/RUNX2/BMPR2/FGF7/PIK3CA/PIK3CG/FZD6/HES2/WNT7
MYL2/MET/HGF/EPHA2/VCL/ENAH/EGFR/ROCK1/NCK1/NCKAP1/PLEKHA7/LPP/CTNND1/CTNNB
NOTCH2/PIK3CA/PIK3CG/REL/FAT1/CDK6/EGFR/TGFBR2/KRAS/AKT3/PRKAA2/NRAS/PIK3R1/DD
NOG/RUNX2/BMPR2/BMPR1B/SMAD1/SMURF1/BMPR1A/BMP2/TOB2/TOB1
IL1A/STAT1/JAK1/HMOX1/STAT3
ROCK1/MYH9/MYH10/PPP1CB/LIMK1/ROCK2/PPP1R12A
SOX4/PMAIP1/JAG1/CTNNB1/MED1/CDH1/RB1/H19/TULP3/CCND1
IBSP/COL4A1/COL4A5/COL4A4/COL4A2/SPP1
OMD/KERA/LUM/PRELP/GALNS/OGN/GNS/GLB1L/ACAN
MET/CDH2/BDNF/IQGAP1/ADAM10/ROBO1/EGFR/IGF2/GNA13/GDNF/MMP3/CTNND1/CTNNB1
TGFB2/IL1RAP/IL1R1/IL1A/IRAK3/IRAK2/TGFB3/IL1RN/IFNA1/TRAF6/MAP3K1/MAPK14/MYD88/
HNF4A/HHEX/INS/HNF1A/PAX6/FOXA3/NKX6-1/PDX1/NKX2-2/MNX1/GCK/PKLR/FOXA2/BHLHA1
MET/HGF/DOCK7/RAP1B/GAB1/RAP1A
RASA1/PIK3CA/PIK3CG/PDGFRA/STAT1/PIK3R1/SOS1/JAK1/SHC1/MAP3K1/STAT3/MAP2K1/ELK1
FN1/ADAM12/THBS1/ITGB1/LAMA3/FGF2/TNC/TFPI/ITGA5
ITGB6/ITGB1/ITGB8/ITGB3/ITGB5/TEAD1/MAP4K5/MAP4K4/STK3/ACTA2/PAK2/LATS1/CTNNB1/
COL1A1/UNC5B/PIK3CA/IL1A/VCAM1/YAP1/ICAM1/CCN2/CIP2A/ARHGEF12/DCSTAMP/RLF/NTN
CXCL8/TICAM2/PIK3CA/PIK3CG/TLR8/TLR4/CXCL9/STAT1/TLR7/CD80/CD86/TLR3/TLR1/AKT3/TL
TGFB2/CDKN2B/CDK6/STAG1/TGFB3/PRKDC/WEE1/YWHAZ/EP300/YWHAG/E2F3/MCM4/BUB1/
RASA1/PIK3CA/STAT1/EGFR/NCK1/KRAS/NRAS/PIK3R1/SOS1/GAB1/GNAI3/SHC1/MAPK1/STAT3
PTGIS/EDNRA/PTGS2/ANXA1/MITF/HSD11B1/PTGFRN/PTGER3/AKR1C2/PTGES/EDN1/ANXA3/AI
HAVCR1/HSPG2/NRP1/GPC6/AGRN/CHMP2B/GPC4/ACE2/PIK3R4/RB1/SDC4/CTSL/CHMP3/SDC2
INPP4B/PIK3CA/PIK3CG/PTPN13/SBF2/MTMR2/PIK3R1/PIK3C2A/PLEKHA2/PTEN/PLEKHA5/RAB5
ITGB1/CAV1/EPHA2/DSG2/ARHGAP5/TRIO/DOCK4/ARHGAP21/DIAPH3/PAK2/ITSN1/VANGL1/EF
IL20RB/F2R/PDGFRB/OSMR/PDGFRA/IL2RA/F2RL2/PTPRD/EGFR/IL9R/IL6ST/SETD7/PIAS3/IL5RA/
MET/CDH11/CDH3/PDGFRB/CDH2/KIT/CDH6/PDGFRA/LATS2/EPHA2/TEAD1/EGFR/PRKCI/STK3/
ARG1/GPT/GATM/GLS2/F7/OTC/GAMT
SRM/CKM/PYCR3/ASL/ACY1/ODC1/ARG1/ARG2/SARDH/GATM/CKB/OTC/GAMT/CPS1
ITGB1/IGFBP5/PIK3CG/IRS1/FBXO32/PDK1/PTEN/SMAD3/GSK3B/IGF1R/MTOR/RICTOR/ILK/WAS
IFNL3/IFNL1/IFNL2/STAT1/JAK1/STAT2
ITGB1/WT1/NOTCH2/KIRREL1/PDGFRB/EMX2/SLIT2/SIX1/ITGA3/GLI3/LHX1/HOXD11/CD2AP/NC
TGFB2/PLAU/MMP2/CXCL8/COL24A1/PDGFRA/TH/DUSP5/NF1/EP300/SERPINB5/DUSP10/CREB
FGF16/PIK3CA/JMJD1C/ATRX/PRKDC/ARID5B/MYBL1/ERBIN/KAT6A/UHRF1/EP300/FOXO3/PTEN
CCL13/CXCL8/CARD18/MEFV/BIRC3/NOD2/RIPK2/NAIP/ERBIN/TAB2/NLRP3/TNFAIP3/NLRC4/BI
ITGB1/SEMA7A/SEMA3E/ITGA1/PLXNA1/PLXNC1
UNC5B/UNC5A/PIK3CA/UNC5C/TRIO/NCK1/PIK3R1/DAPK1/DOCK1/YES1/MAPK1/CDC42/DCC/M

GABRA3/APBA3/CAMKK1/RPS6KA6/NRXN3/PPFIBP2/NRXN1/GNG13/ADCY4/KCNA5/GNG2/CACI
PROC/BGLAP/F9/PROZ/F7/F2
KIT/PIK3CA/PIK3CG/RUNX1/LEF1/KRAS/AKT3/NRAS/PIK3R1/PIM1/SOS1/ZBTB16/PPARD/MAPK1
IFIT3/STAT1/IFIT1/JAK1/STAT2/JAK2/IFNGR1/OAS1/MED14/TAP1/MX1/IFNAR2/IFITM1/IFNAR1/
CP/SERPINE1/NT5E/SLC2A1/HIF1A/BHLHE41/RORA/PLIN2/LDHA/HK2/BHLHE40/PGK1/ADM/ENC
KIR2DL3/IL1A/KIR3DL2/KIR2DL1/CD80/CD86/HLA-G/KLRD1/HLA-DQA1/FAS/HLA-DPA1/KLRC1/H
INHBA/ITGB1/WNT5A/GJB6/RUNX2/PDGFRA/GLI2/FZD1/LEF1/GLI1/SNAI1/CTNNB1/SMO/CDH1
DSG3/DSG2/TJP1/PKP1/CTNNB1/CDH1/OCLN
SLC2A1/LDHA/PGK1/ENO1/PKM
S100A2/IGFBP3/TFAP2C/ITGA3/EGR2/PMAIP1/DST/JAG1/VDR/HBP1/EP300/SERPINB5/MFGE8/I
PTGS2/CXCL8/CSF2/IL2RA/FOSL1/GATA3/EGR2/MAF/PTPRK/ITCH/CBLB/NFATC3/GBP3/NFATC2/
DKK1/PLAU/WNT2/WNT5A/WNT7A/ROR1/FZD6/SFRP2/WNT7B/FOSL1/SFRP4/SOST/WNT10A/C
MYL2/PIK3CA/PIK3CG/PDGFRA/TRIO/NCF2/PIK3R1/LIMK1/MAP3K1/RALBP1/WASF1/PLD1/RPS6
MMP1/BACH1/CDKN2B/STAT1/PTEN/JAK1/SMAD3/RB1/ATF1/CDK2/MSH2/ATR/MAP3K5/BARD
GLDC/AMT/DAO/LIAS/DDO/LIPT2/PXMP2/HOGA1/HAO1/AGXT2/AGXT/GNMT/PRODH2
PLA2G6/PLA2G10/PLA2G2D/CYP2C9/CYP2E1/PLA2G12B/CYP1A2/PLA2G2A/CYP3A4/PLA2G1B
FZD1/LEF1/EP300/CTNNB1/MED1/GSK3B/APC/PITX2
SERPINE1/PTGS2/SLC2A1/SLC2A3/TGFA/IGFBP3/HIF1A/LDHA/PMAIP1/ANGPT1/SLC16A1/PGK1/
HMGA2/TGFB2/RUNX2/CTSK/WNT7B/LEF1/CSNK1A1/CTNNB1/SPP1/SMAD3/LEMD3/AGO2/RB1
TGFB2/GAS1/HHIP/PIK3CA/GLI2/PTHLH/PIK3R1/BOC/SMO/STIL/LRP2/CDON/PTCH1
TMOD3/ROCK1/SPEN/RHOBTB1/ACTN1/ROCK2/PDE5A/HSP90AA1/PHIP/VIM/TWF1/SRRM1/DBI
TGFB2/TLR4/VCAM1/ICAM1/TLR7/TGFB3/TLR2
SERPINE1/SKIL/STAT1/WWTR1/SMURF2/RNF111/USP9X/SMAD3/UBE2D1/SMAD7/SKI/ATP1B4/I
HAVCR1/HSPG2/NRP1/GPC6/AGRN/GPC4/ACE2/SDC4/CTSL/SDC2/SDC1/GPC1
PROC/BGLAP/F9/PROZ/F7/F2
EREG/AREG/TGFA/EGFR/AKT3/EPGN/FOXO3/CREB1/MAPK1/UHMK1/HBEGF/XPO1/ELK1/CCND1
COL1A2/COL1A1/PIK3CA/PIK3CG/PDPN/CLEC1B/PIK3R1/FCER1G/LCP2/PIK3CB/CDC42/PIK3R5/P
MYL2/TGFB2/ITGB6/ITGB1/ITGA2/ITGA11/ITGAV/LAMA2/ITGB8/ITGB3/ITGB5/ITGA5/SGCD/ITG/
H2BC7/H3C14/H2BC8/SMC4/H2BC6/H3C12/NEK7/H2AC7/H2BC14/H2BC4/H2BU1/H4C8/H2BC2
DKK1/WNT2/WNT5A/WNT7A/CTHRC1/FZD6/WNT7B/FZD1/FZD7/RYK/ROR2
NDUFS5/ATP5F1A/NDUFAB1/NDUFC2/NDUFA8/CDK5/UQCRC1/ATP5PF/UQCRHL/COX7C/PSEN2
CTSK/MITF/VCAM1/ICAM1/STAT1/CBL/PIK3R1/TAB2/TNFSF11/MAPK1/TRAF6/MTOR/CDC42/PA
FGF23/MMP1/FN1/VCAN/CP/FBN1/DMP1/PAPPA/IGFBP5/MMP2/MATN3/PENK/LTBP1/MEPE/A
DKK1/ZFHX4/PAX9/NOG/TCF4/TET1/ZNF281/LAMC1/GLI2/NR3C1/STAT1/LHX1/FOXO1/LEF1/PTI
DSG3/DSG2/ROCK1/CLSPN/PAK2/TJP1/PKP1/PLEC/CTNNB1/BIRC2/CDH1/H1-4/APC/OCLN/KPNA
IL31RA/IL11/OSMR/STAT1/IL6ST/CBL/OSM/CNTF/JAK1/JAK2/STAT3/LIF/PTPN11/CLCF1
TICAM2/UNC5B/UNC5A/TLR4/TNFRSF10A/TNFSF10/DAPK1/FAS/DCC/TNFRSF10B/APPL1/CASP8/
TGFB2/CDKN2B/CDK6/STAG1/TGFB3/PRKDC/WEE1/YWHAZ/EP300/YWHAG/E2F3/MCM4/BUB1/
FGF23/ANOS1/FGF2/FLRT3/PIK3CA/FGF10/FGF5/SPRED1/FRS2/KRAS/CBL/NRAS/PIK3R1/SOS1/G
CGB5/CGB3/CGB8/TGFA/KIT/TFAP2C/EGFR/TFAP2D/EP300/TFAP2A/ATAD2/KDM5B/VEGFA/PIT)
TNFSF4/CXCL8/PIK3CA/IL2RA/STAT1/CRLF2/JAK1/YES1/MAPK1/JAK2/STAT3/MTOR/CCL11/PTPN
MC3R/GRIN2C/SSTR3/CHRNA10/GABRB3/GLRA2/P2RX2/GABRA1/P2RY11/GPR83/GABRG2/TACI
INPP4B/MTMR2/PIK3C2A/PIK3R4/PIKFYVE/INPP5F/MTM1/INPP4A
AMN/PRSS3/PRSS1/CTRB1/CTRB2
EREG/AREG/TGFA/PIK3CA/EGFR/PAG1/EPGN/PIK3R1/GAB1/PTPN11/HBEGF
RECQL/CLSPN/PRKDC/BCL6/NBN/TP53BP1/SP1/BRCA2/CDK2/MSH2/TOPBP1/USP1/RBBP8/ATR/
CTSV/CTSK/TLR8/TLR7/TLR3/TLR9/CTSL/CTSB
BHMT/CDO1/CBS/MAT1A/GNMT
MYL2/ITGB1/CLDN6/MMP2/CLDN1/CLDN11/PIK3CA/PIK3CG/VCAM1/MSN/VCL/ICAM1/ARHGAP
DSG3/DSG2/ROCK1/CLSPN/TJP1/PKP1/PLEC/CTNNB1/BIRC2/CDH1/APC/OCLN/VIM/STK24/LMNI

IQGAP1/EPHA2/NCK1/DST/PEAK1/PAK2/ARHGAP31/VANGL1/PIK3R1/SPTBN1/USP9X/DLG5/CLT
QKI/PPP1R3C/TUBB3/NCKAP1/G0S2/TAB2/AZIN1/KLF9/CLOCK/PURA/NCOA4/EIF4G2/PPP2CB/Z
EREG/AREG/TGFA/PIK3CA/EGFR/KRAS/CBL/EPGN/NRAS/PIK3R1/SOS1/GAB1/SHC1/HSP90AA1
ITGB1/LIMS1/FERMT2/FLNC/PARVA/ACTN1/FLNA/ARHGEF6/ILK/RSU1
FGF23/FGF2/PIK3CA/STAT1/FGF5/FRS2/KRAS/NRAS/PIK3R1/LRRFIP1/SOS1/GAB1/BAG4/STAT3
ANLN/PODXL/WT1/APOL1/MYO1E/COL4A5/ITGA3/COL4A4/ACTN4/CD2AP/MYH9/PTPRO/E2F3/
INHBA/CAV1/BMPR2/TGFBR2/ACVR1/TGFB3/TGFBR1/SMAD1/SMAD7/MAPK1
MET/PIK3CA/HGF/KRAS/AKT3/CBL/STRN/PAK2/NRAS/PIK3R1/ETS1/RAP1B/C8orf34/SOS1/GAB1
NOX4/PIK3CA/KRAS/NRAS/PIK3R1/PIM1/SOS1/PTPN11/BCL2L1/GAB2
VCAN/ROS1/PTGS2/ANXA1/CHST11/COL4A1/FCGR2A/CXCL8/IL1R1/GJA1/NOX4/BDNF/SLIT2/TL
HSPA6/SERPINH1/BAG3/HSPA1A/TNFRSF21/HSPA13/HSPH1/NUP160/HSPA1B/NUP153/HSPA12
TLR8/TLR4/IL1A/TLR7/TLR1/TLR2/TRAF6/TLR9/TLR6/MYD88/TLR5/UBE2V1/NFKB1
MYL2/ITGB1/RDX/F2RL2/MSN/VCL/ROCK1/CCN1/PKN2/LIMK1/PTEN/ROCK2/ATF2/PPP1R12A/E
DKK1/PLAU/WNT2/WNT5A/WNT7A/ROR1/FZD6/PRICKLE1/SFRP2/WNT7B/FOSL1/SFRP4/SOST/
AHCY/CHDH/CHKB/GPX4/CSAD/PCYT1B/AMT/PHGDH/SOD3/PCYT2/DNMT3L/BCAT2/GPX2/GAD
ITGAV/MFAP5/ITGB3/ITPR3/CREB1/MAPK1
CXCL8/CXCL5/IFIT2/CSF2/CXCL9/CXCR2/STAT1/TLR7/CXCL6/HAVCR2/DDX58/CXCL10/EP300/TF
DKK1/PLAU/WNT2/WNT5A/WNT7A/ROR1/FZD6/SFRP2/WNT7B/FOSL1/SFRP4/SOST/WNT10A/F
NOS1/SCN4A/ATP1A4/SCN1A/DMPK/TRDN/KCNJ4/ATP2B3/KCNQ1/CACNA1C/GATA4/CACNG8/
COL1A2/COL1A1/IL11/NOX4/HIF1A/CCN2/TGFBR2/IL6ST/ACTA2/TGFBR1/ACE2/SP1/SMAD3/M
RAB23/EVC/PKD2/GLI2/GLI3/LCA5/CNGB1/ARL13B/RP2/GPR161/USP9X/SMO/EVC2/WDPCP/PK
OR56A5/OR10P1/OR2J2/OR51Q1/OR13H1/OR2AJ1/OR52E5/OR2AK2/OR51A7/OR51F2/OR14J1/
CGB5/CGB3/CGB8/TGFA/KIT/TFAP2C/EGFR/TFAP2A/ATAD2/VEGFA/ESR1
H2BC7/H2BC8/RNF144A/H2BC6/H2BC14/H2BC4/PRKDC/SHPRH/CDC73/UBA6/H2BC5/USP9X/R
TGFB2/SLC2A1/CTSK/TGFA/HIF1A/TGFB3/PDGFB/COL21A1/EP300/VEGFA/DIAPH1/ARNT
H2BC7/H3C14/H2BC8/PYGO1/H2BC6/TCF4/BCL9L/H3C12/H2AC7/H2BC14/H2BC4/LEF1/H2BU1/
PTGS2/CYP1B1/NRIP1/KLF6/AHR/EGFR/KRAS/NRAS/NF1/EP300/RB1/NFE2L2/VEGFA/CDK2/HSP
RFXANK/LCK/IGLL1/BLNK/CD3D/AIRE/RAG2/ZAP70/TNFRSF13B/AICDA/CD79A/TNFRSF13C/CD1
HMGA2/H2BC7/H3C14/H2BC8/CXCL8/H2BC6/IL1A/H3C12/H2AC7/CDKN2B/H2BC14/PHC3/CDK
PIK3CA/STAT1/PIK3R1/LRRFIP1/STAT3/FGFR1OP2/GAB2/MYO18A/CPSF6/ZMYM2
COL1A2/SERPINE1/CDKN2B/WWTR1/CCNK/EP300/RNF111/SP1/SMAD3/SMAD7/MAPK1
ITGAV/CAV1/PIK3CA/PDGFRA/PIK3R1/SOS1/JAK1/SHC1
NOD2/RIPK2/DDX58/VCPIP1/IFIH1/TNFAIP3/PTEN/UBE2D1/ZRANB1/APC/TRAF6/ESR1/NOD1/Y
CDH11/MMP8/MMP2/BDNF/TWIST1/EFNA5/TRIO/AKT3/KIDINS220/EFNB2
FN1/ADAM12/ITGB1/TGM2/TNC/F13A1/CSF2/VCAM1/ITGA9/SPP1/CSF2RA/VEGFA
MYLK/PIK3CA/ANGPTL2/PIK3CG/ITPR2/ROCK1/ITPR3/ACTR2/AKT3/PAK2/PIK3R1/LIMK1/ACTR3/
KAT2A/INS/MAMLD1/HNF1A/PAX6/FOXA3/NKX6-1/PDX1/RFX6/NKX2-2/ONECUT3/GCK/PKLR/IN
H2BC7/H3C14/H2BC8/H2BC6/AR/H3C12/H2AC7/H2BC14/H2BC4/H2BU1/MYH9/MYH10/H4C8/
GREM1/NOG/BMPR2/BMPR1B/SMURF2/SMAD1/TAB2/CTDSP2/ZFYVE16/SMAD7/SKI/PPP1R15
ITGB1/CAV1/VCL/ROCK1/TNS1/SOS1/ACTN1/SHC1/MAPK1/RAP1A/MAP2K1/TLN1/MAPK8/RHO
THBS1/F2R/RIPK2/TNFRSF21/PAK2/ETS1/NET1/APP/SMAD7/ANXA5/MAPK1/CTNNA1/VIM/CUL
ITGB1/ITGA2/ITGAV/NRP2/SCN5A/L1CAM/ITGB3/RDX/ITGA5/SCN7A/LAMC1/NRP1/MSN/SPTA1
FGF23/IBSP/ITGAV/COL1A1/ITGB3/PDGFRB/PDGFRA
PIK3CA/PIK3CG/NTRK1/PIK3R1/CREB1/SHC1/MAPK1
NDUFA5/NDUFA10/NDUFAF3/NDUFS2/NDUFA6/FOXRED1/NDUFAF2/TMEM70/NDUFS5/NDUFA
COL1A2/PTGS2/KIT/IQGAP1/BIRC3/CDK6/GATA3/MAF/LEF1/KRAS/KITLG/PPP3CA/PIAS3/TFEC/N
PIK3CA/CDK6/PIK3R1/RB1/CDK2/MAPK1/CCNE1/CCND1/NFKB1/RHOA/TFDP1/PAK1/CHUK/RAC
PIK3CA/IRS1/NTF3/PTPRO/KRAS/NRAS/PIK3R1/SOS1/SHC1
PIK3CA/AKT3/PTEN/SOS1/SHC1/MAPK1/ILK/RBL2/AKT2
DKK1/CCN4/WNT2/TBXT/WNT5A/WNT7A/PYGO1/FZD6/WNT7B/FOSL1/SFRP4/WNT10A/FZD1/L

KIT/PIK3CA/STAT1/KRAS/NRAS/PIK3R1/SOS1/YES1/JAK2/STAT3
NDUFA4/POLR2E/POLR2J3/NDUFS5/CLTA/ATP5F1A/NDUFAB1/BBC3/NDUFC2/NDUFA8/DLG4/B/
NBN/RBBP8/TP73/CHEK1/MDM2/ABL1/NFKB1/MAPK8/RAD50/GADD45A/MRE11/ATM/BRCA1/
H2BC7/H2BC8/H2BC6/H2BC14/H2BC4/H2BU1/CLSPN/H4C8/H2BC21/NBN/H2BC5/TP53BP1/RNF
H2BC7/H2BC8/RNF144A/H2BC6/H2BC14/H2BC4/PRKDC/SHPRH/CDC73/H2BC5/RNF152/UBE2D1
CAV1/AKT3/YWHAZ/YWHAG/YWHAQ/AKT2/YWHAB
ITPR2/ITPR3/PPP3CA/NFATC3/NFATC2/ITPR1/AHCYL1/PPP3R1
FGFR2/MRPL40/KLHL22/C22orf39/TRMT2A/SERPIND1/SREBF1/GNB1L/SHH/SLC25A1/HIRA/TXNI
NOD2/RIPK2/TNFAIP3/ERC1/BIRC2/TRAF6/XPO1/BCL10/MALT1/PRKCA/CYLD/NFKB1/ATM/UBE2
IL1RAP/IL36RN/IL1R1/CSF2/IL1A/IL36B/SLC20A1/STAT1/GATA3/CKAP2L/ARL14/RGPD8/IL1RN/M
HMGA2/H2BC7/H2BC8/H2BC6/H2AC7/H2BC14/H2BC4/H2BU1/H4C8/HMGA1/H2BC21/NBN/H2I
SOX11/GREM1/FAT4/SLIT2/GLI3/LHX1/GATA3/ROBO2/GDNF/FOXC1/FOXC2/CTNNB1
CHST11/CHSY3/DSE/CHST3/CSGALNACT2/CHSY1/XYLT1
WNT2/WNT5A/WNT7A/HHIP/FZD6/WNT7B/GLI2/GLI3/WNT10A/FZD1/LEF1/GLI1/FZD7/CTNNB1
NBN/RAD21/SMC3/SMC1A/RAD50/MRE11/ATM/RAD18/BRCA1
PIK3CA/GNGT1/CREB1/SP1/MAPK1/GNB1/ESR1/MAPK14/MAP2K1/ELK1/NFKB1/BRAF/FOS/CHU
ADRA1B/GNB4/ECT2/RASGRF2/GNG12/TRIO/GNGT1/PLEKHG2/ROCK1/GNA13/ARHGEF12/ITSN1
PLAT/PLAU/MMP2/LRP1/ICAM1/CCN2/PLAUR/ROCK1/THY1/ITGAM/RAP1B/ITGB2/YES1/RAP1A/
H2BC7/H2BC8/H2BC6/H2BC14/H2BC4/RIF1/H2BU1/PRKDC/H4C8/H2BC21/NBN/H2BC5/TP53BP
PLAU/WNT2/WNT5A/WNT7A/FZD6/PPP2R3A/WNT7B/FOSL1/WNT10A/PRKCI/FZD1/CD44/LEF1/
ROCK1/MYH9/MYH10/ARHGEF12/LIMK1/ROCK2
PIK3CA/F2RL2/TRIO/PHLDB2/ROCK1/SPRED1/TAOK1/EPHB2/CLIP1/LIMK1/PTEN/GSK3B/APC/ST/
PIK3CA/PDGFRA/STAT1/KRAS/STRN/NRAS/PIK3R1/SOS1/STAT3/PIK3CB
CTSK/ITGB3/PDGFB/GPR68/SPP1/TNFSF11/IFNAR1/MAPK8/IFNB1/SLC9A1
H2BC7/H3C14/H2BC8/TGFA/H2BC6/H3C12/ZNF217/H2AC7/NRIP1/H2BC14/H2BC4/RUNX1/STA(
CORIN/ANXA1/PIK3CA/PIK3CG/NR3C1/PIK3R1/HSP90AA1
COL5A2/COL6A3/COL3A1/COL5A1/ST8SIA2/COL4A1/COL6A1/COL5A3/COL4A5/COL4A4/COL4A2
GABBR2/SLC6A3/HTR3A/PICK1/KCNJ4/GNG8/TUBB8/CACNG8/VAMP2/PRKCB/NRGN/GNB3/RAB
PLAU/GREM1/FGF2/MMP2/CXCL8/SKIL/TGFA/FGF7/CSF2/HGF/SFTPA1/CCN2/CCR2/PDGFB/EDN
FOXO1/PRKDC/AKT3/YWHAZ/YWHAG/FOXO3/GSK3B/HSP90AA1/KPNA1/MTOR/RICTOR/MAP3K
CYP24A1/RSAD2/CXCL8/CAV1/TLR8/TLR4/STAT1/NOD2/IFI44L/KRAS/VDR/OAS2/TLR2/NRAS/ETS
ITGB1/H2BC7/H3C14/H2AC13/H2BC8/H2BC18/H2BC6/H3C12/H2AC7/H2BC14/TUBB3/H2BC4/E(
CAV1/RASA1/PIK3CA/F2RL2/IRS1/PRKCI/NCK1/CBL/PIK3R1/EXOC5/FOXO3/SOS1/SHC1/RHOQ/P
H2BC7/H3C14/H2BC8/H2BC6/H3C12/H2AC7/H2BC14/H2BC4/RUNX1/LEF1/H2BU1/H4C8/CSF3R/
FGF23/FGF2/FGF16/FGF7/PIK3CA/FGF10/FGF5/FRS2/KRAS/NRAS/PIK3R1/SOS1/GAB1/SHC1
RSAD2/IFIT2/OAS3/IFIT3/STAT1/XAF1/OAS2/IFIT1/IFNA1/HLA-G/JAK1/STAT2/USP18/GBP2/SAM
NDUFA4/SMIM20/TACO1/COX17/COX7C/COX19/PET117/COA6/COX7A2/COX7B/SURF1/COX6C/
EPHA3/MMP2/RASA1/EPHB6/EPHA2/ADAM10/EPHA4/EFNA5/ROCK1/ACTR2/MYH9/MYH10/PA
ITGB1/PIK3CA/PIK3CG/STAT1/IRS1/CBL/PIAS3/PIK3R1/YWHAZ/YWHAG/SOS1/JAK1/FLNA/GSK3B
CHDH/CHKB/GPX4/CSAD/PCYT1B/SOD3/PCYT2/BCAT2/GPX2/GAD1/BHMT/CDO1/SARDH/AGXT2
AREG/CXCL8/PIK3CG/IL1A/CXCL9/AHR/CCR2/CXCL10/PTGES/IFNA1/NLRP3/KLRD1/VEGFA/CSF1/
PIK3CA/CSF2/IL2RA/STAT1/IL9R/HAVCR2/IL5RA/PIK3R1/SOS1/JAK1/CSF2RA/SHC1/JAK2/STAT3/I
IL1A/RIPK2/PRKDC/PAK2/RB1/MAP3K1/FAS/LMNB1/MAP3K7/CASP8/CFLAR/APAF1/SPTAN1/M/
PANX1/IRAK2/MEFV/BIRC3/NOD2/RIPK2/TAB2/P2RX7/NLRP3/TNFAIP3/ITCH/APP/NLRC4/BIRC2/
PIK3CA/HRH1/SOCS5/STAT1/IRS1/GATA3/CBL/PIK3R1/EP300/SOS1/JAK1/ATF2/NFIL3/FLNA/SHC
INHBA/DKK1/FGF2/WNT2/NT5E/WNT5A/NOG/IL11/KIT/HGF/WNT7B/FGF10/TGFB3/KITLG/PDGI
IL20RB/IFNL3/IFNL1/IFNK/IFNL2/IL11/PIK3CA/PIK3CG/OSMR/CSF2/IL2RA/SOCS5/LEPR/STAT1/IL
YAP1/STK3/RIF1/PRKDC/NBN/LATS1/TP53BP1/ATF2/ACTL6A/BRCA2/TRAF6/ATR/SMC3/SMC1A/
THBS1/SERPINE1/IGFBP3/CCNG2/CDK6/PMAIP1/ZMAT3/SERPINB5/PTEN/CDK2/FAS/STEAP3/AT
PIK3CA/PIK3CG/BDNF/IRAK3/IRAK2/IRS1/RIPK2/NTF3/FRS2/KRAS/AKT3/KIDINS220/NTRK1/NRA

PIK3CA/PIK3CG/FOXO1/KRAS/NRAS/PIK3R1/RB1CC1/PDK1/FOXO3/PTEN/GSK3B/PIK3CB/MTOR/
LDHA/PGK1/ENO1/PKM/RB1/PRKAA1/HK1
LAMA2/MYH8/CAV1/SGCD/ICAM1/CD80/CD86/MYH9/MYH10/MYH13/ABL2/HLA-G/MYH15/EIF
GJA1/PIK3CA/FZD1/LEF1/EIF2AK2/PIK3R1/CTNNB1/GSK3B/APC/MYD88/CCND1/NFKB1
MAML2/YAP1/TEAD1/ITGA4/CCN2/WWTR1/RUNX1/LEF1/JAG1/KRAS/SMURF2/EP300/ZFHX3/F(
TLR8/SEC23A/IRAK2/STAT1/TLR7/NOD2/RIPK2/TLR1/SEC24A/NLRP12/TLR2/DDX58/TAB2/IFIH1/
FGF23/FGF19/ANOS1/FGFBP1/FGF2/FGF16/FLRT3/FGF7/PIK3CA/FGF10/FGF5/SPRED1/FRS2/KR/
CAV1/FAM83B/EPHA2/ARHGAP5/KIF14/PTPN13/DST/FRS2/KIDINS220/VANGL1/PIK3R1/UHRF1E
FOSL1/EIF2AK2/FOSL2/TNFSF11/TRAF6/IFNAR2/IFNAR1/NFKB1/MAPK8/IFNB1/FOS
AQP11/AQP3/AQP5/AQP7/AQP4/AQP10/AQP12A/AQP8
ITGB1/ITGAV/L1CAM/ITGB3/ITGA5/NRP1/EGFR
ANKRD1/IL1R1/MYOG/ZEB1/IL1A/ADAM10/CCN1/NR4A3/ATF3/VEGFA/HBEGF/DUSP14/EIF4E/IL
INS/HNF1A/PAX6/FOXA3/NKX6-1/PDX1/RFX6/NKX2-2/GCK/PKLR/FOXA2
RASA1/PIK3CA/BDNF/PRKCI/NTF3/FRS2/KRAS/NTRK1/NRAS/PIK3R1/RAP1B/SOS1/GAB1/DOCK1,
HSPA6/KIR2DL3/KIR3DL2/KIR2DL1/HSPA1A/KIR2DS4/IFNA1/HLA-G/CREB1/HSPA1B/KLRD1/HSP9
ADAM17/ECT2/RASGRF2/BIRC3/TRIO/PRKCI/RIPK2/PLEKHG2/GNA13/ARHGEF12/ITSN1/TNFRSF
NEDD4L/CLCN2/FKBP1B/ATP2C2/SCNN1B/ATP13A1/TRPV1/CASQ1/RYR2/FXYD3/ATP6V1G2/SLC
TGFA/FGF10/EGFR/SMAD1/FOXC1/FOXC2/INHBB/DKK2/PITX2/MAP3K1
OR56A5/OR10P1/OR2J2/OR51Q1/OR13H1/OR2AK2/OR51A7/OR51F2/OR14J1/OR2G3/OR4A16/
LAMA2/YAP1/PMP22/LAMC1/TEAD1/LAMB1/DRP2/WWTR1/EGR2/ADGRG6/UTRN
CXCL8/CD86/CBL/IL5RA/PIK3R1/SOS1/CCR3/JAK1/SHC1/MAPK1/JAK2/STAT3/PTPN11/MAP2K1/
PLAT/TICAM2/PIK3CA/REL/TLR4/IRAK2/TLR3/ITGAM/CXCL10/FGG/CBLB/ITGB2/TRAF6
ITGB1/IGKV1D-33/IGKV2D-28/IL1R1/IGKV5-2/IGLV3-25/NR3C1/ITGA4/TLR7/IGKV2-28/ROCK1/N(
PIK3CA/PIK3CG/EGFR/LEF1/KRAS/AKT3/NRAS/PIK3R1/FOXO3/CTNNB1/PTEN/SOS1/CDH1/GSK3E
VCAN/CHST11/CHSY3/CHST3/CSGALNACT2/DCN/CHSY1/BGN/CHST15
IGFBP3/HIF1A/HSPA1A/EP300/HSP90AA1/DNAJB1/TAF1/MDM2/MAPK8/GADD45A/FHL2/ATM
ITGB1/CLDN1/TLR4/TUBB3/ROCK1/NCK1/YWHAZ/CTNNB1/ROCK2/CDH1/OCLN/EZR/CDC42/WA
PIK3CA/BDNF/NTF3/FRS2/KRAS/NRAS/PIK3R1/SOS1/GAB1/SHC1/PTPN11
AMT/CA9/CA14/ASNS/CA8/GLS2/CA4/CA6/CPS1
OMD/KERA/LUM/PRELP/B4GALT5/B4GALT1/ST3GAL1/CHST6/B3GNT2/GALNS/ST3GAL4/OGN
PRTN3/F5/FGB/F11/F9/KNG1/F12/F13B/SERPINC1/F7/KLKB1/GP1BA/F2/GP9
ATP1A4/PCK2/SLC9A3/SLC25A10/FXYD2/SLC38A3/ATP1A2/GLS2/CA4/ATP1A3
NKX6-1/NKX2-2/LMX1B/SOX2/FGF8/ASCL1/OTX2/DDC/FOXA2/NEUROG2
MYL2/TGFB2/ITGB6/ITGB1/ITGA2/ITGA11/ITGAV/LAMA2/ITGB8/ITGB3/ITGB5/ITGA5/SGCD/ITG/
ECT2/CLSPN/KIF20A/WEE1/CENPE/KIF2A/PPP1CB/ROCK2/BUB1/PPP1R12A/PRC1/STAG2/INCEN
PIK3CA/PDGFRA/STAT1/KRAS/NRAS/PIK3R1/SOS1/STAT3/PIK3CB
TOMM40/TIMM8B/EMD/PHYH/STX1A/SEC61G/CYB5A/HACL1/TIMM10/UBE2J2/BCS1L/ATP5F1/
PTPRU/KIT/PIK3CA/STAT1/FER/KRAS/KITLG/CBL/NRAS/PIK3R1/SOS1/SOCS6/YES1/JAK2/STAT3/S
FN1/ITGB3/QKI/IQGAP1/VCL/SPRED1/KRAS/NRAS/NF1/RAP1B/DUSP10/DUSP7/PPP1CB/MRAS/I
INHBA/MYL2/TBX5/DKK1/TBX20/FGF2/TBXT/NOG/SCN5A/KIT/PDGFRA/BMP1/THY1/MYLK3/IGF
PLAU/ITGAV/ITGB3/SPP1/MAPK1/MAP2K1
ITGB1/TUBB3/KIF18A/TAOK1/DIAPH3/DYNC1LI2/CENPF/CENPE/DYNC1I2/CLIP1/KIF2A/KNL1/NU
RASA1/PIK3CA/IL2RA/STAT1/IRS1/KRAS/NRAS/PIK3R1/SOS1/JAK1/SHC1/CDK2/MAPK1/STAT3/S
IGKV1D-33/IGKV2D-28/MS4A2/IGKV5-2/IGLV3-25/IGKV2-28/IGHV2-70/IGLV7-43/IGHV3-33/IGK\
TICAM2/TLR4/TNFRSF10A/TNFSF10/FAS/TNFRSF10B/CASP8/CFLAR
DKK1/DMP1/NOTCH2/RUNX2/DSPP/SOST/MIR32/NOTCH3
PIK3CA/CSF2/IL2RA/IL5RA/PIK3R1/SOS1/JAK1/CSF2RA/SHC1/JAK2/PIK3CB
TREM1/CXCL8/TLR4/IL1A/TLR2/IFNA1/MAPK1/TRAF6/MYD88
PIK3CA/IRS1/PIK3R1/YWHAZ/SOS1/PRKD1/SHC1/IGF1R/PTPN11/PTPN1/RPS6KB1/CRK/PXN/PTK
TGFB2/MAP4K5/STAT1/MAP4K4/TGFB3/MAP3K2/PAK2/TGFBR1/CREB1/MAPK6/SP1/MAP4K3/A

ADAM12/MFAP5/NOTCH2/MAML2/MFAP2/ADAM10/GATA3/NOTCH3/JAG1/MIB1/CBL/ENO1/E
KRT14/MMP1/KRT5/CXCL8/CSF2/NR3C1/ICAM1/STAT1/GATA3/KRT17/NCOA2/FKBP5/EP300/CR
TREM1/PIK3CA/PIK3CG/NCF2/SIGLEC7/PIK3R1/ITGAM/PIK3C2A/FCER1G/C1QC/ITGB2/CYBB/PIK
FOXO1/AKT3/YWHAZ/YWHAG/FOXO3/YWHAQ/AKT2/YWHAB
PLAU/SERPINE1/E2F7/HBP1/EP300/E2F3/SP1/RYBP/RB1/CDK2/MCL1/TOPBP1/SIRT1/RBBP8/CC
RASA1/BIRC3/PRKDC/PAK2/BIRC2/RB1/BAG4/MAP3K1/FAS/LMNB1/PSEN1/XIAP/MAP3K5/CASP
MRPS7/ERAL1/MRPS11/MRPL28/MRPL11/MRPS15/TUFM/MRPL21/MRPS12/MRPL14/MRPL40/
S100A2/IL1RAP/SERPINE1/WT1/YAP1/CDK6/NTRK1/EP300/FOXO3/JAK1/ITCH/SP1/BUB1/RB1/B
AGPAT2/DGAT2/DGKQ/GLYCTK/AWAT2/PNLIPRP2/PNLIPRP1/CEL/PNLIP
RASA1/EPHB6/ROCK1/ACTR2/ITSN1/EPHB2/EFNB2/LIMK1/ACTR3/ROCK2/YES1/CDC42/WASL/E
MET/HGF/KRAS/NRAS/SOS1/SHC1/RANBP9
DKK1/WNT2/WNT5A/WNT7A/NFAT5/FZD6/PRICKLE1/SFRP2/WNT7B/FOSL1/SFRP4/WNT10A/FZ
ROS1/HIF1A/NAMPT/FOXO1/FOXO3/SIRT1/PPARG/NFKB1/TFAM
SPHKAP/ITGAV/ITGB3/PIK3CA/PIK3CG/PDGFRA/PIK3R1/MAPK1/SPHK1
NUP160/TNPO1/NDC1/NUP107/RCC1/NUP58/NUP205/NUP98/AHCTF1/KPNB1/NUP54/NUP155
PPP3CA/CSNK1A1/YWHAZ/PIM1/EP300/YWHAG/NFATC3/GSK3B/MAP3K1/NFATC2/MAPK14/XP
RIF1/PRKDC/NBN/TP53BP1/RNF168/BUB1/ATF2/PRKAA1/MTOR/RBBP8/ATR/MAP3K5/SMC1A/
RRAD/PIK3CA/BDNF/PCSK5/FOSL1/F3/PCSK6/IRS1/EGR2/MEF2A/NTF3/TCF12/REST/FRS2/PTPR
STAT1/IL6ST/CBL/JAK1/JAK2/STAT3/PTPN11/SOCS3
FGF23/FGF2/FGF16/PIK3CA/FGF5/FRS2/KRAS/CBL/NRAS/PIK3R1/SOS1/GAB1/SHC1/GALNT3/MA
NOX4/PIK3CA/KRAS/CBL/NRAS/PIK3R1/PIM1/SPTBN1/SOS1/TRIP11/PTPN11/GOLGB1/ETV6/BCL
FGF23/FGF19/FGF2/FGF16/FGF7/PIK3CA/FGF10/FGF5/IRS1/IGF2/FRS2/KRAS/NRAS/PIK3R1/PIK3
EP300/FOXO3/PTEN/MCL1/GABPA/CREBBP/ETV6/BCL2L1/ATM/ZFX
TGFB2/STAT1/TGFB3/TGFBR1/CREB1/ATF2/RAPGEF2/SHC1/MAP3K1/CDC42/MAPK14/MAP3K5/
ITGAV/ITGB3/PDGFRB/GNA13/AKT3/FLT1/S1PR3/PDGFB/GNAI3/VEGFA/MAPK1/JAK2/GNAQ
IL31RA/IL11/OSMR/IL6ST/OSM/CNTF/JAK1/JAK2/LIF/CLCF1
MET/SLC2A3/TRPC3/OPRK1/OPRM1/FKBP5/GRIN2A/CREB1
PIK3CA/ANGPT1/KRAS/NRAS/PIK3R1/ANGPT2/SOS1/SHC1/PIK3CB/PTPN11/TEK/ANGPT4
PCBD1/GSTZ1/HGD/QDPR/HPD/TAT
CACNA1C/GNG8/INS/VAMP2/GNB3/KCNG2/ACACB/PRKAR1B/GNA14/GNG4/STXBP1/ADRA2C/S
F5/FGB/F11/F9/SERPINF2/F12/F13B/F7/KLKB1/F2/KLK1
H2BC7/H3C14/H2BC8/CXCL8/H2BC6/IL1A/H3C12/H2AC7/CDKN2B/H2BC14/CDK6/H2BC4/H2BU
CACNA1C/GNG8/INS/VAMP2/GNB3/KCNG2/PRKAR1B/GNA14/GNG4/STXBP1/ADRA2C/SYT5/GN
NCK1/NCKAP1/ACTR2/WIPF1/ACTR3/WASF2/MAPK1/CDC42/ABI2/WASL/CYFIP1/WASF1/WIPF2
RASA1/PIK3CA/PIK3CG/IRS1/PIK3R1/SOS1/SHC1/PTPN11/MAP2K1/ELK1
FGF23/FGF2/FGF16/PIK3CA/FGF5/FRS2/KRAS/NRAS/PIK3R1/SOS1/GAB1/SHC1
FGF23/FGF2/FLRT3/PIK3CA/FGF10/FGF5/FRS2/KRAS/NRAS/PIK3R1/SOS1/GAB1/SHC1/FLRT2/PT
ITGB1/PIK3CA/PIK3CG/PIK3R1/MTOR/XIAP
HSPG2/GPC6/AGRN/GPC4/EXT2/EXT1/SDC4/SDC2/SDC1/GPC1
EREG/AREG/TGFA/EGFR/KRAS/EPGN/NRAS/SOS1/SHC1
HSPA6/SERPINH1/BAG3/HSPA1A/TNFRSF21/HSPA13/HSPH1/EP300/NUP160/HSPA1B/NUP153/
SNAI2/MITF/KIT/PIK3CA/STAT1/FER/SPRED1/PTPRO/KITLG/CBL/PIK3R1/FOXO3/PTEN/SOS1/GA
HSPA6/SERPINH1/HSPA1A/TNFRSF21/HSPH1/EP300/HSPA1B/HSP90AA1/DNAJB1/GML/CREBBP/
ATP2B3/ATP2B2/P2RX2/SLC8A3/ATP2A3/P2RX6/TRPC7/SLC8A2/P2RX1/P2RX5
IL1RAP/IL1R1/IL1A/IRAK2/TAB2/ATF2/MAPK1/TRAF6/MAP3K1/TANK/TAB3/MAPK14/MAP3K8/
RASA1/PIK3CA/PIK3CG/STAT1/PIK3R1/SOS1/SHC1/JAK2/STAT3/MAP2K1
H2BC7/H3C14/H2AC13/H2BC8/H2BC18/H2BC6/H3C12/H2AC7/H2BC14/H2BC4/H2BU1/REST/H2
IFNL3/IFNL1/IFNK/IFNL2/PIK3CA/STAT1/PIK3R1/IFNA1/JAK1/IFNL4/STAT2/JAK2/IFNGR1
PIK3CA/PIK3R1/YWHAZ/PIM1/OSM/YWHAG/SHC1/JAK2/PTPN11/BCL2L1/PRKACB/GAB2
SLC4A1/HBB/CA4/HBA2/HBA1

RUNX1/KRAS/EP300/CBFB/CDKN2A/CCND1
SEMA3A/NRP1/PLXNA1
TUT7/CNOT6/CNOT1/EIF4G1/TNKS1BP1/TUT4/CNOT8/EIF4E/PABPC1/EIF4A2/PAIP1/PARN/CNO
PRKDC/NBN/CDK2/TOPBP1/RBBP8/ATR/BARD1/CCNE1/XRCC5/RAD50/MRE11/ATM/UBE2D3/FA
PLAT/PLAU/TGFBI/FCGR2A/VCAM1/ICAM1/PLAUR/THY1/CCN1/ITGAM/FGG/ITGB2/SPON2/C3
ALDH4A1/GOT1/FH/ASL/MDH2/NOS1/ARG1/NOS2/GPT/GLS2/OTC/CPS1
ATP2B2/PLCB4/GNA14/GRIA1/GRM1/CACNA1A/CA8/GRIA2/PRKCG
CAV1/ST3GAL1/PARP14/PARP9/PARP4/CHMP2B/ACE2/PIK3R4/RB1/GSK3B/ST3GAL4/CANX/GAL
DDO/ADSS1/ACY3/ASNS/ABAT/GAD1/GPT2/GPT/GLS2/AGXT2/AGXT/CPS1
TBX5/RUNX2/YAP1/TEAD1/CCN2/WWTR1/HIPK1
VAMP2/RAB3A/CACNA1B/SLC25A4/SLC18A3/STXBP1/UNC13A/SYN1/ATP1A2/SLC32A1/CACNA1
GOT1/SUOX/AHCY/ETHE1/CSAD/MPST/BHMT/CDO1/CBS/MAT1A
MGST2/GGT7/ACY1/CYP3A5/DPEP3/ACY3/AKR7A3/GGT6/AKR7L/CYP1A2/DPEP1/CYP3A4
ITGB1/CXCL8/IL1A/VCAM1/ICAM1/ITGA4/C7/ITGB2/C3
ITGB1/CXCL8/IL1A/VCAM1/ICAM1/ITGA4/C7/ITGB2/C3
CAV1/RETN/NR3C1/CCNG2/FBXO32/FOXO1/ABCA6/AKT3/BCL6/YWHAZ/SOD2/EP300/YWHAG/F
OMD/KERA/LUM/PRELP/B4GALT5/B4GALT1/ST3GAL1/CHST6/B3GNT2/ST3GAL4/OGN
CRP/PRDM1/STAT1/IL6ST/PIK3R1/SOS1/JAK1/GAB1/GSK3B/SHC1/MAPK1/JAK2/STAT3/PTPN11/
ATP1A4/SCN1A/MYH3/DMPK/TRDN/KCNJ4/ATP2B3/KCNQ1/CACNA1C/GATA4/DES/CACNG8/AT
DEFB134/PRSS3/PRSS2/DEFA6/DEFA5
PRDM1/HIF1A/CD274/EP300/STAT3
PIK3CA/PIK3CG/PIK3R1/EP300/JAK1/IL7/IL7R/NMI/CREBBP
IFNK/CXCL8/DDX3Y/DDX58/CXCL10/IFIH1/IFNA1/AZI2/TRAF6/DDX3X/MAP3K1/TANK/MAPK14/S
COL5A2/COL6A3/COL3A1/COL5A1/ST8SIA2/COL4A1/COL6A1/COL5A3/COL4A5/COL4A4/COL4A2
NOS1/ATP1A4/DMPK/TRDN/ATP2B3/ATP2B2/FXYD1/SLC8A3/FXYD2/FXYD4/CASQ2/ATP1A2/FXY
THBS1/SERPINE1/E2F7/SLC2A1/ICAM1/PMAIP1/NCF2/ZMAT3/TNFRSF10D/PRKAA2/POLK/SERPI
NDUFS5/NDUFAB1/ATF4/CEBPA/BBC3/NDUFC2/NDUFA8/BAX/UQCRC1/UQCRHL/SREBF1/COX7
KIF14/MYH9/MYH10/PPP1CB/PPP1R12A/PRC1
GNG13/ADCY4/GNG2/AQP11/AVP/GNG8/AQP3/GNB3/PRKAR1B/AQP5/GNG4/AQP7/AQP4/GNG
ITGB1/CXCL8/IL1A/VCAM1/ICAM1/ITGA4
PLA2G6/PLA2G10/PLA2G2D/PLA2G12B/PLA2G2A/PLA2G1B
NKX6-1/PDX1/PTF1A/RBPJL
H2BC7/H3C14/H2BC8/SMC4/H2BC6/H3C12/H2AC7/H2BC14/H2BC4/H2BU1/H4C8/H2BC21/H2B
FGF23/FGF19/FGF2/FGF16/FGF7/PIK3CA/FGF10/FGF5/IRS1/FRS2/KRAS/NRAS/PIK3R1/PIK3R4/S
KCNK7/KCNK16/KCNK10/KCNK3
ITGB1/PTPRR/PDGFRA/EGFR/SOS1/SHC1/MAPK1/STAT3/IGF1R/MAP2K1/ELK1
RASA1/PIK3CA/STAT1/EGFR/PIK3R1/SOS1/JAK1/SHC1/MAP3K1/STAT3/MAP2K1/ELK1/PRKCA/M
SST/AVP/PPARGC1A/HTR3A/KCNQ1/IL13/SNTA1/VAMP2/POU2F2/GNB3/HES5/SLC9A3/PHOX2A
KRAS/PTPN12/ERBIN/NRAS/SOS1/SHC1/HSP90AA1
PIK3CA/STAT1/CBL/PIK3R1/RAP1B/EP300/JAK1/DAPK1/SMAD7/MAPK1/JAK2/MAP3K1/STAT3/I
FOXO1/MEF2A/PPP3CA/PPP3R2/PRKAA2/EP300/FOXO3/CREB1/MED1/ATF2/GSK3B/PRKAA1/PP
CTSV/CTSK/AP1S3/SEC23A/TUBB3/KIF18A/KIF20A/DYNC1LI2/SEC24A/CTSO/OSBPL1A/CENPE/DY
FGF23/FGF19/FGF2/FGF16/FGF7/PIK3CA/FGF10/FGF5/IRS1/FRS2/KRAS/NRAS/PIK3R1/PIK3R4/S
MMP1/ITGB1/CAV1/L1CAM/ITGA6/ITGA3/SLC16A1/SLC16A3/SLC7A5
BCAR3/DOCK9/ITSN1/DOCK11/APC/ARHGEF6/RALBP1/CDC42/RACGAP1/NGEF/ARHGAP1/DOCK
MAPK1/CDK1/BLZF1/RAB1A/GOLGA2/CCNB1/USO1/RAB2A/GORASP2
RASA1/MS4A2/PIK3CA/FER/PTPN13/CBL/PAK2/PIK3R1/FCER1G/WIPF1/SOS1/LCP2/FCER1A/CBL
THBS1/H2BC7/H3C14/H2BC8/H2BC6/H3C12/H2AC7/H2BC14/H2BC4/RUNX1/H2BU1/H4C8/EP3
F2R/TIMP2/IL1R1/PIK3CA/ICAM1/TLR1/TLR2/NLRP3/PLA2G7/CCR3/FCER1A
ROCK1/SPEN/RHOBTB1/ROCK2/PDE5A/VIM/SRRM1/DBN1/COPS2/MYO6/CUL3/RBBP6/CCT2/TR

TUBB3/STAG1/KIF18A/TAOK1/DYNC1LI2/CENPF/CENPE/DYNC1I2/CLIP1/KIF2A/KNL1/NUP160/CI
PIK3CA/PIK3CG/PIK3R1/CREB1/SP1/RB1/MAPK1/MAP3K1/MAPK14/MAP2K1
CXCL8/TLR4/IRAK3/TRAM1/TAB2/TRAF6/MYD88/MAP3K7/TBK1/NFKB1
REL/IL1A/OPRM1/GATA3/RIPK2/NCK1/CBL/FYB1/PIK3R1/CREB1/SOS1/LCP2/CBLB/ATF2/SHC1/M
MMP1/THBS1/MMP2/CXCL8/EGFR/KRAS/NRAS/E2F3/DAPK1/CDH1/RB1/VEGFA/MAPK1
MEF2A/DUSP7/CREB1/ATF2/ATF1/RPS6KA3/MAPK1/MEF2C/RPS6KA2/PPP2CB/MAPK14/ELK1/M
PIK3CA/EGFR/KRAS/ERBIN/NRAS/PIK3R1/SOS1/GAB1/SHC1/HSP90AA1
TLR8/TLR7/TLR3/DDX58/IFIH1/MR1
INPP4B/PIK3CA/PIK3CG/PTPN13/SBF2/MTMR2/PIK3R1/PIK3C2A/PLEKHA2/PTEN/PIK3R4/PLEKH
MYLK/MYH9/MYH10/PAK2/PPP1CB/LIMK1/PPP1R12A/FLNA/CDC42
TGFB2/CCN2/ADCY7/TMEM43/TGFB3/KRAS/NRAS/PLEC/SOS1/SMAD3/LEMD3/MAPK1/SYNE2/A
VDR/EP300/MED1/ACTL6A/COPS2/ARID1A/SMARCC2/CREBBP/NCOA1/SUPT16H/BAZ1B/SMARC
EREG/AREG/WNT5A/TGFA/SYT1/HIP1/EGFR/FCHO2/STON1/ACTR2/PICALM/IGF2R/CBL/ITSN1/E
FGF23/FGF2/FGF16/FGF7/PIK3CA/FGF10/FGF5/FRS2/KRAS/NRAS/PIK3R1/SOS1/GAB1
DRD4/MAP2K2/GJB1/PRKCB/GRIA1/GRM1/DRD2/ADCY8/GRIA2/PRKCG/PPP1R1A/GRIN1
SUOX/ETHE1/TXN2/CSAD/TSTD1/TST/MPST/SLC25A10/CDO1
PIK3CA/CHRNA1/KRAS/DOCK7/NRAS/PIK3R1/SOS1/SHC1/MAPK1/JAK2/STAT3/PIK3CB/MTOR/CI
IGKV1D-33/CD163/IGKV2D-28/LRP1/APOL1/IGKV5-2/IGLV3-25/IGKV2-28/JCHAIN/IGHV2-70/IGL
FGF23/FGFBP1/FGF2/FGF16/FGF7/PIK3CA/FGF10/FGF5/FRS2/KRAS/CBL/NRAS/PIK3R1/RBFOX2/
PIK3CA/CSF2/IL2RA/CBL/IL5RA/PIK3R1/YWHAZ/SOS1/JAK1/CSF2RA/SHC1/YES1/JAK2/PIK3CB/PT
LATS2/TEAD1/MAP4K4/STK3/STK38L/CXCL10/TNIK/LATS1/SAV1/MAP4K3/TEAD3/NDRG1/YWHA
APOC2/APOB/APOA1/CETP/APOA2/APOC3
CABP2/LRRC52/CABP1/RIPOR2/ESPNL/MYO15A/FSCN2/KCNMA1/MYO3A/WHRN/VAMP2/ATP2I
UGT2B10/GLYAT/UGT3A1/GLYATL1/CYP2D6/GLYATL2/UGT2B11/UGT1A4/UGT2B7/ACSM2B/CYI
PLCD4/NOS1/SLC25A6/SPHK2/ITPKA/PTGER1/ATP2B3/CACNA1C/CHP2/ATP2B2/PLCB4/PRKCB/C
CHST3/CHSY1/XYLT1/SLC26A2/EXT2/EXT1/BPNT2/PAPSS2
NR3C1/VDR/PPP3CA/SMAD3/MAP3K1/DUSP1/MAPK14/MED14/NFKB1/PPP3R1
DELEC1/BHLHE40/CLOCK/ATR/ARNTL/CHEK1/CRY1/NR1D1/NPAS2
PIK3CG/STAT1/SPRED1/KRAS/IL5RA/PIK3R1/FOXO3/SOS1/JAK1/GSK3B/SHC1/MAPK1/JAK2/STAT
WT1/EGFR/NR2F2/NBN/SP3/MXD1/ZNFX1/SP1/SMAD3/HSP90AA1/MAPK1/MTOR/ESR1/UBE3A
MAP2K2/CACNA1C/CHP2/PLCB4/PRKCB/GRIN2C/GRIA1/GRM1/CALML6/ADCY8/GRIA2/PRKCG/P
SUOX/AHCY/ETHE1/TXN2/CSAD/TSTD1/TST/MPST/SLC25A10/BHMT/CDO1/CBS/MAT1A
IGF2BP1/CDH2/ADAM17/BDNF/STAT1/IRS1/EGR2/NCF2/GRIP1/NCK1/CDKL5/MEF2A/NTF3/FRS2
FGF23/FGF2/FGF16/FGF7/PIK3CA/FGF10/FGF5/FRS2/PIK3R1/GAB1
UACA/PMAIP1/AKT3/YWHAZ/YWHAG/GSDME/MAPK1/TP53BP2/STAT3/BCL2L11/XIAP/TP73/CA
MTMR2/PIK3C2A/PIK3R4/PIKFYVE/MTM1/MTMR9
SERPINE1/SLC2A1/MMP14/TWIST1/BHLHE40/PGK1/FLT1/ETS1/EP300/SP1/VEGFA/SIRT1/ARNT/
GSTO2/CYP4F12/CYP17A1/GPX4/FMO5/NAT9/CYP3A5/SLX1A-SULT1A3/CYP4Z1/GAL3ST1/CYP2S
PIK3CA/PIK3CG/STAT1/PIK3R1/SOS1/JAK2/MAP3K1/MAP2K1
IL1RAPL2/IL1RAP/SLITRK3/PPFIA2/PPFIBP1/PTPRD/SLITRK4/IL1RAPL1/PPFIA4/SLITRK5/PPFIA1
FGF23/FGF2/FGF16/FGF7/PIK3CA/FGF10/STAT1/FGF5/FRS2/KRAS/NRAS/PIK3R1/LRRFIP1/SOS1/
LYPD2/CD109/NTM/LY6D/LYPD3/MELTF/NTNG1/LSAMP/RECK/LYPD4/MDGA2/CNTN4/PLAUR/A
COL5A1/CXCL8/PDGFRA/IL1A/CXCL9/IL2RA/STAT1/CD80/CD86/GDNF/C7/LRRK2/C4B/C1QC/HLA
PTGIS/IGFBP5/PTGS2/ALOX15B/PTGER3/ALOX5AP/PTGES/RB1/SIRT1/GNAQ/PTGER2/ADCY3
RASA1/ITPR2/ITPR3/KRAS/NRAS/VEGFA/SPHK1/PRKCA/ITPR1/AHCYL1/KDR
EREG/AREG/WNT5A/TGFA/SYT1/EGFR/FCHO2/STON1/PICALM/IGF2R/CBL/ITSN1/EPGN/DAB2/M
IL1A/TRAF6/AJUBA/UBE2V1/NFKB1
ABCC2/SREBF1/APOB/GSTM2/INS/GSTA1/SLC27A5/FOXA2/MLXIPL/GSTA2
ADAM17/PIK3CA/BDNF/BIRC3/PRKCI/RIPK2/NTF3/NTRK1/PIK3R1/MMP3/RTN4/MMP7/APP/BIR
LEPR/IRS1/JAK2/STAT3/PTPN11/SOCS3

EPS8/PREX2/RASGRF2/TRIO/SOS1/DOCK1/TIAM2/ARHGEF6/RALBP1/RACGAP1/NGEF/ARHGAP1,
ASL/ARG1/ARG2/OTC/CPS1
H2BC7/H3C14/H2AC13/H2BC8/H2BC18/H2BC6/H3C12/H2AC7/H2BC14/H2BC4/H2BU1/H2AC11/
CYP1A1/CYP2C19/ARNT2/CYP3A5/CYP2S1/CYP2D6/CYP2A6/CYP2A7/CYP2C9/CYP2E1/CYP1A2/C
ASAP2/ASAP1/ARAP2/CYTH3/ARFGEF1/ARFGEF2/ARFGAP3/COPA
FCGR2A/CAV1/ITGB3/PDGFRB/CDH2/NOX4/PIK3CA/LEPR/FER/EGFR/IRS1/PIK3R1/PDGFB/CSF1R
PICK1/CACNG8/PRKCB/GRIA1/TSPAN7/GRIA2/CACNG2/PRKCG/CAMK2B
ECT2/RASGRF2/TRIO/PLEKHG2/GNA13/ARHGEF12/ITSN1/AKAP13/NET1/FGD4/SOS1/TIAM2/ARI
ITGB1/LAMA3/LAMA2/LAMA4/CHRNA1/EGFR/AGRN/PAK2/UTRN/SP1/MUSK/MAPK1/ARHGEF6/
H2BC7/H2BC8/EYA4/H2BC6/H2BC14/H2BC4/H2BU1/H4C8/H2BC21/NBN/H2BC5/TP53BP1/RNF1
ABHD14B/ACSM1/UGT2B10/GLYAT/GSTM2/UGT3A1/GSTZ1/GLYATL1/GLYATL2/UGT2B11/SULT1
H2BC7/H2BC8/H2BC6/H2AC7/H2BC14/H2BC4/H2BU1/RSF1/H4C8/H2BC21/KNL1/H2BC5/CENPI/
BIRC3/NEDD4/HERC3/SMURF2/CBL/PIAS3/UBR5/UBE2E2/UBE4A/TRIP12/UBA6/BIRC6/ITCH/CBL
VAMP2/RAB3A/STXBP1/ABAT/GAD1/SLC32A1/SNAP25/CPLX1/SLC6A13
DKK1/FZD6/CSNK1A1/CTNNB1/TNKS2/DKK2/GSK3B/APC/PPP2R5E/AMER1/PPP2CB/DKK4/LRP6/
FGF23/FGF19/FGF2/FGF16/PIK3CA/FRS2/KRAS/NRAS/PIK3R1/SOS1/GAB1
MT1M/MT1F/SNCB/MT3/MT1H/MT1G
UNC5D/UNC5B/UNC5A/ABLIM3/TRPC3/SLIT2/UNC5C/ROBO1/TRIO/NCK1/TRPC1/NTN4/DOCK1/
HTR7/NR3C1/CREB1/ATF1/ELK4/MAPK1/EGR1/RAP1A/MAP2K1/ELK1
EREG/PIK3CA/EGFR/KRAS/PTPN12/ERBIN/NRAS/PIK3R1/SOS1/GAB1/SHC1/HSP90AA1
RAB31/CHML/DENND4C/SBF2/RIC1/RAB8B/RAB6A/AKT3/TRAPPC10/CHM/DENND5A/DENND2C,
FGF23/CYP24A1/DMP1/ROS1
TOMM5/PMPCB/SAMM50/TIMM9/HSCB/TOMM22/IDH3G/TOMM40/TIMM8B/TIMM10/BCS1L/
APOB/INS/IZUMO1R/SOD3/APOA1/SLC46A1/SAA2/HBB/GPX2/FGB/SAA1/IL4/CBS/GPX3/F7/MA
CYP2C19/CYP2S1/CYP2D6/CYP2A6/CYP2A7/CYP2C9/CYP2E1
FGF23/FGF2/FGF16/FGF7/FGF10/FGF5/FRS2/KRAS/NRAS/SOS1
NOX4/PIM1/PTPN11/BCL2L1/GAB2
PTGS2/CSF2/IL2RA/FOSL1/PPP3CA/NFATC3/NFATC2/POU2F1/RCAN1/PPP3R1
APOC2/APOB/APOA1/APOA5/APOA2/APOC3
PROC/F9/PROZ/F7/F2
ATP2B3/ATP2B2/SLC8A3/ATP2A3/SLC8A2
CTSV/HLA-G/LNPEP/CTSL/B2M
WEE1/YWHAZ/YWHAG/CDK1/CHEK1/YWHAQ/YWHAB/CCNB1
PIK3CA/EPHA2/CBL/PIK3R1/SHC1/RHOA/PTK2/PAK1/ARHGAP35/TIAM1/RAC1
MIR199A2/CXCL8/REL/TLR8/TLR4/IL1A/VCAM1/MIR125B1/ICAM1/TLR7/MIR146B/TAB2
ITGB1/ICAM1/ITGA4/CD44/ITGAM/ITGB2
PIK3CA/EGFR/KRAS/CBL/ITSN1/NRAS/PIK3R1/SOS1/CBLB/UBE2D1/EPS15/SHC1/RAB5A/PIK3CB/
SMC4/NEK7/TUBB3/STAG1/KIF18A/TAOK1/DYNC1LI2/CENPF/NEDD1/CENPE/DYNC1I2/CLIP1/KIF
AREG/COL7A1/TGFA/SEC23A/SEC24A/MCFD2/SEC24C/CD59/CTSC/SEC24D/SEC24B/LMAN1
ITGB1/ITGB3/ACTN1/EZR/TLN1/SPTAN1/RHOA/CAPN1
FCGR3A/ADAM17/MICB/CSF2/ADAM10/FOXO1/NCR3LG1/CD226
ANKRD1/ANGPTL4/ABCA1/CYP4A11/RORA/PLIN2/AHR/GRHL1/TNFRSF21/CHD9/GLIPR1/HMGCS
ITGB1/CLDN1/TLR4/TUBB3/ROCK1/NCK1/YWHAZ/CTNNB1/ROCK2/CDH1/OCLN/EZR/CDC42/WA
KRT14/IVL/CXCL8/GJA1/TCF4/TLR4/FOSL1/PRKCI/FOSL2/PRKAA2/NCOA2/RAP1B/TFAP2A/CTNN
DAO/PXMP2/SLC26A1/HOGA1/HAO1/AGXT/PRODH2
CXCL8/NR3C1/TGFBR2/TGFBR1/EP300/SMAD3/DUSP1/MAPK14/MYD88/MAP3K7/CREBBP/NFK
MIR199A2/FGF2/WNT5A/HDAC9/MYLK/PIK3CA/PIK3CG/PRKG1/MIR125B1/FZD1/ROCK1/MYLK3
SEMA3A/NRP1/PLXNA1/PAK2/LIMK1/HSP90AA1
ECHS1/ACADL/ACADVL/ACADS/ACSM6
H2BC7/H3C14/H2BC8/H2BC6/AR/H3C12/H2AC7/H2BC14/H2BC4/H2BU1/H4C8/NCOA2/H2BC21,

FGF23/FGF2/PIK3CA/STAT1/FGF5/PIK3R1/LRRFIP1/BAG4/STAT3
MYLK/RYR1/MYL1/ROCK1/ITPR3/PPP1CB/ROCK2/GNAQ
TEX15/H2BC7/H3C14/H2BC8/H2BC6/H3C12/H2AC7/H2BC14/H2BC4/H2BU1/H4C8/H2BC21/NBN
PIK3CA/TNFRSF10D/TNFRSF10A/PIK3R1/TNFSF10/MAPK1/MAP3K1/PIK3CB/TNFRSF10B/CASP8/(
MYL2/CLDN6/MYH8/CLDN1/CLDN11/MPDZ/CLDN16/PRKCI/ACTN4/EPB41L2/AMOTL1/MYH9/M
ITGAV/PTGS2/ITGB3/PDGFRB/PDGFB/GNAI3/VEGFA/MAPK1/SPHK1
SCN8A/SCN1B/SCN4A/SCN1A/CACNA1C/CACNG8/SCN3B/FGF14/RANGRF/CACNG6/FGF13/CACN
PIK3CA/EGFR/KRAS/CBL/NRAS/PIK3R1/SOS1/GAB1/SHC1/HSP90AA1
ALDH3A1/UGT1A5/GSTM4/AOX1/GSTO2/FMO5/ALDH3B2/CYP3A5/ADH7/UGT2B10/GSTM2/GS
SLC6A8/CKMT1A/CKMT1B/GATM/CKB/GAMT
PIK3CA/KRAS/ABL2/AKT3/CBL/NRAS/PIK3R1/FOXO3/SOS1/SOCS6/BCL2L11/SH2B3/PTPN11/SLA
SLC2A1/BCAT1/FOSL1/LDHA/MTDH/PMAIP1/ENO1/SNAI1/PIM1/HMGA1/EP300/NBN/E2F3/PDC
HSPA6/SERPINH1/HSPA1A/TNFRSF21/HSPH1/HSPA1B/HSP90AA1/DNAJB1/GML
IL2RA/CD80/CD86/IL6ST/STAT3/IL7R
PHC3/RYBP/MGA/RNF2/RBBP8/CHEK1/APAF1/RRM2/CBX3/TFDP1/BMI1/YAF2/RBBP4/TFDP2/BI
NEDD4L/CLCN2/FKBP1B/SCNN1B/TRPV1/CASQ1/RYR2/SLC9C2/SGK2/SLC9B1/CLCNKB/MCOLN2/
WT1/HSPA1A/LIN7A/RB1/JAK2/IFNGR1
EREG/PIK3CA/EGFR/KRAS/PTPN12/AKT3/ERBIN/NRAS/PIK3R1/SOS1/GAB1/SHC1/HSP90AA1/YES
E2F7/EP300/CNOT6/CDK2/CNOT1/PLK3/CCNE2/CDK1/CCNE1/TNKS1BP1/RBL2/CNOT8/CCNA2/C
STAR/HSD17B1/CYP17A1/CGA/HSD17B14/FDXR/HSD17B3/HSD3B1/FDX2/TSPOAP1/HSD11B2/SI
FGF19/SNAI2/ITGB1/FGF2/DMBX1/NOTCH2/HDAC9/CDH2/MITF/ZIC1/TCF4/CDH6/PRTG/PMP22
MMP1/THBS1/MMP2/CXCL8/EGFR/KRAS/NRAS/PIK3R1/DAPK1/CDH1/RB1/VEGFA/MAPK1/HBE(
H2BC7/H3C14/H2AC13/H2BC8/H2BC18/H2BC6/H3C12/H2AC7/H2BC14/H2BC4/H2BU1/H2AC11/
IL1R1/IL1A/TNFAIP3/TRAF6/MAP3K1/MYD88/MAP3K7/NFKB1/CHUK/RELA/TNFRSF1A/TNF/TNFI
SMC4/RASA1/MYLK/KIF20A/KIF23/EVI5/BUB1/INCENP/TACC1/SMC2/VIM/RACGAP1/SGO1/RHO
CSNK1A1/CTNNB1/GSK3B/APC/PPP2R5E/AMER1/PPP2CB
FGF23/FGFBP1/FGF2/FGF16/FGF7/FGF10/FGF5
PDGFRB/KIT/PDGFRA/FLT1/PIM1/CSF1R/FOXO3/SPRED2/ABL1/GAB2/GADD45A
MMP1/IFNL3/AREG/IL36RN/IFNL1/IFNK/IFNL2/CCL13/CXCL8/CXCL5/IL25/IL11/CSF2/IL1A/CXCL9
GALR1/GPR35/RAMP2/DRD4/SST/AVP/RXFP4/OPN1LW/GABBR2/TAS2R4/CGA/RLN3/FZD10/RAI
PARP14/CSNK1A1/PARP9/PARP4/GSK3B/SRPK2/PARP8/SRPK1
MEF2A/DUSP7/RPS6KA3/MAPK1/MEF2C/RPS6KA2/PPP2CB/MAPK14/ELK1
TLR4/IRAK3/IRAK2/SFTPA1/NOD2/RIPK2/MEF2A/TLR1/TLR2/IKBIP/TAB2/DUSP7/CREB1/FGG/AP
PIK3CA/PIK3CG/IL2RA/IRS1/CBL/PIK3R1/SOS1/JAK1/SHC1/MAPK1/FAS/CFLAR/NMI/SOCS3/RPS6
MORC3/SETX/AEBP2/JARID2/THRAP3/MTF2/BCLAF1/RBBP4/STK38/SUZ12
FN1/ITGB3/QKI/IQGAP1/VCL/KRAS/NRAS/RAP1B/FGG/MAPK1/FXR1/JAK2/BCL2L11/MPRIP/RAP
PIK3CA/PIK3CG/TWIST1/PIK3R1/RB1/CDKN2A/MDM2/ABL1/POLR1A
NRG1/DLG4/LRRC7/NRGN/GRIN2C/GRIA1/GRIA2/GRIN1/CAMK2B
PIK3CA/PIK3CG/PIK3R1/RALA/RALBP1/CDC42/MAP2K1/ELK1/PLD1/NFKB1/RHOA
H2BC7/H3C14/H2BC8/H2BC6/H3C12/H2AC7/H2BC14/H2BC4/H2BU1/CAST/H4C8/SOD2/H2BC21
PLAT/PLAU/SERPINE1/COL4A1/COL4A5/COL4A4/COL4A2
CCKBR/GAST
RORA/CHD9/NCOA2/EP300/MED1/TBL1XR1/CPT1A/CREBBP/NCOA1/NCOA6/CARM1/TGS1/HEL
TGFB2/TLR8/TLR4/CD274/IL2RA/TLR7/TGFBR2/CD80/CD86/TGFB3/PIAS3/CXCL10
PHC3/STAG1/NUP160/NUP153/RNF168/SP100/STAG2/RAD21/SMC5/RNF2/NDC1/NUP107/SMC
IL20RB/IFNL3/IFNL1/IFNL2/STAT1/IL22RA2/JAK1/STAT2/JAK2/STAT3/PTPN11
NT5E/IL1A/MEFV/C3AR1/P2RX7/NLRP3/APP/ENTPD1/HMOX1/C3/TXNIP/IL18/NFKB1
DKK1/H2BC7/H3C14/WNT5A/H2BC8/CAV1/PYGO1/DACT1/H2BC6/TCF4/BCL9L/FZD6/H3C12/SFI
FGF23/FGF2/FGF16/PIK3CA/FGF5/FRS2/PIK3R1/GAB1
TGFB2/CXCL8/IL11/CSF2/IL1A/TGFB3/IFNA1/IL7/CD4/CSF1/HLA-DRA

IRAK2/BIRC3/NOD2/RIPK2/TAB2/TNFAIP3/ITCH/BIRC2/TRAF6/TAB3/NOD1/MAPK14/MAP3K7/C
H2BC7/H3C14/H2BC8/H2BC6/H3C12/TET1/H2AC7/H2BC14/H2BC4/H2BU1/H4C8/DDX21/UHRF1
VAMP2/RAB3A/SLC18A3/STXBP1/ABAT/SLC1A6/TSPOAP1/GAD1/SYN1/LIN7B/SLC32A1/GLS2/PP
F5/FGB/F11/C4BPB/SERPINA1/C8G/F9/C8A/KNG1/C5/SERPINF2/CFD/F12/CPB2/F13B/SERPINC1
NFAT5/PIK3CA/PIK3CG/CSF2/NCK1/KRAS/PPP3CA/AKT3/CBL/PAK2/PPP3R2/NRAS/PIK3R1/SOS1/
PIK3CA/FRS2/KRAS/NRAS/PIK3R1/SOS1/GAB1
MMP2/PIK3CA/MSN/KRAS/GNA13/STRN/NRAS/PIK3R1/SOS1/ROCK2/GNAI3/SHC1/GNB1/IGF1R
ABCA1/RARB/VDR/NR1H4/MED1/PPARD/RARG/NR4A1/PPARG/NCOA1/RPS6KB1/THRB
NT5C/DPYS/UPB1/GLS2/OTC/CPS1
H2BC7/H3C14/H2BC8/NOTCH2/MAML2/H2BC6/H3C12/H2AC7/H2BC14/H2BC4/PRKCI/RUNX1/B
BIRC3/BIRC2/LMNB1/XIAP/CASP8/APAF1/DFFA/CASP4/CASP2
ITGB1/H2BC7/H3C14/H2AC13/H2BC8/H2BC18/H2BC6/H3C12/H2AC7/H2BC14/TUBB3/H2BC4/E
SNAI2/MITF/KIT/STAT1/KITLG/CBL/SNAI1/PIK3R1/EP300/FOXO3/SOS1/SOCS6/SHC1/RPS6KA3/
FGF23/FGF2/FGF16/FGF7/FGF10/FGF5
PPP3CA/SP3/SP1/NFATC3/GNAQ/NFATC2/MARCKS/PRKCA
TIMP3/RB1/CDK2/CCNE1/MDM2/APAF1/CCND1/GADD45A/ATM
NBN/TOPBP1/RBBP8/ATR/ERCC4/WRN/BARD1/ABL1/BRIP1/RAD50/RFC3/MRE11/ATM/RMI1/B
KCNQ1/CACNA1C/CACNG8/CACNG6/KCNE2/CACNA2D2/CACNB2/CACNG7
CD80/CD86/NCK1/KRAS/CBL/PAG1/FYB1/NRAS/PTEN/SOS1/LCP2/RASGRP1/FLNA/SHC1/TRAF6/
EREG/ADAM17/PIK3CA/YAP1/NEDD4/PIK3R1/TAB2/ITCH/WWP1/SHC1/MAPK1/JAK2/PIK3CB
SLC2A1/DIO2/PIK3CA/PIK3CG/MAP4K5/IRS1/MAP4K4/FOXO1/MAP3K2/CBL/PRKAA2/PIK3R1/C
PIK3CA/STAT1/GNA13/PIK3R1/RAP1B/CREB1/JAK1/GNAI3/RB1/CDK2/MAPK1/GNB1/JAK2/STAT
STAT1/IL6ST/JAK1/JAK2/STAT3/CANX
FGF23/FGF2/FGF16/FGF7/FGF10/FGF5/FRS2/CBL
P2RX2/P2RX6/TRPC7/P2RX1/P2RX5
DGKZ/CHKB/DGAT1/PTDSS2/PNPLA2/PCYT2/DGAT2/PEMT/PLA2G1B
MT1X/MT1B/MT4/MT1E/MT1M/MT1F/MT3/MT1H/MT1G
TGFB2/STAT1/TGFBR1/CREB1/ATF2/SHC1/MAP3K1/CDC42/MAPK14/MAP3K5/MAP3K7/ELK1
MITF/KIT/PIK3CA/PREX2/VCL/CDK6/KRAS/AKT3/NRAS/POLK/PIK3R1/ETS1/NF1/CALML5/GRIN2
GOT1/ASL/ABAT/GAD1/GPT/AGXT
IL25/IRAK2/NOD2/RIPK2/MEF2A/TAB2/DUSP7/CREB1/ATF2/ATF1/RPS6KA3/MAPK1/TRAF6/TAB
FGF23/FGF19/FGF2/FGF16/PIK3CA/FRS2/KRAS/CBL/NRAS/PIK3R1/SOS1/GAB1/SHC1/MAPK1
IL6ST/SOS1/JAK1/SHC1/JAK2/STAT3/PTPN11/MAP2K1/ELK1/CEBPB/FOS/CSNK2A1
APOC2/APOA1/CETP/ALB/APOC3
HTR3A/PICK1/KCNJ4/GNG8/TUBB8/CACNG8/PRKCB/NRGN/GNB3/GRIN2C/TUBA8/GABRB3/TUB
H2BC7/H3C14/H2BC8/H2BC6/H3C12/H2AC7/H2BC14/H2BC4/H2BU1/H4C8/UHRF1/H2BC21/H2
CYP4F22/PTGIS/CYP4A11
BHLHE41/BHLHE40/CLOCK/ARNTL/PER3/CRY1/NR1D1/NPAS2
SLC22A2/SLC22A3/NT5C2/ADH1A
SLCO1B3/SLCO2B1/SLCO3A1/SLCO1A2/SLC16A2/SLCO4C1/SLCO2A1/SLCO1C1/SLCO1B1
IL1RAP/IL1RL1/IL36RN/PTPN14/IL1R1/IL1A/IRAK3/IL36B/IRAK2/NOD2/RIPK2/IL1RAPL1/PTPN13/
H2BC7/H3C14/H2BC8/H2BC6/H3C12/H2AC7/H2BC14/H2BC4/H2BU1/H4C8/H2BC21/H2BC5/H2
CCL13/CXCL8/CXCL5/CCR8/GNB4/PIK3CA/PIK3CG/CXCL9/CXCR2/PF4V1/GNG12/STAT1/GNGT1/
ALDH3A1/UGT1A5/GSTM4/GSTO2/CYP4F12/ACSS2/MTARC1/ACY1/ABHD14B/CYP3A5/ACSM1/
ST8SIA2/ST8SIA6/B4GALT5/B4GALT1
MC3R/CCR10/MLN/CCL19/SSTR3/GPER1/AGT/ECE2/GPR37L1/RLN2/TACR1/CCL21/SSTR2/KEL/
GGT6/GPX2/PLA2G6/PLA2G10/PLA2G2D/CYP2C9/CYP2E1/PLA2G12B/EPHX2/LTC4S/GPX3/ALOX
F5/FGB/CLU/SERPINA1/C8G/F9/KNG1/SERPINF2/CFD/F12/CPB2/F13B/SERPINC1/F7/CR2/KLKB1/
THBS1/H2BC7/H3C14/CTSV/H2BC8/RUNX2/CTSK/H2BC6/CSF2/H3C12/IL2RA/YAP1/H2AC7/H2B
WNT5A/GNB4/PRKG1/FZD6/ITPR2/GNG12/GNGT1/ITPR3/LEF1/KRAS/PPP3CA/CTNNB1/WNT11/

FN1/ITGB3/RAP1B/SOS1/FGG/RAP1A/TLN1
TGFB2/MET/RUNX2/MITF/CSF2/CDK6/EP300/ELF1/E2F3/ATF2/RB1/ATF7/CDK2/SIRT1/MEF2C/M
H2BC7/H3C14/H2BC8/H2BC6/H3C12/H2AC7/H2BC14/H2BC4/H2BU1/H4C8/H2BC21/H2BC5/H2A
STAG2/SGO2/APC/RAD21/PPP2R5E/PDS5B/PPP2CB/SMC3/SMC1A/CDK1/NIPBL/PDS5A/WAPL/E
PIK3CA/IRS1/AKT3/PIK3R1/SOS1/JAK1/GSK3B/SHC1/MAPK1/SOS2
GJA1/TCF4/ROR1/CDK6/LEF1/CSNK1A1/CTNNB1/RYK/ROR2/GSK3B/APC/MAPK1/MTOR/NFATC2
PIK3CA/IRS1/PIK3R1/PTEN/EIF4G3/MAPK1/MTOR/EIF4G2/MAPK14/EIF4G1/PRKCA/EIF4E/RPS6K
DKK1/ADAM10/NEDD4/FZD1/CSNK1A1/CTNNB1/DKK2/GSK3B/APC/PPARD/MAPK1/PSEN1/NKD
HMGA2/RUNX2/CDK6/LEF1/CSNK1A1/CTNNB1/SPP1/AGO2/GSK3B/APC/TOR1AIP1/DICER1/ICM
FFAR3/MC3R/CCR10/MLN/CCL19/SSTR3/GPER1/AGT/ECE2/PTGDR2/GPR37L1/P2RY11/RLN2/GN
APOC2/APOB/MIR33A/APOA1/ABCG5/CETP/ACSS1/APOA5/APOA2/PDIA2/APOC3
STON1/TAF1L/GTF2I/GTF2A1/TAF2/GTF2H2/GTF2E1/TAF7L/TAF1/GTF2H3/TAF4B/GTF2H1/TAF1
PLAT/PLAU/SERPINE1/SERPINB2/PLAUR/SERPINB8
ALDH3A1/UGT1A5/GSTM4/GSTO2/ALDH3B2/CYP3A5/ADH7/CYP2S1/UGT2B10/GSTM2/GSTZ1/A
SLC2A1/RRAD/PIK3CA/PIK3CG/MAP4K5/IRS1/PRKCI/MAP4K4/FOXO1/CYTH3/MAP3K2/CBL/PRKA
CDK6/RUNX1/AGO2/CBFB/PTPN11/TNRC6B/AGO3/AGO4/CCND1/PML/AGO1/TNRC6A
CAV1/CCNG2/FOXO1/FOXO3/SMAD3/FOXG1/RBL2/SMAD2/GADD45A
CXCL8/PIK3CA/PIK3CG/RETN/AKT3/MAPK1/PIK3CB/MAPK14/NFKB1/ITPR1/AKT2
FABP6/FABP4/FABP2/ACSL6/FABP7
IL6ST/JAK1/JAK2/PTPN11/MAP2K1/CDK1
CTDSP2/NUP153/SMAD3/MAPK1/MAP3K1/SMAD2/CTDSPL/KPNB1/PPM1A
IFIT2/CXCL9/GBP1/ICAM1/STAT1/EIF2AK2/CXCL10/JAK1/CYBB/STAT2/JAK2/IFNGR1/PTPN11/OA
SERPINE1/ARNTL2/BHLHE41/NAMPT/BHLHE40/CHD9/NCOA2/NOCT/MED1/CLOCK/TBL1XR1/AR
ADH1C/PDK4/ARG1/ARG2/ASNS/ACSS1/MPST/PNMT/TPH1/BHMT/GPT2/TAT/CBS/DDC/OTC/CP
SGMS2/ARSI/ARSJ/CERS6/SPTLC1/STS/ARSB/PRKD3/B3GALNT1/ACER2/SGPP2/B4GALNT1/ESYT2
ALDH2/CYP1A1/ALDH1A1/ADH7/CYP2D6/ADH1C/CYP2A6/ADH6/CYP2E1/CYP1A2/CYP3A4
LIPC/CHKB/GCDH/ACSS2/CRAT/PNPLA2/ECHS1/ACADL/ACAT1/ACADVL/CPT1B/LIPE/ACADS/ECI1
FRS2/KRAS/KIDINS220/NTRK1/RALB/NRAS/SOS1/RALA/SHC1/MAPK1/RIT1/MAPK14/RAP1A/MA
PIK3CA/IL2RA/PIK3R1/UGCG/FOXO3/SOS1/JAK1/SHC1/HSP90AA1/MTOR/PTPN11/SGMS1/RPS6I
H2BC7/H3C14/H2BC8/H2BC6/H3C12/H2AC7/H2BC14/H2BC4/H2BU1/H4C8/H2BC21/H2BC5/H2A
ADAM17/ECT2/RASGRF2/TRIO/PRKCI/RIPK2/PLEKHG2/GNA13/ARHGEF12/ITSN1/AKAP13/NET1/
BEND6/TLR8/NCF2/GTF2I/ARHGAP31/SP1/CYBB/EOGT/MAPK1/TLR9/SHCBP1/ESR1/CDC42
SEMA3A/NRP1/DPYSL3/PLXNA1/GSK3B
APOC2/LRAT/APOB/AKR1C4/APOM/TTR/APOA1/APOA2/APOC3/PNLIP/CLPS
GAS1/HHIP/EVC/GLI2/GLI3/GLI1/GPR161/SMURF2/CSNK1A1/SPOPL/CDC73/BOC/ITCH/SMO/EV(
PIK3CA/PIK3R1/PTEN/EIF4G3/MTOR/EIF4G2/EIF4G1/SHLD2P3/EIF4E/RPS6KB1/EIF3A
CA9/CA14/CA4/CA6
GRIA3/DLG4/LRRC7/GRIN2C/GRIA1/GRIA2/GRIN1/CAMK2B
FN1/ITGB3/RAP1B/SOS1/FGG/RASGRP1/SHC1/RAP1A/PTPN1/TLN1
NOG/FSTL1/BMPR2/BMPR1B/SMURF2/SMAD1/ZFYVE16/UBE2D1/SMAD7/SKI/SMURF1/SMAD5/
MET/ITGB3/GULP1/HGF/EPHA2/EGFR/ARAP2/NCK1/CYTH3/ACAP2
KCNA5/KCNA1/KCNH5/KCNH8/KCNQ1/KCNG2/KCNAB3/KCNC1/KCNF1/KCNG1/KCNV2/KCNV1/K
MUC6/CCK/PGA5/GAST
CACNA2D3/CACNA1B/CACNA2D2/CACNB2/CACNA1A/CACNG2
FPR2/PIK3CA/PIK3CG/EPHB2/PDK1/PIK3CB/MTOR
PICK1/PRKCB/GRIA1/TSPAN7/GRIA2/PRKCG
AR/NR3C1/RORA/VDR/PIAS3/NR1H4/NR4A2/ESR1/PGR/PPARG/PIAS1/SUMO3/THRB
KCNJ4/KCNK15/KCNK7/KCNK16/KCNK12/KCNK10/KCNK3
PEX7/PAOX/ACOT8/HAO2/CRAT/PEX6/DAO/DDO/MLYCD/RPS27A/DECR2/ECI2/UBA52/TYSND1/
PIK3CA/PIK3CG/HIF1A/FLT1/PIK3R1/SHC1/VEGFA/ARNT/PRKCA/EIF2S1

PIK3CA/PIK3R1/PTEN/GSK3B/IGF1R/MTOR/IGF1/EIF4E/RPS6KB1/EIF2S1
SGMS2/B4GALT1/B3GALNT1/SGPP2/B4GALNT1/UGCG/SPHK1/SGPP1/GALNT1/UGT8/KDSR/SGM
FOXL2/MITF/TFAP2C/PIAS3/TFAP2A/SP3/TP53BP1/CDKN2A/MDM2/PIAS1/SUMO3
FGF23/FGF2/FGF16/FGF5/FRS2/KRAS/NRAS/SOS1
P4HB/GPIHBP1/LIPC/LMF1/APOF/ANGPTL3/CREB3L3/LMF2/APOC2/APOB/APOA1/ANGPTL8/CE
ATP5MG/ATP5PD/TMEM11/CHCHD6/ATP5F1A/ATP5MF/ATP5PF/APOO/ATP5F1E/ATP5MC2/AT
PIK3CG/BDNF/TRPC3/HOMER1/KRAS/NRAS/CREB1/SOS1/GAB1/SHC1/MAPK1/MTOR/MAP2K1/
NTF3/KRAS/MAP3K2/NRAS/RAP1B/CREB1/MAPK1/EGR1/RIT1/MEF2C/MAPK14/RAP1A/EHD4/M
PEX7/PAOX/ACOT8/HAO2/CRAT/PEX6/MVK/DAO/DDO/MLYCD/PEX16/DECR2/ECI2/PXMP2/DH
ECT2/RASGRF2/TRIO/PLEKHG2/GNA13/ARHGEF12/ITSN1/AKAP13/NET1/FGD4/SOS1/TIAM2/AR
TMOD3/ACTN1/HSP90AA1/PHIP/TWF1/SRRM1/DBN1/MYO6/CUL3/CCT2/TRA2B/HSP90AB1/HN
DBP/IL17A/AKR1C4/IL17B/RORC/SLC27A2/SLC27A5/LBP/EPHX2/CYP3A4/CYP46A1/IL17F
GCKR/GPIHBP1/LIPC/LMF1/APOC2/DGAT2/APOA1/CETP/ACSS1/APOA5/GCK/APOA2/PDIA2
GPX4/GSTM2/ANPEP/GSTA1/GPX2/GSTT2/GPX3
PIK3CA/PRDM1/HIF1A/CD274/IRS1/FRS2/PIK3R1/EP300/SHC1/STAT3/PIK3CB
NBN/BRCA2/RBBP8/RAD51AP1/WRN/BARD1/PALB2/BRIP1/RAD50/MRE11/ATM/RMI1/BRCA1/
TGFB2/PIK3CA/PIK3CG/TGFBR2/TGFB3/TGFBR1/PIK3R1/SMAD1/PTEN/MTOR/SMAD5/CDKN2A/
BACH1/TLR4/NRIP1/RORA/CLEC1B/CHD9/NCOA2/EP300/CREB1/MED1/ATF2/NFE2L2/HMOX1/C
TEX15/H2BC7/H3C14/H2BC8/H2BC6/H3C12/H2AC7/H2BC14/H2BC4/STAG1/H2BU1/H4C8/H2BC
PMAIP1/AKT3/YWHAZ/YWHAG/TP53BP2/BCL2L11/TP73/YWHAQ/BMF/AKT2/YWHAB/MAPK8/P
NBN/BRCA2/TOPBP1/RBBP8/ATR/RAD51AP1/WRN/BARD1/CHEK1/PALB2/BRIP1/RAD50/RFC3/
RASA1/PTPRU/PIK3CG/STAT1/IRS1/PDK1/SOS1/SHC1/MAPK1/JAK2/STAT3
H2BC7/H3C14/H2BC8/H2BC6/H3C12/H2AC7/H2BC14/H2BC4/H2BU1/H4C8/H2BC21/H2BC5/H2
NOTCH2/MAML2/MFAP2/ADAM10/LEF1/NOTCH3/JAG1
AFMID/MTHFD2L/HAO2/PGP/MDH2/HYI/GLYCTK/HAO1
PTGIS/PTGS2/NT5E/NAMPT/BST1/PARP14/PARP9/PARP4
NEK7/NUP160/NUP153/NEK6/NDC1/NUP107/CDK1/NUP58/NUP205/NUP98/TPR/RANBP2/CCN
ASAP1/PKD2/CNGB1/ARL13B/RP2/EXOC5/MCHR1/SMO/ARF4/RHO/BBS9/BBS10/EXOC1/ARL6
DNMT3L/MPST/LDHC/BHMT/CDO1/TAT/CBS/MAT1A
ATP5MG/ATP5PD/ATP5F1A/ATP5MF/ATP5PF/ATP5F1E/ATP5MC2/ATP5PO/ATP5MC1/ATP5ME/
FGF2/BDNF/OPRK1/TET1/NREP/FGF5/IGF2/REST/BCL6/FKBP5/NF1/SP3/PTEN/CREB1/TET2/SP1/
PRDM1/NLRP10/MEFV/NOD2/RIPK2/NLRP2/NAIP/NLRP12/ERBIN/NLRP3/NLRC4/HSP90AA1/CA
MAPK10/SOCS1/CACNA1C/INS/SLC2A4/CACNA1B/PIK3R2/PDX1/IRS4/GCK/KCNJ11/CACNA1A/A
ITGB1/PIK3CA/PIK3R1/KLRD1/KLRC1/MAP2K1/B2M/KLRC4/IL18
TSHB/CGA/FSHB/GNRH2/GPHA2
TUBB8/TTLL2/TUBA8/TUBAL3/TUBA3C/AGBL1/TUBA3E/TPGS1/AGBL4/TTLL6
TTC19/UQCRFS1/UQCRH/UQCC1/BCS1L/UQCRC1/CYC1/UQCRB/UQCR10/UQCC2/UQCRQ/UQCC
APOC2/APOB/APOA1/APOA2/APOC3
PANX1/MEFV/P2RX7/NLRP3/APP/HMOX1/TXNIP/NFKB1
FGF23/ANOS1/FGF2/FGF10/FGF5
VCAN/DCN/HSPG2/GPC6/AGRN/BGN/GPC4/SDC4
CDH11/CLDN6/CDH3/CDH2/CLDN1/CLDN11/CDH6/NECTIN3/CLDN16/PRKCI/CDH13/PARD6B/CL
VCAN/DSE/DCN/DSEL/BGN
SLC39A3/MT1M/MT1F/SLC30A2/MT3/MT1H/MT1G/SLC39A5
FN1/AREG/FOSL1/NRIP1
SERPINH1/ULK4P2/KLF13/GOLGA8R/ULK4P1/CREBBP/GOLGA8Q
PTGS2/CXCL8/IL1R1/CSF2/IL1A/FPR1/ICAM1/CCR1/CD80/CD86/CCR2/IL1RN/CXCL10/JAK1/STAT
KRAS/NRAS/MRAS/MAPK1/RAB5A/PDCD6IP
FGF23/FGF2/FGF16/FGF5/KRAS/NRAS/SOS1/SHC1
EPHA3/MMP2/EPHB6/EPHA2/ADAM10/EPHA4/EFNA5/EPHB2/EFNB2/CLTC/EPHA6/YES1/EPHA5

OLIG1/MOG/CXCL2/NKX2-2/NKX2-6/MAG/MYT1/ASCL1/SOX8
APOA1/APOA5/APOA2/KLK15/APOC3
PIK3CA/AKT3/NTRK1/SOS1/SHC1/MAP2K1/ELK1/AKT2/RHOA
FRS2/KIDINS220/NTRK1/MAPK1/RAP1A/MAP2K1/YWHAB/BRAF/CRK
PRKDC/PAK2/RB1/BAG4/MAP3K1/LMNB1/MAP3K7/CASP8/SPTAN1/MAPK8/DFFA/CASP2/PAK1/
PRDM1/NUAK1/TAF1L/AKT3/PRKAA2/HIPK1/KAT6A/EP300/NBN/TAF2/PPP1R13L/PHF20/PRKAA
IMPDH2/GGT7/SULT1A1/ABCC2/NME1/UGT1A5/BSG/VAV3/ACY1/SLC29A2/NME2/UGT2B10/GL
GH1/IGFBP2/IGFALS/GHRH
BDNF/FRS2/KRAS/NRAS/SOS1/PTPN11/NTF4
H2BC7/H3C14/H2BC8/H2BC6/H3C12/H2AC7/H2BC14/H2BC4/H2BU1/H4C8/DDX21/EP300/H2B
TRPC3/GUCY1A2/GUCY1A1/KCNQ5/TRPV4
KCNJ2/KCNJ8/KCNJ14/GNG13/GNG2/GABBR2/KCNJ4/GNG8/GNB3/GNG4/GNG7/KCNJ10/KCNJ6/
ATP2B3/CACNA1C/GNG8/GJB1/ATP2B2/PRKCB/GNB3/CACNA1B/PRKAR1B/GNG4/CACNA1S/GN
NR3C1/NF1/GTF2A1/ARID1A/SMARCC2/SMARCC1
PIK3CA/PIK3CG/KRAS/NRAS/PIK3R1/SOS1/GAB1/SHC1/JAK2/PIK3CB/PIK3R5
SEC23A/INSIG2/CHD9/SEC24A/HMGCS1/NCOA2/MBTPS2/SEC24C/MED1/SP1/SEC24D/SEC24B/
EREG/AREG/TGFA/PIK3CA/PIK3CG/EGFR/NCK1/KRAS/ABL2/AKT3/CBL/PAK2/NRAS/PIK3R1/SOS1
GGT7/CSAD/GGT6/GAD1/CDO1
CHRNA2/CHRNA4/CHRNB2/CHRNB3
HSD17B10/ACSM1/CYP2A6/ABAT/CYP2C9
FGF23/FGF19/FGF2/FGF16/FGF7/PIK3CA/FGF10/FGF5/IRS1/FRS2/KRAS/NRAS/PIK3R1/PIK3R4/S
SMC4/SMC2/CDK1/NCAPG/CCNB1/NCAPH/CSNK2A1
DHPS/DPH1/DPH7/PROC/BGLAP/ARSL/DOHH/FN3K/TPST2/F9/PROZ/F7/F2
RASA1/EGFR/CBL/SOS1/SHC1/MAPK1/PTPRB/MAP2K1/SPRY3/SPRY4/SPRY1
CD80/CD86/AKT3/YES1/PPP2R5E/PTPN11/PPP2CB
AP1S3/PICALM/IGF2R/SH3D19/M6PR/PIK3C2A/CLTC/TBC1D8B/ACBD3/APP/SNX9/PUM1/CPD/S
PLCD4/ARC/PRKCB/GABRA1/GRM1/POMC/CAMK2B
DLG4/ACTA1/NRXN3/ANK1/NFASC/PCLO/NCAM1/GRIN1
ASAP2/FCGR2A/FCGR3A/PIK3CA/PIK3CG/ASAP1/AKT3/PIK3R1/LIMK1/PLPP3/WASF2/MAPK1/PI
FBXO11/SETD7/CSNK1A1/EP300/PPP1R13L/UBE2D1/GSK3B/CDK2/CSNK1G3/HUWE1/ATR/MAP
PLAT/PLAU/SERPINE1/F2R/F13A1/SERPINB2
HSD17B7/HSD17B1/CYP17A1/HSD17B3/HSD3B1/CPN1/F13B
NEK7/VRK2/NUP160/NUP153/LEMD3/LMNB1/NEK6/NDC1/NUP107/CNEP1R1/CDK1/NUP58/NU
FGF23/FGF2/PIK3CA/FGF10/FGF5/FRS2/PIK3R1/GAB1
FCGR2A/REL/F3/NLRP3/CGAS/TBK1/NFKB1/ITPR1/STING1/IFNB1/CHUK
GSTM4/GPX4/CYP2D6/CYP2C9/CYP2E1/EPHX2/LTC4S/CYP1A2/CYP3A4
FN1/HIP1/STRN/BIRC6/CLTC/PRKAR1A
ZP1/ZP3/ZP2/SPAM1/OVGP1/ADAM2
FGF23/FGF19/FGF2/FGF16/PIK3CA/FRS2/PIK3R1/GAB1
PIK3CA/PIK3CG/PIK3R1/SOS1/SHC1/MAP2K1/ELK1/MAPK8
ATP12A/ATP1A4/ATP2B3/ATP2B2/FXYD1/ATP13A5/ATP8A1/FXYD2/FXYD4/ATP1A2/FXYD7/ATP
CXCL5/CCR8/GNB4/PIK3CA/PIK3CG/CXCL9/CXCR2/GNG12/STAT1/GNGT1/CCR1/ROCK1/ADCY7/
PIK3CA/EGFR/KRAS/CBL/NRAS/PIK3R1/SOS1/GAB1/SHC1/HSP90AA1
C5/CFD
EPOR/GRIN1/EPO
H2BC7/H2BC8/EYA4/H2BC6/H2BC14/H2BC4/RIF1/H2BU1/CLSPN/PRKDC/H4C8/POLK/H2BC21/
FGF23/ANOS1/FGF2/FGF10/FGF5/FRS2/CBL
ABLIM3/TRIO/NCK1/DOCK1/CDC42/DCC/WASL
SELL/PTCRA/IL21/PDCD1/FCRL5/BLNK/CCL19/CCL21/IL4/CCR7/AICDA/CXCR5/CR2/CD79A/CD22/
EPHA3/EPHA2/EPHA4/EFNA5/ROCK1/MYH9/MYH10/ROCK2/EPHA6/YES1/EPHA5

FGF2/PIK3CA/PIK3CG/CAMK1G/PPP3CA/CSNK1A1/PIK3R1/EDN1/NFATC3/GSK3B/MAPK1/HAND
SOS1/JAK1/SHC1/STAT3/SOS2/IL15RA/GAB2/IL15
MMP1/PTGS2/NFE2L2/VEGFA
LEF1/KRAS/NTRK1/NRAS/CTNNB1/CDH1/MAPK1/NCOA4/CCDC6/MAP2K1/PPARG/CCND1/TCF7L
UGT1A6/UGT1A10/UGT2B28/UGT1A3/UGDH/UGT1A8/UGT1A1
H2BC7/H2BC8/H2BC6/H2AC7/H2BC14/H2BC4/STAG1/H2BU1/H4C8/H2BC21/H2BC5/H2AC6/H4[illegible]
FGF2/PIK3CA/EGFR/LEF1/KRAS/AKT3/NRAS/POLK/PIK3R1/FOXO3/CTNNB1/PTEN/SOS1/CDH1/G[illegible]
SLC8A3/SLC24A5/SLC8A2
PIK3CA/PIK3CG/SBF2/MTMR2/PIK3C2A/PTEN/PIK3R4/PIK3CB/PIKFYVE/PIP4K2A/MTMR6/PIP5K[illegible]
WNT2/WNT5A/WNT7A/FZD6/WNT7B/WNT10A/FZD1/FZD7/CTNNB1/WNT11
KIF18A/TAOK1/DYNC1LI2/CENPF/CENPE/DYNC1I2/CLIP1/KIF2A/KNL1/NUP160/CENPI/CKAP5/SP
LPAR4/P2RY1/P2RY2/ADORA2B/P2RY6/P2RY4
H2BC7/H2BC8/H2BC6/H2AC7/H2BC14/H2BC4/H2BU1/H4C8/H2BC21/H2BC5/H2AC6/H4C12/H2[illegible]
RARB/NRIP1/VDR/NCOA2/EP300/NCOA3/MAPK1/RARG/MAPK14/CDK1/CREBBP/PRKCA/NCOA1
PIK3CA/PIK3CG/HIF1A/AKT3/PRKAA2/PIK3R1/CAB39/DDIT4/PRKAA1/VEGFA/RPS6KA3/MAPK1/[illegible]
PIK3CA/PIK3CG/FOXO1/PIK3R1/FOXO3/HSP90AA1
CHRNA2/CHRNA4/CHRNB2/CHRNB3
H2BC7/H2BC8/H2BC6/H2BC14/H2BC4/H2BU1/CLSPN/H4C8/POLK/H2BC21/NBN/H2BC5/TP53BP
EREG/AREG/TGFA/EGFR/PTPN12/CBL/EPGN/PTPRK/EPS15/STAM2/CDC42/STAM/HBEGF/SH3KB
BIRC3/PMAIP1/TNFRSF21/IGF2/BNIP3L/PIK3R1/TNFSF10/BIRC2/SCAF11/MCL1/MAP3K1/IGF1R/[illegible]
H2BC7/H3C14/H2BC8/H2BC6/H3C12/H2AC7/CDKN2B/H2BC14/PHC3/CDK6/H2BC4/MAP4K4/H2
CYP17A1/HSD3B1/HSD11B2/SERPINA6/POMC
HSD17B1/RDH16/RDH5/PDE6B/GRK7/APOC2/OPN1LW/LRAT/APOB/RBP3/AKR1C4/APOM/TTR/[illegible]
DRD3/CDK5/GNG2/DRD4/DRD2/CHRNA4/CHRNB2/PPP1R1B/KCNK3/DDC
GNB3/PAX6/FFAR4/PCSK1/SEC11C/CDX2
CRHR2/GNG13/GNG2/PTH2/SHH/RAMP2/FZD10/RAMP3/GNG8/CRHR1/GNB3/CRHBP/WNT8B/[illegible]
FOXO1/EP300/FOXO3/SIRT1/CREBBP/TXNIP
KRAS/NRAS/HTR2A/RASGRP1/ELK4/MAPK1/HTR2C/GNAQ/MAP2K1/ELK1/ITPR1
TGFB2/FCGR3A/CD44
PTGIS/CYP7B1/AKR1C2/NCOA2/NR1H4/CYP8B1
CHDH/ASMT/BHMT/PEMT/GRIN1
TLR7/TLR3/EIF2AK2/TRAF6/MAP3K1/MAPK14/MYD88/MAP3K7/ELK1/NFKB1/MAPK8
PROC/F5/FGB/F11/F9/KNG1/F12/SERPINC1/KLKB1/F2
GLI2/GLI3/GLI1/SMO/GSK3B/PRKAR1A/PTCH1/SUFU/PRKAR2A
KIT/KITLG/CBL/SOS1/SOCS6/YES1/SH2B3
PLA2G4F/PLA2G6/PLA2G10/PLA2G2D/PLA2G12B/PLA2G1B
FPR1/NCF2/CAMK1G/PPP3CA/NFATC3/MAPK1/MAP3K1/NFATC2/MAPK14/MAP2K1/ELK1/GNA[illegible]
FN1/ITGB3/IQGAP1/VCL/KRAS/NRAS/RAP1B/IL17RD/FGG/MAPK1/RAP1A/MAP2K1/TLN1
VCAN/DCN/HSPG2/XYLT1/GPC6/AGRN/BGN/GPC4
GAS1/EVC/CSNK1A1/BOC/SMO/EVC2/CDON/PTCH1
MAP2K2/CRHR1/PLCB4/PRKCB/PLA2G6/PRKG2/GNAO1/PLA2G10/PLA2G2D/GRIA1/GRM1/PLA2
H2BC7/H2BC8/H2BC6/H2BC14/H2BC4/H2BU1/CLSPN/WEE1/H4C8/YWHAZ/H2BC21/NBN/YWH[illegible]
FN1/ITGB3/RAP1B/FGG/RAP1A/TLN1
NOTCH2/MAML2/ACTA2/EP300/SMAD3/RBPJ/MAML1/CREBBP
INHBA/FSTL3/INHBB/SMAD3/MAPK1/ACVR1C/ACVR2A/SMAD2
MYLK/ANGPTL2/ROCK1/ACTR2/FLNC/PAK2/FSCN1/LIMK1/ACTR3/ROCK2/FLNA/CDC42/WASL/R[illegible]
BIRC3/TNFRSF10A/TNFSF10/BIRC2/PELI1/SDCBP/HSP90AA1/FAS/PDCD6IP/XIAP/TNFRSF10B/CA[illegible]
CLSPN/YWHAZ/NBN/BRCA2/CDK2/TOPBP1/ATR/CDC6/CHEK1/MDM2/CCNA2/FBXW11/MCM2/[illegible]
APOB/INS/SOD3/APOA1/SAA2/HBB/MMAB/SAA1/CBS/F7/MAT1A/HBA1/F2/ALB
ADAM17/FZD1/CTNNB1/GSK3B/APC/PSEN1/RBPJ

EREG/AREG/TGFA/PIK3CA/EGFR/FOXO1/NCK1/KRAS/ABL2/AKT3/CBL/PAK2/NRAS/PIK3R1/SOS1/
MYL2/VCL/ARHGAP5/ROCK1/LIMK1/DIAPH1/OPHN1/PIP5K1A/TLN1/ARHGAP1/ARHGAP6/RHOA
PIK3CA/KRAS/NRAS/PIK3R1/SOS1/LCP2/KLRD1/SHC1/PIK3CB
BIRC3/CTNNB1/BIRC2/TNFRSF12A/GSK3B/MAPK1/MAPK14/MAP3K7/CASP8/NFKB1/AKT2/MAPI
FGF23/FGF2/FGF16/FGF7/FGF10/FGF5
CTSV/CTSK/AP1S3/SLC11A1/ARSB/IGF2R/NPC1/CTSO/M6PR/LAMP2/LIPA/CTSC/MANBA/CLTC/G
TDGF1/CFC1/CER1/LEFTY1
ARL2/SLC25A6/SLC29A2/SLC25A4/SLC29A4
TGFB2/IL2RA/TGFBR2/TGFB3/TGFBR1/SMAD3
HTR7/PLIN3/RHOBTB3/CCNE1/CUL3/RAB9A/VHL/LRRC41
FOXO1/AKT3/FOXO3/CREB1/NR4A1/AKT2
ACHE/CGA/PCSK2/GATA4/INS/CTSG/VAMP2/ANPEP/GNB3/FSHB/KIF5C/CPE/PAX6/ISL1/AGT/UC
UGT1A6/UGT1A10/UGT2B28/HSD17B6/CYP1B1/HSD11B1/CYP7B1/UGT1A3/UGT1A8/UGT1A1/S
FGFBP1/FGF2/FGF7/FGF10
NCK1/NCKAP1/NTRK1/WASF2/ABI2/WASL/WASF1
IQGAP1/ARHGAP5/NCF2/NCKAP1/ACTR2/PAK2/LIMK1/ACTR3/CTNNB1/ATF2/CDH1/CYBB/WASI
STAT1/JAK1/JAK2/IFNGR1/PTPN11/PTPN1/SOCS3/PIAS1
CDK6/SMURF2/CDK2/ESR1/SMURF1/CDK1
ADORA2A/CRHR2/GNG13/ADCY4/GPR25/GNG2/PTH2/TSHB/RAMP2/AVP/CGA/RLN3/RAMP3/GI
CAV1/PIK3CA/HIF1A/IRS1/CAVIN1/ITGB4
COL1A2/ITGB1/ITGA2/COL1A1/ITGA1/GP5/FCER1G
ASAP1/PKD2/CNGB1/EXOC5/ARF4/RHO/EXOC1/EXOC8/EXOC7/EXOC2/RAB11A
WNT3A/FGF8/RIPPLY2
RIMS1/SLC1A7/VAMP2/RAB3A/STXBP1/SLC1A6/TSPOAP1/GLS2/PPFIA3/SNAP25/CPLX1/SLC1A2
MET/HGF/GAB1/ARF6
CHRNA2/CHRNA4/CHRNB2/CHRNB3
AKT3/YWHAZ/YWHAG/YWHAQ/AKT2/YWHAB/PPP3R1
PAPPA/SOS1/ATF2/SHC1/RPS6KA3/MAPK1/MAP3K1/MAP2K1/ELK1
DLG4/LGI2/LGI3/ADAM11/CACNG8/LGI4/LGI1/CACNG2
AR/ROR1/RARB/NR3C1/RORA/VDR/NR2F1/NR2F2/NR4A2/PPARD/ESR1/RARG/NR2C2/NR4A1/N
PTGS2/MITF/MEF2A/HBP1/CREB1/ATF2/ATF1/ELK4/RAB5A/ESR1/MEF2C/MAPK14/KRT19/EIF4E
NUAK1/TAF1L/PRKAA2/HIPK1/NBN/TAF2/PRKAA1/CDK2/TOPBP1/RBBP8/ATR/PLK3/MAPK14/DY
MMP1/CXCL8/GUCY1A2/GUCY1A1/MMP3/ACE2/GCLM/NFE2L2/HMOX1
GNG8/GNB3/GNG4/VIPR1/SCTR/GNG7/VIPR2/GIPR/GHRHR/GHRH
CRHR2/LINC02210-CRHR1/ADGRG2/CRHR1/ADGRL1/VIPR1/SCTR/VIPR2/GIPR/GHRHR
SLC2A1/HIF1A/LDHA/PDK1/NFE2L2/PRKAA1/VEGFA
VRK2/LEMD3/LMNB1/CDK1/PPP2R2A/ANKLE2/CCNB1/KPNB1/PPP2CA
IGFBP3/TNFRSF10D/TNFRSF10A/TP53BP2/FAS/TNFRSF10B/TP73
H2BC7/H2BC8/H2BC6/H2AC7/H2BC14/H2BC4/ATRX/H2BU1/H4C8/H2BC21/H2BC5/H2AC6/H4C
ADCY1/CYP1A1/CYP2C19/MAPK12/MAPK11/ADORA2A/MAPK10/FAAH/PRKAR1B/CYP2C9/CYP3
SLC2A1/SLC2A2/LDHA/PGK1/PGAM1/PFKP
CYP17A1/CGA/HSD17B3/HSD3B1/POMC
KRAS/NTRK1/RALB/NRAS/SOS1/RALA/SHC1/MAPK14
TNFRSF10A/TNFSF10/FAS/TNFRSF10B/CASP8/CFLAR
LPAR4/P2RY1/P2RY2/P2RY6/P2RY12/P2RY4/P2RY13
AP1S3/PICALM/IGF2R/SH3D19/PIK3C2A/CLTC/TBC1D8B/ACBD3/SNX9/PUM1/CPD/VAMP7/AP3S
FGF23/FGF2/FGF10/FGF5/SPRED1
PLAT/F2R/COL4A1/TFPI/COL4A5/COL4A4/COL4A2
CDH2/MYOG/TCF4/MEF2A/TCF12/SPAG9/CTNNB1/BOC/CDON/CTNNA1/BNIP2/CDC42/MEF2C/I
TOP2A/CDK2/CCNE2/CDK1/CCNE1/CDC6/RBL2/CCNA2/LIN9/TFDP1/LIN54/RBBP4/TFDP2/DYRK1

DDX58/IFIH1/CASP8/CHUK/CASP10/FADD/IKBKG/IKBKB/TRIM4/RIPK1/RNF135/TRIM25/MAVS
LEPR/PRKAA2/PRKAA1/CPT1A/PRKAB2
EP300/TRAF6/CREBBP/NFKB1/CHUK/RELA/TNFRSF1A/TNF/TNFRSF1B/NFKBIA/FADD/IKBKG/IKBK
PRDM1/LATS2/YAP1/TEAD1/STK3/FOXO1/STK38L/FYB1/LATS1/FOXO3/SAV1/MOB1A/TEAD3/ND
NDUFA4/SMARCD3/TACO1/FABP1/COX7C/HM13/COX19/COX7B/SURF1/COX6C/COX6B1/COX14
FN1/FGF7/LOXL4/ANXA5/NFE2L2/SIRT1/IGFBP7
PTGER1/S1PR4/CNR2/GPR50
GSTA4/SRM/MGST2/GGT7/GSTM4/GSTO2/GPX4/ODC1/GSTM2/GSTZ1/ANPEP/GSTA1/GGT6/GP
TUBB8/GRIN2C/TUBA8/TUBAL3/TUBA3C/TUBA3E/LIN7B/GRIN1/CAMK2B
ANXA1/MYLK/ITGB5/CALD1/GUCY1A2/VCL/TPM4/ITGA1/ACTA2/PAK2/GUCY1A1/PDE5A/DYSF/M
C1QL4/MUC12/MUC6/PARM1/LGALS9C/C1QTNF9/CLEC3B/C1QTNF4/SFTPD/CSPG5/MUC7/SFTP
TGFBR2/PMEPA1/SMURF2/TGFBR1/PPP1CB/SMAD3/SMAD7/PPP1R15A/SMURF1/XPO1/SMAD2
UROS/HMBS/ALAS2/HBB/HBA2/HBA1
FGF23/ANOS1/FGF2/FGF5
CGA/MAPK10/MAP2K2/CACNA1C/PLCB4/PRKCB/FSHB/MAP2K6/PLA2G6/CACNA1S/GNRH2/PLA
GJA1/DAB2/CLTC/CLTCL1/MYO6/AP2M1
PLAT/CALU/ITGA1/JAG1/SOX6/VEGFA/LPAR1/MGP

2/ITGA11/LAMA3/ITGAV/COL5A1/LAMC2/COL1A1/LAMA2/TGFBI/COL7A1/COL4A1/NID1/TNC/F
A2/ITGA11/ITGAV/COL5A1/COL1A1/COL7A1/COL8A2/COL4A1/LUM/ICAM5/ITGB8/TNC/ITGB3/IT
OL1A2/MMP20/LAMA3/COL5A1/LAMC2/COL1A1/PXDN/COL22A1/CTSV/ADAMTS2/COL7A1/COL
MMP20/LAMA3/COL5A1/LAMC2/COL1A1/PXDN/CTSV/COL7A1/COL8A2/COL4A1/COL17A1/COL2

11/LAMA3/ITGAV/COL5A1/LAMC2/COL1A1/LAMA2/COL4A1/ITGB8/TNC/ITGB3/ITGB5/ITGA5/CC

1/LAMA3/ITGAV/COL5A1/COL1A1/LAMA2/ASPN/MATN3/SPARC/HAPLN1/COL4A1/LUM/TNC/ITG
/LAMC2/FGF2/COL1A1/LAMA2/DDR2/COL4A1/TNC/ITGB3/ITGB5/HSPG2/LAMA4/COL5A3/COL4
TGB3/EFEMP1/ITGB5/ITGA5/FBN2/MFAP2/LOXL3/FBLN2/TGFB3/EFEMP2/FBLN1/EMILIN1/GDF5/
/PDGFRB/LAMA4/COL4A5/LAMC1/COL4A4/LAMB1/PLAUR/TGFBR2/THY1/CCN1/PDGFB/FGG/SP
EDN2/NR3C1/SLC26A2/BHLHE40/SERTAD2/PRRG4/ALOX5AP/LRRC8A/AKAP13/ARL5B/ENC1/FG
AMA2/COL24A1/LAMA4/HGF/COL5A3/LAMC1/ITGA3/LAMB1/LAMB3/DOCK7/RAP1B/TNS4/GAB
EDIL3/FGF2/COL1A1/COL7A1/COL8A2/COL4A1/ITGB3/COL17A1/PIK3CA/COL6A1/COL4A5/COL4A

P20/MMP8/COL5A1/COL1A1/MMP2/COL7A1/COL8A2/MMP11/ADAM9/COL4A1/MMP14/CTSK/
/COL6A1/PDGFRA/COL5A3/COL4A5/COL4A4/COL4A2/STAT1/NCK1/COL6A2/KRAS/PTPN12/COL6
COL1A1/COL7A1/COL8A2/COL4A1/COL17A1/COL6A1/HGF/COL4A5/COL4A4/COL14A1/COL6A2/
A1/COL22A1/ADAMTS2/COL7A1/COL8A2/COL4A1/COL17A1/P4HA1/COL24A1/ADAMTS3/COL6A
KN2B/STAT1/WWTR1/TGFBR2/PMEPA1/TGFB3/SMURF2/CBL/TGFBR1/CCNK/EP300/RNF111/PP
/ITGA6/LAMC1/BIRC3/ITGA3/CDKN2B/COL4A4/COL4A2/LAMB1/CDK6/LAMB3/AKT3/PIAS3/PIK3
2/MET/ITGA11/LAMA3/ITGAV/COL5A1/LAMC2/PDGFC/COL1A1/LAMA2/COL4A1/ITGB8/CAV1/TN
3/SOS1/ACTN1/ROCK2/DOCK1/CAPN2/YES1/MAPK1/WASL/RAP1A/GIT2/MAP2K1/TLN1/PTPN21/
2/SPARC/KRT6A/CXCL8/TNC/F13A1/TIMP2/PDGFRB/DCN/SLURP1/NOX4/TLR8/HGF/TLR4/IL1A/S
A1/COL1A2/MMP20/LAMA3/MMP8/COL5A1/LAMC2/COL1A1/MMP2/CTSV/COL7A1/COL8A2/M
0/ADAM17/TCF4/GLI2/NR3C1/DKK3/CCN2/EGFR/FZD1/GATA3/EGR2/KLK7/LEF1/KITLG/PHLDA1/
P12/YAP1/CDKN2B/KLF6/TGFBR2/KLF10/ROCK1/MEF2A/SMURF2/PAK2/TGFBR1/DAB2/PIK3R1/E
3/ITGA6/LAMC1/BIRC3/ITGA3/CDKN2B/COL4A4/COL4A2/LAMB1/CDK6/LAMB3/AKT3/POLK/PIK3
L1/ITGB3/BMPR2/ITGB5/CDKN2B/STAT1/WWTR1/TGFBR2/PMEPA1/BMPR1B/TGFB3/SMURF2/
V/LAMC2/PDGFC/COL1A1/LAMA2/COL4A1/ITGB8/CAV1/TNC/ITGB3/CAV2/PDGFRB/MYLK/ITGB

4/IQGAP1/ITGA6/ITGA3/NEDD4/FLNB/STAT1/ITGA4/ITGA1/EGFR/ACTN4/FLNC/HAVCR2/NPC1/E
A3/LAMC2/NID2/EDIL3/IGFBP5/FNDC1/LAMA2/TNFAIP6/PXDN/TGFBI/MATN3/LTBP2/SPARC/LTB
/UNC5B/UNC5A/ABLIM3/SEMA3E/SLIT2/UNC5C/EPHB6/NRP1/EPHA2/EPHA4/ROBO1/EFNA5/RC
RPL23/FAU/RPL27/RPL7/RPS10/RPL6/RPL37/RPS15A/RPL31/RPS4X/RPL17/RPS13/RPL12/RPL36A
/RPS3/RPL23/FAU/RPL27/RPL7/RPS10/RPL6/RPL37/RPS15A/RPL31/RPS4X/RPS14/RPL17/RPS13/

RPL23/FAU/RPL27/RPL7/RPS10/RPL6/RPL37/RPS15A/RPL31/RPS4X/RPS14/RPL17/RPS13/RPL12/
RPS3/RPL23/FAU/RPL27/RPL7/RPS10/RPL6/RPL37/RPS15A/TRIB3/RPL31/RPS4X/RPS14/RPL17/R
/SEC61B/RPS3/RPL23/FAU/RPL27/RPL7/RPS10/RPL6/RPL37/RPS15A/RPL31/RPS4X/RPS14/RPL17
RPS8/RPL11/RPS3/RPL23/FAU/RPL27/RPL7/RPS10/RPL6/RPL37/RPS15A/RPL31/RPS4X/RPS14/RP
STK/RPS8/RPL11/RPS3/RPL23/FAU/RPL27/RPL7/RPS10/RPL6/RPL37/RPS15A/RPL31/RPS4X/RPS1
RPL11/RPS3/RPL23/FAU/RPL27/RPL7/RPS10/RPL6/RPL37/RPS15A/RPL31/RPS4X/RPS14/RPL17/R
3A/COL17A1/ULBP3/IGKV5-2/KIR2DL3/MICB/IGLV3-25/VCAM1/CD300E/KIR3DL2/KIR2DL1/ICAM
TGB3/PDGFRB/FGF11/MYLK/ITGB5/FGF7/RDX/ITGA5/PIK3CA/PIK3CG/PDGFRA/IQGAP1/ITGA6/IT
T8/TCIRG1/RPL10A/RPS8/RPL11/RPS3/RPL23/FAU/RPL27/RPL7/RPS10/RPL6/RPL37/RPS15A/TRI
3/RPL10A/RPS8/RPL11/RPS3/RPL23/FAU/RPL27/RPL7/RPS10/RPL6/RPL37/SLC25A6/RPS15A/RPL
/RPL4/RPL36AL/NOP2/RPL41/TSR3/RPS25/RPL10/RPL5/NHP2/DDX49/SNU13/FBL/RPS4Y1/RPS1

PS23/AHCY/PAOX/PYCR2/OCA2/RPL10A/CHDH/SEM1/ETHE1/ASL/GCDH/TXN2/DCT/PSTK/RPS8/
0/RPL4/MRPL11/MRPS15/TUFM/MRPL21/RPL36AL/MRPS12/EIF3B/RPL41/RPS25/RPL10/RPL5/S
OL4A5/LAMC1/COL4A4/COL4A2/LAMB1/LAMB3/COL6A2/COL15A1/AGRN/COL6A6/NTN4/LAME
/CLDN1/ZEB1/ZEB2/CLDN11/ITGA5/PIK3CA/FZD6/HIF1A/WNT7B/LATS2/COL4A5/TWIST1/COL4
AMA2/COL24A1/PIK3CA/LAMA4/HGF/COL5A3/LAMC1/ITGA3/LAMB1/LAMB3/KRAS/DOCK7/CBL/
3/RPL10A/SEM1/RPS8/ELOB/RPL11/RPS3/RPL23/PSMB6/FAU/RPL27/RPL7/RPS10/RPL6/RPL37/R
ITGA5/CDH6/PDGFRA/LATS2/ITGA6/ITGA3/EPHA2/TEAD1/VGLL4/ITGA4/CCN2/ITGA1/EGFR/CD4

2/EPHA2/ARHGAP23/ARHGAP5/TRIO/FERMT2/ARAP2/NCF2/PLEKHG2/DOCK4/NCKAP1/DOCK9/
GFBR2/BMPR1B/ROCK1/ACVR1/TGFB3/SMURF2/TGFBR1/SMAD1/COMP/EP300/INHBB/ZFYVE16

NCKAP1/ACTR2/KRAS/CBL/EIF2AK2/ACTA2/PAG1/NRAS/PIK3R1/YWHAZ/PDGFB/RAP1B/YWHAG
T3/SPON1/ADAMTS5/THSD7B/MUC4/DCN/CHSY1/ADAMTS3/THSD4/HSPG2/ADAMTS7/THSD7A
P23/ARHGAP5/TRIO/ARAP2/ROCK1/ARHGAP21/DIAPH3/SOWAHC/ARHGEF12/ARHGAP31/VANG
/NCKAP1/ARHGAP21/JAG1/DIAPH3/ABL2/PAK2/VANGL1/ERBIN/ARHGAP32/NHS/PIK3R1/SLITRK

SM/MMP3/CREB1/SOS1/JAK1/SHC1/VEGFA/CDK2/MAPK1/JAK2/STAT3/MTOR/RICTOR/LDLR/EG
EP300/CTNND1/CTNNB1/ACTN1/SMAD3/CDH1/WASF2/YES1/MAPK1/CTNNA1/IGF1R/CDC42/W
PINH1/GLI3/STAT1/ADAMTS4/SLC38A2/PTHLH/IGF2/HMGCS1/CAB39/NKX3-2/SOX6/SPP1
PINH1/GLI3/STAT1/ADAMTS4/SLC38A2/PTHLH/IGF2/HMGCS1/CAB39/NKX3-2/SOX6/SPP1
N1/ETS1/EP300/EDN1/CTNNB1/ELF1/PTEN/CREB1/SP1/ATF2/NFATC3/ATF3/BCL2L11/ESR1/DUS
AP32/CDC42EP3/SLC4A7/DLC1/SRGAP2/GOPC/SNAP23/SYDE1/VAMP3/RHOQ/CDC42/STEAP3/C
A2/EGFR/MAP4K4/PMAIP1/BNIP3L/VDR/DUSP5/TNFRSF10D/BCL6/TNFRSF10A/CCNK/EP300/SE
22A1/COL7A1/COL8A2/COL4A1/COL17A1/COL24A1/COL6A1/COL5A3/COL4A5/COL4A4/COL4A2/
22A1/COL7A1/COL8A2/COL4A1/COL17A1/COL24A1/COL6A1/COL5A3/COL4A5/COL4A4/COL4A2/
1/CLDN11/PIK3CA/CDH6/NECTIN3/SIRPB1/IQGAP1/ITGA6/SFTPA1/FERMT2/CLDN16/PRKCI/ACT
B1/CDH1/EPS15/SHC1/APC/MAPK1/RAB5A/CTNNA1/MAP3K1/MTOR/CDC42/EGR1/WASL/PTPN
FR/PRKCI/ROCK1/FOXO1/NCK1/MEF2A/KRAS/PTPN12/MAP3K2/CBL/PIAS3/RALB/PIK3R1/PLSCR
217/H2AC7/NRIP1/H2BC14/GNG12/H2BC4/EGFR/RUNX1/STAG1/GNGT1/GATA3/H2BU1/KRAS/A

1/ITPR3/NCKAP1/IGHV2-70/IGLV7-43/ACTR2/MYH9/IGHV3-33/IGKV1-39/PIK3R1/IGKV1-33/MY
/DIAPH3/PAK2/VANGL1/ERBIN/ARHGAP32/NHS/PIK3R1/SLITRK5/VRK2/DOCK1/LEMD3/CYBB/SY

3/COL4A4/VCL/FAT1/PLAUR/ACTN4/CD80/CD2AP/NCK1/JAG1/MYH9/PTPRO/AGRN/UTRN/PARV

AF1/GBP5/EIF2AK2/OAS2/DDX58/TRIM17/IFIT1/IFNA1/KPNA4/HLA-G/NUP160/JAK1/TRIM34/N
IST1/STAT1/LDHA/AK4/CD44/MTDH/ACTA2/SNAI1/GOLGA6L17P/EP300/TFAP2A/PTEN/CDH1/A
/OLR1/IGKV5-2/ITGA5/PIK3CA/IGLV3-25/PF4V1/ITGA6/ITGA3/PSG9/ITGA4/CD44/IGKV2-28/JCH
6/ATP5MF/UQCRC1/ATP5PF/COX7C/NDUFV3/CYC1/NDUFS3/UQCRB/NDUFC1/COX19/UQCR10/

4A7/ACTN1/BASP1/SRGAP2/SNAP23/SYDE1/VAMP3/TOR1AIP1/DIAPH2/LMNB1/STEAP3/ADD3/
HE40/PRKAA2/EFNB2/RB1CC1/SMAD1/LAMP2/BMP3/TNFSF11/SERPINF1/CLOCK/TRAF6/JAK2/M

RPS13/RPS17/RPS27A/RPS20/RPS3A/RPS7/RPS11/EIF3G/RPS19/RPS18/RPS2/RPS29/RPSA/EIF4E
XL2/SERPINE1/MMP20/TGM2/PAPPA/TIMP3/MMP8/SERPINB4/CST8/MMP2/CTSV/ADAMTS2/M
HX1/GATA3/HOXD11/GRIP1/CELSR1/CRIM1/GLI1/ROBO2/GDNF/SMAD1/FOXC1/CTNNB1/SMO/
/PIAS3/NCOA2/EP300/SP3/FOXO3/CREB1/SP1/SMAD3/ATF2/SMAD7/SKI/ATF3/CDK2/ESR1/CBFB
-33/IGKV1-39/PIK3R1/IGKV1-33/IGKV1D-39/FCER1G/IGHV2-5/IGLV6-57/SOS1/FCER1A/IGHV3-30

/IQGAP1/MSN/VCL/FGF10/GNG12/FGF5/ENAH/ITGA1/EGFR/SSH1/MYL1/ROCK1/NCKAP1/DIAPH
NRAS/PIK3R1/PDGFB/SOS1/JAK1/GAB1/GNAI3/SHC1/VEGFA/JAK2/IGF1R/IFNGR1/PTPN11/TEK/I
X7C/NDUFV3/NDUFS3/UQCRB/NDUFC1/UQCR10/NDUFA9/COX7A2/NDUFA1/NDUFB10/COX7B/

1/YWHAZ/SMAD1/YWHAG/ACTR3/CREB1/SOS1/GAB1/RALA/ATF2/CAPN2/ARF4/ATF1/WASF2/RI

F1/KRAS/AKT3/BCL6/NRAS/PIK3R1/SOD2/PDK1/PIK3C2A/FOXO3/CTNNB1/PTEN/PIK3R4/SOS1/V

1/SMAD3/ATF2/SHC1/MAPK1/JAK2/STAT3/EZR/CDC42/DIAPH1/MAPK14/MYD88/MAP2K1/CASI
32/AKAP13/PIK3R1/PKN2/CAVIN1/SLK/DLC1/ROCK2/ARHGAP18/FMNL2/VAMP3/ARHGEF17/LM

PGN/NRAS/PIK3R1/S1PR3/MMP3/FOXO3/CREB1/MMP7/GNAI3/SHC1/HSP90AA1/MAPK1/GNB1/
1/CDH6/NECTIN3/ITGA6/FERMT2/CLDN16/PRKCI/LAMB3/CDH13/DST/FLNC/PARD6B/CLDN8/CT
PP3CA/IGHV3-33/IGKV1-39/IGKV1-33/IGKV1D-39/FCER1G/IGHV2-5/IGLV6-57/SOS1/LCP2/FCER1
A/RPL31/RPS4X/RPS14/RPL17/RPS13/RPL12/RPL36A/RPS17/RPL24/RPL14/RPLP0/RPS27A/PAK6
/ARHGAP5/TRIO/CPNE8/ARAP2/PLEKHG2/DOCK9/ARHGAP21/DIAPH3/DOCK7/GNA13/ARHGEF1
3/PAK2/NRAS/IGKV1-39/IGKV1-33/IGKV1D-39/FCER1G/IGHV2-5/IGLV6-57/SOS1/LCP2/FCER1A/I
ADAMTS7/THSD7A/ADAMTS4/ADAMTSL4/ADAMTS6/ADAMTS9/B3GLCT/ADAMTS16/ADAMTSL1

TNNB1/SOS1/ITCH/ZFYVE16/SMAD3/SMAD7/PPP1R15A/WWP1/SHC1/OCLN/SMURF1/XIAP/PPI

/RPS14/RPS13/RPS17/RPS27A/RPS20/RPS3A/RPS7/RPS11/RPS19/RPS18/RPS2/RPS29/RPSA/RPS

NB1/ATF2/CDH1/GSK3B/APC/SEPTIN2/YES1/MAPK1/ARHGEF6/CTNNA1/MAP3K1/MTOR/DLG1/

F1/SHC1/MAPK1/AGTR1/PTPN11/TEK/MAPK14/ANGPT4/ELK1/RPS6KB1/NFKB1/MAPK8
PIK3R1/CTNNB1/GAB1/PRKAA1/VEGFA/HSP90AA1/MAPK1/CTNNA1/CDC42/PTPN11/MAPK14/M

TGA4/ITGA1/ROCK1/AKT3/PAK2/ITGAM/RAP1B/TNS1/ITGA9/SOS1/ROCK2/ITGB4/DOCK1/MAPK
B/ASAP2/KLK6/S100A4/HILPDA/TIMP2/CST6/ADAMTS5/IL25/KRT16/IGFBP3/PRDM1/HIF1A/SLC
3R1/SLC4A7/FNBP1L/SNAP23/SYDE1/VAMP3/CDC42/STEAP3/CDC42BPA/WASL/OPHN1/FMNL3/
OX17/UQCRHL/COX7C/ATP6V0A1/NDUFV3/CYC1/NDUFS3/UQCRB/NDUFC1/UQCR10/NDUFA9/
K1/FOXO1/JAG1/KRAS/PIK3R1/TJP1/FOXO3/CTNNB1/CREB1/SOS1/MMP7/PRKD1/SP1/ATF2/BI

IDUFV3/CYC1/NDUFS3/UQCRB/NDUFC1/COX19/UQCR10/NDUFA9/NDUFA1/NDUFB10/COX7B/SU

2/CSNK1G3/BCL2L11/SIRT1/ZFAND5/CSNK1G1/XPO1/RBL2/CREBBP/YWHAQ/SGK1/YWHAB/MA
3R1/JAK1/E2F3/RALA/SMAD3/RB1/VEGFA/BRCA2/MAPK1/ARHGEF6/STAT3/PIK3CB/RALBP1/CD

S1/E2F3/CBLB/SMAD3/RB1/SHC1/MAPK1/PIK3CB/PIK3R5/PTPN11/SOS2/MAP2K1/CDKN2A/MD

3/ACTR2/MYH9/IGHV3-33/IGKV1-39/IGKV1-33/MYO5A/IGKV1D-39/IGHV2-5/WIPF1/IGLV6-57/AC

S/PIK3R1/PDGFB/EP300/CTNNB1/PTEN/CREB1/SOS1/E2F3/SRD5A2/RB1/GSK3B/CDK2/HSP90AA1
/APOL1/IGKV5-2/IGLV3-25/COL4A2/COLEC12/IGKV2-28/JCHAIN/IGHV2-70/IGLV7-43/IGHV3-33/H
/ERBIN/ARHGAP32/AKAP13/PIK3R1/MYO9A/NET1/PKN2/CAVIN1/SLK/DLC1/RHPN2/ROCK2/SNAP

1/GSK3B/SHC1/RPS6KA3/MAPK1/LNPEP/CDC42/RPS6KA2/SOS2/PTPN1/CDKN2A/IGFBP1/YWHAG

2/SLC4A7/ACTN1/MOSPD2/LEMD3/VAMP3/TOR1AIP1/DIAPH2/LMAN1/LMNB1/STEAP3/ADD3/D

FRP4/FGF10/WNT10A/FGF5/CDK6/EGFR/FZD1/LEF1/NOTCH3/FZD7/KRAS/AKT3/CSNK2A3/CSNK1

ETS1/PDGFB/RAP1B/EP300/SOS1/GAB1/VEGFA/MAPK1/PIK3CB/CDC42/PIK3R5/PTPN11/ARNT/S
GF/FGF10/FGF5/CDK6/EGFR/KRAS/AKT3/NRAS/PIK3R1/PDGFB/PTEN/E2F3/CDH1/RB1/MAPK1/P
CAM1/TWIST1/SOCS5/OPRM1/RORA/ICAM1/STAT1/GATA3/FOXO1/BCL6/PIK3R1/FSCN1/ITGAM
1/RARB/MIR125B1/TEAD1/GATA3/BHLHE40/ACVR1/LEF1/MACF1/ARID5B/SLC2A12/SNAI1/UBR5

D3/VEGFA/CXCR1/STAT3/PTPN11/MAPK14/CCND1/RRM2/NFKB1/CTTN/BCL2L1/MAPK8

AKT3/CBL/NRAS/PIK3R1/NF1/EP300/PIK3C2A/FOXO3/PTEN/GAB1/RB1/BRCA2/CDK2/MAPK1/PIK

XO1/GNA13/PAG1/RALB/PIK3R1/RAP1B/LIMK1/ITGA9/PTEN/ITCH/GNAI3/STAT2/YES1/GNB1/JA

COA2/CCN1/HBP1/YWHAZ/EP300/TNIK/SALL4/YWHAG/CTNNB1/CDH1/INCENP/APC/PITX2
R3/IGHV2-70/IGLV7-43/IGHV3-33/IGKV1-39/PIK3R1/IGKV1-33/IGKV1D-39/IGHV2-5/IGLV6-57/IGH

1/NDUFA8/NDUFAF6/ATP5MF/UQCRC1/ATP5PF/ADHFE1/COX7C/NDUFV3/CYC1/MDH2/NDUFS3/
DGFRA/HGF/FGF10/FGF5/EGFR/IRS1/CD80/CD86/FRS2/KITLG/AKT3/STRN/EPGN/PIK3R1/PDGFB
7/C1S/H2BC14/H2BC4/ACTN4/CD80/CD86/H2BU1/RO60/H2AC11/H4C8/C7/C1R/FCGR3B/H2BC2

/TRIM34/SMAD7/SP100/GBP2/TRIM5/GBP3/JAK2/IFNGR1/TRIM29/HLA-DQA1/PTPN11/HLA-DPA
MC3/HLTF/SMC1A/BARD1/CDK1/CCNE1/TTK/MSH6/CHEK1/MDM2/ABL1/CCND1/CCNA2/RRM2/S

ROCK2/SP1/GSK3B/SHC1/PRKAA1/MAPK1/JAK2/STAT3/MTOR/ESR1/CDC42/PTPN11/MAPK14/PT
SEL/PRELP/XYLT1/B4GALT5/SLC26A2/CD44/B4GALT1/GPC6/ST3GAL1/ARSB/AGRN/HS2ST1/BGN/
LR3/TLR1/TLR2/IKBIP/BPI/ITGAM/TAB2/DUSP7/CREB1/PIK3R4/FGG/APP/UBE2D1/ATF2/BIRC2/IT

F10/FGF5/EGFR/IRS1/CD80/CD86/FOXO1/FRS2/KITLG/AKT3/STRN/EPGN/PIK3R1/PDGFB/FOXO3/
FH/ATP11C/C3AR1/CSMD1/TGFB3/C7/PARD6B/C1R/CAP1/ITGAM/PLSCR1/C4B/C1QC/CD59/ATP1
KT3/TGFBR1/TLR2/DDX58/NRAS/PIK3R1/YWHAZ/TAB2/EP300/IFIH1/IFNA1/CREB1/SOS1/JAK1/SM

MUC4/ADAMTS3/THSD4/ADAMTS7/THSD7A/ADAMTS4/MUC21/ADAMTSL4/NOTCH3/ADAMTS6
PON1/ADAMTS5/THSD7B/MUC4/ADAMTS3/THSD4/ADAMTS7/THSD7A/B4GALT5/ADAMTS4/MU(
AT1/NAMPT/AHR/WWTR1/IRS1/BMP1/FZD1/GATA3/EGR2/FOXO1/MEF2A/IL6ST/NCOA2/NR2F1

AM/TAB2/JAK1/ITGB2/MAPK1/TRAF6/JAK2/IFNGR1/HLA-DQA1/FCGR2C/C3/HLA-DPA1/MAPK14
KRAS/AKT3/PAK2/NRAS/FLT1/PIK3R1/CTNND1/CTNNB1/ROCK2/DOCK1/CYBB/VEGFA/WASF2/HS
3-33/IGKV1-39/IGKV1-33/IGKV1D-39/IGHV2-5/IGLV6-57/IGHV3-30/IGHV1-2/IGHV4-39/YES1/IGK

TIN3/VCAM1/CD274/ITGA6/ICAM1/ITGA4/CLDN16/PDCD1LG2/CD80/CD86/ALCAM/CLDN8/ITGA
KPNA3/FLNA/USP18/KPNA1/UBE2L6/EIF4G2/ARIH1/NDC1/OAS1/NUP107/EIF4G1/ABCE1/MX1/U

F10D/TNFRSF10A/NTRK1/PIK3R1/TNFSF10/BIRC2/CAPN2/PIK3CB/FAS/PIK3R5/PRKAR1A/XIAP/TN

PGK1/TGFB3/ENO1/FLT1/PDGFB/EP300/SDS/PBRM1/PTEN/PGM2/PKM/PFKP/VEGFA/STAT3/MT

RAET1L/KRAS/PPP3CA/PPP3R2/TNFRSF10D/TNFRSF10A/NRAS/FCGR3B/PIK3R1/KIR2DS4/TNFSF1
DM3A/RPS6KA3/NCOA4/UBA3/PAWR/UBE3A/RANBP9/NKX3-1/APPL1/XRCC5/CASP8/CDKN2A/T(
33/C1R/IGKV1-39/IGKV1-33/IGKV1D-39/C1QC/IGHV2-5/IGLV6-57/IGHV3-30/IGHV1-2/IGHV4-39/

/FZD6/WNT7B/FGF10/WNT10A/FGF5/EGFR/FZD1/BMPR1B/ACVR1/ACTR2/IL6ST/FZD7/AKT3/PD
CRHL/COX7C/NDUFV3/CYC1/NDUFS3/UQCRB/NDUFC1/SLC25A31/UQCR10/NDUFA9/COX7A2/NI

XOC5/CTNNB1/ITGA9/CLTC/RALA/CDH1/VAMP3/CTNNA1/AGTR1/ARF6/EDNRB/ITGA8/EXOC1
DUFS3/NDUFC1/NDUFA9/NDUFB10/ATP5F1E/NDUFB1/NDUFA2/ATP5MC2/NDUFV1/NDUFB2/AT

3/S100A7/CREB1/ITGB4/SP1/SMAD3/GTF2H2/NFYA/NDRG1/SFXN3/CSDE1/CFLAR/SMAD2/CCND

2/TNFRSF10A/YWHAZ/TNFSF10/TJP1/CHMP2B/YWHAG/PKP1/PLEC/CTNNB1/DAPK1/BIRC2/CDH

C5/TP53BP1/RNF168/H4C12/TOPBP1/RBBP8/ATR/H2BC13/WRN/BARD1/CDK1/H2BC11/CHEK1/I
LF4/SLC7A7/CREB3L2/RGS1/CD4/C3/GAL3ST4/SH2B3/IGSF6/CYTL1/ADAP2/IL13RA1/ABCC4/IL18
V7-43/KRAS/PPP3CA/IGHV3-33/PAK2/NRAS/IGKV1-39/PIK3R1/IGKV1-33/TAB2/IGKV1D-39/FCER1

NNB1/RALA/SMAD3/GSK3B/APC/MSH2/MAPK1/BCL2L11/DCC/MAP2K1/MSH6/APPL1/SMAD2/M
/KCNQ1/GNG8/GNB3/KCNG2/KCNAB3/KCNC1/HCN4/HCN1/GNG4/KCNF1/KCNG1/KCNV2/KCNK7
/2-70/IGLV7-43/IGHV3-33/IGKV1-39/IGKV1-33/IGKV1D-39/IGHV2-5/IGLV6-57/CREB1/IGHV3-30/I
1A/ARG2/NOS2/AOC1/CKMT1B/PRODH/GATM/GLS2/CKB/OTC/GAMT/CPS1/PRODH2
3B/APC/MSH2/MAPK1/PIK3CB/DCC/PIK3R5/MAP2K1/MSH6/APPL1/SMAD2/MSH3/CCND1/TCF7L
A/RASGRF2/FGF10/GNG12/FLNB/FGF5/EGFR/TGFBR2/MAP4K4/STK3/HSPA1A/NTF3/TAOK1/TGFI
WTR1/RUNX1/MAF/AKT3/SMAD1/NKX3-2/RB1/WWP1/GSK3B/YES1/MAPK1/SATB2/ESR1/HAND2
/GLI1/CSNK1A1/SMO/WNT11/LRP2/GSK3B/CSNK1G3/WNT5B/PTCH1/SUFU/CSNK1G1

P300/TFAP2A/SP3/NUP160/NR1H4/TP53BP1/NUP153/RNF168/H4C12/NR4A2/TOP1/SP100/STA(

1/NDUFA9/NDUFA1/NDUFB10/NDUFB1/NDUFA2/NDUFV1/NDUFB2/NDUFB8/NDUFS8/ECSIT/ND
N/KRT79/DSG2/KRT4/PPL/SPRR1B/KRT85/PCSK6/KRT80/KRT34/KRT6C/KRT75/KRT17/KRT83/CDS
K2/SP1/SMAD3/RB1/FLNA/GSK3B/NCOA3/STAT3/NCOA4/SIRT1/CDC42/UBE3A/NR2C2/CCNE1/N

COX6B1/ATP1A4/COX7B2/COX8A/COX5A/COX4I2/COX6A1/CACNA1C/CACNG8/UQCRQ/COX4I1/C
/H2BC4/GATA3/H2BU1/CLSPN/SMURF2/H2AC11/TGFBR1/DDX58/SMAD1/H2BC21/IFIH1/H2BC5/

TF2/PELI1/USP18/ATF1/RPS6KA3/MAPK1/TRAF6/TLR9/MAP3K1/TAB3/MEF2C/RPS6KA2/PPP2CE
/HBEGF/MAPK14/MAP3K5/CCNE1/ELK1/CDKN2A/CFLAR/CCND1/CCNA2/BMF/BCL2L1/MAPK8

5/ITGA3/DSG2/ITGA4/ITGA1/ACTN4/LEF1/DSC2/CTNNB1/ITGA9/ACTN1/ITGB4/CACNB4/ATP2A2/

1/GAB1/GSK3B/SHC1/CDK2/RPS6KA3/MTOR/CDC42/PIK3R5/RPS6KA2/SOS2/PTPN1/CDKN2A/IGF

NRAS/PIK3R1/PTPRK/SOS1/GAB1/EPS15/SHC1/STAM2/CDC42/STAM/PTPN11/HBEGF/SH3KBP1

A/YWHAZ/TNFSF10/TJP1/YWHAG/PKP1/PLEC/CTNNB1/DAPK1/BIRC2/CDH1/H1-4/GSDME/APC/N
K1/TAB3/PTPN11/MAPK14/MYD88/MAP2K1/MAP3K7/UBE2V1/MAP3K3/NFKB1/MAPK8
FR/PRKCI/ARAP2/HSPA1A/ACAP2/SMURF2/CBL/PARD6B/DAB2/NTRK1/FLT1/RAB11FIP1/CHMP2

KT3/TLR2/PIK3R1/CXCL10/TAB2/IFNA1/SPP1/CXCL11/MAPK1/TRAF6/TLR9/PIK3CB/PIK3R5/TLR6/

D/PPP1CB/CREB1/NOCT/MED1/ATF2/NFIL3/CLOCK/SIRT1/MEF2C/TBL1XR1/FBXL3/ARNTL/CPT1A

33/C1R/IGKV1-39/IGKV1-33/IGKV1D-39/C4B/C1QC/IGHV2-5/IGLV6-57/IGHV3-30/IGHV1-2/IGHV
DGFB/NF1/FOXO3/PTEN/MRAS/SOS1/JAK1/GAB1/GSK3B/SHC1/VEGFA/MAPK1/JAK2/STAT3/PIK3
S2/TLR2/DDX58/PIK3R1/TAB2/IFIH1/IFNA1/TNFAIP3/HSPA1B/JAK1/CBLB/STAT2/CDK2/TRAF6/TL
5/ITGA3/DSG2/ITGA4/ITGA1/ACTN4/LEF1/DSC2/CTNNB1/ITGA9/ACTN1/ITGB4/CACNB4/ATP2A2/

/BIRC2/USP18/ATF1/RPS6KA3/MAPK1/TRAF6/TANK/TAB3/PTPN11/MEF2C/RPS6KA2/PPP2CB/N
/PIK3R1/JAK1/E2F3/RALA/SMAD3/RB1/VEGFA/BRCA2/MAPK1/ARHGEF6/STAT3/PIK3CB/MTOR/F
CR3/JAK1/ITGB2/GSK3B/CXADR/MAPK1/STAT3/EIF4G2/BNIP2/ILK/SGCB/CD4/EIF4G1/CASP8/TLR

B/GLI2/FGF10/GLI3/WNT10A/STAT1/FGF5/PRKCI/FZD1/BMPR1B/NOTCH3/FZD7/JAG1/PRKDC/P

IT4/CTNNB1/PTEN/GAB1/BIRC2/RB1/NFE2L2/PRKAA1/VEGFA/PIK3CB/IGF1R/MTOR/RICTOR/AJU

1/MMP7/CDH1/RAB5A/CTNNA1/ZBTB33/IGF1R/CDC42/ARF6/PTPN1/RAB7A/RIN2/ABL1/CREBBP

LR2/PIK3R1/CXCL10/TAB2/IFNA1/SPP1/CXCL11/MAPK1/TRAF6/TLR9/PIK3CB/TAB3/PIK3R5/TLR6/
/SMAD3/RB1/GSK3B/STAG2/RAD21/CDK2/CDC27/BUB1B/ANAPC1/ATR/CCNE2/CDC14A/SMC3/S

5A/PIK3CB/PIK3R5/SYNJ1/SYNJ2/PIP4K2A/MTMR6/PLEKHA1/PIP5K1A/RAB14/PLEKHA8/MTM1/P
RBIN/ARHGAP32/PIK3R1/VRK2/DOCK5/DOCK1/LEMD3/KTN1/GARRE1/VAMP3/LMAN1/CDC42/C
/CSF3R/JAK1/MAPK6/CSF2RA/MAPK1/JAK2/STAT3/IFNGR1/MTOR/RICTOR/AGTR1/MAPK14/IL7R
LEF1/CDH13/PRKD3/PRKAA2/NTRK1/FLT1/CSF1R/LATS1/CTNNB1/GNAI3/SMAD3/CDH1/PRKAA1/

1/ATF2/RB1/ATF3/MAPK1/ESR1/DUSP1/CBFB/MAPK14/POU2F1/SOCS3/CCND1/PRKCA/CCNA2/E
I/IL17RD/H1-4/MGA/ARID4B/NSD1/BCORL1/MORF4L1/SMC1A/CMTR2/NFIB/KMT2C/CHEK1/ARI
RC2/CCL7/HSP90AA1/MAPK1/TRAF6/CARD6/TAB3/CCL11/XIAP/NOD1/MAPK14/MAP3K7/CASP8

NA2D3/RIMS1/NCALD/LRRTM1/LRRC7/LRRTM4/HRAS/KCNA1/KCNMB2/SLC1A7/KCNN1/HCN3/K

/STAT3/PIK3CB/MTOR/PIK3R5/SOS2/MAP2K1/RUNX1T1/CCND1/TCF7L2/RPS6KB1/NFKB1/AKT2/

D1/NCOA2/ETS1/PFKFB3/EP300/EDN1/CREB1/SP1/SMAD3/ITGB2/PKM/VEGFA/HMOX1/MCL1

DK6/FZD1/LEF1/FZD7/CSNK2A3/CSNK1A1/TFAP2A/CTNNB1/RYK/DKK2/WNT11/SERPINF1/ROR2/

A6/ITGA3/ITGA4/TPM4/ITGA1/TGFB3/PRKAA2/ITGA9/ITGB4/MYL3/CACNB4/ATP2A2/PRKAA1/C

1/VRK2/H2BC5/NUP160/NUP153/H2AC6/H4C12/H2AC14/LEMD3/RB1/ARPP19/MAPK1/SMC2/L

/NDUFV3/CYC1/NDUFS3/UQCRB/NDUFC1/UQCR10/NDUFA9/COX7A2/NDUFA1/NDUFB10/COX7

PSS2/MAPK14/MAP2K1/MAP3K7/NFKB1/AKT2/MAPK8/GAB2/FHL2/CALCR/FOS/PTK2/CHUK

MTN/PRSS23/CALU/TNC/FSTL1/MELTF/CDH2/APOL1/IGFBP3/LAMC1/ADAM10/LAMB1/MXRA8/

HLH/SLC2A12/DUSP5/DAB2/UBR5/ASCC3/PHF6/CDC73/FOXN3/CTNNB1/APP/TRERF1/SMAD3/E

1/VIM/STK24/LMNB1/CASP8/SATB1/SPTAN1/STK26/TJP2/DFFA/H1-0/BMX/KPNB1

SMAD3/RB1/GSK3B/STAG2/RAD21/CDK2/CDC27/ANAPC1/ATR/CCNE2/CDC14A/SMC3/SMC1A/

R1/SSTR2/MLNR/ADRA2C/GRIA1/GRM4/VIPR1/KISS1R/GH1/SCTR/S1PR4/GRM1/VIPR2/DRD2/C

SMC1A/WRN/BARD1/CDK1/XRCC5/CHEK1/MDM2/SMARCC2/PALB2/BRCC3/MCM2/ABRAXAS1/

5/ITGA4/CLDN16/ACTN4/NCF2/ROCK1/THY1/CLDN8/PIK3R1/ITGAM/RAP1B/CTNND1/CTNNB1/

R4/IL1A/MMP12/XYLT1/EPHA4/ICAM1/EGFR/TNFSF13B/PPP3CA/CCR2/GDNF/EFNB2/CXCL10/RT
A/BAG4/GSK3B/MAPK1/SIRT1/ATR/BAG2/NDC1/DNAJB1/NUP107/GML/HSPA5/NUP58/NUP205/

WNT10A/FZD1/LEF1/FZD7/PPP3CA/CSNK2A3/VANGL1/PPP3R2/CSNK1A1/GPC4/CTNNB1/RYK/DK

AP2A/ACE2/JAK1/SMAD3/STAT2/CXCL11/TRAF6/TANK/CASP8/MX1/IFNAR2/IFITM1/IFNAR1/TBK1
ZD1/MTDH/LEF1/FZD7/CSNK2A3/CSNK1A1/CTNNB1/RYK/DKK2/WNT11/SERPINF1/ROR2/GSK3B/
ATP2B2/KCNIP2/FXYD1/SCN3B/FGF14/KCNK15/NPPA/RANGRF/CACNG6/SLC8A3/KCNK7/FXYD2/K

HD1/TRIM32/NEK1/CILK1/PTCH1/CNGA1/TMEM67/SUFU/BBS9/BBS10/ARNTL/TRAF3IP1/ARL6/C
/OR2G3/OR4A16/OR4N5/OR51D1/OR51S1/OR52E2/OR5AN1/OR6C76/OR8D1/OR9Q2/OR10K1/C

NF152/UBE2D1/PEX13/UBE2Q2/H2BC13/HLTF/BCL10/UBE2W/UBE2H/UBE2E3/H2BC11/RTF1/UB

5/H2BC4/MAP4K4/H2BU1/H4C8/ETS1/HMGA1/H2BC21/NBN/TNIK/H2BC5/E2F3/SP1/H2AC6/H4C

NCOA2/YWHAZ/PKN2/H2BC21/YWHAG/PPP1CB/H2BC5/H2AC6/H4C12/H2AC14/PPP1R12A

/LAMB1/TUBB3/ITGA1/EGFR/EPHB2/ALCAM/SPTBN1/SPTBN2/ITGA9/CLTC/SDCBP/RPS6KA3/MA

B1/NDUFAF4/NDUFC2/NDUFA8/NDUFAF6/NDUFV3/NDUFS3/NDUFC1/NDUFA1/NDUFB10/NDUF

AX/UQCRC1/ATP5PF/UQCRHL/CREB3L4/COX7C/NDUFV3/CYC1/NDUFS3/UQCRB/NDUFC1/SLC25A

F168/H4C12/PPP4R2/CDK2/TOPBP1/RBBP8/ATR/H2BC13/WRN/BARD1/H2BC11/CHEK1/H4C14/B
1/PEX13/H2BC13/HLTF/BCL10/H2BC11/RTF1/UBE2E1/RNF40/UBE2V2/UBE2D3/UBC/RAD18/UBE

RD2/CLDN5/EMC10/SEPTIN5/DGCR6L/NPRL2/SLC2A4/SLC7A4/RORC/THAP7/AIFM3/TMEM191A/

BC5/H2AC6/H4C12/H2AC14/RB1/H1-4/CDK2/LMNB1/CCNE2/UBN1/H2BC13/CCNE1/H2BC11/H4

1/RNF168/H4C12/H2BC13/BARD1/H2BC11/XRCC5/H4C14/BRCC3/ABRAXAS1/RAD50/MRE11/ATI
/FZD7/LRRK2/EP300/PPP2R2C/CTNND1/CTNNB1/MMP7/PRKD1/WNT11/GSK3B/APC/PPARD/ZBT

G1/GATA3/H2BU1/GREB1/H4C8/NCOA2/EP300/H2BC21/GTF2A1/H2BC5/MED1/SP1/H2AC6/H4C

/SPTA1/COL6A2/KRAS/AGRN/GDNF/COL6A6/NRAS/SPTBN1/SPTBN2/CREB1/SOS1/CACNB4/MAP
3A/CACNA1B/GRIN2C/TUBA8/GABRB3/TUBAL3/PRKAR1B/GLRA2/GABRA1/SLC18A3/GNG4/TUBA

GFR/H2BU1/DYNC1LI2/H2AC11/H4C8/DYNC1I2/H2BC21/H2BC5/NUP160/CREB1/NUP153/H2AC6

/EP300/H2BC21/H2BC5/CREB1/KMT2A/H2AC6/H4C12/H2AC14/CDK2/STAT3/CBFB/H2BC13/H3C

HD1/KPNA1/EGR1/PTPN11/ADAR/IFIT5/OAS1/PTPN1/ABCE1/MX1/MX2/IFNAR2/IFITM1/IFNAR1/

K2/ITSN1/EPHB2/EFNB2/LIMK1/ACTR3/CLTC/ROCK2/SDCBP/EPHA6/YES1/EPHA5/CDC42/PSEN1/
/SHC1/MAPK1/JAK2/STAT3/PIK3CB/MTOR/PTPN11/SIRPA/RPS6KA2/MAPK14/PTPN1/MAP2K1/E

/HMOX1/TRAF6/TAB3/NOD1/MAPK14/MAP3K7/CASP8/UBE2V1/TXNIP/CYLD/NFKB1/BCL2L1

9R/SPRED1/IL6ST/AKT3/CBL/PIAS3/IL5RA/CSF3R/CRLF2/PIK3R1/PIM1/OSM/CNTF/EP300/IFNA1/

R/CCNE2/RRM2B/PERP/TNFRSF10B/CDK1/CCNE1/TP73/CASP8/CDKN2A/CHEK1/MDM2/APAF1/C
S/PIK3R1/YWHAZ/RAP1B/CALML5/YWHAG/FOXO3/SOS1/GAB1/GSK3B/SHC1/RPS6KA3/MAPK1/T

4G3/ITGB2/CXADR/EIF4G2/HLA-DQA1/MYH1/SGCB/HLA-DPA1/HLA-DRA/EIF4G1/CASP8/ABL1/C

OXO3/CTNNB1/SPP1/SMAD3/BCL2L11/SMURF1/CBFB/TEAD3/PSMD5/PSMA6/PSMC1/PSMD2/R
SEC24C/IFNA1/NLRP3/HLA-G/NUP160/PIK3R4/JAK1/NUP153/SEC24D/SEC24B/STAT2/G3BP1/HS
AS/CBL/NRAS/PIK3R1/RBFOX2/SOS1/GAB1/SHC1/GALNT3/MAPK1/SPRED2/FLRT2/PTPN11/PPP2

/SHC1/MAPK1/STAT3/CDC42/RIT1/PTPN11/RAP1A/EHD4/MAP2K1/ABL1/CCND1/NTF4/RHOA/G
0AA1/CANX/KIR2DL4/HLA-DQA1/CD4/CTSL/HLA-DPA1/NFYA/KLRC1/HLA-DRA/CTSB/TAP1/HSPA
10D/TNFRSF10A/AKAP13/TNFSF10/NET1/FGD4/TAB2/TNFAIP3/RTN4/SOS1/BIRC2/TIAM2/BAG4/
9C2/SGK2/SLC9B1/TCIRG1/CLCNKB/ATP6V0A1/MCOLN2/ASIC1/ATP12A/TRPM6/ATP1A4/ATP6V

OR4N5/OR51D1/OR51S1/OR52E2/OR5AN1/OR6C76/OR8D1/OR9Q2/OR10K1/OR2AG1/OR4D2/C

CK1/IGHV2-70/IGLV7-43/REST/IGHV3-33/IGKV1-39/IGKV1-33/IGKV1D-39/IGHV2-5/IGLV6-57/AC
3/APC/MAPK1/CTNNA1/PIK3CB/ILK/PIK3R5/SOS2/MAP2K1/ELK1/CCND1/TCF7L2/AKT2

SL/TUBB/TUBB6/TUBA1B/TLR5/TUBA1C/ABL1/YWHAQ/PRKCA/CTTN/ARPC2/RHOA

P/BUB1B/ERCC6L/CDK1/SGO1/FBXW11/TPX2/RHOA/CDC14B/RAB1A/GOLGA2/CCNB1

A/PEX7/PAOX/TIMM17B/SGTA/COX17/ACOT8/HAO2/TIMM22/GFER/CYC1/CHCHD2/PMPCA/FXN

FGG/MAPK1/FXR1/JAK2/SPRED2/BCL2L11/MPRIP/SHOC2/RAP1A/MAP2K1/ESRP1/TLN1/AP3B1/

P160/CENPI/CKAP5/SPDL1/BUB1/SRGAP2/SGO2/INCENP/FMNL2/DIAPH2/PPP2R5E/NDE1/BUB1

/1-39/IGKV1-33/TAB2/IGKV1D-39/FCER1G/IGHV2-5/IGLV6-57/RASGRP4/FCER1A/IGHV3-30/RAS

ATF2/RAPGEF2/SHC1/RPS6KA3/MAPK1/MAP3K1/RPS6KA2/MAPK14/MAP3K5/MAP3K8/MAP3K1

:EB1/FGG/GSK3B/HSP90AA1/MAPK1/EGR1/MAPK14/NR4A1/POU2F1/MDM2/SMARCC2/CREBBP

NE2/CDK1/CCNE1/WASF1/TP73/CDC6/CDKN2A/RBL2/CREBBP/APAF1/CCNA2/RRM2/TFE3/PRMT

MRPL46/MRPL36/MRPL20/MTIF3/MRPL51/MRPS17/CHCHD1/MRPL58/MRPL2/MRPL43/MRPL5

RCA2/CDK2/BCL2L11/SIRT1/FAS/FBXO45/PLK3/CCNE2/PEA15/MAPK14/CDK1/TP73/CHEK1/MDN

D1/ROCK1/LEF1/FZD7/PPP3CA/VANGL1/PPP3R2/CSNK1A1/EP300/CTNNB1/DKK2/MMP7/ROCK2

O1/MAP3K8/NR4A1/CREBBP/YWHAQ/PRKCA/RCAN1/YWHAB/BCL2L1/MEF2D/MAPK8/PPP3R1/

)/KRAS/KIDINS220/NTRK1/RALB/NRAS/PIK3R1/EP300/DUSP7/CREB1/SOS1/GAB1/CLTC/RALA/AT

YT5/GNG7/CACNA2D2/CACNB2/CHRM3/KCNJ11/CACNA1A/ADCY8/ABCC8/PKLR/KCNC2/SNAP25

1/H4C8/H2BC21/H2BC5/H2AC6/H4C12/UBE2D1/H2AC14/CDK2/RPS6KA3/MAPK1/CDC27/STAT3/

G7/CACNA2D2/CACNB2/CHRM3/KCNJ11/CACNA1A/ADCY8/ABCC8/KCNC2/SNAP25/KCNB1

HSPA12A/BAG4/GSK3B/HSP90AA1/MAPK1/MTOR/SIRT1/ATR/BAG2/NDC1/DNAJB1/NUP107/GM

AC11/H4C8/H2BC21/H2BC5/RCOR1/H2AC6/H4C12/H2AC14/ARID4A/H2AC21/ARID4B/H2BC13/I

FOXO3/SMAD3/AGRP/BCL2L11/SIRT1/NFYA/FOXG1/RBL2/SMAD2/CREBBP/IGFBP1/YWHAQ/TXNI

P2B2/KCNIP2/FXYD1/CAV3/TMOD1/CACNA1H/SCN3B/FGF14/CACNA1I/MYL5/KCNK15/NPPA/RA

NB5/DDIT4/PTEN/ADORA2B/CX3CL1/PRKAA1/CDK2/MSH2/ACAD11/MTOR/LIF/FAS/DRAM1/RRM

C/NDUFV3/CYC1/NDUFS3/UQCRB/NDUFC1/UQCR10/NDUFA9/COX7A2/NDUFA1/NDUFB10/COX7

C5/H2AC6/H4C12/H2AC14/RB1/SMC2/H2BC13/H3C10/CDK1/NCAPD3/H2BC11/H4C14

/CAV3/PRKAR1B/SLC25A4/GABRA1/PHOX2B/SCN3B/TACR1/SSTR2/VIPR1/NKX2-2/AQP4/TPH1/L

PARD/GABPA/SIRT1/MEF2C/MAPK14/PPARG/NCOA1/MEF2D/TFAM/PPP3R1/PRKAB2/CAMK4

YNC1I2/KIF23/KIF2A/SEC24C/SPTBN2/CTSC/CLTC/KIF3C/SEC24D/SEC31A/CAPZA1/SEC24B/CANX/

0/H2BC21/H2BC5/KMT2A/H2AC6/NR4A3/H4C12/H2AC14/KMT2E/CBFB/H2BC13/H3C10/NFE2/K

ENPI/CKAP5/SPDL1/BUB1/STAG2/SGO2/INCENP/RAD21/PPP2R5E/NDE1/PDS5B/BUB1B/DYNC1H

MAPK1/TRAF6/VIM/CDC42/FAS/CD4/NFATC2/SH2B3/PTPN11/MAPK14/MAP3K8/BCL10/MAP2K1

A5/TNFAIP8L3/RAB5A/PIK3CB/PIK3R5/SYNJ1/PIKFYVE/SYNJ2/PIP4K2A/MTMR6/INPP5F/PLEKHA1

PGN/DAB2/SNX18/M6PR/PIK3C2A/ACTR3/CLTC/FNBP1L/SNX9/SGIP1/LRP2/EPS15/AGFG1/VAMP

V7-43/IGHV3-33/IGKV1-39/IGKV1-33/IGKV1D-39/IGHV2-5/IGLV6-57/IGHV3-30/IGHV1-2/IGHV4-3

32/USH1C/RAB3A/CHRNA10/STRC/PCLO/LHFPL5/OTOG/PCDH15/KCNN2/SYN1/CACNA2D2/BSN/

CACNA1B/GRIN2C/SLC25A4/CACNA1H/P2RX2/GNA14/CACNA1I/CACNA1S/TACR1/NOS2/SLC8A3/

/WRN/XRCC5/IFNAR2/ABL1/SMG6/CCND1/RPS6KB1/NFKB1/RAD50/SIN3A/MRE11/ATM/POT1/T

2/MAP3K2/KIDINS220/PRKAA2/NTRK1/PIK3R1/FOXO3/CTNNB1/CREB1/DOK5/SPP1/GSK3B/SHC1

51/HS6ST2/CHST9/GLYAT/GSTM2/GSTZ1/GLYATL1/CYP2D6/AKR1C4/GLYATL2/CYP2A6/UGT2B11/

A-G/BHMT2/VEGFA/CXCL11/GABPA/VIM/HLA-DQA1/FAS/C1QB/C3/C4A/AGTR1/HLA-DPA1/HLA-D

M6PR/CLTC/SGIP1/LRP2/EPS15/AGFG1/VAMP3/CHRM2/SCARB2/VAMP7/STAM2/COPS8/CD4/LDL

C2/SHC1/TRAF6/BCL2L11/PSEN1/XIAP/MYD88/APAF1/ZNF274/NTF4/MAPK8/RHOA/PRKACB

/H4C8/NCOA2/KAT6A/EP300/H2BC21/YEATS2/H2BC5/H2AC6/H4C12/PHF20/ATF2/H2AC14/ACTL

.68/H4C12/H2BC13/BARD1/H2BC11/H4C14/ABL1/BRCC3/ABRAXAS1/MAPK8/RAD50/BAZ1B/EYA
LC2/UGT1A4/GSTA1/GGT6/SLC26A1/UGT2B7/ACSM2B/TPST2/GSTT2/SULT1A2/SULT1A4/UGT2B

.B/UBE2D1/BIRC2/WWP1/HERC4/CDC27/UBE2NL/TRAF6/HUWE1/MAP3K1/UBE2Q2/TRIM32/UB

/DENND1B/RAB3IL1/RAB5A/RAB3GAP2/RAB10/RAB12/RAB3GAP1/RAB14/DENND2B/ALS2CL/RA

/ATP5F1A/TIMM17B/COX17/TIMM22/GFER/CYC1/CHCHD2/PMPCA/FXN/COX19/PAM16/COA6/T

F2A/KNL1/YWHAG/NUP160/CENPI/NEK2/CKAP5/SPDL1/BUB1/STAG2/ALMS1/SGO2/INCENP/RAD

i1/NCOA2/G0S2/MED13L/EP300/MED13/FADS1/NR1H4/MED1/SP1/TXNRD1/TIAM2/NCOA3/CLC
SL/TUBB/TUBB6/TUBA1B/TLR5/TUBA1C/ABL1/YWHAQ/PRKCA/CTTN/ARPC2/RHOA
B1/CREB1/SP1/NR4A2/ECE1/GSK3B/HSP90AA1/MAPK1/GNB1/GNAQ/MAPK14/MAP3K5/MAP2K

;/IL6ST/PPP3CA/PIK3R1/EDN1/CTNNB1/ROCK2/GSK3B/MAPK1/STAT3/PIK3CB/IGF1R/MTOR/LIF/

YH10/KRAS/MYH13/AKT3/PARD6B/CLDN8/NRAS/TJP1/PPP2R2C/ASH1L/CTNNB1/PTEN/MYH15/

TZ1/CYP2D6/ADH1C/CYP2A6/UGT2B11/UGT1A4/GSTA1/ADH6/UGT2B7/CYP2A7/CYP2C9/GSTT2/

/ASIC1/TRPM6/RPS27A/TRDN/ASIC5/SCNN1D/UBA52/ASIC4/ANO9/STOML3/ANO7/WNK2/UNC8

/H4C8/CHMP2B/H2BC21/H2BC5/NUP160/NUP153/H2AC6/H4C12/H2AC14/H2AC21/NDC1/H2BC

/IL36B/PF4V1/CXCL14/CXCL6/TNFSF13B/CCL18/IL1RN/CXCL10/OSM/MMP3/CNTF/IFNA1/XCL1/C

MP3/PTGER1/PRLH/GNG8/CRHR1/OXER1/LPAR2/ACKR1/GNB3/CRHBP/FSHB/FFAR3/GPRC6A/MC

P/ATF2/PELI1/USP18/ATF1/RPS6KA3/MAPK1/TRAF6/MAP3K1/TAB3/MEF2C/RPS6KA2/PPP2CB/N

L/H2BC5/FOXO3/H2AC6/H4C12/APP/H2AC14/CAPN2/GSDME/BCL2L11/LMNB1/H2BC13/H3C10/

3/SMC1A/WRN/CDKN2A/NUP58/NUP205/PIAS1/SMC6/NUP98/SUMO3/TPR/RANBP2/TDG/PML/

RP2/HECW1/USP34/H2AC7/H2BC14/SOST/H2BC4/SOX4/FZD1/LEF1/H2BU1/SMURF2/H4C8/CSNK

l/EP300/H2BC21/H2BC5/TET2/H2AC6/H4C12/TET3/H2AC14/GSK3B/GTF2H2/TAF1B/SIRT1/ARID4

/LCP2/CBLB/RASGRP1/NFATC3/GSK3B/MAPK1/PIK3CB/DLG1/CDC42/CD4/NFATC2/PIK3R5/MAPK

34GALT1/H2BU1/NOTCH3/RAB6A/H4C8/EP300/H2BC21/H2BC5/E2F3/H2AC6/H4C12/H2AC14/AG

GFR/H2BU1/DYNC1LI2/H2AC11/H4C8/DYNC1I2/CHMP2B/H2BC21/H2BC5/NUP160/CREB1/NUP1

AP1/PIK3C2A/FOXO3/PIK3R4/SOS1/MAPK6/CBLB/MAP4K3/GSK3B/SHC1/SLC16A2/PRKAA1/RHOC
3/IGF1R/MTOR/GNAQ/EGR1/ADCY3/MAPK14/RAP1A/MAP2K1/CCNE1/RBL2/PLD1/RPS6KB1/PAX

3/MEF2C/RPS6KA2/PPP2CB/NOD1/MAPK14/MAP3K8/MAP2K1/MAP3K7/ELK1/UBE2V1/FBXW11

AL3/PRKAR1B/GLRA2/GABRA1/GNG4/TUBA3C/GABRG2/HTR3E/GRIA1/GNG7/CHRNA2/TSPAN7/

/PTPN12/IKBIP/IL1RN/TAB2/APP/SMAD3/PELI1/USP18/PTPN9/TRAF6/STAT3/TAB3/PTPN11/NOD

CCR1/ROCK1/ADCY7/CXCL14/CXCL6/KRAS/AKT3/CCR2/NRAS/CCL18/PIK3R1/CXCL10/RAP1B/XCL1
DPEP3/ADH7/CYP2S1/UGT2B10/GLYAT/GSTM2/UGT3A1/GSTZ1/GLYATL1/ACY3/FDXR/CYP2D6/AI

MLNR/CXCL3/QRFPR/CXCL2/SAA1/CORT/CCK/KISS1R/GAL/PROK1/CCL25/KNG1/C5/TACR2/AGTR2

C14/PHC3/CDK6/H2BC4/RUNX1/GATA3/H2BU1/TCF12/H4C8/TJP1/EP300/H2BC21/H2BC5/ELF1/F
/AGO2/GNB1/TNRC6B/AGO3/MAP3K7/AGO4/PRKCA/GNAT2/CAMK2A/TCF7L2/ITPR1/PPP3R1/PD

!/DVL3/TEK/CSNK1G1/MAP3K7/PPARG/LRP6/CCND1/PRKCA/TCF7L2/MAPK8/RHOA

IRH2/TACR1/CCL21/SSTR2/GPR18/KEL/MLNR/CXCL3/QRFPR/ADRA2C/CXCL2/SAA1/CORT/CCK/FI

ADH1C/AKR1C4/UGT2B11/UGT1A4/GSTA1/ADH6/UGT2B7/CYP2C9/GSTT2/DHDH/UGT2B4/CYP2E
AA2/PIK3R1/CAP1/PIK3C2A/FOXO3/PTEN/PIK3R4/SOS1/GAB1/MAPK6/CBLB/MAP4K3/SNAP23/G

!/UGCG/PRKD1/NEU2/PLPP3/SPTSSB/CERT1/SPHK1/GALC/SGPP1/DEGS1/SPTLC3/UGT8/SPTLC2/

'FGD4/RTN4/SOS1/TIAM2/ARHGEF6/TRAF6/ARHGEF17/BCL2L11/PSEN1/SOS2/MYD88/PLEKHG5/

LOCK/SIRT1/MEF2C/XPO1/TBL1XR1/ARNTL/RAI1/CREBBP/NCOA1/NCOA6/NR1D1/CRTC3/MEF2[illegible]

[illegible]21/NBN/H2BC5/H2AC6/H4C12/H2AC14/STAG2/BRCA2/RAD21/CDK2/SYNE2/RBBP8/LMNB1/AT[illegible]

TET3/CDON/IGF1R/GRID1/MTOR/DLX5/MEF2C/SMC3/UBE3A/FOXG1/TAP1/TAF1/SGK1/CAMK2[illegible]

1/CDK2/TP53BP2/TOPBP1/MTOR/RICTOR/RBBP8/ATR/PLK3/PPP2CB/PIP4K2A/MAPK14/DYRK2/
YAT/UGT3A1/GLYATL1/CYP2D6/GLYATL2/UGT2B11/UGT1A4/GSTA1/GGT6/UGT2B7/ACSM2B/CY

21/H2BC5/H2AC6/H4C12/H2AC14/GSK3B/TAF1B/TAF1A/H2BC13/SF3B1/H3C10/DEK/H2BC11

AO1/RGS7/SLC8A3/FXYD2/CACNA1C-IT2/RGS9/GJA8/GNG7/ADRB1/CASQ2/CHRM1/GNAZ/CHRM

/GAB1/CBLB/GSK3B/SHC1/MAPK1/PIK3CB/MTOR/PIK3R5/HBEGF/SOS2/MAP2K1/ELK1/ABL1/PR

NAP23/VAMP7/AP3S1/TGOLN2/BLOC1S6/AP4E1/GOLGB1/CLINT1/AP3B1/GNS/TFRC/CTSZ/HSPA

K3CB/SPHK1/CDC42/FCGR2C/PIK3R5/WASL/ARF6/PIKFYVE/MAP2K1/DNM3/WASF1/PIP5K1A/MA
K14/DYRK2/CSNK1G1/CDKN2A/CHEK1/MDM2/ABL1/CREBBP/CCNA2/MAPK8/PRMT5/ATM

CXCL14/KRAS/AKT3/CCR2/NRAS/PIK3R1/CXCL10/RAP1B/XCL1/FOXO3/SOS1/CCR3/CCL17/ROCK2

BN/H2BC5/TP53BP1/RNF168/H4C12/PPP4R2/BRCA2/CDK2/TOPBP1/RBBP8/POLQ/ATR/H2BC13/

2/NFATC2/PRKAR1A/MEF2C/HBEGF/MAPK14/MAP2K1/NKX2-5/PRKAR2A/CREBBP/IGF1/RPS6KB

C12/H2AC14/STAG2/RAD21/SYNE2/LMNB1/ATR/H2BC13/SMC3/SMC1A/H2BC11/SUN1/H4C14/S

DL1/BUB1/UBE2D1/SGO2/INCENP/CDC27/PPP2R5E/NDE1/BUB1B/DYNC1H1/ANAPC1/CLASP1/E

1/RNF168/H4C12/PPP4R2/BRCA2/CDK2/TOPBP1/RBBP8/POLQ/ATR/H2BC13/RAD51AP1/ERCC4/

BCL2L11/FAS/XIAP/TNFRSF10B/TP73/CASP8/CDKN2A/CFLAR/MDM2/APAF1/IGF1/NFKB1/BCL2L1

BU1/H4C8/H2BC21/TNIK/H2BC5/E2F3/H2AC6/H4C12/H2AC14/MAPK1/RNF2/H2BC13/MAPK14/

RAMP1/UCN/WNT10B/GNG4/CALCB/WNT6/IHH/VIPR1/WNT3A/SCTR/GNG7/VIPR2/CALCA/WNT

G/H2BC5/TP53BP1/MCM4/RNF168/H4C12/CDK2/TOPBP1/RBBP8/ATR/MCM10/H2BC13/WRN/

/GAB1/CBLB/GSK3B/SHC1/MAPK1/PIK3CB/BCL2L11/MTOR/HBEGF/SOS2/MAP2K1/ELK1/MDM2/

[illegible]NPTAB/GALNS/SCARB2/GALC/AP3S1/CTSL/AP4E1/CTSB/TPP1/PPT1/CLTCL1/HGSNAT/ATP6V0D2

N/ERO1B/FFAR4/GH1/KIF5A/CPB2/CMA1/PCSK1/SEC11C/POMC/CDX2/INHA/CPB1

NG8/CRHR1/GNB3/FSHB/MC3R/PRKAR1B/RAMP1/GNG4/P2RY11/GPR83/RLN2/CALCB/VIPR1/SC

[illegible]RK2/WRN/BARD1/TAF7L/TAF1/CHEK1/MDM2/TAF4B/CCNA2/TPX2/BRIP1/RAD50/SUPT16H/PR[illegible]

[illegible]1/TGOLN2/BLOC1S6/AP4E1/GOLGB1/CLINT1/AP3B1/TFRC/HSPA8/AP1G1/TXNDC5

ᵖB/CLEC4M/REG4/CLEC18A/LGALS2/OVGP1/REG1B/C1QL1/FCN2/CLEC4G/ITLN2/CLEC17A/ITLN1,

2G10/PLA2G2D/CALML6/PLA2G12B/ADCY8/PLA2G2A/CAMK2B/CALML3/PLA2G1B

13A1/ITGA5/COL6A1/LAMA4/VCAM1/COL4A5/ITGA6/LAMC1/ITGA3/COL4A4/ITGA4/LAMB1/ITG
GB5/ITGA5/COL6A1/HSPG2/VCAM1/COL5A3/COL4A5/ITGA6/ITGA3/COL4A4/COL4A2/ICAM1/ITG
8A2/COL4A1/COL17A1/P4HA1/COL24A1/ADAMTS3/COL6A1/COL5A3/SERPINH1/LOXL3/COL4A5/
4A1/COL6A1/COL5A3/LOXL3/COL4A5/ITGA6/COL4A4/COL4A2/BMP1/LAMB3/COL14A1/DST/CO

L6A1/HSPG2/LAMA4/COL5A3/ITGA6/LAMC1/ITGA3/COL4A4/COL4A2/ITGA4/LAMB1/ITGA1/CD4

B3/DSPP/ITGB5/DCN/COL6A1/HSPG2/LAMA4/COL5A3/COL4A5/LAMC1/COL4A4/COL4A2/LAMB
A5/ITGA6/LAMC1/COL4A4/COL4A2/LAMB1/LAMB3/AGRN/PDGFB/ACTN1/NTN4/ITGB4/LAMB2

D4/TNS4/PPP1R14C/CDC42EP3/TNFAIP3/ACKR3/SRGN/MFGE8/CPEB4/BIRC2/THBD/KTN1/HSP90

4/VCL/IRS1/TGFBR2/COL14A1/ROCK1/ADGRA2/COL6A2/COL15A1/CBL/PIK3R1/CSF1R/PIK3C2A/

COL17A1/ADAM17/COL6A1/COL5A3/MMP12/COL4A5/COL4A4/COL4A2/MMP19/ADAM10/COL1

1/COL5A3/SERPINH1/COL4A5/COL4A4/COL4A2/PLOD1/BMP1/P4HA3/COL14A1/COL6A2/COL15
P1CB/USP9X/SP1/SMAD3/UBE2D1/SMAD7/SKI/PPP1R15A/ATP1B4/MAPK1/TRIM33/SMURF1

C/ITGB3/CAV2/PDGFRB/MYLK/ITGB5/ITGA5/PIK3CA/PIK3CG/COL6A1/LAMA4/PDGFRA/HGF/CO

FRP2/SERPINH1/CXCR2/ICAM1/TLR7/NOD2/CXCL14/TLR3/TGFB3/TLR1/TLR2/MMP3/LGALS1/TN
MP11/ADAM9/MMP16/COL4A1/MMP14/SCUBE3/NID1/CTSK/COL17A1/TIMP2/ADAMTS5/ADAM

TS1/EP300/PDK1/RNF111/SPTBN1/SOS1/ITCH/NUP153/ZFYVE16/ITGB4/SP1/APP/SMAD3/ATF2/

CBL/TGFBR1/CCNK/SMAD1/FSTL3/EP300/RNF111/PPP1CB/INHBB/USP9X/ZFYVE16/SP1/SMAD3/
5/ITGA5/PIK3CA/LAMA4/PDGFRA/HGF/COL5A3/ITGA6/LAMC1/BIRC3/ITGA3/COL4A4/VCL/COL4

IF2AK2/DDX58/CD300A/PIK3R1/EP300/TFAP2A/HLA-G/ACTN1/MFGE8/CLTC/RASA2/MERTK/TOP
P1/CTHRC1/SRPX2/MFAP5/ECM2/MEPE/MFAP3/NDNF/NID1/TNC/SPON1/AEBP1/EFEMP1/CRIS
OCK1/NCK1/PLXNA1/ROBO2/KRAS/ARHGEF12/PLXNC1/PPP3CA/PAK2/PPP3R2/EPHB2/EFNB2/NR
/RPS17/RPL24/RPL14/RPLP0/RPS27A/RSL24D1P11/RPS20/RPL7A/RPS3A/RPS7/RPS11/RPS19/RP
RPL12/RPL36A/RPS17/EEF1B2/RPL24/RPL14/RPLP0/RPS27A/RPS20/RPL7A/RPS3A/RPS7/RPS11/

RPL36A/RPS17/RPL24/RPL14/RPLP0/RPS27A/RPS20/RPL7A/RPS3A/RPS7/RPS11/RPS19/RPL3/RP
PS13/RPL12/RPL36A/RPS17/RPL24/RPL14/RPLP0/RPS27A/RPS20/RPL7A/RPS3A/RPS7/RPS11/RPS
/RPS13/RPL12/RPL36A/RPS17/RPL24/RPL14/RPLP0/RPS27A/RPS20/RPL7A/RPS3A/RPS7/RPS11/
L17/RPS13/RPL12/RPL36A/RPS17/RPL24/RPL14/RPLP0/RPS27A/RPS20/RPL7A/RPS3A/RPS7/RPS
4/RPL17/RPS13/RPL12/RPL36A/RPS17/RPL24/RPL14/RPLP0/RPS27A/RPS20/RPL7A/RPS3A/RPS7/
PS13/RPL12/RPL36A/RPS17/RPL24/RPL14/RPLP0/RPS27A/RPS20/RPL7A/RPS3A/RPS7/RPS11/RP
1/ITGA4/COLEC12/IGKV2-28/RAET1E/LILRA6/IGHV2-70/IGLV7-43/SIGLEC7/TREML4/IGHV3-33/C
GA3/MSN/VCL/FGF10/GNG12/ITGA4/FGF5/ENAH/ITGA1/EGFR/ACTN4/SSH1/ROCK1/NCKAP1/M
33/RPL31/RPS4X/RPS14/RPL17/RPS13/RPL12/ATP6V0B/WDR24/RPL36A/RPS17/RPL24/RPL14/R
31/RPS4X/RPS14/RPL17/RPS13/RPL12/RPL36A/RPS17/RPL24/RPL14/RPLP0/RPS27A/RPS20/RPL7
6/RPL35A/RPS23/IMP3/RPL10A/SENP3/RPP25/RPS8/EMG1/RPL11/RRP1/BUD23/RPS3/RPL23/FA

TSHB/RPL11/RPS3/RPL23/PSMB6/CSAD/FAU/GLDC/RPL27/OAZ3/ASMT/RPL7/ODC1/ECHS1/AMT
RPRB/MRPL14/MRPL40/RPS4Y1/MRPL46/RPS16/MRPL36/RPL35A/RPS23/MRPL20/EIF3K/CARS2

A4/COL4A2/WNT10A/CLDN16/EIF5A2/FZD1/TGFBR2/NOTCH3/FZD7/JAG1/TGFB3/KRAS/AKT3/TG
/NRAS/PIK3R1/RAP1B/TNS4/SOS1/GAB1/LAMB2/EPS15/SHC1/TNS3/STAT3/STAM2/STAM/PTPN1
PS15A/RPL31/RPS4X/RPS14/RPL17/RPS13/RPL12/RPL36A/RPS17/RPL24/RPL14/RPLP0/RPS27A/[illegible]
4/STK3/CDH13/KRAS/PAK2/NTRK1/NRAS/FLT1/ITGAM/CXCL10/CSF1R/PPP1CB/LATS1/CTNNB1/[illegible]

ARHGAP21/JAG1/DIAPH3/DOCK7/GNA13/ABL2/PAK2/ARHGAP31/VANGL1/ERBIN/ARHGAP32/N[illegible]
/ROCK2/SP1/SMAD3/SMAD7/GDF5/MAPK1/PITX2/ACVR1C/NODAL/SMURF1/PPP2CB/SMAD5/A

/ACTR3/PTEN/SOS1/GAB1/SHC1/WASF2/RPS6KA3/YES1/MAPK1/RAB5A/JAK2/STAT3/PIK3CB/SP[illegible]
/PRELP/ADAMTS4/SLC26A2/MUC21/ADAMTSL4/B4GALT1/GPC6/NOTCH3/ADAMTS6/AGRN/BGN
L1/ERBIN/ARHGAP32/ARHGAP28/AKAP13/PIK3R1/ARHGAP11A/MYO9A/NET1/PKN2/CAVIN1/SL[illegible]
5/VRK2/SRGAP2/LEMD3/CYBB/SNAP23/SYDE1/GARRE1/VAMP3/WASF2/SWAP70/LMAN1/CDC4[illegible]

RPINB5/DDIT4/PTEN/E2F3/SPP1/SP1/NLRC4/RB1/CX3CL1/ATF3/APC/MSH2/MCL1/TP53BP2/DU[illegible]

N4/LAMB3/CD2AP/NCK1/CDH13/DST/FLNC/FYB1/PARD6B/CLDN8/SKAP2/PIK3R1/SPTBN1/CTNN
11/ARF6/NUMB/SH3KBP1/RAP1A/PTPN1/MAP2K1/RANBP9/RIN2/DEPTOR/EIF4E/AKT2/MAPK8/[illegible]
1/PTEN/CREB1/SOS1/JAK1/GAB1/ITCH/RALA/SP1/CBLB/EPS15/SHC1/NCOA3/ATF1/ELK4/RPS6KA
KT3/GREB1/STRN/H4C8/NCOA2/EPGN/NRAS/PIK3R1/FKBP5/S1PR3/MMP3/EP300/H2BC21/GTF[illegible]

D5A/IGKV1D-39/IGHV2-5/LIMK1/WIPF1/IGLV6-57/ACTR3/IGHV3-30/DOCK1/IGHV1-2/IGHV4-39/[illegible]
DE1/GARRE1/VAMP3/WASF2/SWAP70/LMAN1/CDC42/ABI2/CYFIP1/OPHN1/RACGAP1/GIT2/AR[illegible]

UP153/EIF4G3/KPNA3/SMAD7/STAT2/FLNA/USP18/SP100/GBP2/TRIM5/GBP3/SAMHD1/JAK2/K

AIN/IGHV2-70/ANGPT1/IGLV7-43/SLC16A1/SLC16A3/KRAS/IGHV3-33/CD84/SLC7A5/TNFRSF10D/
NDUFA9/NDUFA1/NDUFB10/COX7B/SURF1/COX6C/COX6B1/ATP5F1E/NDUFB1/COX14/NDUFA2/

MP11/ADAM9/MMP16/MMP14/ADAM19/ADAMTS20/CTSK/F13A1/TIMP2/P4HA1/CST6/ADAM[illegible]

/MEF2C/DCP1A/FOXG1/NKX2-5/SMAD2/CREBBP/HSPA8/NCOA1/TFE3/SIN3A/TFDP1/NCOR1/IF[illegible]
/IGHV1-2/IGHV4-39/SHC1/IGKV3-15/PIK3CB/IGKV1D-16/IGHV1-46/IGKV3-20/IGKV2-30

3/MYH10/KRAS/GNA13/PAK2/NRAS/PIK3R1/PDGFB/PIK3C2A/LIMK1/PIK3R4/MRAS/SOS1/ACTN[illegible]

/SURF1/ATP5IF1/COX6C/SLC25A6/COX6B1/ATP5F1E/NDUFB1/NDUFA2/ATP5MC2/NDUFV1/COX[illegible]

PS6KA3/MAPK1/RAB5A/MAP3K1/STAT3/PIK3CB/MTOR/RICTOR/DUSP1/CDC42/EGR1/WASL/ME[illegible]

WNT11/SMAD3/GSK3B/SHC1/APC/MAPK1/MAP3K1/PIK3CB/PPP2R5E/BCL2L11/CDC42/WNT5B/[illegible]

IAN1/DIAPH1/OPHN1/FMNL3/RACGAP1/DAAM1/ARHGEF10/ARHGAP1/TFRC/MYO9B/RHOA/TJP

NND1/PLEC/PARVA/CTNNB1/ACTN1/ITGB4/PARD6G/CDH1/FLNA/ARHGEF6/CTNNA1
A/IGHV3-30/IGHV1-2/NFATC3/IGHV4-39/SHC1/IGKV3-15/IGKV1D-16/IGHV1-46/IGKV3-20/IGKV2
/RPS20/RPL7A/RPS3A/RPS7/RPS11/RPS19/RPL22L1/RPL3/RPS18/RPS2/RPL19/UBA52/RPL13A/R
2/PAK2/ARHGAP31/ITSN1/VANGL1/ARHGAP32/PIK3R1/PLEKHG1/FGD4/DOCK11/CDC42EP3/WI[illegible]
GHV3-30/IGHV1-2/IGHV4-39/SHC1/MAPK1/IGKV3-15/MAP3K1/IGKV1D-16/IGHV1-46/IGKV3-20/[illegible]

5/ITGB2/CAPN2/SHC1/MAPK1/CDC42/ILK/RHO/RAP1A/GIT2/MAP2K1/ITGA8/TLN1
37A2/NRIP1/CDKN2B/EFNA5/SPRR1B/ALPG/KRT34/STS/FOXO1/PTHLH/VDR/LRRC8A/CREG2/BCL[illegible]

COX7A2/NDUFA1/NDUFB10/COX7B/COX6C/ATP12A/COX6B1/ATP5F1E/NDUFB1/COX7B2/NDUF[illegible]
RC2/CDH1/GSK3B/SHC1/VEGFA/YES1/MAPK1/JAK2/TFF2/STAT3/HDC/MTOR/GNAQ/CDC42/EGR[illegible]

JRF1/COX6C/COX6B1/NDUFB1/COX14/NDUFA2/NDUFV1/COX8A/NDUFB2/COX5A/COX6A1/NDU

C42/PIK3R5/MAP2K1/CDKN2A/SMAD2/PLD1/CCND1/NFKB1/AKT2/BCL2L1/MAPK8

CTR3/IGHV3-30/DOCK1/IGHV1-2/IGHV4-39/WASF2/YES1/MAPK1/IGKV3-15/IGKV1D-16/IGHV1-46

1/MAPK1/CREB3L2/PIK3CB/IGF1R/MTOR/CCNE2/PIK3R5/SOS2/MAP2K1/CCNE1/NKX3-1/PDGFD/
ISPH1/IGKV1-39/SSC5D/IGKV1-33/IGKV1D-39/IGHV2-5/IGLV6-57/IGHV3-30/IGHV1-2/IGHV4-39/I
P23/VAMP3/ARHGEF17/DIAPH1/OPHN1/RACGAP1/STARD8/DAAM1/ARHGEF10/ARHGAP1/TFRC/

A1/NRAS/POLK/PIK3R1/NBN/CTNNB1/PTEN/SOS1/E2F3/WNT11/SP1/TNFSF11/RB1/GSK3B/SHC1

/PIM1/OSM/MMP3/FOXO3/JAK1/ITGB2/VEGFA/HMOX1/HSP90AA1/MCL1/JAK2/STAT3/LIF/VIM
5/ASCC3/SMAD1/FOXC1/PHF6/FOXC2/LATS1/TRERF1/SMAD3/EMSY/TBX3/EXT2/EXT1/NFE2L2/W

HV3-30/IGHV1-2/IGHV4-39/IGKV3-15/PIK3CB/IGKV1D-16/IGHV1-46/IGKV3-20/IGKV2-30

/UQCRB/NDUFC1/COX19/UQCR10/BSG/HAGH/NDUFA9/NDUFA1/NDUFB10/COX7B/SURF1/COX6

21/C4B/GRIN2A/C1QC/H2BC5/ACTN1/H2AC6/H4C12/H2AC14/H2AC21/HLA-DQA1/FCGR2C/C1QE

A1/TRIM22/OAS1/HLA-DRA/PTPN1/PTAFR/HLA-DQB2/FCGR1A/B2M/SOCS3/PIAS1/MID1/CAMK2

PN1/MAP2K1/ELK1/SOCS3/CCND1/NCOA1/EIF4E/RPS6KB1/NFKB1/BCL2L1/MAPK8/RHOA
/HAS2/HS3ST2/CHST6/GPC4/CHST15/HMMR/B3GNT2/GALNS/EXT2/EXT1/NDST3/ST3GAL4/SDC4
GB2/PELI1/USP18/GSDME/ATF1/RPS6KA3/MAPK1/TRAF6/TLR9/MAP3K1/TANK/TAB3/CTSL/PTPI

11A/MFGE8/DLGAP5/MERTK/ITGB2/PARD6G/CX3CL1/SERPING1/PROS1/CX3CR1/FAS/C1QB/C3/
MAD3/ATF2/STAT2/NFATC3/MAPK1/TRAF6/DDX3X/JAK2/MAP3K1/STAT3/CREB3L2/PIK3CB/FAS/I

C21/ADAMTSL4/ST3GAL1/CHST4/ADAMTS6/GALNT5/ADAMTS9/GALNT10/B3GLCT/ADAMTS16/G
./OSM/HMGA1/FOXC2/CYP26A1/CTNNB1/CREB1/PNPLA3/MBNL1/SP1/SMAD3/BMP3/RB1/STAT

AM/SIGLEC1/HLA-G/ITGA9/CD276/ITGB2/CD226/CDH1/SDC4/CD58/OCLN/HLA-DQA1/CD4/HLA-D
JBE2E1/MX2/NUP58/NUP205/EIF4E/NUP98/RNASEL/TPR/RANBP2/UBC/KPNB1/PPM1B/KPNA7/I

NFRSF10B/MYD88/PRKAR2A/CASP8/CFLAR/APAF1/NFKB1/AKT2/BCL2L1/PRKACB/DFFA/PPP3R1/C

0/FCER1G/IFNA1/HLA-G/SOS1/LCP2/KLRD1/SH2D1B/ITGB2/NFATC3/SHC1/MAPK1/PIK3CB/IFNG

GFB/SMAD1/CTNNB1/PTEN/SOS1/JAK1/GAB1/WNT11/MAPK6/SMAD7/GSK3B/APC/MAPK1/STA
DUFA1/NDUFB10/COX7B/COX6C/SLC25A6/COX6B1/ATP5F1E/NDUFB1/COX7B2/SLC6A3/SEPTIN5

P5PO/NDUFB8/NDUFS8/NDUFV2/NDUFB9/ATP5MC1/NDUFA3/NDUFB7/NDUFA7/NDUFS6/NDU

1/PELI1/H1-4/GSDME/SDCBP/APC/HSP90AA1/MAPK1/OCLN/TP53BP2/OPA1/STAT3/KPNA1/BCL

H4C14/YWHAQ/BRCC3/ABRAXAS1/YWHAB/BRIP1/RAD50/RFC3/MRE11/ATM/UBE2V2/CCNB1

1G/IGHV2-5/IGLV6-57/RASGRP4/SOS1/LCP2/FCER1A/IGHV3-30/RASGRP1/UBE2D1/IGHV1-2/NFA

7/KCNK16/GNG7/KCNN2/KCNJ10/KCNJ6/KCNV1/KCNK10/KCNH3/KCNJ11/ABCC8/HCN2/KCNK3/K

IGHV1-2/IGHV4-39/YES1/IGKV3-15/IGKV1D-16/IGHV1-46/IGKV3-20/IGKV2-30/ADCY3/PRKAR1A

B3/FLNC/KRAS/MAP3K2/PPP3CA/AKT3/PAK2/PPP3R2/TGFBR1/NTRK1/LRRK2/NRAS/CACNA1E/PI

2/SMURF1/CBFB/DLX5/PSMD5/PSMA6/PSMC1/CDK1/PSMD2/ABL1/HIVEP3/BMP2/CCND1

G2/INCENP/RAD21/SATB2/SMC5/ESR1/RNF2/NDC1/NUP107/SMC3/SMC1A/WRN/SENP1/TOPOR

UFV2/NDUFB9/NDUFB11/NDUFA3/NDUFA13/NDUFB7/NDUFA7/NDUFS6/NDUFA11/NDUFS7

MDM2/CREBBP/TGFB1I1/ZMIZ1/CCND1/PIAS1/NCOA1/ETV5/BMF/RHOA/SIN3A/FHL2/RNF6/CAR

COX5B/UQCR11/CACNA1S/CACNG6/FXYD2/ACTC1/CACNA2D2/CACNB2/ATP1A2/MYH6/TNNT2/A

/USP9X/PTEN/TNKS2/H2AC6/USP25/SMAD3/H2AC14/SMAD7/BIRC2/USP18/TRAF6/CCP110/H2A

3/NOD1/MAPK14/MAP3K8/MYD88/MAP2K1/MAP3K7/CASP8/ELK1/UBE2V1/USP14/FBXW11/NF

MAPK1/OCLN/TP53BP2/OPA1/STAT3/KPNA1/BCL2L11/VIM/STK24/FAS/LMNB1/DCC/XIAP/TNFRS

B/CSF1R/SH3GLB1/HLA-G/HSPA1B/ITCH/CLTC/EHD3/CBLB/PARD6G/WWP1/RAB11FIP5/EPS15/R

MAPK14/MAP3K8/MYD88/MAP2K1/MAP3K7/CASP8/IFNAR2/TLR5/IFNAR1/TBK1/NFKB1/AKT2/N

/RAI1/CREBBP/NCOA1/CRY1/NCOA6/NR1D1/CRTC3/MEF2D/ELOVL3/USP46/NPAS2/CARM1/UBC

R9/STAT3/PIK3CB/FAS/CCNE2/ADAR/OAS1/EIF2AK4/MYD88/CCNE1/TP73/MAP3K7/CASP8/MX1/

D1/MAPK14/MAP3K8/MAP2K1/MAP3K7/CASP8/ELK1/UBE2V1/USP14/TBK1/FBXW11/NFKB1/M
ALBP1/CDC42/MAP2K1/CDKN2A/SMAD2/PLD1/CCND1/RPS6KB1/NFKB1/AKT2/BCL2L1/MAPK8/

/MAPK14/MAP3K8/MYD88/MAP2K1/MAP3K7/CASP8/IFNAR2/TLR5/IFNAR1/TBK1/NFKB1/AKT2/M
MC1A/CDK1/CCNE1/TTK/CDC6/CDKN2A/CHEK1/MDM2/RBL2/ABL1/SMAD2/CREBBP/YWHAQ/C

CNMA1/KCNH5/ACHE/GABBR2/KCNH8/SLC6A3/HTR3A/PICK1/KCNJ4/KCNQ1/GNG8/TUBB8/CAC

MNB1/NEK6/NDC1/PPP2CB/H2BC13/NUP107/CNEP1R1/H3C10/CDK1/NCAPD3/H2BC11/BLZF1/H

B/NOS1/COX6C/HSD17B10/BAD/COX6B1/ATP5F1E/NDUFB1/COX7B2/NDUFA2/ATP5MC2/NDUF

MSY/EXT1/CAND1/APC/WDHD1/TRIM5/ELK4/GDF3/ZNF462/RFX7/NODAL/RARG/BCORL1/CUL4B

CDK1/CCNE1/TTK/CDC6/CDKN2A/CHEK1/MDM2/RBL2/ABL1/SMAD2/YWHAQ/CCND1/CCNA2/M

RNA2/TACR2/AGTR2/CHRNA4/ADRB1/CHRM1/GALR3/CHRNB2/UTS2R/CHRM3/GRPR/CHRNB3/

ACTN1/ROCK2/GNAI3/ITGB2/CYBB/CTNNA1/OCLN/PIK3CB/EZR/CDC42/MYL10/PIK3R5/PTPN11/

N4/FOXO3/ROCK2/KLK8/LILRB3/RB1/CDK2/MAPK1/VIM/CDC42/C1QB/EGR1/SLIT3/ZFP36/NR4A

K2/ROCK2/WNT11/SERPINF1/NFATC3/ROR2/GSK3B/APC/WNT5B/PRICKLE2/NFATC2/DVL3/DAAI

KCNK16/FXYD4/FGF13/KCNE2/KCNK12/CACNA2D2/CASQ2/CACNB2/ATP1A2/FXYD7/KCNK10/KCI

OR2AG1/OR4D2/OR4F4/OR4K15/OR51H1/OR52A5/OR5B17/OR5D16/OR5F1/OR5H2/OR5L1/OR6

C12/UBE2D1/H2AC14/RB1/H1-4/CDK2/RPS6KA3/MAPK1/CDC27/STAT3/RNF2/LMNB1/ANAPC1/I

APK1/DLG1/EZR/RPS6KA2/CNTN1/NUMB/TUBB6/MAP2K1/TUBA1B/DNM3/KCNQ3/RANBP9

FB1/DMAC1/NDUFA2/NDUFV1/NDUFB2/NDUFB8/ECSIT/NDUFV2/NDUFB9/NDUFB11/NDUFA3/N

A31/UQCR10/NDUFA9/COX7A2/DNALI1/NDUFA1/NDUFB10/COX7B/CREB3L3/COX6C/SLC25A6/C

/ARVCF/DGCR5/ACTC1/LINC01637/USP41/DRD2/LRRC74B/PRODH/LINC00896/FGF8/TUBA3FP/P

TB33/PPP2R5E/WNT5B/LDLR/DVL3/NFYA/PPP2CB/RACGAP1/NKD1/MAP3K7/CREBBP/LRP6/CCN

12/ATF2/H2AC14/AGO2/STAG2/NCOA3/RAD21/HSP90AA1/CHD1/ESR1/CBFB/H2BC13/SMC3/SN

A3C/STXBP1/GABRG2/ABAT/SLC1A6/TSPOAP1/GAD1/HTR3E/GRIA1/GNG7/CHRNA2/TSPAN7/SYN

/H4C12/H2AC14/H2AC21/DYNC1H1/NDC1/H2BC13/NUP107/TUBB6/TUBA1B/H3C10/TBL1XR1/H

IL22RA2/SOS1/JAK1/CBLB/CSF2RA/STAT2/IL7/JAK2/STAT3/PIK3CB/SPRED2/IFNGR1/STAM2/LIF/

TRAF6/MAP3K1/PIK3CB/CDC42/PSEN1/PIK3R5/SH2B3/PTPN11/RPS6KA2/MAPK14/SOS2/RAP1A/

P90AA1/TRAF6/TAB3/PTPN11/NDC1/NOD1/NUP107/G3BP2/SIKE1/MAP3K7/IFNAR2/UBE2V1/NU

/ARHGEF6/TRAF6/ARHGEF17/BCL2L11/SPPL2A/FAS/PSEN1/TAB3/XIAP/TNFRSF10B/SOS2/MYD88/

0B/RPS27A/TRDN/ATP2B3/ASIC5/SCNN1D/ATP2B2/UBA52/FXYD1/ASIC4/ANO9/STOML3/ATP13A

OR4F4/OR4K15/OR52A5/OR5B17/OR5D16/OR5F1/OR5H2/OR5L1/OR6C1/OR6C3/OR6N2/OR7G1/

E2/SOS1/JAK1/IGHV3-30/ROCK2/RCOR1/IGHV1-2/STAT2/IGHV4-39/NFE2L2/VEGFA/HSP90AA1/T

/CRAT/COX19/PEX6/SEC61B/PAM16/COA6/TIMM44/DAO/DDO/SLC25A6/MLYCD/GDAP1/FIS1/F

B/DYNC1H1/CDC42/CLASP1/DIAPH1/ERCC6L/DVL3/FMNL3/PPP2CB/NUP107/XPO1/TUBB6/TUBA

GRP1/UBE2D1/IGHV1-2/IGHV4-39/TRAF6/IGKV3-15/IGKV1D-16/IGHV1-46/TAB3/IGKV3-20/IGKV2

3/MRPS33/MRPS21/MRPS34/MRPS2/MRPS25/MRPL52/MRPL27/MRPL4/AURKAIP1/MRPL24/MF

2/TBL1Y/WNT11/SMAD3/NFATC3/GSK3B/APC/PPARD/PPP2R5E/WNT5B/PSEN1/PRICKLE2/NFATC

TF2/SHC1/ATF1/RPS6KA3/MAPK1/NAB1/STAT3/PIK3CB/EGR1/RIT1/PTPN11/MEF2C/RPS6KA2/PP

NGRF/CACNG6/MYLPF/SLC8A3/MYBPC2/KCNK7/FXYD2/KCNK16/FXYD4/ACTC1/FGF13/KCNE2/K[illegible]

[illegible]B/COX6C/COX6B1/NDUFB1/COX7B2/NDUFA2/NDUFV1/COX8A/NDUFB2/COX5A/COX4I2/COX6A[illegible]

[illegible]MX1B/VIPR2/SOX2/CHRNA4/GCK/CHRNB2/TF/TPPP/ATP1A3/SSTR1/ASCL1/FEV/DDC/SNAP25/K[illegible]

[illegible]DYNC1H1/HLA-DQA1/CTSL/HLA-DPA1/CAPZA2/HLA-DRA/CTSB/RACGAP1/TUBB6/TUBA1B/DNM[illegible]

1/CLASP1/ERCC6L/PPP2CB/NUP107/SMC3/XPO1/SMC1A/TUBB6/TUBA1B/CDK1/PDS5A/WAPL/R

P3/CHRM2/SCARB2/RAB5A/VAMP7/STAM2/COPS8/CD4/LDLR/TGOLN2/WASL/AGTR1/STAM/ARF

GRM1/CALML6/TACR2/ADRB1/CHRM1/CHRM3/GRPR/MYLK2/CACNA1A/ADCY8/PRKCG/ATP2A3

/PRKAA1/APC/RPS6KA3/MAPK1/TRAF6/JAK2/MAP3K1/STAT3/BCL2L11/MTOR/DLG1/CDC42/EGI

/SULT1C2/GAL3ST2/UGT1A4/KCNAB3/AKR7A3/GSTA1/UGT2B7/NAT14/GPX2/HS3ST5/HS6ST3/CY

.6A/CLOCK/DR1/H2AC21/MBIP/H2BC13/MORF4L1/H3C10/H2BC11/ATXN7/H4C14/BRPF3/CREBB

E2L6/SMURF1/ANAPC1/XIAP/CUL4B/CUL5/UBA3/UBE3A/UBE2W/UBE2H/UBE2E3/UBE3C/UBE2I

B7A/RIN2/GAPVD1/ANKRD27/TRAPPC11/RAB18/ALS2/DENND4A/DENND6A/AKT2/RAB21/RAB3

IMM44/SLC25A6/NDUFB8/LDHD/TOMM7/CHCHD5/ATP5MC1/SLC25A4/TIMM13/CHCHD10/OT

21/HSP90AA1/CCP110/HAUS2/PPP2R5E/NDE1/PLK4/SMC2/PDS5B/BUB1B/DYNC1H1/NEK6/CLA

CK/NFYA/ARNT/MED14/TBL1XR1/CDK19/ARNTL/MED21/CPT1A/CREBBP/THRAP3/RGL1/PPARG/

MRAS/ACTN1/GNAI3/PARD6G/MAP3K20/YES1/CTNNA1/OCLN/CASK/CDC42/MYH1/MYL10/MAG

0/TRPA1/CASQ2/ASIC3/MCOLN3/BSND/BEST2/UNC79/CLCNKA/TRPC7/CLCA1/TTYH1/WNK4

C3R/CCR10/MLN/CCL19/SSTR3/GPER1/WNT8B/RAMP1/AGT/ECE2/UCN/PTGDR2/GPR37L1/WNT1

NOD1/TLR6/MAPK14/MAP3K8/MYD88/MAP2K1/MAP3K7/CASP8/ELK1/UBE2V1/USP14/SFTPA2/F

K1A1/YWHAZ/EP300/H2BC21/CDC73/H2BC5/CTNNB1/RYK/TNKS2/DKK2/SOX6/H2AC6/H4C12/H2

4B/TAF1A/BAZ2A/H2BC13/SF3B1/AEBP2/JARID2/H3C10/DEK/H2BC11/H4C14/GTF2H3/ERCC6/H3

iO2/ATP2A2/ST3GAL4/H2BC13/H3C10/TNRC6B/AGO3/H2BC11/MIR302A/RBPJ/H4C14/MAML1/(

53/H2AC6/H4C12/H2AC14/H2AC21/DYNC1H1/NDC1/H2BC13/NUP107/CHMP3/TUBB6/TUBA1B/

Q/RPS6KA3/MAPK1/MAP3K1/PIK3CB/MTOR/RICTOR/RPS6KA2/MAPK14/SOS2/MAP3K5/MAP3K8

CHRNA4/TUBA3E/KCNJ10/CHRNB2/KCNJ6/LIN7B/CHRNB3/ADCY8/MAPT/GRIA2/KCNJ3/KCNJ5/G

)1/MAP3K8/MYD88/PSMD5/MAP2K1/PSMA6/PSMC1/PSMD2/MAP3K7/PTPN4/CASP8/UBE2V1/U

L/FOXO3/SOS1/CCR3/CCL17/ROCK2/GNAI3/CCL28/STAT2/CX3CL1/TIAM2/GSK3B/SHC1/CCL7/CX(

DH1C/GLYATL2/CYP2A6/UGT2B11/SULT1C2/UGT1A4/AKR7A3/GSTA1/GGT6/SLC26A1/ADH6/UGT

:/EDN3/GALR3/UTS2R/GRPR/CCR7/PPY/CXCR5/SSTR1/CXCL13/POMC/SSTR5/HCRT/INSL5/F2/TA(

PBRM1/ITCH/KMT2A/H2AC6/NR4A3/H4C12/H2AC14/AGO2/RYBP/ACTL6A/OCLN/KMT2E/ESR1/R

FAR4/KISS1R/GAL/PROK1/CCL25/PLPPR1/S1PR4/KNG1/C5/DRD2/TACR2/AGTR2/ADRB1/EDN3/R[illegible]

SK3B/SHC1/PRKAA1/RHOQ/RPS6KA3/MAPK1/MAP3K1/PIK3CB/IGF1R/MTOR/EHD2/EGR1/PTPN[illegible]

WRN/BARD1/CDK1/TAF7L/TP73/TAF1/CDKN2A/CHEK1/MDM2/BRPF3/TAF4B/SGK1/PIP4K2C/CC[illegible]
[illegible]P2C9/SULT1A4/UGT2B4/CYP2E1/SULT2A1/SLC22A7/GLYATL3/UGT2B17/CYP3A4/GSTA2/PON1/[illegible]

/GNAI3/CCL28/STAT2/CX3CL1/TIAM2/GSK3B/SHC1/CCL7/CXCL11/MAPK1/GNB1/JAK2/STAT3/PI[illegible]

[illegible]RAD51AP1/ERCC4/WRN/BARD1/H2BC11/XRCC5/CHEK1/H4C14/ABL1/PALB2/BRCC3/CCNA2/AB[illegible]

RCC6L/PPP2CB/NUP107/XPO1/RCC2/UBE2E1/ZWILCH/SGO1/KNTC1/CENPK/CENPQ/NUP98/CEN

/WRN/BARD1/H2BC11/CHEK1/H4C14/ABL1/PALB2/BRCC3/CCNA2/ABRAXAS1/POLH/BRIP1/RAD5

PSMD5/PSMA6/BARD1/PSMC1/CDK1/PSMD2/H2BC11/CDC6/CHEK1/H4C14/YWHAQ/BRCC3/MC

:/LAPTM4A/AP3B1/GNS/ASAH1/PLA2G15/CTSZ/LAMP3/CD68/AP1G1/NAGA/CTSD/SLC17A5/SOR

:TR/GNG7/VIPR2/ADRB1/GNAZ/CALCA/ADCY8/GPR150/GIPR/POMC/PTH/GHRHR/GHRH/GPHA2

;A4/ITGA1/CD44/COL6A2/AGRN/COL6A6/ITGAM/COMP/ITGA9/FGG/SPP1/COL13A1/ITGB2/CDH
/ITGA6/COL4A4/COL4A2/PLOD1/BMP1/P4HA3/LAMB3/COL14A1/DST/COL6A2/COL15A1/P3H2/C

\4/LAMB3/COL6A2/AGRN/COL6A6/GP5/COMP/ITGA9/HMMR/SPP1/ITGB4/LAMB2

A1/P3H2/COL6A6/COL21A1/PCOLCE/TLL1/COL13A1/PCOLCE2/COLGALT1/ADAMTS14/COL6A5

L5A3/ITGA6/LAMC1/BIRC3/ITGA3/COL4A4/VCL/COL4A2/ARHGAP5/FLNB/ITGA4/LAMB1/ITGA1/E

/17/DCN/COL6A1/HSPG2/FBN2/COL5A3/MMP12/COL4A5/LAMC1/COL4A4/COL4A2/MMP19/AD

SMAD7/SKI/WWP1/ATF3/SHC1/MAPK1/TRAF6/KLF11/CDC42/SMURF1/MEF2C/MAPK14/DCP1A/

\2/ARHGAP5/FLNB/ITGA4/LAMB1/ITGA1/EGFR/ACTN4/LAMB3/ROCK1/MYLK3/COL6A2/FLNC/AK

'1/FLNA/EPS15/MAPK1/RAB5A/PIK3CB/IGF1R/KPNA1/CDC42/HLA-DQA1/CTSL/HLA-DPA1/HLA-D
'LD2/DSPP/SVEP1/NTNG1/IGFBP3/THSD4/LAMA4/FGL2/FBN2/SLIT2/MFAP2/CRISPLD1/LAMC1/C
AS/LRRC4C/LIMK1/SRGAP1/NTN4/ROCK2/GNAI3/SRGAP2/SEMA4B/NFATC3/GSK3B/SEMA4C/EP
L22L1/RPL3/RPS18/RPS2/RPL19/UBA52/RPL13A/RPS29/RPL29/RPSA/RPL23A/RPL39/RPL8/RPS9/
RPS19/RPL22L1/RPL3/RPS18/RPS2/RPL19/UBA52/RPL13A/RPS29/RPL29/RPSA/RPL23A/RPL39/RF

S18/RPS2/RPL19/UBA52/RPL13A/RPS29/RPL29/RPSA/RPL23A/RPL39/RPL8/RPS9/RPL9/RPS6/RPL
19/RPL22L1/RPL3/RPS18/RPS2/RPL19/UBA52/RPL13A/RPS29/RPL29/RPSA/RPL23A/RPL39/RPL8,
RPS19/RPL22L1/RPL3/RPS18/RPS2/RPL19/UBA52/RPL13A/RPS29/RPL29/RPSA/RPL23A/RPL39/RF
11/EIF3G/RPS19/RPL22L1/RPL3/RPS18/RPS2/RPL19/UBA52/RPL13A/RPS29/RPL29/RPSA/RPL23A
'RPS11/RPS19/RPL22L1/RPL3/RPS18/RPS2/RPL19/UBA52/RPL13A/RPS29/RPL29/RPSA/RPL23A/R
S19/RPL22L1/RPL3/RPS18/RPS2/RPL19/UBA52/RPL13A/RPS29/RPL29/RPSA/RPL23A/RPL39/RPL8
D300LD/IGKV1-39/CD300A/SIGLEC9/IGKV1-33/LILRA1/SIGLEC1/IGKV1D-39/IGHV2-5/HLA-G/LILR
YLK3/MYH9/DIAPH3/MYH10/KRAS/GNA13/ARHGEF12/PAK2/NRAS/PIK3R1/PDGFB/ITGAM/PPP1
'LP0/RPS27A/RPS20/RPL7A/RPS3A/RPS7/LAMTOR2/RPS11/NPRL2/RPS19/RPL22L1/RPL3/RPS18/
A/RPS3A/RPS7/RPS11/RPS19/RPL22L1/RPL3/RPS18/RPS2/RPL19/UBA52/RPL13A/POLR2L/RPS29
U/RPL27/BOP1/RPL7/RPS10/HSD17B10/RPL6/RPL37/EXOSC5/RPS15A/RPL31/RPS4X/RPS14/RPL

/DAO/LIAS/RPS10/DDO/SLC6A8/HSD17B10/RPL6/RPL37/RPS15A/ACAT1/LIPT2/RPL31/RPS4X/RP
/RPL10A/MTIF3/EIF3F/MRPL51/EEF1D/MRPS17/CHCHD1/MRPL58/RPS8/MRPL2/MRPL43/RPL11

FBR1/SNAI1/CLDN8/PIK3R1/TJP1/FOXC2/PKP1/CTNNB1/SOS1/WNT11/SMAD3/CDH1/GSK3B/SH

RPS20/RPL7A/RPS3A/RPS7/RPS11/RPS19/RPL22L1/RPL3/RPS18/RPS2/RPL19/UBA52/RPL13A/RPS
TGA9/SAV1/ITGB4/ITGB2/CDH1/PPP1R12A/CTNNA1/IGF1R/AJUBA/TEAD3/PRKAR1A/CDH18/TEK

HS/PIK3R1/PLEKHG1/PKN2/DOCK11/VRK2/DOCK5/WIPF1/SRGAP1/DLC1/SOS1/DOCK1/SRGAP2/

HK1/PIK3R5/WASL/PTPN11/RAP1A/PTPN1/MAP2K1/SLA/AFDN/ELK1/ABL1/YWHAQ/PRKCA/TAG
N/CHST6/ADAMTS9/GPC4/B3GLCT/ADAMTS16/ALG11/EXT2/EXT1/SDC4/GALNT3/OGN/ADAMTSI
K/TMEM87A/SRGAP1/DLC1/RHPN2/ROCK2/ARHGAP18/SNAP23/KTN1/VAMP3/ARHGEF17/LMAN
2/ABI2/CYFIP1/OPHN1/RACGAP1/GIT2/WASF1/ARHGAP1/RAB7A/TFRC/ABI1/DOCK10/ARHGAP6/

ID1/PLEC/PARVA/CTNNB1/ACTN1/ITGB4/PARD6G/CDH1/FLNA/ARHGEF6/CTNNA1/PIK3CB/CASK/

3/MAPK1/RAB5A/JAK2/MAP3K1/STAT3/STAM2/MTOR/RICTOR/RALBP1/CDC42/STAM/PTPN11/
2A1/H2BC5/FOXO3/CREB1/MMP7/MED1/GNAI3/SP1/H2AC6/H4C12/ATF2/H2AC14/AGO2/STAG2

WASF2/HSP90AA1/YES1/MAPK1/IGKV3-15/PIK3CB/IGKV1D-16/IGHV1-46/CDC42/IGKV3-20/ABI2/

PNA1/IFNGR1/UBE2L6/EIF4G2/TRIM29/ARIH1/HLA-DQA1/EGR1/PTPN11/NDC1/HLA-DPA1/ADAR

PSG4/TNFRSF10A/NRAS/IGKV1-39/PIK3R1/ANGPT2/IGKV1-33/ITGAM/IGKV1D-39/FCER1G/IGHV
/ATP5MC2/NDUFV1/COX8A/NDUFB2/COX5A/COX6A1/ATP5PO/NDUFB8/UQCRQ/COX4I1/NDUFS

TS5/ADAM17/ADAMTS3/PCSK5/ADAMTS7/SERPINH1/MMP12/ADAM22/LOXL3/SULF2/CPAMD8/

1/ROCK2/DOCK1/MAPK6/MYL3/PPP1R12A/APC/WASF2/MAPK1/CHRM2/ARHGEF6/PIK3CB/EZR/

3A/NDUFB2/COX5A/COX6A1/ATP5PO/NDUFB8/UQCRQ/COX4I1/NDUFS8/NDUFV2/UCP2/NDUFB

F2C/ZFP36/MAP2K1/ELK1/YWHAQ/PLD1/PRKCA/USP6NL/ABI1/YWHAB/BCL2L1/MAPK8/ARPC2/

DLR/PIK3R5/DVL3/SOS2/TP73/MAP3K7/CDKN2A/MDM2/RBL2/ABL1/CCND1/TCF7L2/NFKB1/AK

PS29/RPL29/RPSA/PSMA8/RPL23A/RPL39/RPL8/RPS9/RPL9/PAK5/RPS6/ISL1/RPLP1/RPS12/RPL2

PF1/SRGAP1/DLC1/SRGAP2/FNBP1L/SNAP23/SYDE1/KTN1/FMNL2/VAMP3/ARHGEF6/RALBP1/CD

6/G0S2/ITGAM/DUSP10/SALL4/TNFAIP3/SLC34A2/MXD1/SPP1/COL13A1/S100G/RASGRP1/TNFS

A2/ATP6V0B/ATP5MC2/NDUFV1/COX8A/NDUFB2/COX5A/COX4I2/COX6A1/ATP5PO/NDUFB8/UQ

L/ANXA2/PTPN11/MEF2C/MAPK14/MAP2K1/ELK1/CDKN2A/PPARG/BMP2/CCND1/PRKCA/RPS6K

JFB8/UQCRQ/COX4I1/NDUFS8/ECSIT/NDUFV2/NDUFB9/COQ10A/COX5B/UQCR11/NDUFB11/ND

5/CDC42/IGKV3-20/ABI2/IGKV2-30/WASL/CYFIP1/WASF1/WIPF2/IGHV3-11/ABL1/IGHV3-53/FGR/

/DHD1/TRIM5/ELK4/GDF3/PITX2/ZNF462/JAK2/CCDC6/NODAL/RARG/BCORL1/PRKAR1A/CUL4B/

C/COX6B1/ATP5F1E/NDUFB1/COX14/NDUFA2/ATP5MC2/NDUFV1/COX8A/NDUFB2/COX5A/COX

N11/MEF2C/RPS6KA2/PPP2CB/NOD1/TLR6/MAPK14/CTSB/MAP3K8/MYD88/MAP2K1/DNM3/M/

NFATC2/MAPK14/SOS2/MYD88/MAP2K1/EGR3/MAP3K7/CASP8/ELK1/SMAD2/IFNAR1/CREBBP/Y

;ALNT2/GALNT7/GALNT13/B3GNT2/GALNT4/ST3GAL4/GALNT3/GALNT14/ADAMTSL1/ADAMTS1[illegible]

R1/KIR2DL4/FAS/NFATC2/PIK3R5/PTPN11/KLRC1/NCR1/TNFRSF10B/SOS2/MAP2K1/SH3BP2/IFN[illegible]

/NDUFA2/ATP5MC2/NDUFV1/COX8A/NDUFB2/COX5A/SNCA/COX4I2/COX6A1/ATP5PO/NDUFB8

2L11/VIM/STK24/FAS/LMNB1/DCC/PDCD6IP/XIAP/CHMP3/TNFRSF10B/PSMD5/PSMA6/PSMC1/[illegible]

ATC3/IGHV4-39/SHC1/MAPK1/TRAF6/IGKV3-15/MAP3K1/PIK3CB/IGKV1D-16/IGHV1-46/TAB3/IGK

DGFB/NF1/RAP1B/TAB2/DUSP10/DUSP7/RASGRP4/MRAS/SOS1/HSPA1B/RASA2/RASGRP1/MAP4

S/CDKN2A/MDM2/H4C14/NUP58/NUP205/PGR/CREBBP/PPARG/UHRF2/PIAS1/NCOA1/SATB1/S

C21/STAM2/H2BC13/MAT2B/USP24/PSMD5/PSMA6/PSMC1/RNF128/PSMD2/H2BC11/MAP3K7/

F10B/PSMD5/PSMA6/PSMC1/TP73/PSMD2/APPL1/CASP8/CDKN2A/CFLAR/YWHAQ/APAF1/SATB

AB5A/TRAF6/CXCR1/IGF1R/STAM2/EHD2/CDC42/SMURF1/PDCD6IP/LDLR/STAM/ARF6/PIKFYVE/

/IFNAR2/NECTIN4/IFNAR1/APAF1/TBK1/CCND1/HSPA8/NFKB1/BCL2L1/MAPK8/EIF2S1

NG8/VAMP2/PRKCB/SYT10/NRGN/GNB3/RAB3A/KCNG2/CACNA1B/LRRTM2/KCNAB3/GRIN2C/TU

I4C14/NUP58/NUP205/LPIN2/MASTL/PRKCA/H3C4/NUP98/NCAPG2/TPR/RAB1A/RANBP2/SET/G

/1/COX8A/NDUFB2/COX5A/SNCA/COX4I2/COX6A1/ATP5PO/CACNA1C/CHP2/NDUFB8/UQCRQ/C

/GRHL2/AEBP2/NAA15/JARID2/CDYL/ATP8B2/NKX2-1/RTF1/BPTF/BMPR1A/SMAD2/PARP8/TAF4

HRH3/GRIA2/SSTR1/PRLR/GIPR/SSTR5/GHRHR/GRIK5/CNR2/P2RX6/PRSS3/GRIN1/F2/P2RX1/HTF

M1/NKD1/MAP3K7/CHD8/DKK4/LRP6/CCND1/PRKCA/CAMK2A/TCF7L2/DAAM2/SENP2/MAPK8/

C1/OR6C3/OR6N2/OR7G1/OR8B8/OR8H1/OR10AC1/OR2AE1/OR51I1/OR11H4/OR52D1/OR52K1

GFBP7/CCNE2/UBN1/RPS6KA2/H2BC13/MAPK14/MAP3K5/H3C10/CCNE1/TNRC6B/AGO3/H2BC1

OX6B1/ATP5F1E/PPARGC1A/NDUFB1/COX7B2/NDUFA2/ATP5MC2/NDUFV1/COX8A/NDUFB2/CC

MC1A/H3C10/TNRC6B/AGO3/POU2F1/H2BC11/H4C14/PGR/CREBBP/AGO4/CCND1/H3C4/NCOA1

N1/CHRNA4/CACNA2D2/TUBA3E/CACNB2/KCNJ10/CHRNB2/KCNJ6/LIN7B/SLC32A1/CHRNB3/CAC

PIK3R5/STAM/PTPN11/SOS2/CLCF1/IL13RA1/IL7R/SPRY3/IFNAR2/IFNAR1/CREBBP/IL15RA/SOCS3

MAP3K5/MAP2K1/TP73/ABL1/YWHAQ/ZNF274/MAP3K3/CAMK2A/NTF4/NFKB1/AKT2/YWHAB/I

/PLEKHG5/ARHGEF10/MAP3K7/CASP8/NGEF/CFLAR/TAX1BP1/CYLD/NFKB1/MAPK8/RHOA/USP4
A5/ATP8A1/ANO7/WNK2/UNC80/FXYD2/FXYD4/TRPA1/ATP6V1C2/CASQ2/ASIC3/ATP1A2/ATP6V

/OR8B8/OR8H1/OR2AE1/OR51I1/OR11H4/OR52D1/OR52K1/OR5K1/OR1E2/OR10G3/OR1I1/OR4

LR9/JAK2/IGKV3-15/ARID4A/IFNGR1/IGKV1D-16/IGHV1-46/ARID4B/IGKV3-20/IGKV2-30

RPS27A/PEX16/DECR2/ECI2/NDUFB8/LDHD/VAMP2/UBA52/TYSND1/TOMM7/PXMP2/DHRS4/CH

A1B/DAAM1/RCC2/ZWILCH/TUBA1C/SGO1/KNTC1/CENPK/CENPQ/NUP98/CENPL/ZW10/RHOA/A

2-30/BCL10/PSMD5/PSMA6/PSMC1/MALT1/PSMD2/MAP3K7/UBE2V1/IGHV3-11/IGHV3-53

RPL34/GADD45GIP1/MRPL57/MRPL23/MRPL38/MRPL54/MRPL55/MRPS24/MRPS26/MRPL41/M

2/DVL3/PPP2CB/DAAM1/TBL1XR1/NKD1/MAP3K7/CHD8/SMAD2/CREBBP/DKK4/LRP6/CCND1/P

CNK12/CACNA2D2/CASQ2/CACNB2/ATP1A2/MYH6/TNNT3/FXYD7/KCNK10/KCNJ11/TNNT2/ATP1

1/NDUFB8/INS/UQCRQ/COX4I1/NDUFS8/NDUFV2/NDUFB9/COX5B/PIK3R2/UQCR11/NDUFB11/

3/HLA-DQB2/CAPZB/KIF11/TUBA1C/RAB7A/KIF4B/KIF5B/HLA-DOA/AP1G1/CTSD/HLA-DQB1/KIF4

.CC2/ZWILCH/TUBA1C/SGO1/KNTC1/CENPK/CENPQ/NUP98/CENPL/ZW10/AHCTF1

'P2A7/CYP2C9/GSTT2/CHST8/SULT1A2/SULT1A4/UGT2B4/CYP2E1/EPHX2/HS3ST6/NAT8L/CYP1A

SP1/ERCC6L/PPP2CB/NUP107/SMC3/TUBB/XPO1/SMC1A/TUBB6/TUBA1B/CDK1/PDS5A/WAPL/(

10B/GNG4/P2RY11/RLN2/GNRH2/TACR1/CCL21/CALCB/SSTR2/WNT6/GPR18/KEL/MLNR/CXCL3/

/H3C10/TBL1XR1/H2BC11/ELK1/H4C14/NUP58/TUBA1C/NUP205/UBAP1/CHMP4C

CL11/MAPK1/CXCR1/GNB1/JAK2/STAT3/PIK3CB/CX3CR1/CDC42/PIK3R5/CCL11/WASL/ADCY3
T2B7/AKR7L/FDX2/ACSS1/ACSM2B/TPST2/CYP2A7/CES3/CYP2C9/GSTT2/AOC1/SULT1A2/SULT1A

NF2/PCGF5/CBFB/NFATC2/CTSL/PTPN11/ARID1B/H2BC13/PSMD5/PSMA6/PSMC1/H3C10/ARID2

GR/CHRM1/GALR3/UTS2R/CHRM3/GRPR/CCR7/PPY/HRH3/CXCR5/SSTR1/CXCL13/POMC/SSTR5/

11/ARF6/RPS6KA2/MAPK14/SOS2/MAP3K5/MAP3K8/PTPN1/MAP3K13/MAP2K1/MAP3K7/ELK1/

NA2/TPX2/AKT2/BRIP1/RAD50/SUPT16H/PRKAB2/PRMT5/TAF3/RFC3/MRE11/ATM/JMY/PML/U

RAXAS1/POLH/MAPK8/BRIP1/RAD50/BAZ1B/RFC3/EYA3/MRE11/SMARCA5/ATM/UBE2V2

M2/ABRAXAS1/YWHAB/BRIP1/RAD50/MCM6/RFC3/MRE11/ATM/PSME4/UBE2V2/PSMD7/CCNB

.T1/GM2A/SUMF1/AP3M2/FUCA1/ENTPD4/LAPTM5/CTSS/IDS/LGMN/GLB1/LAPTM4B/PSAP/ACF

:OL6A6/COL21A1/MMP3/PCOLCE/PLEC/MMP7/TLL1/ITGB4/COL13A1/LOXL4/PCOLCE2/COLGALT

:GFR/ACTN4/LAMB3/ROCK1/MYLK3/COL6A2/FLNC/AKT3/PAK2/COL6A6/FLT1/PIK3R1/PDGFB/RA

)AM10/LAMB1/ADAMTS4/BMP1/HTRA1/CD44/LAMB3/COL14A1/KLK7/COL6A2/CAST/COL15A1/(

/MAP2K1/CDK1/PRKAR2A/MAP3K7/RBL2/SMAD2/CREBBP/TGFB1I1/CCND1/PIAS1/NEDD9

(T3/PAK2/FLT1/PIK3R1/PDGFB/RAP1B/COMP/PPP1CB/PARVA/CTNNB1/ITGA9/PTEN/SOS1/ACTN

:ILP2/FBLN2/CCN2/LAMB1/LAMB3/ABI3BP/CRIM1/ZP4/TSKU/AGRN/PXDNL/EFEMP2/KCP/SNED1

/RPL9/RPS6/RPLP1/RPS12/RPL28/RPS5/RPL27A/RPS26/RPL36/RPL32/RPS24/RPL35/RPS27/RPL34

PL8/RPS9/RPL9/RPS6/RPLP1/RPS12/RPL28/RPS5/RPL27A/RPS26/RPL36/RPL32/RPS24/RPL35/RPS

.P1/RPS12/RPL28/RPS5/RPL27A/RPS26/RPL36/RPL32/RPS24/RPL35/RPS27/RPL34/RPL37A/RPS2

/RPS9/RPL9/RPS6/RPLP1/RPS12/RPL28/RPS5/RPL27A/RPS26/RPL36/RPL32/RPS24/RPL35/RPS27/

PL8/RPS9/RPL9/RPS6/RPLP1/RPS12/RPL28/RPS5/RPL27A/RPS26/RPL36/RPL32/RPS24/RPL35/RPS

/RPL39/EIF4EBP1/RPL8/RPS9/RPL9/RPS6/RPLP1/RPS12/RPL28/RPS5/RPL27A/RPS26/RPL36/RPL3

RPL39/RPL8/RPS9/RPL9/RPS6/RPLP1/RPS12/RPL28/RPS5/RPL27A/RPS26/RPL36/RPL32/RPS24/RP

3/RPS9/RPL9/RPS6/RPLP1/RPS12/RPL28/RPS5/RPL27A/RPS26/RPL36/RPL32/RPS24/RPL35/RPS27

A2/IGLV6-57/NCR3LG1/PILRA/LILRA5/KLRD1/LILRB5/IGHV3-30/CD300LB/CD300LF/SH2D1B/ITGB

CB/LIMK1/ITGA9/MRAS/SOS1/ACTN1/ROCK2/ITGB4/DOCK1/ITGB2/PPP1R12A/TIAM2/APC/TMSE

/LAMTOR4/RPS2/RPL19/UBA52/RPL13A/RPS29/RPL29/RPSA/RPL23A/RPL39/RPL8/RPS9/RPL9/RP

/RPL29/RPSA/RPL23A/RPL39/RPL8/RPS9/RPL9/POLR2I/RPS6/RPLP1/RPS12/POLR2J/RPL28/RPS5/

17/RPS13/RPL12/RPL36A/RPS17/RPL24/RPL14/RPLP0/RPS27A/RPS20/RPL7A/RPS3A/RPS7/RPS1

S14/RPL17/RPS13/CGA/RPL12/PHGDH/RPL36A/RPS17/RPL24/RPL14/RPLP0/RPS27A/RPS20/RPL
/MRPL53/SEC61B/RPS3/RPL23/FAU/RPL27/MRPS33/RPL7/MRPS21/RPS10/RPL6/RPL37/RPS15A/

S29/RPL29/RPSA/PSMA8/RPL23A/RPL39/RPL8/RPS9/RPL9/RPS6/ISL1/RPLP1/RPS12/RPL28/RPS5/

LEMD3/CYBB/SNAP23/KTN1/TIAM2/GARRE1/VAMP3/WASF2/SWAP70/ARHGEF6/RALBP1/CDC42

I1/DIAPH1/OPHN1/FMNL3/RACGAP1/STARD8/PLEKHG5/DAAM1/FAF2/ARHGEF10/NGEF/ARHGA

ARF6/MEF2C/RPS6KA2/SYNJ1/SH3KBP1/MAPK14/SOS2/RAP1A/MAP2K1/USP8/ELK1/ABL1/MAP3
2/SHC1/NCOA3/RAD21/HSP90AA1/MAPK1/CHD1/GNB1/IGF1R/SPHK1/ESR1/GNAT3/CBFB/UHMK

/IGKV2-30/WASL/CYFIP1/WASF1/WIPF2/IGHV3-11/ABL1/FCGR1A/IGHV3-53/PLD1/FGR/ABI1/MY

R/IFIT5/TRIM22/OAS1/NUP107/HLA-DRA/PTPN1/EIF4G1/ABCE1/PTAFR/HLA-DQB2/MX1/UBE2E1

2-5/IGLV6-57/SOS1/CEACAM8/PSG5/IGHV3-30/MERTK/ITGB2/THBD/IGHV1-2/SLC7A7/IGHV4-39/
58/ECSIT/NDUFV2/UCP2/NDUFB9/COQ10A/COX5B/ATP5MC1/UQCR11/NDUFB11/NDUFA3/SLC25

/MMP19/ADAM10/CPN2/SERPINB2/PLOD1/PCSK6/ADAMTS4/BMP1/HTRA1/P4HA3/ADAMTSL4/

9/COX5B/ATP5MC1/SLC25A4/UQCR11/NDUFA3/SLC25A27/NDUFB7/NDUFA7/NDUFS6/ATP5ME/

8/RPS5/RPL27A/RPS26/RPL36/RPL32/RPS24/RPL35/RPS27/RPL34/RPL37A/PSMB11/RPS28/NTN1

C42/STEAP3/CDC42BPA/WASL/OPHN1/FMNL3/RACGAP1/GIT2/GOLGA8R/STARD8/DAAM1/WIP

CRQ/COX4I1/NDUFS8/NDUFV2/NDUFB9/COX5B/ATP5MC1/UQCR11/NDUFA3/NDUFB7/NDUFA7

/GRHL2/AEBP2/JARID2/ATP8B2/ACVR2A/BMPR1A/SMAD2/PARP8/KLF5/CCND1/PIAS1/TEAD2/SC

(6A1/MPC2/ATP5PO/GSTZ1/NDUFB8/D2HGDH/UQCRQ/COX4I1/PDK4/NDUFS8/ECSIT/NDUFV2/U

;/UQCRQ/COX4I1/NDUFS8/NDUFV2/UCHL1/NDUFB9/COX5B/ATP5MC1/SLC25A4/UQCR11/NDUF

TP73/PSMD2/APPL1/CASP8/CDKN2A/CFLAR/CHMP4C/YWHAQ/APAF1/SATB1/IL18/BMF/AKT2/Y\

V3-20/IGKV2-30/NFATC2/BCL10/PSMD5/PSMA6/PSMC1/MALT1/PSMD2/MAP3K7/UBE2V1/IGH

4K3/ATF2/CACNB4/RAPGEF2/FLNA/NFATC3/MAP3K20/CACNA2D1/ELK4/RPS6KA3/MAPK1/TRAF

MC6/NUP98/ZBED1/SENP2/SUMO3/TPR/THRB/SIN3A/UBA2/RANBP2/HNRNPK/MRTFA/TDG/P

/ATXN7/USP8/MDM2/SMAD2/USP14/USP33/SIAH2/CCNA2/CYLD/USP4/USP47/USP48/USP10/P

1/BMF/AKT2/YWHAB/SPTAN1/BCL2L1/MAPK8/STK26/TJP2/DFFA/PPP3R1/TFDP1/LY96/PSME4/

/SH3KBP1/CHMP1B/CHMP3/EHD4/GIT2/RAB11FIP2/CLTCL1/DNM3/PIP5K1A/ARFGAP3/USP8/M

JBA8/GABRB3/TUBAL3/KCNC1/PRKAR1B/HCN4/SLITRK2/GLRA2/GABRA1/BEGAIN/SLC18A3/HCN

OX4I1/PLCB4/APBB1/NDUFS8/NDUFV2/GRIN2C/NDUFB9/COX5B/ATP5MC1/UQCR11/NDUFA3/C

./OR6J1/OR5K1/OR1E2/OR10G3/OR1I1/OR4K14/OR7A17/OR4E2/OR10J3/OR52J3/OR2T33/OR2N

1/UBE2E1/CDKN2A/MDM2/H4C14/AGO4/CCNA2/H3C4/NFKB1/MAPK8/RAD50/TFDP1/MRE11/A

)X5A/COX4I2/COX6A1/ATP5PO/NDUFB8/UQCRQ/COX4I1/PLCB4/POLR2L/DNAH2/NDUFS8/NDUF

CNA1A/ADCY8/GLS2/MAPT/PPFIA3/GRIA2/KCNJ3/SNAP25/CPLX1/KCNJ5/GRIK5/CACNG2/PRKCG/

/0E2/FXYD7/MCOLN3/BSND/ATP1A3/BEST2/UNC79/ATP2A3/CLCNKA/TRPC7/CLCA1/CAMK2B/TT

K14/OR7A17/OR4E2/OR10J3/OR52J3/OR2T33/OR2M5/OR2T2/OR2V2/OR6C2/OR6C75/OR7G2/C

CHD5/ATP5MC1/SLC25A4/SLC27A2/NOS2/TIMM13/CHCHD10/HAO1/EPHX2/PIPOX/OTC/AGXT

RKCA/FBXW11/CAMK2A/TCF7L2/DAAM2/SENP2/MAPK8/RHOA/PRKACB/PORCN/PPP3R1

LA3/KCNK3/TCAP/KCNH2/TRIM72/NPPC/ATP2A3/SLC8A2/CAMK2B/CACNG7/MYL7

NDUFA3/NDUFA13/NDUFB7/NDUFA7/NDUFS6/NDUFA11/NDUFS7/CYP2E1/PKLR/COX6A2/MLXIP

\2/GPX3/SULT2A1/HS3ST4/CYP2W1/UGT2B17/GSTA3/CYP3A4/CYP46A1/GSTA2/CHST5/GAL3ST3

:EP135/RCC2/ZWILCH/TUBA1C/NME7/MZT1/AKAP9/SGO1/KNTC1/CENPK/CENPQ/EML4/NUP98/

IHH/QRFPR/ADRA2C/CXCL2/SAA1/CORT/CCK/GRM4/FFAR4/VIPR1/KISS1R/GAL/WNT3A/PROK1/[illegible]

4/UGT2B4/CYP2E1/PODXL2/CYP1A2/SULT2A1/DPEP1/CYP2W1/POMC/CHAC1/GLYATL3/UGT2B1

2/NFE2/KMT2C/TNRC6B/AGO3/TP73/PSMD2/H2BC11/ARID1A/H4C14/SMARCC2/ABL1/CREBBP/S

/STXBP4/SGK1/MAP3K3/STXBP3/SOCS3/PRKCA/KIF5B/EIF4E/RPS6KB1/AKT2/MAPK8/INPP4A/ENI

.P1B/COMP/PPP1CB/PARVA/CTNNB1/ITGA9/PTEN/SOS1/ACTN1/SPP1/ROCK2/ITGB4/DOCK1/LAN

COL6A6/MMP10/ADAMTS9/MMP3/MMP7/TLL1/ADAMTS16/SPP1/COL13A1/A2M/CDH1

1/SPP1/ROCK2/ITGB4/DOCK1/LAMB2/BIRC2/PPP1R12A/FLNA/CAPN2/GSK3B/SHC1/VEGFA/MAF

L/CCN1/FBLN1/COMP/PCOLCE/VWA2/EMILIN1/MFGE8/FGG/NTN4/SPP1/LAMB2/CCN3/CILP

4/RPL37A/RPS28/RPL18/RPL21/RPL26/RPL38/RPL13/RPS21/RPL18A/RPLP2/RPS15/RPL3L/RPL10L

527/RPL34/RPL37A/RPS28/EEF1A2/RPL18/RPL21/RPL26/RPL38/RPL13/RPS21/RPL18A/RPLP2/RPS

/RPL34/RPL37A/RPS28/ASNS/RPL18/RPL21/RPL26/RPL38/RPL13/RPS21/RPL18A/RPLP2/RPS15/R

527/RPL34/RPL37A/RPS28/RPL18/RPL21/RPL26/RPL38/RPL13/RPS21/RPL18A/RPLP2/RPS15/RPS4

32/RPS24/RPL35/RPS27/RPL34/RPL37A/RPS28/RPL18/RPL21/RPL26/RPL38/RPL13/RPS21/RPL18A

'L35/RPS27/RPL34/RPL37A/RPS28/RPL18/RPL21/RPL26/RPL38/RPL13/RPS21/RPL18A/RPLP2/RPS

7/RPL34/RPL37A/RPS28/RPL18/RPL21/RPL26/RPL38/RPL13/RPS21/RPL18A/RPLP2/RPS15/RPS4Y

2/CD226/CDH1/LILRB3/IGHV1-2/LILRB2/CD33/IGHV4-39/CXADR/IGKV3-15/IGKV1D-16/CD1A/KIF

34XP8/WASF2/DIAPH2/MAPK1/CHRM2/ARHGEF6/PIK3CB/EZR/CDC42/MYL10/ABI2/PIK3R5/DIAP

'S6/RPLP1/RPS12/RPL28/RPS5/RPL27A/RPS26/RPL36/RPL32/RPS24/RPL35/RPS27/RPL34/RPL37A

/RPL27A/RPS26/RPL36/RPL32/RPS24/RPL35/RPS27/RPL34/RPL37A/RPS28/RPL18/RPL21/RPL26/

1/RPS19/RPL22L1/PELP1/RPL3/RPS18/RPS2/RPL19/UBA52/RPL13A/NOL12/RPP21/PWP2/RPS29/

7A/RPS3A/RPS7/PCBD1/GSTZ1/RPS11/AGMAT/RPS19/IDO2/RPL22L1/RPL3/RPS18/RPS2/RPL19/U
/MRPS34/RPL31/MRPS2/RPS4X/MRPS25/RPS14/RPL17/RPS13/RPL12/RPL36A/RPS17/EEF1B2/RP

/RPL27A/RPS26/RPL36/RPL32/RPS24/RPL35/RPS27/RPL34/RPL37A/PSMB11/RPS28/RPL18/RPL2

2/ABI2/CDC42BPA/WASL/CYFIP1/OPHN1/RACGAP1/SOS2/GIT2/WASF1/WIPF2/ARHGEF10/NGEF/

P1/TFRC/PLD1/FARP1/DOCK2/MYO9B/ARHGAP6/RHOA/TJP2/STARD13/JUP/MCF2/ARHGEF25

/MX2/IFNAR2/IFITM1/NUP58/FCGR1A/NUP205/IFNAR1/B2M/SOCS3/PIAS1/MID1/CAMK2A/EIF4

/SHC1/SDC4/CXADR/YES1/CD58/IGKV3-15/PROS1/PIK3CB/IGKV1D-16/IGHV1-46/IGKV3-20/IGKV
5A27/NDUFA13/ETFB/NDUFB7/NDUFA7/NDUFS6/NDUFA11/ATP5ME/NDUFS7/ATP5F1D/PM20D

PAMR1/ADAMTS6/TGM4/CTSO/P3H2/SERPINB10/MMP10/SERPINB8/PAPPA2/CST2/ADAMTS9/I

1/RPL18/RPL21/RPL26/RPL38/RPL13/RPS21/RPL18A/RPLP2/RPS15/PAK3/RPS4Y2/RPL3L/SLIT1/R

'/NDUFS6/NDUFA11/ATP5ME/NDUFS7/ATP6V1C2/ATP6V0E2/ATP5F1D/COX6A2/ATP4A

JCP2/NDUFB9/COQ10A/COX5B/ATP5MC1/UQCR11/NDUFB11/NDUFA3/SLC25A27/NDUFA13/ETF

WHAB/SPTAN1/BCL2L1/MAPK8/STK26/TJP2/DFFA/PPP3R1/TFDP1/CASP4/LY96/PSME4/H1-0/PSM

V3-11/IGHV3-53/FBXW11/NFKB1/ITPR1/MAPK8/AHCYL1/GAB2/PPP3R1/TEC/IGKV3D-20/PSME4/

5/MAP3K1/DUSP1/CDC42/FAS/CACNA2D4/MEF2C/CACNG4/MAPK14/SOS2/RAP1A/MAP3K5/MA

1/GNG4/KCNF1/TUBA3C/STXBP1/LRFN1/GABRG2/KCNG1/SYT7/ABAT/KCNV2/SLC1A6/TSPOAP1/

CACNA1S/NDUFB7/NDUFA7/NDUFS6/NDUFS7/CALML6/ATP5F1D/MAPT/COX6A2/ATP2A3/GRIN1/

M5/OR2T2/OR2V2/OR6C2/OR6C75/OR7G2/OR10A6/OR10C1/OR14C36/OR2M3/OR2W1/OR51A2

ATM/ETS2/H1-0/UBC/POT1/CDC23/AGO1/PHC2/TERF1/BMI1/IFNB1/CEBPB/RBBP4

V2/CREB3L1/NDUFB9/POLR2I/COX5B/POLR2J/ATP5MC1/SLC25A4/UQCR11/NDUFA3/NDUFB7/N

)R10A6/OR10C1/OR14C36/OR2M3/OR2W1/OR51A2/OR52M1/OR5V1/OR8G1/OR2A5/OR10A3/[illegible]

/CENPL/ZW10/AHCTF1/NCAPG/HAUS6/RANBP2/CEP70/CEP192/CENPN/CCNB1/MAPRE1

:CL25/PLPPR1/SCTR/S1PR4/KNG1/GNG7/GRM1/C5/VIPR2/DRD2/TACR2/AGTR2/ADRB1/TAS1R2/

MB2/BIRC2/PPP1R12A/FLNA/CAPN2/GSK3B/SHC1/VEGFA/MAPK1/PIK3CB/IGF1R/CDC42/ILK/MYI

R2DL4/IGHV1-46/LAIR1/IGKV3-20/C3/IGKV2-30/KLRC1/NCR1/SIGLEC5/OSCAR/CD200R1/IFITM1/I

A/RPS28/ASNS/RPL18/RPL21/RPL26/RPL38/RPL13/RPS21/RPL18A/RPLP2/RPS15/ATP6V1C2/ATP6

MRM3/RPL29/NOC4L/RPSA/RPL23A/RPL39/RPL8/RPS9/RPL9/EXOSC4/RPS6/RPLP1/RPS12/WDR

JBA52/RPL13A/RIMKLA/RPS29/RPL29/SLC45A2/RPSA/PXMP2/PSMA8/RPL23A/RPL39/RPL8/RPS[illegible]
L24/RPL14/RPLP0/RPS27A/RPS20/RPL7A/RPS3A/RPS7/MRPL52/RPS11/MRPL27/EIF3G/RPS19/R[illegible]

L/RPL26/RPL38/RPL13/RPS21/RPL18A/RPLP2/RPS15/RPS4Y2/RPL3L/SLIT1/RPL10L/MSI1

/ARHGAP1/RAB7A/ARHGAP12/TFRC/PLD1/FARP1/ALS2/ABI1/DOCK10/DOCK2/MYO9B

MMP3/SERPINB5/HYAL4/MMP7/CTSC/TLL1/ADAMTS16/A2M/HTRA3/ADAM23/LOXL4/SERPING1

FB/NDUFB7/NDUFA7/NDUFS6/LDHC/NDUFA11/ATP5ME/NDUFS7/ATP5F1D/PM20D1

/KCNK7/KCNK16/GAD1/HTR3E/GRIA1/LRRC4B/SLITRK1/GNG7/GRM1/KCNN2/CHRNA2/TSPAN7/S

2/OR52M1/OR5K4/OR5V1/OR8G1/OR2A5/OR10A3/OR51B4/CNGA4/OR2A14/OR10H2/OR10W1/

OR51B4/CNGA4/OR2A14/OR10H2/OR10A5/OR5AU1/OR51B2/OR14I1/OR6B2/OR2AT4/OR2F1/C

/EDN3/RGR/CHRM1/CALCA/GALR3/UTS2R/CHRM3/GRPR/CCR7/PPY/HRH3/CXCR5/SSTR1/WNT4/

18/RPL28/RPS5/RPL27A/RPS26/RPL36/RPL32/RPS24/RPL35/RPS27/RPL34/RPL37A/MRM1/RPS2[illegible]

)/RPL9/ARG1/BCAT2/RPS6/RPLP1/RPS12/RPL28/RPS5/RPL27A/HGD/RPS26/RPL36/RPL32/SDSL/[illegible]
PL22L1/MRPL4/RPL3/AURKAIP1/RPS18/RPS2/RPL19/UBA52/RPL13A/RPS29/RPL29/MRPL24/RPS[illegible]

SYN1/CHRNA4/CACNA2D2/TUBA3E/CACNB2/KCNJ10/CHRNB2/KCNJ6/KCNV1/LIN7B/SLC32A1/CH

/OR2V1/OR10A5/OR5AU1/OR4F21/OR51B2/OR14I1/OR6B2/OR2AT4/OR2F1/OR2T34/OR1E3/OR

OR2T34/OR10T2/OR10S1/OR5L2/OR10H3/OR13C3/OR13C4/OR1D2/OR5M1/OR6B1/OR10K2/OR2

/FZD9/GIPR/CXCL13/POMC/PTH/SSTR5/TAS2R8/GHRHR/HCRT/INSL5/CNR2/F2/TAC3/FFAR2/HTR

3/RPL18/RPL21/RPL26/RPL38/RPL13/RPS21/RPL18A/RPLP2/RPS15/RPS4Y2/RPL3L/RPL10L

RPS24/RPL35/RPS27/RPL34/RPL37A/PSMB11/RPS28/HOGA1/TSTD1/CKMT1A/ARG2/ASNS/RPL18
A/MRPL34/RPL23A/RPL39/EIF4EBP1/GADD45GIP1/RPL8/RPS9/RPL9/RPS6/RPLP1/RPS12/RPL28/

RNB3/KCNK10/KCNH3/KCNJ11/CACNA1A/ADCY8/GLS2/ABCC8/HCN2/MAPT/KCNK3/PPFIA3/GRI

10T2/OR10S1/OR5L2/OR10H3/OR13C3/OR13C4/OR1D2/OR4A8/OR5M1/OR6B1/OR10D3/OR10I

2T12/OR4B1/OR4C12/OR4F15/OR4F3/OR51L1/OR5D18/OR6K2/OR6Q1/OR6Y1/OR7G3/OR8H2/C

3/RPL21/RPL26/TST/QDPR/MPST/SLC25A10/AMDHD1/PNMT/CRYM/RPL38/RPL13/RPS21/PSAT1/
RPS5/RPL27A/MRPL57/RPS26/MRPL23/MRPL38/RPL36/RPL32/RPS24/RPL35/RPS27/RPL34/RPL3

A2/KCNC2/KCNJ3/KCNH6/DLGAP1/DLGAP3/SNAP25/CPLX1/KCNH2/KCNJ5/GRIK5/KCNA4/CACNG

[illegible]2/OR2J1/OR2T12/OR4B1/OR4C12/OR4C5/OR4F15/OR4F3/OR51L1/OR5D18/OR5K3/OR6K2/OR[illegible]

R8J1/OR9K2/PDE1C/OR13D1/OR2A12/OR11G2/OR6B3/OR4D11/OR6V1/OR10G8/OR2T29/OR1

/RPL18A/RPLP2/CKMT1B/TPH1/BHMT/CDO1/SARDH/RPS15/GPT2/GPT/PRODH/HPD/HAO1/PIPC
37A/RPS28/EEF1A2/MRPL54/MRPL55/RPL18/RPL21/RPL26/MRPS24/MRPS26/RPL38/RPL13/RPS2

5Q1/OR6Y1/OR7G3/OR8H2/OR8J1/OR8J2/OR9K2/OR13D1/OR2A12/OR11G2/OR14K1/OR6B3/OI

)A7/OR10J5/OR10Z1/OR2M7/OR4A5/OR51A4/OR51G2/OR5B3/OR8K1/OR10A4/OR5B12/OR56B

DX/RPS4Y2/GATM/NAT8L/TAT/GLS2/AGXT2/CBS/RPL3L/CKB/DDC/OTC/GAMT/RPL10L/AGXT/CPS
21/RPL18A/MRPL41/RPLP2/MRPL12/RPS15/EEF1G/RPS4Y2/SSR4/SEC11C/RPL3L/RPL10L

R2T35/OR4D11/OR6V1/OR10G8/OR11H2/OR2T29/OR10A7/OR10J5/OR10Z1/OR2M7/OR4A5/OR

1/OR2H2/OR1L6/OR7D4/OR8A1/OR1A2/OR2J3/OR12D3/OR13F1/OR13G1/OR52E6/OR5AP2/OR

[illegible]4K3/OR51A4/OR51G2/OR5B3/OR6P1/OR8K1/OR10A4/OR5B12/OR56B1/OR2H2/OR1L6/OR7D4/

14A16/OR4D10/OR4F17/OR4F6/OR9G4/OR6C4/GNAL/OR8D4/OR10A2/OR5P3/OR4D6/OR2S2/C

'OR8A1/OR1A2/OR2J3/OR12D3/OR13F1/OR13G1/OR52E6/OR5AP2

OR6K6/OR2G2/OR2M2/OR1C1/OR1D5/OR2F2/OR2T8/OR2B11/OR7A5
