# Supplementary material for: Identification of fibronectin type III domain containing 3B as a potential prognostic and therapeutic target for pancreatic cancer: a preliminary analysis
Source: Eur J Med Res. 2024 Apr 5;29:221. doi: 10.1186/s40001-024-01823-6 (PMC10996089; doi:10.1186/s40001-024-01823-6)
Supplement: Supplementary file 6 — Additional file 6: Table S5. The prognostic value of FNDC3B (Disease Specific Survival) in various PAAD subgroups. [file 40001_2024_1823_MOESM6_ESM.docx]

**Table S5.** The prognostic value of FNDC3B (Disease Specific Survival) in various PAAD subgroups

| **Characteristics** | **N (%)** | **HR (95% CI)** | **P value** |
| --- | --- | --- | --- |
| T stage |  |  |  |
| T1&T2 | 31 | 1.032(0.208-5.114) | 0.9691 |
| T3&T4 | 145 | 1.684(1.042-2.721) | **0.0340** |
| N stage |  |  |  |
| N0 | 50 | 1.482(0.478-4.597) | 0.4974 |
| N1 | 123 | 1.206(0.727-2.001) | 0.4679 |
| M stage |  |  |  |
| M0 | 79 | 1.679(0.871-3.235) | 0.1319 |
| M1 | 5 | - | - |
| Radiation therapy |  |  |  |
| No | 118 | 2.146(1.257-3.665) | **0.0058** |
| Yes | 45 | 1.382(0.439-4.351) | 0.5719 |
| Primary therapy outcome |  |  |  |
| PD&SD | 58 | 1.333(0.728-2.443) | 0.3425 |
| PR&CR | 81 | 2.565(1.040-6.326) | **0.0463** |
| Gender |  |  |  |
| Female | 80 | 1.319(0.692-2.513) | 0.3983 |
| Male | 98 | 2.599(1.345-5.023) | **0.0047** |
| Race |  |  |  |
| White | 157 | 2.091(1.280-3.414) | **0.0029** |
| Asian&Black or African American | 17 | 0.934(0.168-5.204) | 0.9334 |
| Age |  |  |  |
| <=65 | 93 | 2.801(1.496-5.241) | **0.0012** |
| >65 | 85 | 1.248(0.631-2.469) | 0.5237 |
| Residual tumor |  |  |  |
| R0 | 107 | 1.916(1.014-3.623) | **0.0477** |
| R1&R2 | 57 | 1.505(0.724-3.128) | 0.2656 |
| Histologic grade |  |  |  |
| G1&G2 | 126 | 2.084(1.180-3.683) | **0.0113** |
| G3&G4 | 50 | 1.276(0.577-2.824) | 0.5382 |
| Anatomic neoplasm subdivision |  |  |  |
| Head of Pancreas | 138 | 1.432(0.871-2.357) | 0.1593 |
| Other | 40 | 6.330(1.908-21.000) | **0.0062** |

Total patients’ number does not equal to 178 in all variates due to lack of patient’s information for some cases. CR, complete response; FNDC3B, fibronectin type III domain containing 3B; G1: well-differentiated; G2: moderately-differentiated; G3: poorly-differentiated; G4: undifferentiated; M: metastasis; N: lymph node; PC, pancreatic cancer; PR, partial response; PD, progressive disease; SD, stable disease; T: tumor. Bold values indicate that P values <0.05 which are statistically significant.
