# Supplementary material for: Identification of fibronectin type III domain containing 3B as a potential prognostic and therapeutic target for pancreatic cancer: a preliminary analysis
Source: Eur J Med Res. 2024 Apr 5;29:221. doi: 10.1186/s40001-024-01823-6 (PMC10996089; doi:10.1186/s40001-024-01823-6)
Supplement: Supplementary file 7 — Additional file 7: Table S6. The prognostic value of FNDC3B (Progress Free Interval) in various PC subgroups. [file 40001_2024_1823_MOESM7_ESM.docx]

**Table S6.** The prognostic value of FNDC3B (Progress Free Interval) in various PC subgroups

| Characteristics | N (%) | HR (95% CI) | P value |
| --- | --- | --- | --- |
| T stage |  |  |  |
| T1&T2 | 31 | 1.844(0.594-5.722) | 0.3040 |
| T3&T4 | 145 | 1.461(0.972-2.198) | 0.0640 |
| N stage |  |  |  |
| N0 | 50 | 1.101(0.518-2.340) | 0.8022 |
| N1 | 123 | 1.190(0.763-1.855) | 0.4424 |
| M stage |  |  |  |
| M0 | 79 | 1.189(0.691-2.045) | 0.5312 |
| M1 | 5 | - | - |
| Radiation therapy |  |  |  |
| No | 118 | 1.741(1.092-2.775) | **0.0195** |
| Yes | 45 | 1.514(0.697-3.286) | 0.2745 |
| Primary therapy outcome |  |  |  |
| PD&SD | 58 | 0.969(0.562-1.670) | 0.9090 |
| PR&CR | 81 | 3.402(1.719-6.733) | **0.0004** |
| Gender |  |  |  |
| Female | 80 | 1.467(0.833-2.583) | 0.1851 |
| Male | 98 | 1.827(1.081-3.086) | **0.0180** |
| Race |  |  |  |
| White | 157 | 1.832(1.210-2.774) | **0.0032** |
| Asian&Black or African American | 17 | 0.634(0.178-2.261) | 0.4370 |
| Age |  |  |  |
| <=65 | 93 | 2.864(1.673-4.906) | **4.84e-05** |
| >65 | 85 | 1.007(0.575-1.763) | 0.9810 |
| Residual tumor |  |  |  |
| R0 | 107 | 2.073(1.215-3.538) | **0.0081** |
| R1&R2 | 57 | 0.927(0.506-1.698) | 0.8038 |
| Histologic grade |  |  |  |
| G1&G2 | 126 | 1.747(1.091-2.798) | **0.0176** |
| G3&G4 | 50 | 1.753(0.880-3.491) | 0.0842 |
| Anatomic neoplasm subdivision |  |  |  |
| Head of Pancreas | 138 | 1.284(0.841-1.959) | 0.2432 |
| Other | 40 | 3.945(1.541-10.099) | **0.0017** |

Total patients’ number does not equal to 178 in all variates due to lack of patient’s information for some cases. CR, complete response; FNDC3B, fibronectin type III domain containing 3B; G1: well-differentiated; G2: moderately-differentiated; G3: poorly-differentiated; G4: undifferentiated; M: metastasis; N: lymph node; PC, pancreatic cancer; PR, partial response; PD, progressive disease; SD, stable disease; T: tumor. Bold values indicate that P values <0.05 which are statistically significant.
