# Supplementary material for: Identification of fibronectin type III domain containing 3B as a potential prognostic and therapeutic target for pancreatic cancer: a preliminary analysis
Source: Eur J Med Res. 2024 Apr 5;29:221. doi: 10.1186/s40001-024-01823-6 (PMC10996089; doi:10.1186/s40001-024-01823-6)
Supplement: Supplementary file 8 — Additional file 8: Table S7. Univariate and multivariate Cox analysis (Disease Specific Survival) of prognostic covariates in patients with PC. [file 40001_2024_1823_MOESM8_ESM.docx]

**Table S7.** Univariate and multivariate Cox analysis (Disease Specific Survival) of prognostic covariates in patients with PC.

| Characteristics | Total(N) | Univariate analysis | | Multivariate analysis | |
| --- | --- | --- | --- | --- | --- |
|  |  | Hazard ratio (95% CI) | *P* value | Hazard ratio (95% CI) | *P* value |
| T stage (T3&T4 vs. T1&T2) | 170 | 3.177 (1.372-7.359) | **0.007** | 56715262.6184 (0.000-Inf) | 0.997 |
| N stage (N1 vs. N0) | 167 | 2.699 (1.449-5.029) | **0.002** | 2.841 (0.584-13.811) | 0.196 |
| M stage (M1 vs. M0) | 81 | 1.250 (0.296-5.276) | 0.762 |  |  |
| Pathologic stage (Stage II&Stage III&Stage IV vs. Stage I) | 169 | 3.312 (1.199-9.149) | **0.021** | 0.000 (0.000-Inf) | 0.997 |
| Radiation therapy (Yes vs. No) | 157 | 0.438 (0.234-0.819) | **0.010** | 0.204 (0.059-0.710) | **0.012** |
| Primary therapy outcome (CR&PR vs. PD&SD) | 136 | 0.283 (0.164-0.489) | **< 0.001** | 0.324 (0.129-0.812) | **0.016** |
| Age (>65 vs. ≤ 65) | 172 | 1.082 (0.680-1.719) | 0.740 | 1.124 (0.463-2.732) | 0.796 |
| Race (White vs. Asian&Black or African American) | 169 | 1.452 (0.628-3.354) | 0.383 | 5.776 (0.680-49.099) | 0.108 |
| Gender (Male vs. Female) | 172 | 1.301 (0.821-2.060) | 0.262 | 0.832 (0.346-2.000) | 0.681 |
| Histologic grade (G3&G4 vs. G1&G2) | 170 | 1.588 (0.979 - 2.578) | 0.061 | 2.273 (0.946-5.465) | 0.067 |
| Residual tumor (R1&R2 vs. R0) | 158 | 1.849 (1.135-3.011) | **0.014** | 0.861 (0.361-2.054) | 0.736 |
| Anatomic neoplasm subdivision (Other vs. Head of Pancreas) | 172 | 0.456 (0.239-0.871) | **0.017** | 1.649 (0.390-6.978) | 0.497 |
| FNDC3B (High vs. Low) | 172 | 2.168 (1.344-3.498) | **0.002** | 2.209 (0.821-5.946) | 0.117 |

Total patients’ number does not equal to 178 in all variates due to lack of patient’s information for some cases. CR, complete response; FNDC3B, fibronectin type III domain containing 3B; G1: well-differentiated; G2: moderately-differentiated; G3: poorly-differentiated; G4: undifferentiated; M: metastasis; N: lymph node; PC, pancreatic cancer; PR, partial response; PD, progressive disease; SD, stable disease; T: tumor. Bold values indicate that P values <0.05 which are statistically significant.
