# Supplementary material for: Identification of fibronectin type III domain containing 3B as a potential prognostic and therapeutic target for pancreatic cancer: a preliminary analysis
Source: Eur J Med Res. 2024 Apr 5;29:221. doi: 10.1186/s40001-024-01823-6 (PMC10996089; doi:10.1186/s40001-024-01823-6)
Supplement: Supplementary file 9 — Additional file 9: Table S8. Univariate and multivariate Cox analysis (Progress Free Interval) of prognostic covariates in patients with PC. [file 40001_2024_1823_MOESM9_ESM.docx]

**Table S8.** Univariate and multivariate Cox analysis (Progress Free Interval) of prognostic covariates in patients with PC

| Characteristics | Total(N) | Univariate analysis | | Multivariate analysis | |
| --- | --- | --- | --- | --- | --- |
|  |  | Hazard ratio (95% CI) | *P* value | Hazard ratio (95% CI) | *P* value |
| T stage (T3&T4 vs. T1&T2) | 176 | 2.414 (1.309-4.451) | **0.005** | 2.648 (0.322-21.755) | 0.365 |
| N stage (N1 vs. N0) | 173 | 1.767 (1.127-2.771) | **0.013** | 1.859 (0.603-5.738) | 0.281 |
| M stage (M1 vs. M0) | 84 | 0.828 (0.257-2.667) | 0.751 | 0.403 (0.045 - 3.639) | 0.418 |
| Pathologic stage (Stage II&Stage III&Stage IV vs. Stage I) | 175 | 2.965 (1.354-6.492) | **0.007** | 0.180 (0.014-2.369) | 0.192 |
| Radiation therapy (Yes vs. No) | 163 | 0.741 (0.472-1.163) | 0.192 | 0.327 (0.129 - 0.828) | **0.018** |
| Primary therapy outcome (CR&PR vs. PD&SD) | 140 | 0.335 (0.215-0.521) | **< 0.001** | 0.326 (0.154-0.690) | **0.003** |
| Age (>65 vs. ≤ 65) | 178 | 1.240 (0.837-1.837) | 0.284 | 1.054 (0.509-2.183) | 0.886 |
| Race (White vs. Asian&Black or African American) | 174 | 1.025 (0.532-1.974) | 0.941 | 4.350 (0.890-21.275) | 0.069 |
| Gender (Male vs. Female) | 178 | 1.027 (0.698-1.510) | 0.894 | 0.572 (0.274-1.194) | 0.137 |
| Histologic grade (G3&G4 vs. G1&G2) | 176 | 1.671 (1.106-2.526) | **0.015** | 3.423 (1.555-7.531) | **0.002** |
| Residual tumor (R1&R2 vs. R0) | 164 | 2.270 (1.506-3.420) | **< 0.001** | 2.123 (0.995-4.527) | 0.051 |
| Anatomic neoplasm subdivision (Other vs. Head of Pancreas) | 178 | 0.481 (0.287-0.806) | **0.005** | 1.172 (0.395-3.476) | 0.774 |
| FNDC3B (High vs. Low) | 178 | 1.822 (1.223-2.716) | **0.003** | 1.545 (0.686-3.476) | 0.293 |

Total patients’ number does not equal to 178 in all variates due to lack of patient’s information for some cases. CR, complete response; FNDC3B, fibronectin type III domain containing 3B; G1: well-differentiated; G2: moderately-differentiated; G3: poorly-differentiated; G4: undifferentiated; M: metastasis; N: lymph node; PC, pancreatic cancer; PR, partial response; PD, progressive disease; SD, stable disease; T: tumor. Bold values indicate that P values <0.05 which are statistically significant.
